# Supplementary material for: Chemoenzymatic synthesis of genetically-encoded multivalent liquid N-glycan arrays
Source: Nat Commun. 2023 Aug 28;14:5237. doi: 10.1038/s41467-023-40900-y (PMC10462762; doi:10.1038/s41467-023-40900-y)
Supplement: Supplementary file 1 — Supplementary Information [file 41467_2023_40900_MOESM1_ESM.pdf]

Supplementary Information

Chemoenzymatic Synthesis of Genetically-Encoded Multivalent Liquid *N*-glycan Arrays

Chih-Lan Lin[1], Mirat Sojitra[1], Eric J. Carpenter[1], Ellen S. Hayhoe[1], Susmita Sarkar[1], Elizabeth A. Volker[1], Chao Wang[2], Duong T. Bui[1], Loretta Yang[3], John S. Klassen[1], Peng Wu[2], Matthew S. Macauley[1,4], Todd L. Lowary[1,5,6], and Ratmir Derda[1]

[1]Department of Chemistry, University of Alberta, Edmonton, AB T6G 2G2, Canada

[2]Department of Molecular Medicine, The Scripps Research Institute, 10550 N. Torrey Pines Road, La Jolla, CA 92037, USA

[3]Lectenz Bio, 111 Riverbend Rd, Athens, GA 30602, USA

[4]Department of Medical Microbiology and Immunology, University of Alberta, Edmonton, AB T6G 2E1, Canada

[5]Institute of Biological Chemistry, Academia Sinica, Taipei, Taiwan

[6]Institute of Biochemical Sciences, National Taiwan University, Taipei, Taiwan

*Corresponding author: ratmir@ualberta.ca

Table of Contents

1. Supplementary Methods 5
1.1. List of abbreviations 5
1.2. General Synthetic methods 5
1.3. Detailed information of LC-MS 6
1.4. Extraction, Isolation and Trimming of SGP 6
1.5. Acylation of the asparagine amino group with 8-azido-octanoic acid 7
1.6. Expression and purification of enzymes 9
1.7. One-step PCR protocol 10
1.8. *Two-step PCR protocol* 10
1.9. Procedures for enzymatic reactions on the phage surface 11
Supplementary Table. 1: The *in vitro* deep-sequencing data used in this paper. 14
Supplementary Table. 2: URL for deep-sequencing data used in this paper. 15

Supplementary Figure. 1: LC-MS analysis of isolated SGP 1. 16
Supplementary Figure. 2: LC-MS results of pronase treated SGP **2** after purification...... 17
Supplementary Figure. 3: LC-MS results after neuraminidase treatment of **2**, giving **3**. .. 18
Supplementary Figure. 4: LC-MS results after β-galactosidase treatment of **3**, giving **4**. . 19
Supplementary Figure. 5: LC-MS results of modification of **4** with 8-azido-octanoic acid to give **6**. 20
Supplementary Figure. 6: LC-MS results modification of **2** with 8-azido-octanoic acid to give **7**. 21
Supplementary Fig. 7: LC-MS results after B4GalT1-catalyzed reaction of mixtures **7**, giving **8**. 22
Supplementary Figure. 8: Optimization of MALDI-TOF of sialylated biantennary *N*-linked oligosaccharides **7** on phage. 23
Supplementary Figure. 9: On phage trimming of "homogeneous" N-linked terminal galactose structure. 24
Supplementary Figure. 10: Direct *β*-galactosidase treatment of SGP **7** to confirm the corresponding peak S2' are indeed "ghost" species. 25
Supplementary Figure. 11: Model study of Pd26ST-catalyzed sialylation on LacNAc-modified pVIII. 26
Supplementary Figure. 12: On-phage enzymatic trimming of biantennary glycosyl-asparagines to generate terminal mannose *N*-glycans ($Man_3GlcNAc_2$). 28
Supplementary Figure. 13: Model study of B4GalT1-catalyzed galactosylation of GlcNAc to generate LacNAc-modified pVIII. 29
Supplementary Figure. 14: On-phage B4GalT1-catalyzed galactosylation of biantennary N-glycans. 30
Supplementary Figure. 15: Model study of Pd26ST-catalyzed sialylation of lactose-modified pVIII and use unnatural CMP-sialic acid as a donor. 31
Supplementary Figure. 16: Model study of Pm2,3ST-catalyzed sialylation on LacNAc-modified pVIII. 32
Supplementary Figure 17. On-phage enzymatic extension of Gal-terminated N-glycan by Pm2,3ST and structure validation by binding to diCBM40 lectin. 34
Supplementary Figure. 18: Galactosidase digestion of incomplete Pd26ST-catalyzed sialylation on LacNAc-modified pVIII. 35
Supplementary Figure. 19: Comparison of the intensity before enzymatic glycan extension and after enzymatic glycan cleavage on pVIII. 36
Supplementary Figure. 20: Enzymatic extension of SGP glycan (by B4GalT1) displayed on phage at 5 different densities monitored by MALDI TOF MS. 37
Supplementary Figure. 21: Two steps enzymatic extension (by B4GalT1 and Pd26ST) of SGP glycan displayed on phage at 5 different densities monitored by MALDI TOF MS.... 38
Supplementary Figure. 22: On-phage of Hp1,3FT-catalyzed α-(1 →3) fucosylation of Gal-terminated N-glycan. 39
Supplementary Figure. 23: On-phage Hp1,3FT-catalyzed α-(1 →3) fucosylation by one pot two steps enzymatic strategy. 40

Supplementary Figure. 24: Binding of phage displayed *N*-glycans with α-(1 →3)-linked fucose. 41
Supplementary Figure. 25: Conjugation of SGP glycan to phage at 5 different densities as monitored by MALDI TOF MS. 42
Supplementary Figure. 26: Conjugation of *N*-glycan **10** to phage at 5 different densities as monitored by MALDI TOF MS. 43
Supplementary Figure. 27: Conjugation of *N*-glycan **6** to phage at 5 different densities as monitored by MALDI TOF MS. 44
Supplementary Figure. 28: On-phage enzymatic trimming of **6** to generate terminal mannose *N*-glycan ($Man_3GlcNAc_2$) at 5 different densities as monitored by MALDI TOF MS. 45
Supplementary Figure. 29: Binding of LiGA6×5 library to SNA-I. 46
Supplementary Figure. 30: Binding of LiGA6×5 library to CD22 (Siglec-2). 47
Supplementary Figure. 31: Binding of LiGA6×5 to SiaFind™ Pan-specific Lectenz® before and after remodeling by Pm2,3ST. 48
Supplementary Figure. 32: Binding of LiGA6×5 to SiaFind™ α-(2→6)-specific reagent before and after remodeling by Pm2,3ST. 50
Supplementary Figure. 33: Binding of LiGA6×5 to sialic acid-specific diCBM40 lectin before and after remodeling by Pm2,3ST. 52
Supplementary Figure. 34: Binding of LiGA6×5 library to ConA. 54
Supplementary Figure. 35: Binding of LiGA6×5 library to LCA. 55
Supplementary Figure. 36: Binding of LiGA6×5 library to PSA. 56
Supplementary Figure. 37: Binding of LiGA6×5 library to GNL (GNA). 57
Supplementary Figure. 38: Binding of LiGA6×5 library to RCA-I. 58
Supplementary Figure. 39: Binding of LiGA6×5 library to ECL. 59
Supplementary Figure. 40: Binding of LiGA6×5 library to WGA. 61
Supplementary Figure. 41: MALDI-TOF MS results of five phage conjugates for the study of steric occlusions. 62
Supplementary Figure. 42: Steric occlusions in the interaction between lectins and *N*-glycans on phage. 63
Supplementary Figure. 43: Binding measurements of each *N*-glycan with 6 lectins by Mass- spectrometry. 65
Supplementary Figure. 44: Titration curve data measured by Mass Spectrometry. 66
Supplementary Figure. 46: Binding of ConA to MSDB-paucimannose conjugates and LiGA6×5 69
Supplementary Figure. 47: Comparison of LiGA-mixtures with and without binding paucimannose *N*-glycans. 71
Supplementary Figure. 48: Binding of GNL by to MSDB-phage paucimannose conjugates and LiGA6×5. 72
Supplementary Figure. 49: Binding of WGA by to MSDB-paucimannose conjugates and LiGA6×5 73

Supplementary Figure. 50: Summary of LiGA interaction with spleen *in-vivo* described as fold change enrichment in spleen with respect to plasma from the same animal. 74
Supplementary Figure. 51: Summary of LiGA interaction with liver *in-vivo* described as fold change (FC) enrichment in liver with respect to plasma from the same animal. 75
Supplementary Figure. 52: Summary of LiGA interaction with left kidney *in-vivo* described as fold change (FC) enrichment in left kidney with respect to plasma from the same animal. 76
Supplementary Figure. 53: Summary of LiGA interaction with right kidney *in-vivo* described as fold change (FC) enrichment in right kidney with respect to plasma from the same animal. 77
Supplementary Figure. 54: Summary of LiGA interaction with heart *in-vivo* described as fold change (FC) enrichment in heart with respect to plasma from the same animal. 78
Supplementary Figure. 55: Summary of LiGA interaction with lungs *in-vivo* described as fold change (FC) enrichment in lungs with respect to plasma from the same animal. 79
Supplementary Figure. 56: Summary of glycans enriched in liver compared to plasma... 80
Supplementary Figure. 57: Summary of clonal binding of 6 glycans to HepG2 cells that expressed ASGPR. 81
Supplementary Figure. 58: Comparison of "on-phage" enzymatic synthesis of liquid glycan array 82
Supplementary References.. 83

1. Supplementary Methods

1.1. List of abbreviations

BSA	Bovine Serum Albumin
B4GalT1	Beta-1,4-Galactosyltransferase 1
CHO	Chinese Hamster ovary [cells]
CD22	Cluster of Differentiation-22, also called SIGLEC-2
ConA	Concanavalin A
DBCO	dibenzylcyclooctyne
DNA	Deoxyribonucleic acid
DMEM	Dulbecco's Modified – Eagle's Medium
ECL	*Erythrina cristagalli* lectin
EDTA	ethylenediaminetetraacetic acid
ELISA	Enzyme linked immuno-assay
FACS	Fluorescence Activated Cell Sorting
FC	Fold Change – ratio of read counts
GNL	*Galanthus nivalis* Lectin
HEPES	Hydroxyethyl piperazineethanesulfonic acid
HRPO	Horseradish peroxidase
LiGA	Liquid Glycan Array – mixture of glycan labelled phages
LB	Lysogeny Broth
LCA	*Lens culinaris* agglutinin
MALDI-TOF	Matrix-assisted Laser Desorption/Ionization-Time of Flight
MS	Mass Spectrometry
MWCO	Molecular Weight Cutoff
NHS	N-hydroxysuccinimide
PBS	Phosphate Buffered Saline
PCR	Polymerase Chain Reaction
Pd26ST	*Photobacterium damselae* α-2,6-sialyltransferase

1.2. General Synthetic methods

All reagents were purchased from commercial sources and used without further purification. Oven-dried glassware was used for all reactions. Reaction solvents were dried by passage through columns of alumina and copper under nitrogen. All reactions, unless stated otherwise, were carried out at rt under positive pressure of argon. Organic solutions were concentrated under vacuum below 40 °C using a rotary evaporator. Reaction progress was monitored by TLC on Silica Gel 60 F_{254} (0.25 mm, E. Merck). Visualization of the TLC spots was done either under UV light or charring TLC plates with acidified *p*-anisaldehyde solution in ethanol or phosphomolybdic acid stain. Column chromatography was performed using silica gel 40–60 µM. ^{1}H NMR spectra were recorded using 500 MHz or 400 MHz instruments, and the data are reported as if they were first order. ^{13}C NMR (APT) spectra were recorded at 125 MHz.

1.3. Detailed information of LC-MS

For the detection of products, reverse phase high performance liquid chromatography followed by detection using MS (RP-HPLC-MS) was performed using an Agilent 1200 SL HPLC System with a Phenomenex Luna Omega Polar C18, 1.6 µm, 100 Å, 2.1x50 mm column (Phenomenex, Torrance, USA) with guard thermostated at 50 °C. A buffer gradient system composed 0.1% formic acid in water as mobile phase A and 0.1% formic acid in acetonitrile (ACN) as mobile phase B was used. An aliquot of 2 µL of sample was loaded onto the column at a flow rate of 0.5 $mLmin^{-1}$ and an initial buffer composition of 100% mobile phase A and 0% mobile phase B. After injection, the column was washed using the initial loading conditions for 1 min to effectively remove salts. Elution of the analytes was done by using a linear gradient from 0% to 98% mobile phase B over a period of 5.0 min, then held at 98% mobile phase B for 1.5 min and then returned to the initial condition over 0.5 min. Mass spectra were acquired in positive mode of ionization using an Agilent 6220 Accurate-Mass TOF HPLC/MS system (Santa Clara, CA, USA) equipped with a dual sprayer electrospray ionization source with the second sprayer providing a reference mass solution. Mass correction was performed for every individual spectrum using peaks at m/z 121.0509 and 922.0098 from the reference solution. Mass spectrometric conditions were drying gas 10 L/min at 325 °C, nebulizer 30 psi, mass range 100-3200 Da, acquisition rate of ~1.03 spectra/sec, fragmentor 175 V, skimmer 65 V, capillary 4000 V, instrument state 4 GHz High Resolution. Data analysis was performed using the Agilent Mass Hunter Qualitative Analysis software package version B.07.00 SP2. For High Resolution Mass Spectrometry (HRMS), the electrospray ionization spectra were recorded on an Agilent Technologies 6220 TOF spectrometer with samples dissolved in CH_3OH or H_2O.

1.4. Extraction, Isolation and Trimming of SGP

Sialylated Biantennary Glycopeptide (SGP) Extraction

SGP **1** was extracted according to reported procedures[1]. Commercially available egg yolk powder (100 g, Rembrandt Foods, Inc) was suspended twice in 95% ethanol (1.5 L) and stirred for 2 h. at rt to extract lipids and other organic soluble components. The suspension was filtered, dried via vacuum filtration and the filtrate was discarded to yield an off-white, dry powder. To extract SGP, the powder was suspended twice in aqueous ethanol (40% v/v ethanol, 1.5 L) solution and stirred for 2 h at rt. The insoluble material was discarded and the filtrate was concentrated under reduced pressure at 40 °C. Cold aqueous ethanol (40% v/v ethanol, 0.5 L) was added to the concentrated solution to precipitate proteins, which were removed by centrifugation (3000 rpm, 10 min). The resulting translucent liquid was purified on an active carbon / Celite column (50 g of active carbon and 50 g Celite). Impurities were removed by flushing the column with 100 mL of water (0.1% v/v TFA), 100 mL L of 5% acetonitrile in water (0.1% v/v TFA), and 100 mL 10% acetonitrile in water (0.1% v/v TFA). The SGP was eluted from the column using a solution of 25% acetonitrile in water (0.1% v/v TFA), and fractions containing the product were collected and concentrated under reduced pressure. The resulting white powder was subjected to size-exclusion chromatography (Sephadex™ G-25 Superfine Gel, fine particle size 15–88 µm, column dimensions 5.0 cm x 80 cm, 250 mL fractions) eluting with 0.1 M ammonium bicarbonate to yield SGP **1** as white powder (0.75 mg SGP/g egg yolk powder).

Trimming and Modification of SGP 1

The procedures used followed a previous report[1]. Isolated SGP **1** (20 mg) was dissolved in 5 mL Tris buffer (100 mM, pH 8.0) containing 5 mM $CaCl_2$. Pronase (Conc. 2 mg/100 μL) from *Streptomyces griseus* (Roche # 10165921001-1G) was added, and the reaction was incubated for 5 days at 37 °C with shaking. The mixture was heated at 80 °C for 20 min followed by Pronase removal using an Amicon Ultra-10 (MWCO-10k) centrifugal filter. The filtrate was lyophilized and purified by size exclusion chromatography (Sephadex™ G-25 Superfine Gel, fine particle size 15–88 μm), eluting with a 0.1 M ammonium bicarbonate. The fractions containing the glycosylated asparagine **2** (7 mg) were collected and lyophilized.

The glycosylated asparagine **2** (7 mg) was dissolved in 1 mL of sodium acetate buffer (50 mM, pH 5.5) containing 5 mM $CaCl_2$ and treated with neuraminidase from *Clostridium perfringens* (Sigma–Aldrich, 10 μL, 0.2 units). The reaction was incubated at 37 °C with shaking and monitoring by TLC (*i*PrOH:NH_4OH:H_2O = 4:3:1). After 4 h, the mixture was concentrated under reduced pressure and purified by size exclusion chromatography (Sephadex™ G-25 Superfine Gel), eluting with a 0.1 M ammonium bicarbonate. The fractions containing the mixture of symmetric and asymmetric products (3.2 mg) were collected and lyophilized. Products were further dissolved in 0.5 mL of sodium acetate buffer (50 mM, pH 4.5) containing 5 mM $CaCl_2$ and *β*-galactosidase (30 μL, 5 units) from *Aspergillus niger* (Sigma-Aldrich) was added to the reaction mixture. The reaction was incubated at 37 °C. The reaction was monitored by TLC (*i*PrOH:NH_4OH:H_2O = 4:3:1). After 3 h, the enzymes were removed using an Amicon Ultra-10 (MWCO-10k) centrifugal filter. The filtrate was concentrated under reduced pressure and purified by size exclusion chromatography (Sephadex™ G-25 Superfine Gel), eluting with a 0.1 M ammonium bicarbonate. The fractions containing the desired core structure (3.0 mg) was collected and lyophilized. The results were confirmed by LC-MS analysis.

1.5. Acylation of the asparagine amino group with 8-azido-octanoic acid

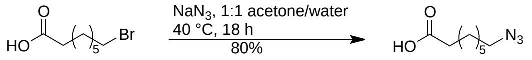

To a stirred solution of 8-bromooctanoic acid (2.00 g, 8.96 mmol) in acetone–water (1:1, 5 mL) was added sodium azide (1.16 g, 17.8 mmol). The solution was heated to 40 °C and stirred for 18 h. After cooling to rt, EtOAc (10 mL) was added and the organic phase separated from the aqueous phase. The aqueous phase was extracted with EtOAc (3 × 10 mL) and the organic phase and extractions were combined, washed with water (5 mL) and brine (5 mL), before being dried ($MgSO_4$) and filtered. The filtrate was concentrated to give 8-azidooctanoic acid as a colourless oil (1.28 g, 80%). R_f 0.5 (hexane:EtOAc = 3:1). This procedure was adapted from the reported literature[2]. HR ESIMS: *m/z* [M–H$^-$] calcd for $C_8H_{14}N_3O_2$: 184.1092. Found: 184.1093.

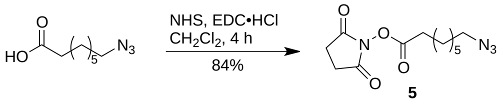

1-Ethyl-3-(3-dimethylaminopropyl)carbodiimide (EDC, 1.59 g, 8.3 mmol) was added to a suspension of 8-azidooctanoic acid (1.28 g, 6.9 mmol) and *N*-hydroxysuccinimide (NHS, 0.95 g, 8.3 mmol) in CH_2Cl_2 (35 mL) at rt, and the reaction was monitored by TLC (EtOAc–hexane, 1:3). After 5 h, 1 N HCl was added and the organic layer was separated, washed with saturated aqueous sodium bicarbonate, water, dried over sodium sulfate and filtered. The filtrate was concentrated and the resulting residue was purified by column chromatography (EtOAc:hexane = 1:2) to give 8-azidooctanoic acid NHS ester (**5**) as a colorless oil (1.08 g, 84%). R_f 0.3 (hexane:EtOAc = 3:1). This procedures were followed according to a reported literature[2]. HR ESIMS: *m/z* [$M+Na^+$] calcd for $C_{12}H_{18}N_4NaO_4$: 305.1220. Found: 305.1228.

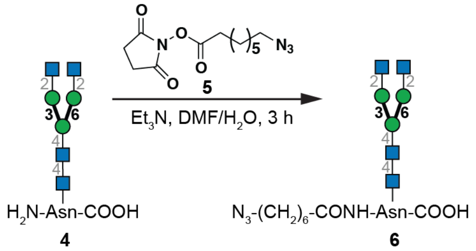

To a solution of **4** (2 mg, 0.001 mmol) in DMF (1.0 mL) and H_2O (added dropwise until the solution turned clear) at rt was added 8-azidooctanoic acid NHS ester **5** (1 mg, 0.004 mmol). Triethylamine was added dropwise to make ensure the reaction was basic by using pH test paper rolls. The reaction was monitored by TLC (*i*-PrOH:NH_4OH:H_2O = 4:3:1). After 3 h, the solution was concentrated and the residue was purified by size exclusion chromatography (Sephadex™ G-25 Superfine Gel). The fractions containing **6** (1.6 mg) (R_f = 0.6, *i*-PrOH:NH_4OH:H_2O = 4:3:1) were collected and lyophilized. The results were confirmed by LC-MS (Supplementary Fig. 5) and NMR analysis. Note: The reaction of NHS esters with amines is pH-dependent. At lower pH, the amino group is protonated, and no modification takes place. At higher-than-optimal pH, hydrolysis of the NHS ester is rapid and modification yield diminishes. The optimal pH value for this modification is 8.3–8.5. We have attempted the reaction in sodium bicarbonate buffer (pH = 8.5); however, acylation proceeded slowly and the NHS ester was hydrolyzed. The use of trimethylamine in DMF with a small amount of H_2O avoided these issues.

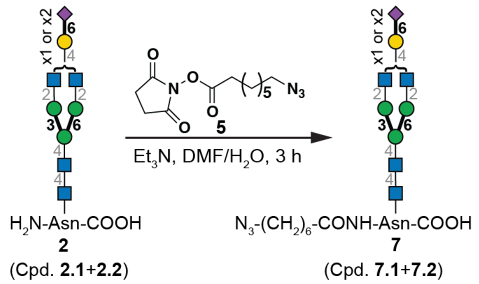

To a solution of heterogeneous *N*-glycans **2** (2 mg) in DMF (1.0 mL) and H_2O (added dropwise until the solution turned clear) at rt was added 8-azidooctanoic acid NHS ester **5** (1 mg, 0.004 mmol). Triethylamine was added dropwise to make sure the reaction was basic by pH test paper rolls. The reaction was monitored by TLC until the spots of starting material have consumed. After 3 h, the solution was concentrated, and the residue was purified by size exclusion chromatography (Sephadex™ G-25 Superfine Gel). The fractions containing the desired azido- *N*-glycans **7** (1.4 mg) was collected and lyophilized. The results were confirmed by LC-MASS (Supplementary Fig. 6).

1.6. Expression and purification of enzymes

Expression and purification of Histag-Pd26ST (*α*2→6 sialyltransferase) in *E. coli*

A truncated Pd26ST gene encoding for amino acid residues 16–497 of the full-length protein was cloned into a pET15b vector and expressed as a N-His6-tagged protein. LB broth powder (27.5 g) was added to MilliQ water (1.1 L) and the mixture was agitated until the powder completely dissolved. This LB solution (100 mL) was added to a clean baffled 500 mL Erlenmeyer flask and the remaining solution (1 L) was added to a clean baffled 4 L Erlenmeyer flask. The solutions in both flasks were sterilized by autoclaving for 15 min. Prior to inoculation, ampicillin (100 µL of a 100 µg/mL solution) was added to LB media (100 mL). Then, the plasmid-bearing *E. coli* strain was cultured in LB medium at 37 °C overnight with shaking at 200 rpm. Overexpression of the target protein was achieved by inducing the *E. coli* culture with 0.2 mM of isopropyl 1-thio-*β*-D-galactopyranoside (IPTG) when the OD_{600nm} of the culture reached 0.4–0.6 and incubating at 20 °C for 20 h with vigorous shaking at 250 rpm in an incubator shaker. The cell culture was centrifuged at 6,000 RPM (9000×g) (JLA8.1, Beckman Coulter) for 30 min. The cell pellet was resuspended in cold resuspension buffer (50 mM Tris-Cl, 0.2% Triton X-100, 10 mM Imidazole, 300 mM NaCl, pH 8.0). To the resuspended cell pellet protease inhibitor (What inhibitor) cocktail tablet was mixed. The cell suspension was passed through cell disruptor at 20,0000 PSI. The cell lysate was collected and kept at 4 °C. The cell lysate was then centrifuged at 36,000 rpm (320000×g) for 60 min (Ti45 rotor) and the supernatant was decanted and stored at 4 °C. The cell lysate containing the His-tagged Pd26ST was passed through pre-equilibrated (5x3 mL, 5 mM imidazole, 0.5 M NaCl, 50 mM Tris-HCl, pH 7.5) Ni-NTA superflow/agarose column. The column was washed (vol, 50 mM Tris-Cl, 20 mM imidazole, 300 mM NaCl, pH 8.0). The His tagged Pd26ST was eluted using elution buffer (vol, 50 mM Tris-Cl, 250 mM imidazole, 300 mM NaCl, pH 8.0). The fractions containing the protein (absorbance at A280) were combined and stored at -20 °C. The final yield was determined to be 1.29 mg/mL.

Recombinant expression of β-(1→4)-galactosyltransferase 1 (B4GalT1) in *E. coli*

Expression procedures: a single colony of freshly transformed MBPT-HP0826 in E. coli K12 TB1 was grown at 37 °C in LB containing 100 µg/ml ampicillin. After the overnight cultures were inoculated into the scale-up cultures (LB broth containing 100 µg/ml ampicillin, 0.2% glucose), the cells were induced with 0.3 mM IPTG at OD600 reaching around 0.5. The culture was incubated at 20 °C with shaking and after 22 h the cells harvested. The weight of the cell pellets was 12 g/L culture.

Purification procedures: The cell paste (1 L culture) was resuspended with 50 mM HEPES, pH 7.5 containing 1 mM EDTA and 300 mM NaCl supplemented with 1 EDTA free protease inhibitor cocktail. This was passed through a cell disruptor at 20,000 psi and the lysate collected on ice. The lysate was centrifuged at 40000 rpm (400000×g) for 1 h to remove cellular debris and membrane proteins. The supernatant was filtered with a Millex 0.22 um GV-13 filter and loaded onto a MBP-Trap HP affinity column (GE) and followed by the washing step until the UV profile returned to the baseline. The column was eluted with the elution buffer (50 mM HEPES, pH 7.5 containing 300 mM NaCl and 10 mM maltose). The eluted fractions were aliquoted and stored in a -80 °C freezer. The yield is 11.6 mg/L culture.

1.7. One-step PCR protocol

DNA template (phage solution) (15 μL) was amplified in Phusion® HF buffer with Phusion® High-Fidelity DNA Polymerase (NEB, #M0530S). Sequence of forward barcoded and reverse barcoded primers are provided in Supplementary Data. A typical 50 μL reaction mixture contained:

a) DNA template (phage solution).	15 μL
b) Phusion HF buffer (5x)	10 μL
c) Phusion® polymerase (1 unit)	0.5 μL
d) 10 mM dNTPs	1 μL
e) Forward barcoded primer (10 μM)	2.5 μL
f) Reverse barcoded primer (10 μM)	2.5 μL
g) Nuclease-free water	18.5 μL (for a total volume of 50 μL)

Exceptions: For amplification of clonal phages and naïve libraries, the volume of the template (phage solution) was 2 μL.

PCR cycles were performed using the following thermocycler settings:

a) 98 °C 3 min,
b) 98 °C 10 s,
c) 50 °C 20 s,
d) 72 °C 30 s,
e) repeat b)-d) for 10 cycles,
f) 98 °C 10 s,
g) 72 °C 30s,
h) repeat f)-g) for 20 cycles,
i) 72 °C 5 min, i) 4 °C hold

1.8. *Two-step PCR protocol*

<u>1st step PCR:</u>

a) DNA template (phage solution).	1 μL
b) DMSO	2 μL
c) Phusion HF buffer (5x)	10 μL
d) Phusion® polymerase (1 unit)	0.5 μL

e) 10 mM dNTPs 1 μL
f) NF10 primer (10 μM) 2.5 μL
g) -96 primer (10 μM) 2.5 μL
h) Nuclease-free water 30.5 μL (for a total volume of 50 μL)

PCR cycles were performed using the following thermocycler settings:

a) 98 °C 3 min,
b) 98 °C 10 s,
c) 50 °C 20 s,
d) 72 °C 20 s,
e) repeat b)-d) for 30 cycles,
f) 12 °C 1 min,
g) 4 °C hold

2nd step PCR:
a) PCR product from 1st step 5 μL
b) Phusion HF buffer (5x) 10 μL
c) Phusion® polymerase (1 unit) 0.5 μL
d) 10 mM dNTPs 1 μL
e) Forward barcoded primer (10 μM) 2.5 μL
f) Reverse barcoded primer (10 μM) 2.5 μL
g) Nuclease-free water 28.5 μL (for a total volume of 50 μL)

PCR cycles were performed using the following thermocycler settings:

a) 98 °C 3 min,
b) 98 °C 10 s,
c) 58 °C 30 s,
d) 72 °C 20 s,
e) repeat b)-d) for 20 cycles,
f) 12 °C 1 min,
g) 4 °C hold

1.9. Procedures for enzymatic reactions on the phage surface

Procedures for phage surface glycosidase treatment:

Cleavage of galactose in phage-displayed N-glycans by β-galactosidase
To a solution of phage modified with terminal galactose containing mixtures of *N*-glycans I1 and I2 in Fig. 4, 5% PEG-8000, 0.5 M NaCl (12 μL) was added and the solution was kept for 1.5 h at 0 °C. The solution was centrifuged at 21000×g for 10 min at rt. The supernatant was removed, and the pellet was resuspended in sodium acetate buffer (40 μL, 50 mM, pH 4.5 containing 5 mM $CaCl_2$) followed by the addition of *Aspergillus niger* β-galactosidase (2 μL, 0.47 units). The reaction was incubated at 37 °C and monitored by MALDI-TOF MS every 1 h. The reaction was

complete in 4 h to afford a symmetric biantennary GlcNAc-terminating *N*-glycan on the phage surface.

Cleavage of acetylglucosamine in phage-displayed N-glycans by β-N-acetylglucosaminidase S
To a solution of phage modified with the symmetric biantennary terminal GlcNAc *N*-glycan in Supplementary Fig. 9, 5% PEG-8000, 0.5 M NaCl (12 µL) was added and the solution was kept for 1.5 h at 0 °C. The solution was then centrifuged at 21000×g for 10 min at room temperature. The supernatant was removed, and the pellet was resuspended in nuclease free water (35 µL) and glycobuffer (4 µL, New England BioLabs Inc. #P0744S) followed by the addition of *Streptococcus pneumoniae β-N*-Acetylglucosaminidase S (1 µL, 4 units, New Englang BioLabs Inc. #P0744S). The reaction was incubated at 37 °C and monitored by MALDI-TOF MS. The reaction was complete in 1 h to afford mannose-terminating *N*-glycan on the phage surface.

Procedures for phage surface glycosylation:

Installation of α-(2→6)-linked sialic acid using Pd26ST
The conditions described below were used in the model study (Supplementary Fig. 11b). After modification of phage clones with LacNAc (β-Gal-(1→4)-β-GlcNAc-$OCH_2CH_2N_3$) to afford the conjugated phage (60 µL, ~10^{12}–10^{13} PFU/mL in PBS, pH 7.4), 5% PEG-8000, 0.5 M NaCl (12 µL) was added and the solution was kept for 1.5 h at 0 °C. After 1.5 h, the solution was centrifuged at 21000×g for 10 min at rt and supernatant was decanted. The pellet was resuspended in Tris-HCl buffer (25 µL, 100 mM with 20 mM $MnCl_2$, pH 8.5) containing CMP-Neu5Ac (300 µg), recombinant Shrimp Alkaline Phosphatase (rSAP, 0.5 µL) and Pd26ST (10 µL, 1.29 mg/mL). The reaction mixture was incubated at 37 °C and monitored by MALDI-TOF MS. Pd26ST (5 µL, 1.5 mg/mL) was added twice at 3 h and 6 h reaction time. The reaction was complete in 9 h and was validated by a β-galactosidase digestion experiment (Supplementary Fig. 11e, f).

Installation of β-(1→4)-linked galactose using B4GalT1
The condition described below was used in the model study (Supplementary Fig. 13). After modification of phage clones with *N*-Acetylglucosamine (β-GlcNAc-$OCH_2CH_2N_3$) to afford the conjugated phage (60 µL, ~10^{12}-10^{13} PFU/mL in PBS, pH 7.4), 5% PEG-8000, 0.5 M NaCl (12 µL) was added and the solution was kept for 1.5 h at 0 °C. After 1.5 h, the solution was centrifuged at 21000×g for 10 min at rt and the supernatant was decanted. The pellet was resuspended in HEPES buffer (20 µL, 50 mM with 10 mM $MnCl_2$, pH 7.5) containing UDP-Gal (100 µg), Shrimp Alkaline Phosphatase (1 µL) and B4GalT1 (15 µL, 0.048 mg/mL). The reaction mixture was incubated at 37 °C and monitored by MALDI-TOF MS. The reaction was complete in 40 h and was validated by MALDI-TOF MS.

Installation of α-(1→3)-linked fucose using Hp1,3FT
To a solution of phage modified with terminal galactose *N*-glycan **10** (50 µL, ~10^{12} PFU/mL in PBS, pH 7.4), 5% PEG-8000, 0.5 M NaCl (15 µL) was added and the solution was kept for 1.5 h at 0 °C. After 1.5 h, the solution was centrifuged at 21000×g for 10 min at rt and the supernatant was decanted. The pellet was resuspended in Tris-HCl buffer (20 µL, 50 mM with 10 mM $MnCl_2$, pH 7.5) containing GDP-Fuc (100 µg), Shrimp Alkaline Phosphatase (0.5 µL) and Hp1,3FT (2 µL,

1.5 mg/mL). The reaction mixture was incubated at 37 °C and monitored by MALDI-TOF MS. The reaction was complete in 1 h and was validated by MALDI-TOF MS (Supplementary Fig. 22).

Type	Test Dataset(s)	Columns	Control Dataset(s)	Columns
SNA 20 µg	20211216-87SCsnaBI-CT	1,2,3,4,5	20211216-87SCbsBI-CT<br>20220219-87SCbsBI-CT<br>20220219-87TCbsBI-CT	2,4,5<br>2,6,8,10,11,12,20<br>7,8,9
CD22 20 µg	20211216-87SCkhBI-CT	1,2,3,4,5	20211216-87SCbsBI-CT<br>20220219-87SCbsBI-CT<br>20220219-87TCbsBI-CT	2,4,5<br>2,6,8,10,11,12,20<br>7,8,9
ConA 20 µg	20211216-87SCcaBI-CT<br>20220219-87SCcaBI-CT	3,4,5<br>1,2,3,4,5	20211216-87SCbsBI-CT<br>20220219-87SCbsBI-CT<br>20220219-87TCbsBI-CT	2,4,5<br>2,6,8,10,11,12,20<br>7,8,9
LCA 20 µg	20220219-87SClaaBI-CT	1,2,3,4,5,6	20211216-87SCbsBI-CT<br>20220219-87SCbsBI-CT<br>20220219-87TCbsBI-CT	2,4,5<br>2,6,8,10,11,12,20<br>7,8,9
PSA 20 µg	20220219-87SCqbBI-CT	1,2,3,4,5	20211216-87SCbsBI-CT<br>20220219-87SCbsBI-CT<br>20220219-87TCbsBI-CT	2,4,5<br>2,6,8,10,11,12,20<br>7,8,9
GNL 20 µg	20220314-87SCgnlBI-CT	1,2,3,4,5	20211216-87SCbsBI-CT<br>20220219-87SCbsBI-CT<br>20220219-87TCbsBI-CT	2,4,5<br>2,6,8,10,11,12,20<br>7,8,9
ECL 10 µg	20220408-87SCeaaBI-CT	1,2,3,4,5	20220408-87SCbsBI-CT	1,2,3,4,5,6,7,8,9,10
ECL 20 µg	20220219-87SCeaaBI-CT	1,2,3,4,5	20211216-87SCbsBI-CT<br>20220219-87SCbsBI-CT<br>20220219-87TCbsBI-CT	2,4,5<br>2,6,8,10,11,12,20<br>7,8,9
RCA 1 µg	20220408-87SCrcaaBI-CT	1,2,3,4,5	20220408-87SCbsBI-CT	1,2,3,4,5,6,7,8,9,10
RCA 20 µg	20211216-87SCrcaaBI-CT	1,2,3,4,5	20211216-87SCbsBI-CT<br>20220219-87SCbsBI-CT<br>20220219-87TCbsBI-CT	2,4,5<br>2,6,8,10,11,12,20<br>7,8,9
G3C 50 µg	20220408-87SCgaBI-CT	6,7,8,9,10	20220408-87SCbsBI-CT	1,2,3,4,5,6,7,8,9,10
WGA 1 µg	20220314-87SCwgacBI-CT	1,2,3,4,5	20211216-87SCbsBI-CT<br>20220219-87SCbsBI-CT<br>20220219-87TCbsBI-CT	2,4,5<br>2,6,8,10,11,12,20<br>7,8,9
WGA 5 µg	20220314-87SCwgabBI-CT	1,2,3,4,5	20211216-87SCbsBI-CT<br>20220219-87SCbsBI-CT<br>20220219-87TCbsBI-CT	2,4,5<br>2,6,8,10,11,12,20<br>7,8,9
WGA 10 µg	20220314-87SCwgaaBI-CT	1,2,3,4,5	20211216-87SCbsBI-CT<br>20220219-87SCbsBI-CT<br>20220219-87TCbsBI-CT	2,4,5<br>2,6,8,10,11,12,20<br>7,8,9
WGA 20 µg	20220219-87SCwgaBI-CT	1,2,3,4,5,6,7,8,9,10,11	20211216-87SCbsBI-CT<br>20220219-87SCbsBI-CT<br>20220219-87TCbsBI-CT	2,4,5<br>2,6,8,10,11,12,20<br>7,8,9
CD22+ cells	20211216-87KEkhZW-MS	3,5	20211216-87KErbZW-MS	3,5
DC-SIGN+ cells	20220314-87SCrdZW-MS	1,2,3,4,5	20220314-87SCrfZW-MS	1,2,3,4,5

Supplementary Table. 1: The *in vitro* deep-sequencing data used in this paper.
The columns lists which column numbers were used. To access the data, enter the dataset name at https://48hd.cloud/ home page search bar.

Type	Test Dataset(s)	URL
SNA 20 µg	20211216-87SCsnaBI-CT	https://48hd.cloud/file/10131
CD22 20 µg	20211216-87SCkhBI-CT	https://48hd.cloud/file/10128
ConA 20 µg	20211216-87SCcaBI-CT<br>20220219-87SCcaBI-CT	https://48hd.cloud/file/10127<br>https://48hd.cloud/file/10331
LCA 20 µg	20220219-87SClaaBI-CT	https://48hd.cloud/file/10333
PSA 20 µg	20220219-87SCqbBI-CT	https://48hd.cloud/file/10335
GNL 20 µg	20220314-87SCgnlBI-CT	https://48hd.cloud/file/10599
ECL 10 µg	20220408-87SCeaaBI-CT	https://48hd.cloud/file/10892
ECL 20 µg	20220219-87SCeaaBI-CT	https://48hd.cloud/file/10332
RCA 1 µg	20220408-87SCrcaaBI-CT	https://48hd.cloud/file/10896
RCA 20 µg	20211216-87SCrcaaBI-CT	https://48hd.cloud/file/10130
G3C 50 µg	20220408-87SCgaBI-CT	https://48hd.cloud/file/10893
WGA 1 µg	20220314-87SCwgacBI-CT	https://48hd.cloud/file/10603
WGA 5 µg	20220314-87SCwgabBI-CT	https://48hd.cloud/file/10602
WGA 10 µg	20220314-87SCwgaaBI-CT	https://48hd.cloud/file/10601
WGA 20 µg	20220219-87SCwgaBI-CT	https://48hd.cloud/file/10336
CD22+ cells	20211216-87KEkhZW-MS	https://48hd.cloud/file/10175
DC-SIGN+ cells	20220314-87SCrdZW-MS	https://48hd.cloud/file/10661

Supplementary Table. 2: URL for deep-sequencing data used in this paper.
The files with DNA sequences and raw sequencing counts were uploaded to http://48hd.cloud/ server. Each experiment has a unique alphanumeric name and unique static URL that correspond to names in Supplementary Table. 1.

a

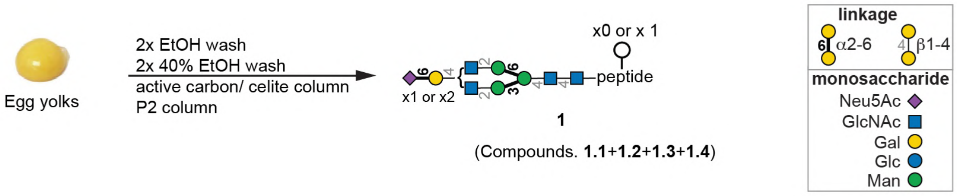

b

Compound table

Compound Label	RT	Mass	Abund	Formula	Tgt Mass	Diff (ppm)
Compound1.1:C95H162N14O57	2.42	2411.0174	8317	C95H162N14O57	2411.0208	-1.43
Compound1.2:C112H189N15O70	2.45	2864.1626	47419	C112H189N15O70	2864.1691	-2.25
Compound1.3:C101H172N14O62	2.45	2573.071	6892	C101H172N14O62	2573.0736	-1.01
Compound1.4:C118H199N15O7	2.47	3026.2159	6213	C118H199N15O75	3026.2219	-1.99

Compound 1.1

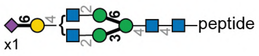

peptide = NH_2-Lys-Val-Ala-Asn-Lys-Thr-COOH

Compound 1.3

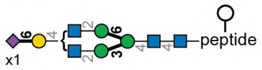

Compound 1.1	*m/z*	RT	Algorithm	Mass
$C_{95}H_{162}N_{14}O_{57}$	805.014	2.42	Find by Formula	2411.017

Compound 1.3	*m/z*	RT	Algorithm	Mass
$C_{101}H_{172}N_{14}O_{62}$	859.032	2.45	Find by Formula	2573.071

Compound Chromatograms

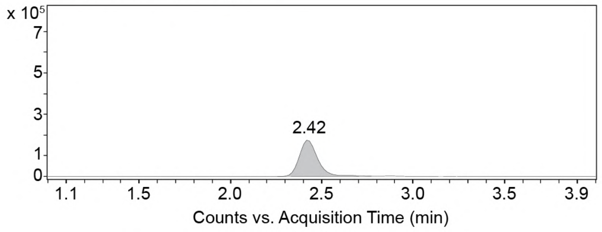

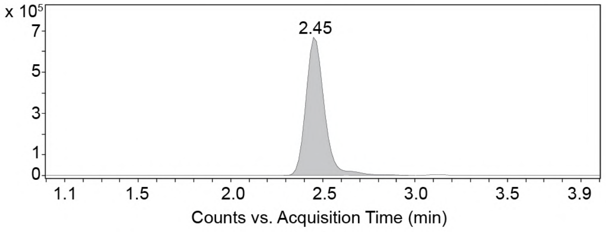

Compound 1.2

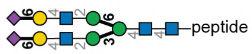

Compound 1.4

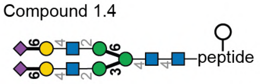

Compound 1.2	*m/z*	RT	Algorithm	Mass
$C_{112}H_{189}N_{15}O_{70}$	956.0627	2.45	Find by Formula	2864.1626

Compound 1.4	*m/z*	RT	Algorithm	Mass
$C_{118}H_{199}N_{15}O_{7}$	1010.0805	2.47	Find by Formula	3026.2159

Compound Chromatograms

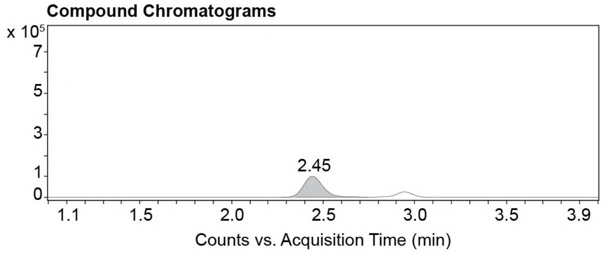

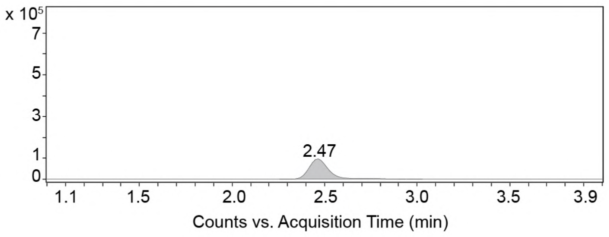

Supplementary Figure. 1: LC-MS analysis of isolated SGP 1.

a, Scheme of SGP production from egg yolk powder. **b,** Analytical LC-MS chromatogram of isolated SGP showing four components, Compounds 1.1–1.4 with different retention times.

a

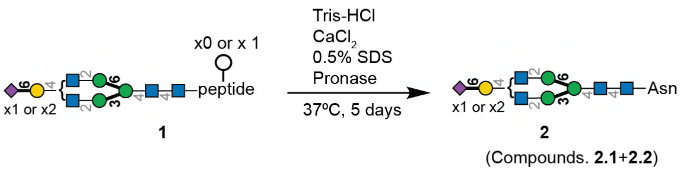

Compound table

Compound Label	RT	Mass	Abund	Formula	Tgt Mass	Diff (ppm)
Compound2.1:C88H144N8O64	6.08	2336.8233	1279	C88H144N8O64	2336.8259	1.11
Compound2.2:C71H117N7O51	5.09	1883.674	850	C71H117N7O51	1883.6777	1.95

b

Compound 2.1

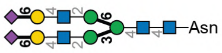

Compound 2.2

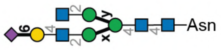

x, y = 3 or 6

Compound 2.1	*m/z*	RT	Algorithm	Mass
$C_{88}H_{144}N_8O_{64}$	780.2822	6.08	Find by Formula	2336.8233

Compound 2.2	*m/z*	RT	Algorithm	Mass
$C_{71}H_{117}N_7O_{51}$	942.8444	5.09	Find by Formula	1883.674

Compound Chromatograms

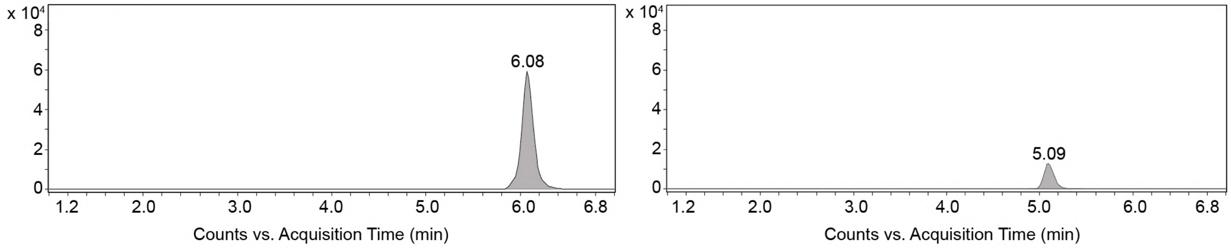

Supplementary Figure. 2: LC-MS results of pronase treated SGP **2** after purification.
a, Scheme for treating SGP **1** with pronase to trim the peptide down to Asparagine (Asn). **b,** LC-MS analysis of pronase treated SGP **2** showing two components with different retention times. Compound 2.1 is a homogeneous product with terminal sialic acid and Compound 2.2 is a mixture of heterogeneous structures.

a

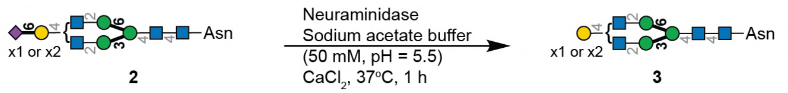

2 (Compounds. **2.1**+**2.2**) → **3** (Compounds. **3.1**+**3.2**)

b

Compound table

Compound Label	RT	Mass	Abund	Formula	Tgt Mass	Diff (ppm)
Compound3.1: C60H100N6O43	5.80	1592.5823	3741	C60H100N6O43	1592.5823	-0.02
Compound3.2: C66H110N6O48	6.04	1754.6327	12078	C66H110N6O48	1754.6351	-1.34

Compound 3.1

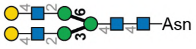

Compound 3.2

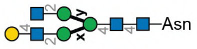

x, y = 3 or 6

Compound 3.1	*m/z*	RT	Algorithm	Mass
$C_{88}H_{144}N_8O_{64}$	797.2912	5.80	Find by Formula	1592.5823

Compound 3.2	*m/z*	RT	Algorithm	Mass
$C_{71}H_{117}N_7O_{51}$	878.3164	6.04	Find by Formula	1754.6327

Compound Chromatograms

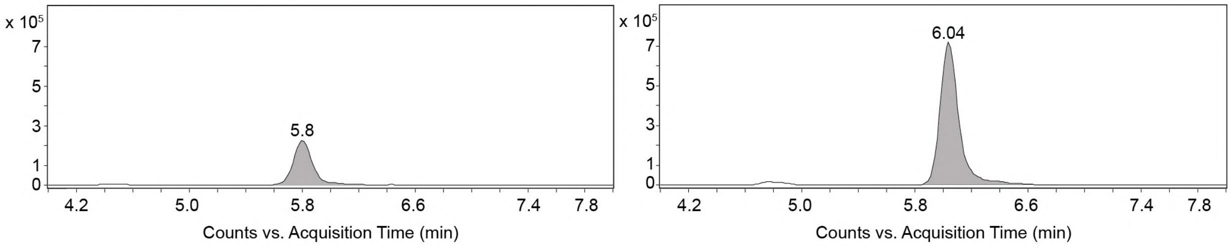

Supplementary Figure. 3: LC-MS results after neuraminidase treatment of **2**, giving **3**.
a, Scheme for cleaving the non-reducing end sialic acid in **2** by neuraminidase to give a heterogenous mixture **3** (Compounds 3.1 and 3.2). **b,** LC-MS analysis showing two components with different retention times.

a

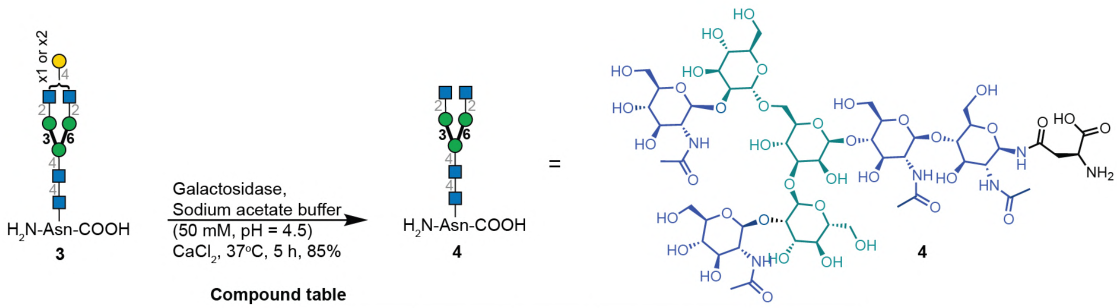

b

Compound table

Compound Label	m/z	RT	Algorithm	Mass
C54H90N6O38	716.2722	5.33	Find by Formula	1431.5368

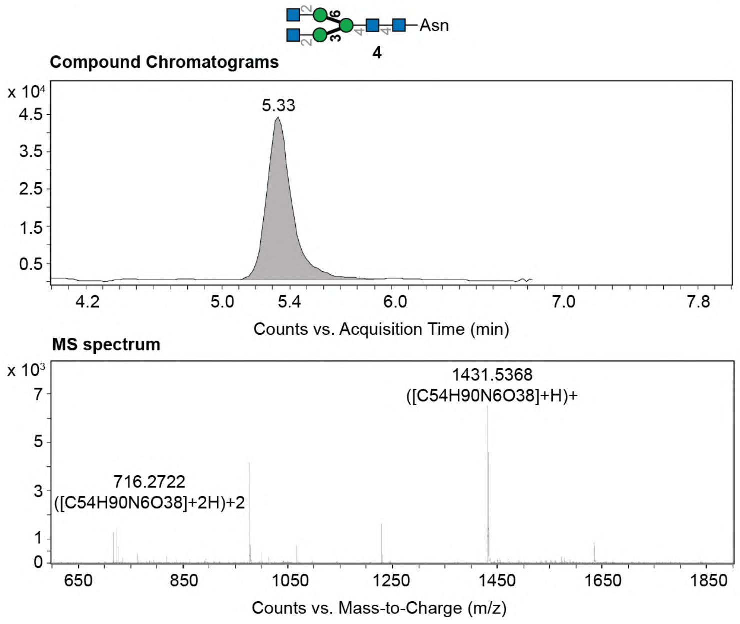

Supplementary Figure. 4: LC-MS results after β-galactosidase treatment of **3**, giving **4**.
a, Scheme for cleaving the non-reducing end galactose residues in **3** by β-galactosidase to give a homogenous biantennary *N*-glycan terminating in GlcNAc **3. b,** LC-MS result of compound **4**.

a

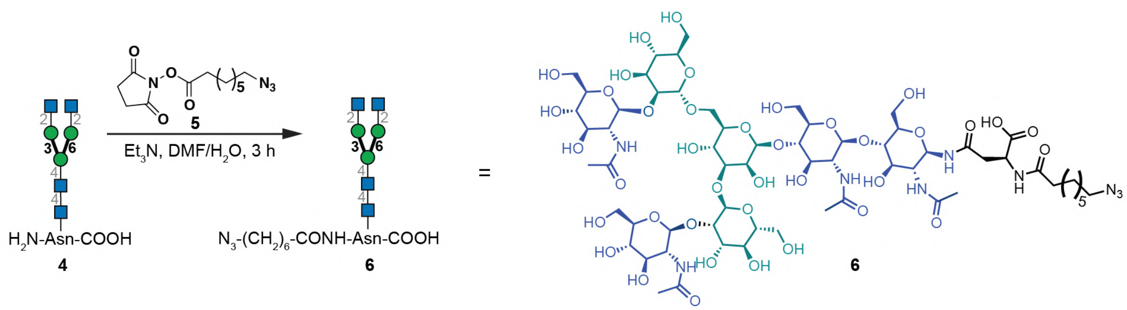

b

Compound table

Compound Label	m/z	Tgt Mass	Algorithm	Diff (ppm)
C62H103N9O39	1597.6365	1597.6365	Find by Formula	-0.76

MS spectrum

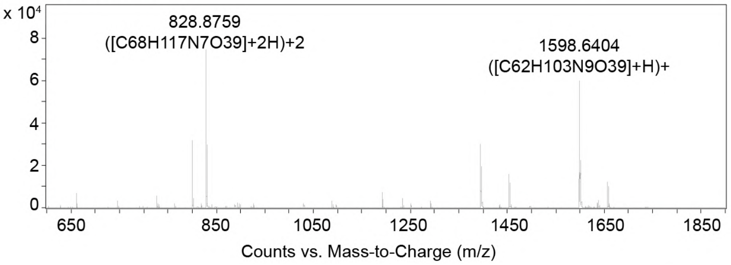

Supplementary Figure. 5: LC-MS results of modification of **4** with 8-azido-octanoic acid to give **6**.

a, Scheme for preparing **6** by reaction of **4** with **5**. **b,** LC-MS result of compound **6**.

a

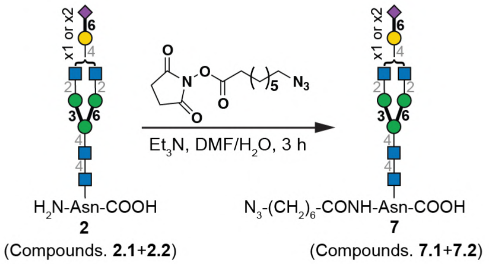

b

Compound table

Compound Label	RT	Mass	Abund	Formula	Tgt Mass	Diff (ppm)
Compound7.1: C96H157N11O65	3.54	2503.9273	54234	C96H157N11O65	2503.9318	-1.8
Compound7.2: C79H130N10O48	3.70	2050.7842	12104	C79H130N10O52	2050.7836	-0.34

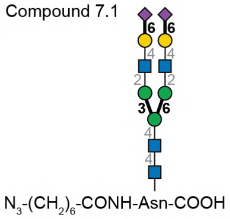

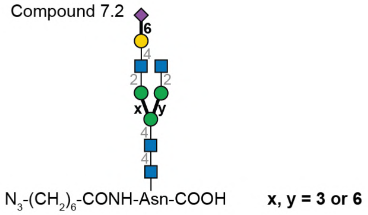

Compound 7.1	*m/z*	RT	Algorithm	Mass
$C_{96}H_{157}N_{11}O_{65}$	1253.4721	3.54	Find by Formula	2503.9273

Compound 7.2	*m/z*	RT	Algorithm	Mass
$C_{79}H_{130}N_{10}O_{52}$	1026.3994	3.70	Find by Formula	2050.7842

Compound Chromatograms

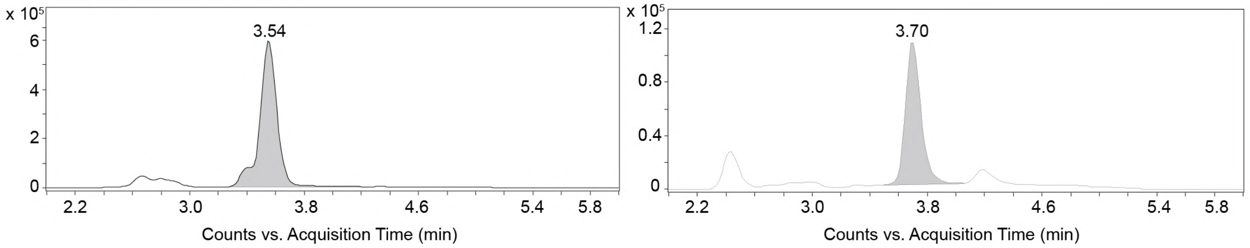

Supplementary Figure. 6: LC-MS results modification of **2** with 8-azido-octanoic acid to give **7**. **a,** Scheme for preparing **7** via reaction of **2** with **5** (procedure analogous to Supplementary Fig. 5). **b,** LC-MS analysis of Compounds 7.1+7.2 showing 2 components with different retention time.

a

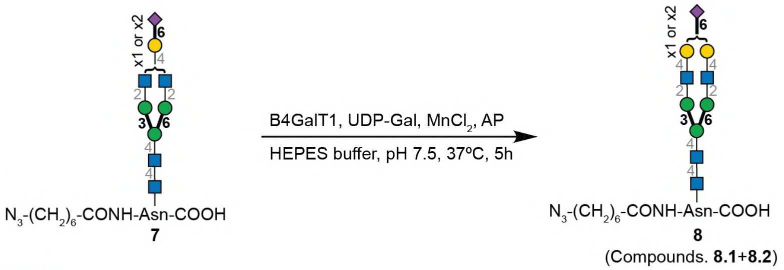

b

Compound table

Compound Label	RT	Mass	Abund	Formula	Tgt Mass	Diff (ppm)
Compound8.1: C96H157N11O65	5.94	2503.9295	4740	C96H157N11O65	2503.9318	-0.9
Compound8.2: C85H140N10O57	5.98	2212.8358	1642	C85H140N10O57	2212.8364	-0.26

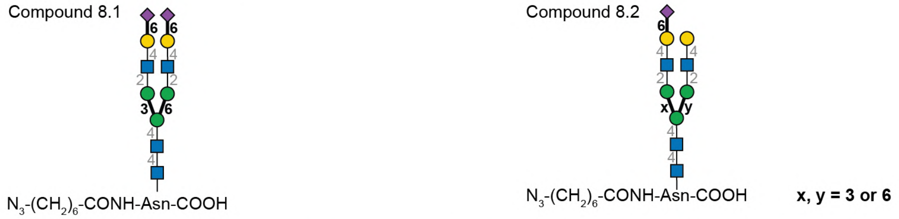

Compound 8.1	*m/z*	RT	Algorithm	Mass
$C_{96}H_{157}N_{11}O_{65}$	835.9852	5.94	Find by Formula	2503.9295

Compound 8.2	*m/z*	RT	Algorithm	Mass
$C_{85}H_{140}N_{10}O_{57}$	1107.9275	5.98	Find by Formula	2212.8358

Compound Chromatograms

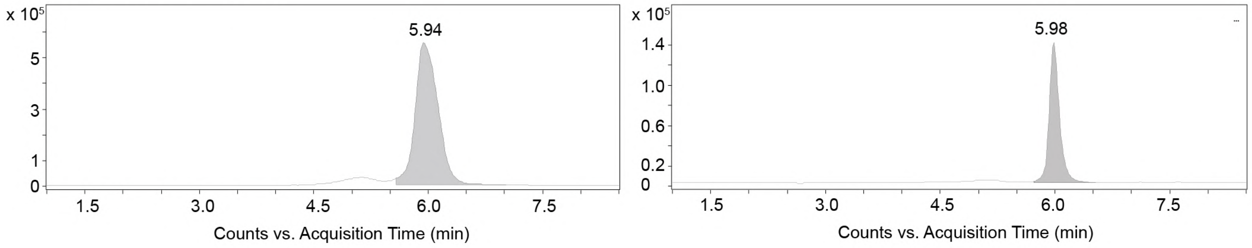

Supplementary Fig. 7: LC-MS results after B4GalT1-catalyzed reaction of mixtures **7**, giving **8**. **a,** Scheme of B4GalT1-catalyzed reaction on mixtures **7** to generate desired structures of **8**. Enzymatic reaction in solution proved the possibility of installing galactose using heterogeneous mixtures **7** as starting material to afford mixtures **8** (Compounds 8.1+8.2). **b,** LC-MS analysis confirmed the completion of B4GalT1-catalyzed reaction to afford mixtures **8**.

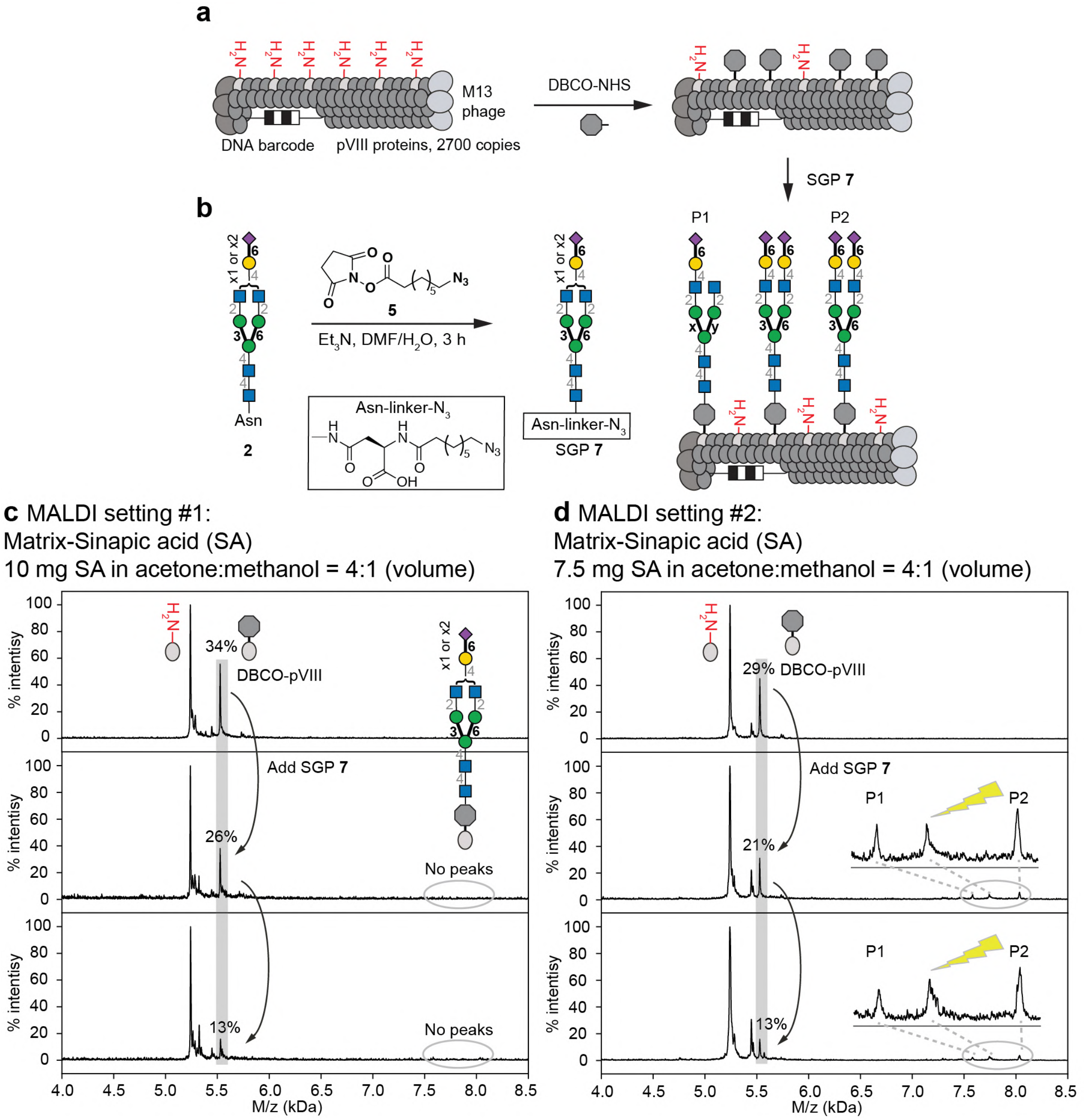

Supplementary Figure. 8: Optimization of MALDI-TOF of sialylated biantennary *N*-linked oligosaccharides **7** on phage.

a, Two steps chemical glycosylation of **7** on phage. **b,** Preparation of heterogeneous azido-*N*-glycan **7**. **c,** MALDI-TOF MS spectrometry characterized the decreasing intensity of alkyne-functionalized product (DBCO-pVIII) after incorporation of azido-modified biantennary *N*-linked oligosaccharides **7**, which indicated the chemical glycosylation step did work; however glycosylated pVIII has not been observed under previously reported MALDI conditions[3]. The problem was solved by changing the MALDI matrix; minor attenuation in the amount of the sinapic acid made it possible to observe the glycosylated pVIII (**d**).

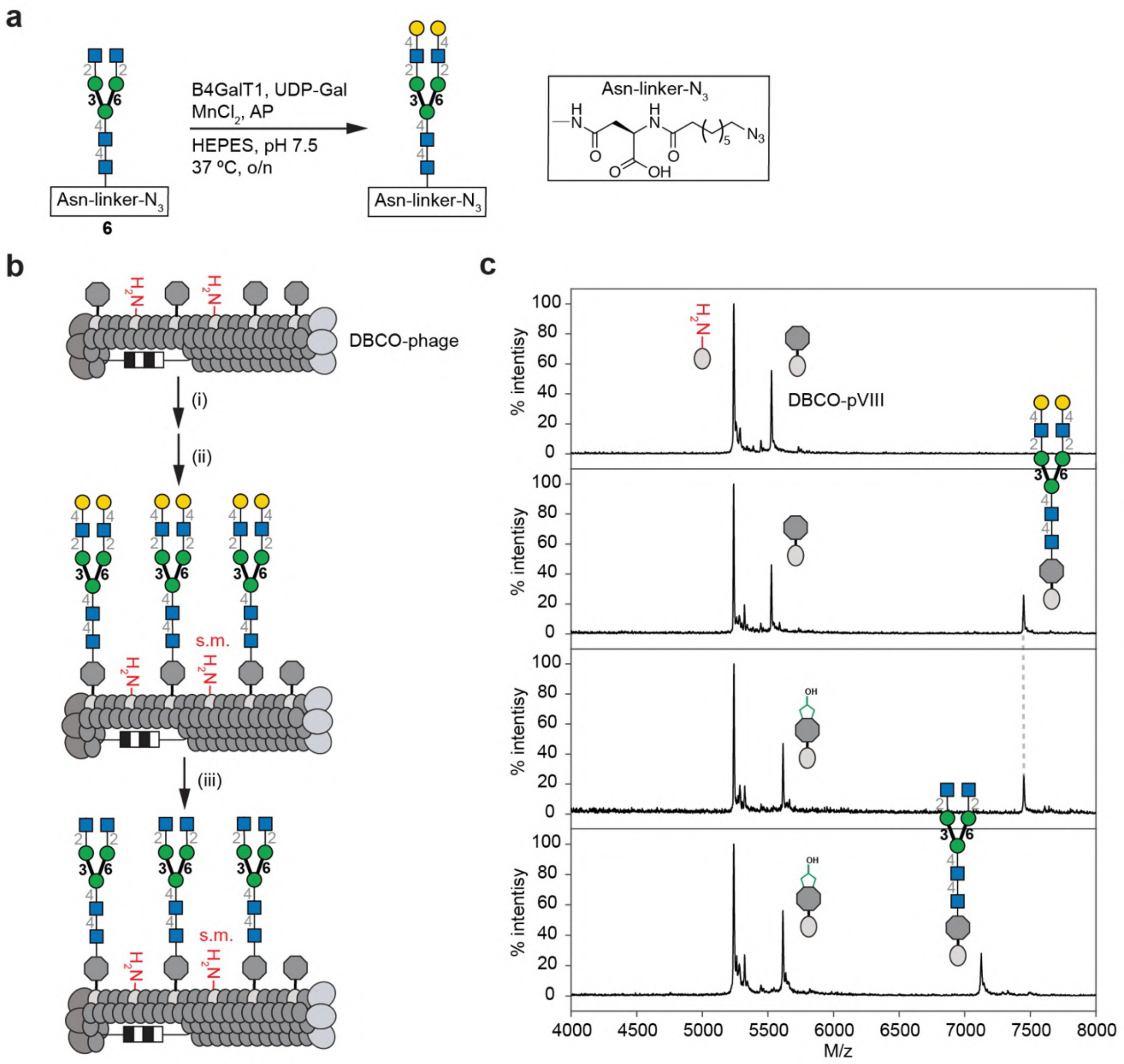

Supplementary Figure. 9: On phage trimming of "homogeneous" N-linked terminal galactose structure.

a, Compound **10** was used as starting material to prepare "homogeneous" *N*-linked terminal galactose structure. **b,** Scheme of galactosidase treatment of homogeneous *N*-linked galactosylated derivative to afford terminal *N*-acetylglucosamine moieties. **c,** MALDI-TOF MS characterized the azidoethanol capping step after chemical glycosylation to block the unreacted DBCO-pVIII intermediate, and the peak of the desired product was generated after the cleavage of galactose.

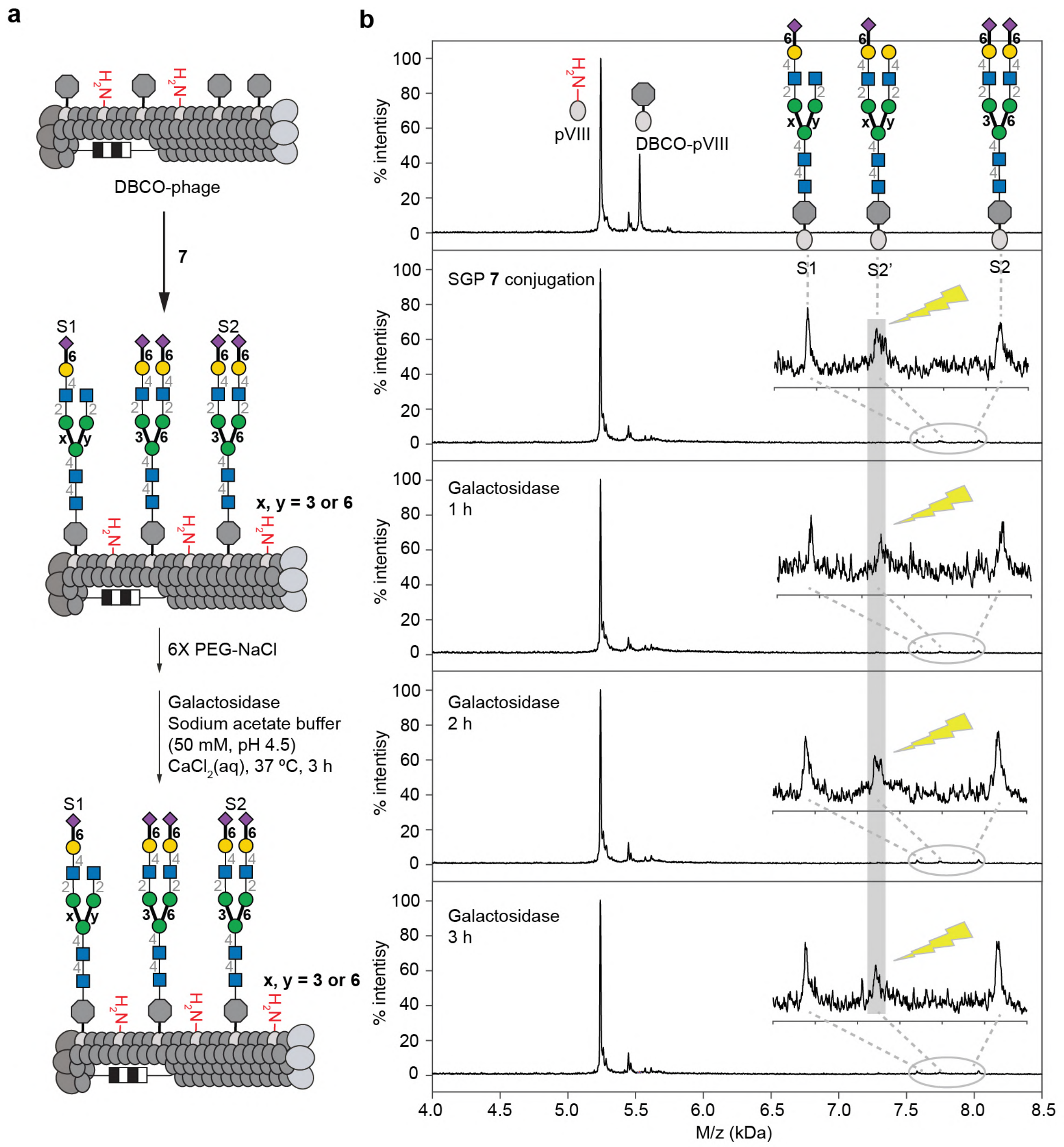

Supplementary Figure. 10: Direct *β*-galactosidase treatment of SGP **7** to confirm the corresponding peak S2' are indeed "ghost" species.

a, Scheme for SGP **7** conjugation followed by β-galactosidase digestion for 3 h. **b,** Time course results indicated the S2' peak is not responding to β-galactosidase treatment indicating that the species with terminal galactose are not present and they were generated due sialic acid cleavage during MALDI-TOF MS analysis.

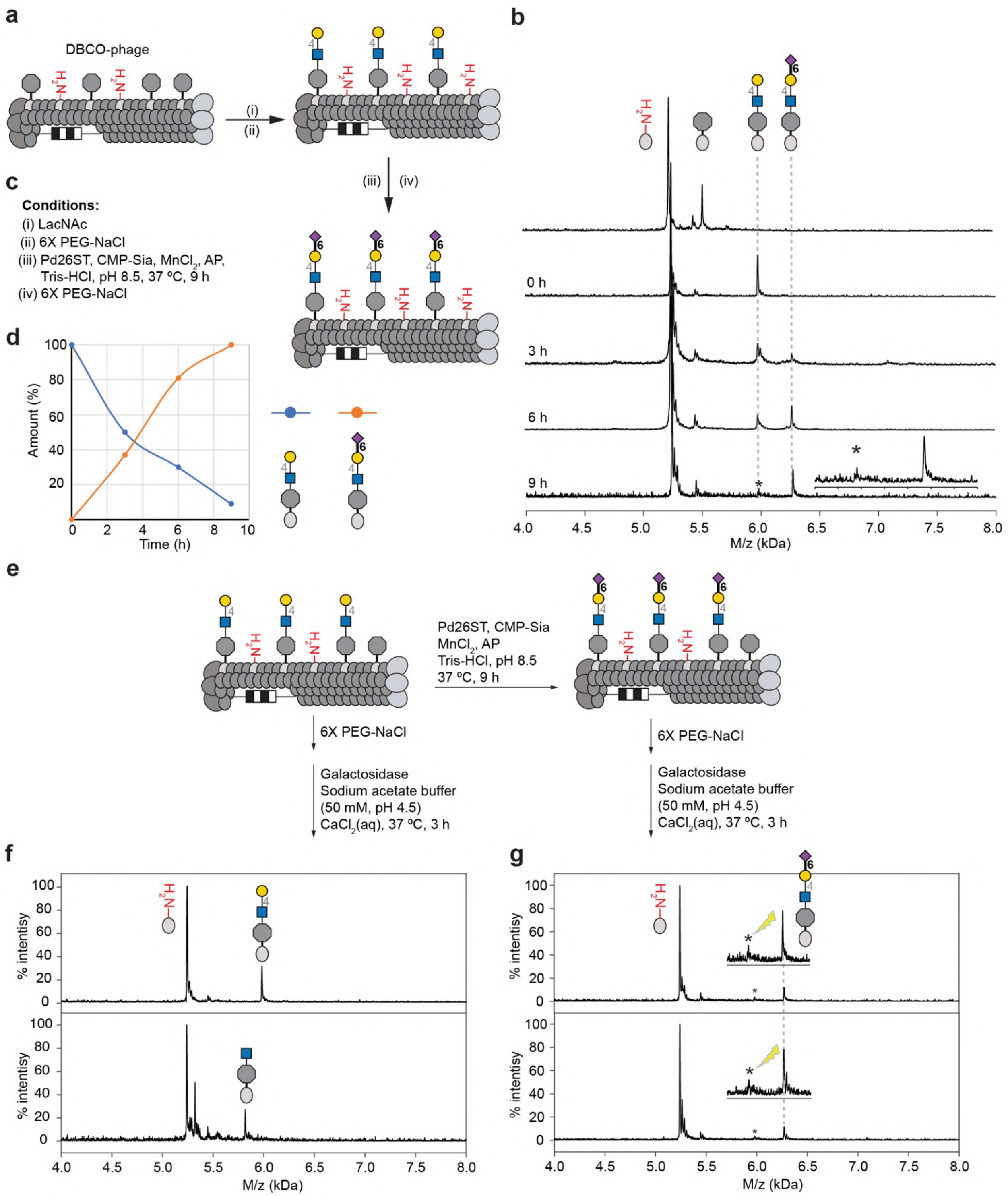

Supplementary Figure. 11: Model study of Pd26ST-catalyzed sialylation on LacNAc-modified pVIII.

a, Scheme for α-(2 →6)-linked sialylation, with reaction pushed to completion by a 9 h incubation. **b,** Monitoring progress by MALDI-TOF MS indicated detailed time course changes. **c,** Conditions

for Pd26ST-catalyzed sialylation. **d,** Plot of time course results with the amount (%) of LacNAc and α-(2 →6)-sialylated LacNAc over time (hours). **e,** Scheme for galactosidase digestion to confirm complete Pd26ST-catalyzed sialylation of LacNAc modified pVIII. (Identical strategy as Supplementary Fig. 14) **f,** MALDI-TOF MS of the positive control. **g,** With the positive control in parallel, MALDI-TOF MS validated the completion of Pd26ST-catalyzed sialylation.

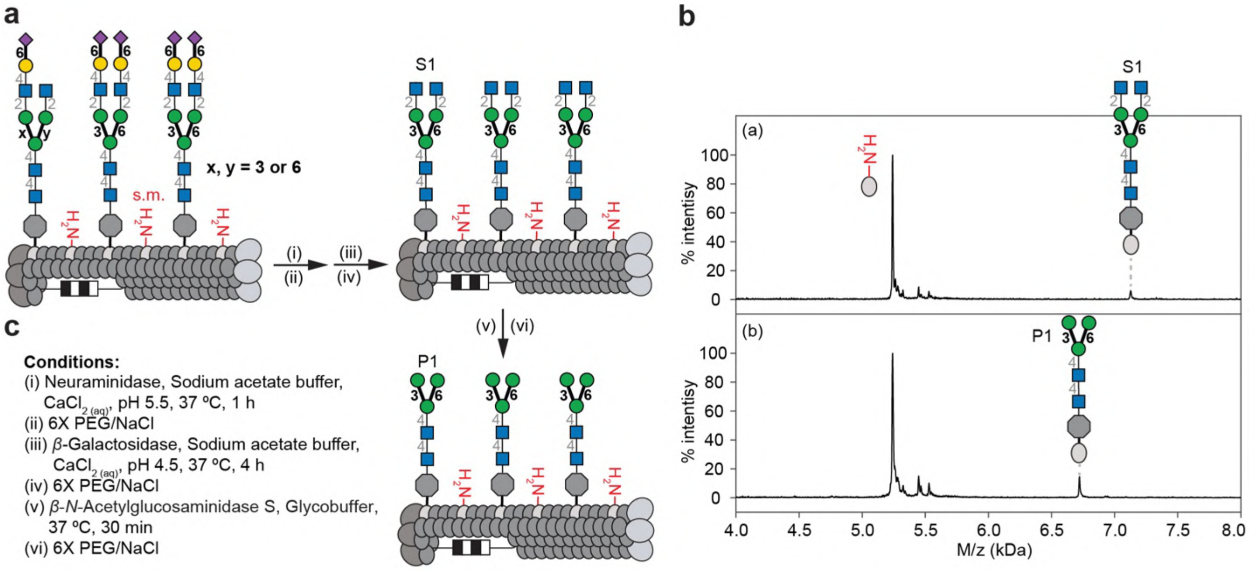

Supplementary Figure. 12: On-phage enzymatic trimming of biantennary glycosyl-asparagines to generate terminal mannose *N*-glycans ($Man_3GlcNAc_2$).
a, Glycosidase treatment as described in Fig. 3 to afford conjugate P1 with terminal mannose on pVIII. **b,** MALDI-TOF MS characterization: (a) Substrate S1 was obtained as described in Fig. 3. (b) *β*-N-Acetylglucosaminidase S treatment of S1 provided P1 on pVIII. **c,** Conditions of the three glycosidase treatments.

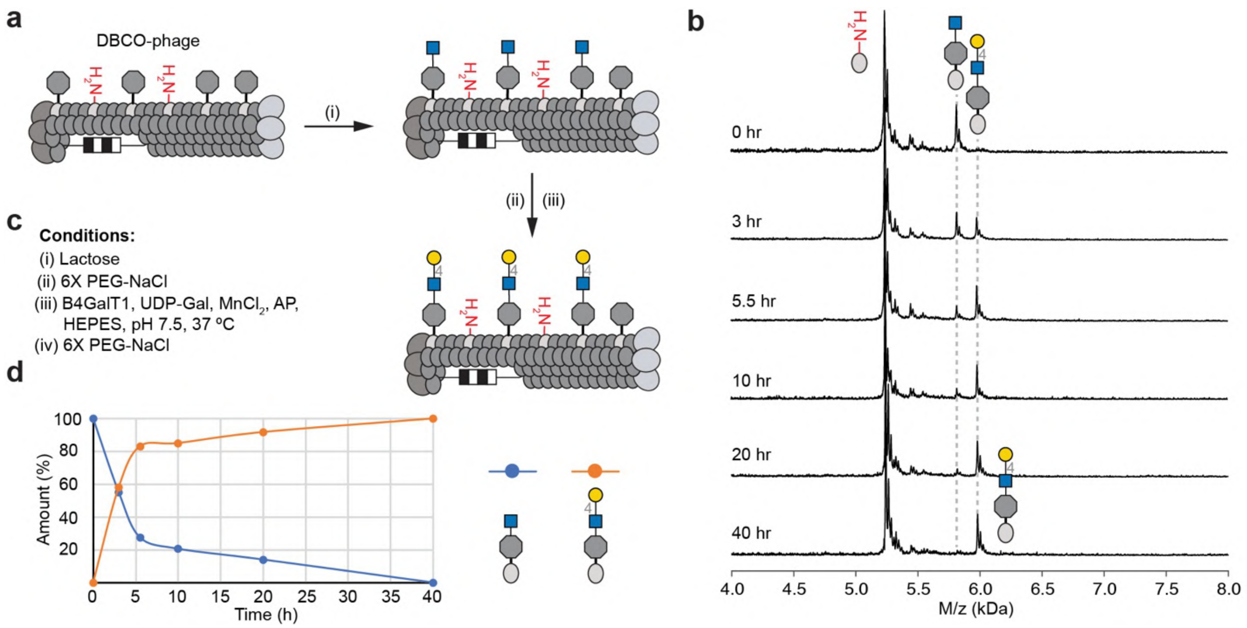

Supplementary Figure. 13: Model study of B4GalT1-catalyzed galactosylation of GlcNAc to generate LacNAc-modified pVIII.

a, Addition of *N*-acetylglucosamine (GlcNAc) to phage by SPAAC and then β-(1→4)-galactosyltransferase (B4GalT1)-catalyzed transfer of galactose (Gal) from UDP-Gal. **b,** Progress of the β-(1→4)-galactosylation as followed by MALDI-TOF MS to indicate time course changes. **c,** Conditions of B4GalT1-catalyzed galactosylation. **d,** Plot of the amount (%) of GlcNAc substrate and LacNAc product over time (h).

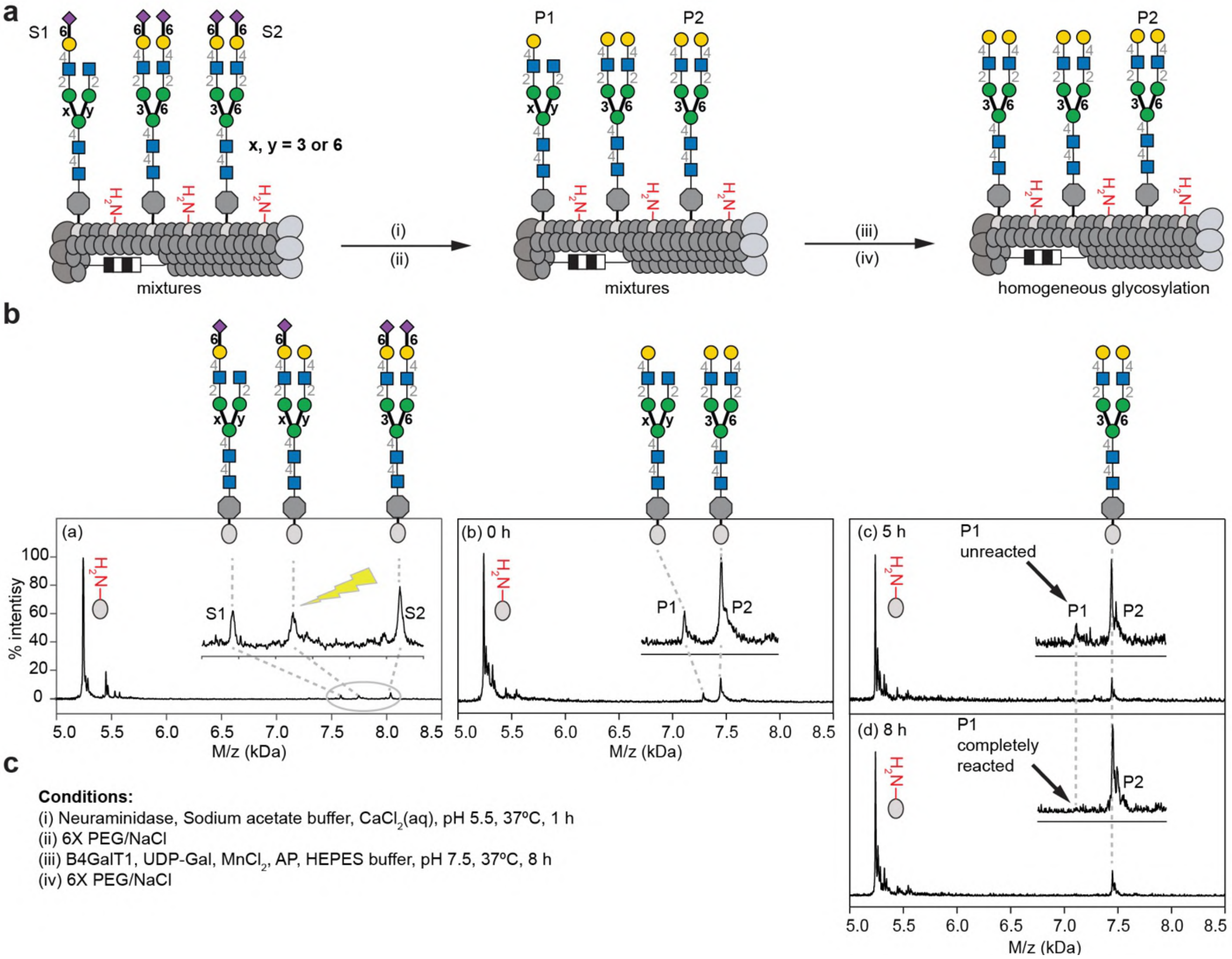

Supplementary Figure. 14: On-phage B4GalT1-catalyzed galactosylation of biantennary N-glycans.

a, Neuraminidase trimming followed by B4GalT1-catalyzed galactosylation to afford galactose terminated glycosyl-asparagine structure P2 on pVIII. **b,** MALDI-TOF MS characterization: (a) substrates S1, S2 were trimmed by neuraminidase to yield (b) a mixture of products P1 and P2. (c) B4GalT1-catalyzed galactosylation converted P1 to P2 yielding a homogeneous display of P2 on pVIII of M13 phage. **c,** Conditions of each enzymatic treatment step.

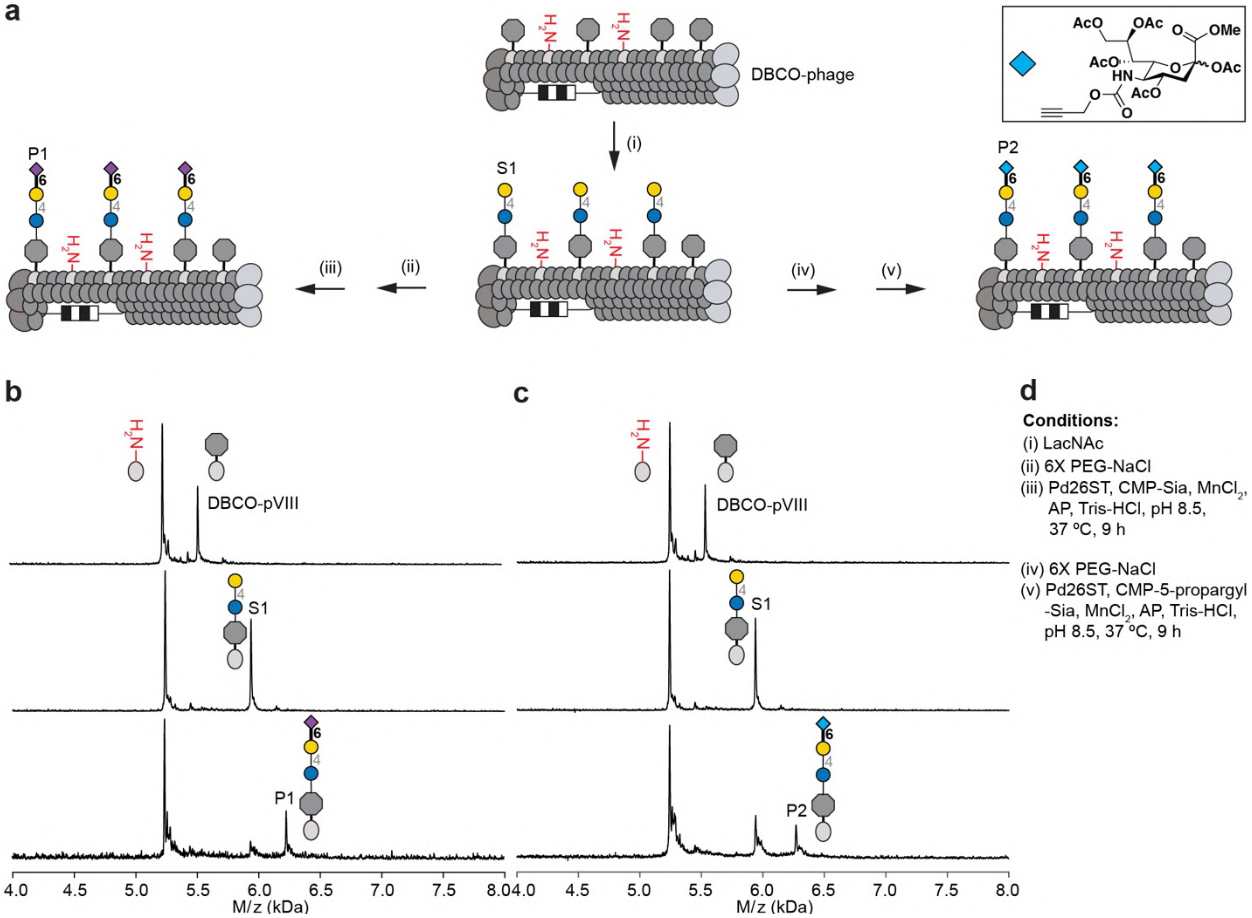

Supplementary Figure. 15: Model study of Pd26ST-catalyzed sialylation of lactose-modified pVIII and use unnatural CMP-sialic acid as a donor.

a, Scheme for α-(2 →6)-linked sialylation and use of unnatural CMP-sialic acid to modify biantennary N-glycans on pVIII. **b,** MALDI-TOF MS indicates that Pd26ST catalyzes the transfer of N-acetylneuramic acid (Neu5Ac) from CMP-Neu5Ac to lactose (S1) to give α-(2 →6)-sialyl-lactose (P1). **c,** MALDI-TOF MS indicates that Pd26ST S1 transfers a C5-modified-sialic acid residue from C5-propargylamido-CMP-sialic acid to S1 to form product P2 on pVIII. **d,** Conditions for α-(2 →6)-sialylation.

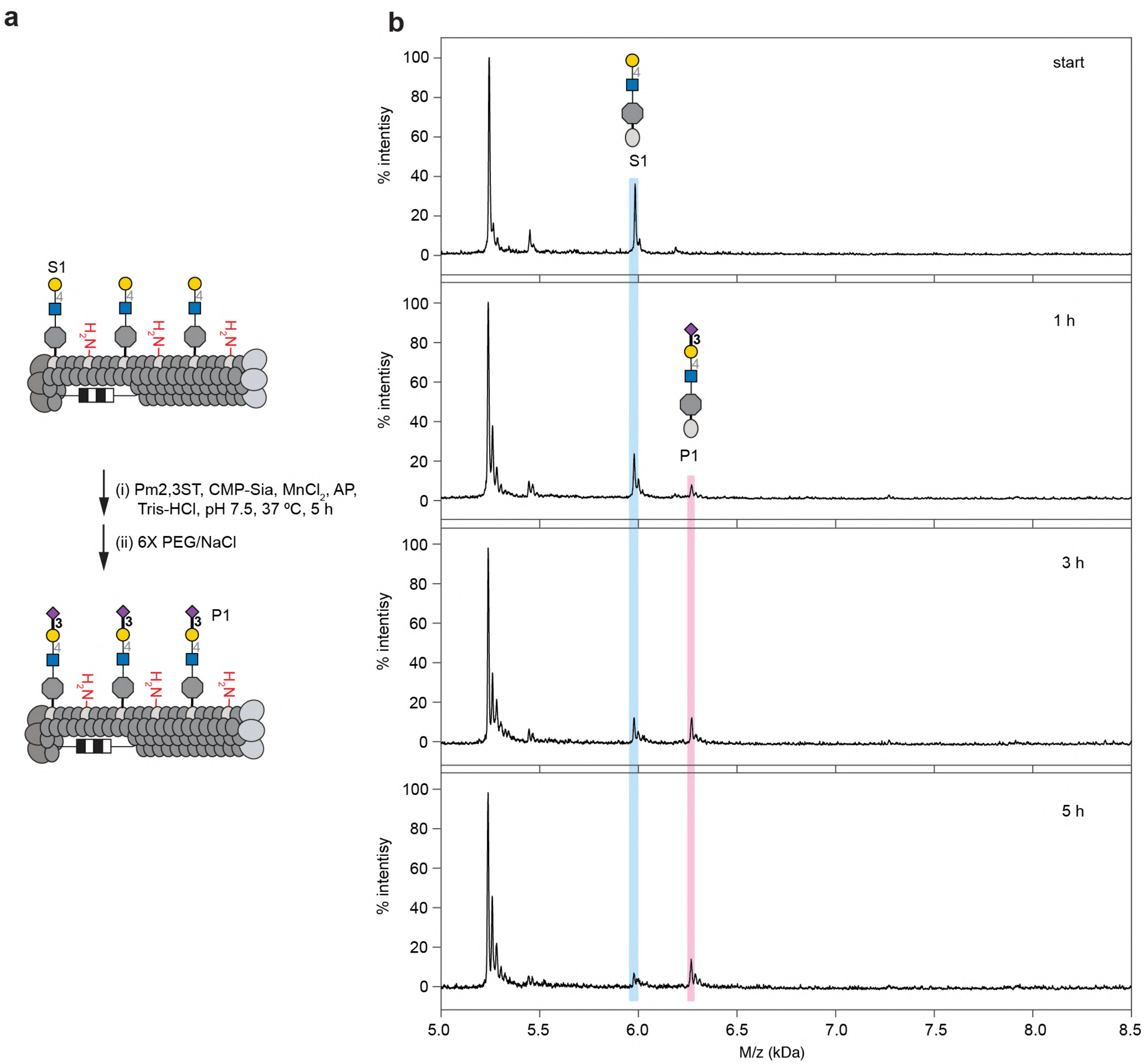

Supplementary Figure. 16: Model study of Pm2,3ST-catalyzed sialylation on LacNAc-modified pVIII.
a, Scheme of α-(2 →3)-linked sialylation. **b,** Monitoring progress by MALDI-TOF MS. Reaction approached completion by 5 h of incubation.

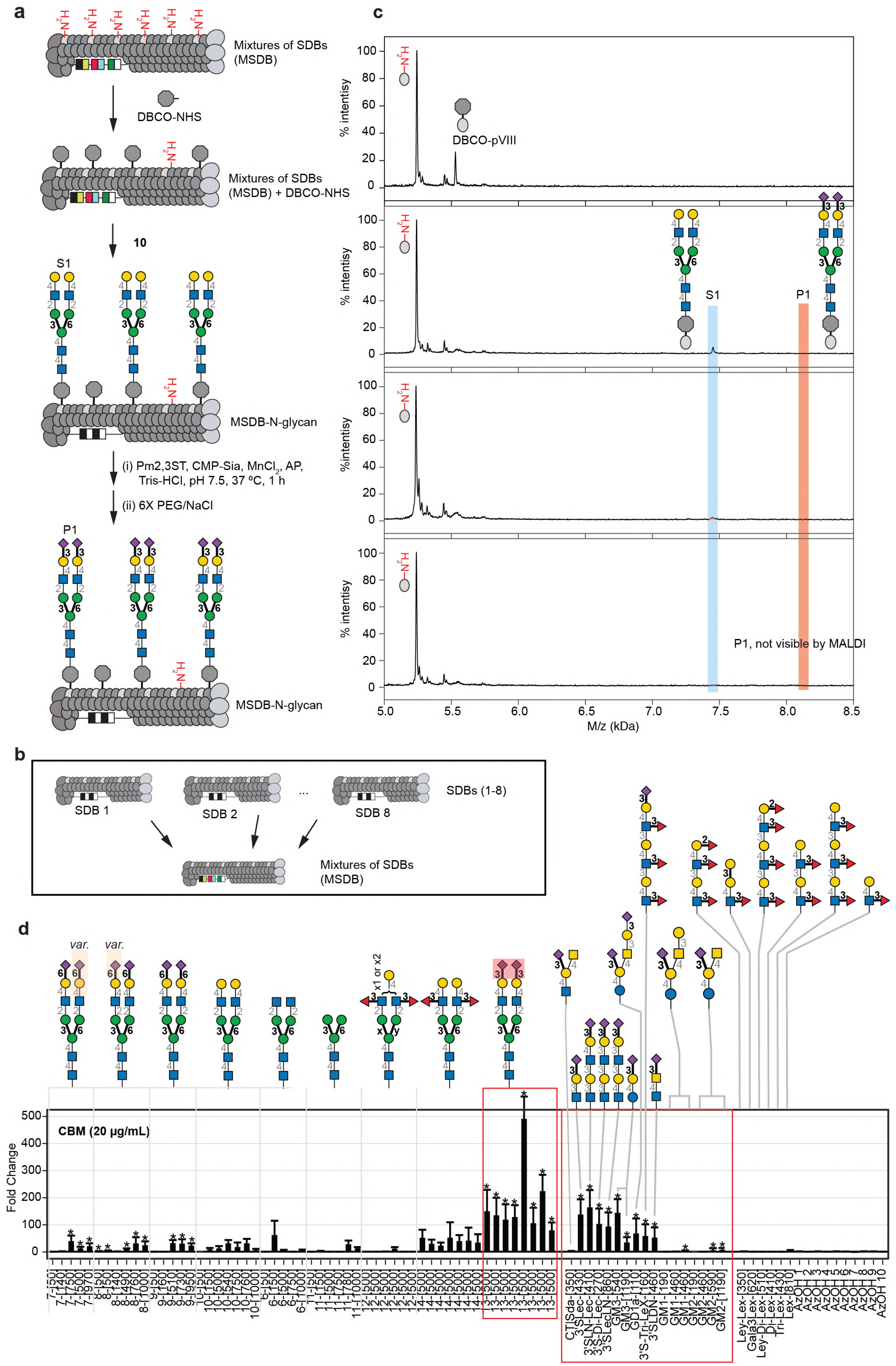
a
Mixtures of SDBs (MSDB)
DBCO-NHS
Mixtures of SDBs (MSDB) + DBCO-NHS
10
S1
MSDB-N-glycan
(i) Pm2,3ST, CMP-Sia, MnCl2, AP, Tris-HCl, pH 7.5, 37 ºC, 1 h
(ii) 6X PEG/NaCl
P1
MSDB-N-glycan
b
SDB 1
SDB 2
SDB 8
SDBs (1-8)
Mixtures of SDBs (MSDB)
c
% intentisy
DBCO-pVIII
S1
P1
P1, not visible by MALDI
M/z (kDa)
d
var.
x1 or x2
CBM (20 μg/mL)
Fold Change

Supplementary Figure 17. On-phage enzymatic extension of Gal-terminated N-glycan by Pm2,3ST and structure validation by binding to diCBM40 lectin.

a, Scheme of α-(2 →3)-linked sialylation. Prior to reaction, 8 phage clones with defined SDBs were combined to create a mixture with multiple SDB (MSDB). This MSDB was used to perform the modification by DBCO-NHS, ligation of biantennary N-glycan, and then its enzymatic modification. **b,** Scheme of mixing 8 phage clones to create multiple SDB (MSDB). **c,** MALDI-TOF MS indicated substrate S1 was consumed after 1 h incubation. The negative charge of terminal α-(2 →3)-linked sialic acid affected the product P1 detection by MALDI-TOF MS. The desired product peak P1 was not visible in MALDI-TOF MS. **d,** To validate the formation of terminal α-(2 →3)-linked sialic acid *N*-glycan on MSDB, we mixed MSDB glycoconjugates with LiGA6×5, fucosylated N-glycans, α-(2 →3)-linked sialic acid O-glycans and fucosylated O-glycans. The mixture was tested for binding to diCBM40. The results confirmed the binding of glycans with terminal α-(2 →3)-linked sialic acid to diCBM40. Binding to diCBM40 was calculated as FC of each glycophage clone in diCBM40 coated wells when compared to BSA coated wells. * denotes significantly enriched reads (FDR ≤ 0.05, n = 5 independent binding experiments for diCBM40 and BSA). Error bars represent s.d. propagated from the variance of the normalized sequencing data.

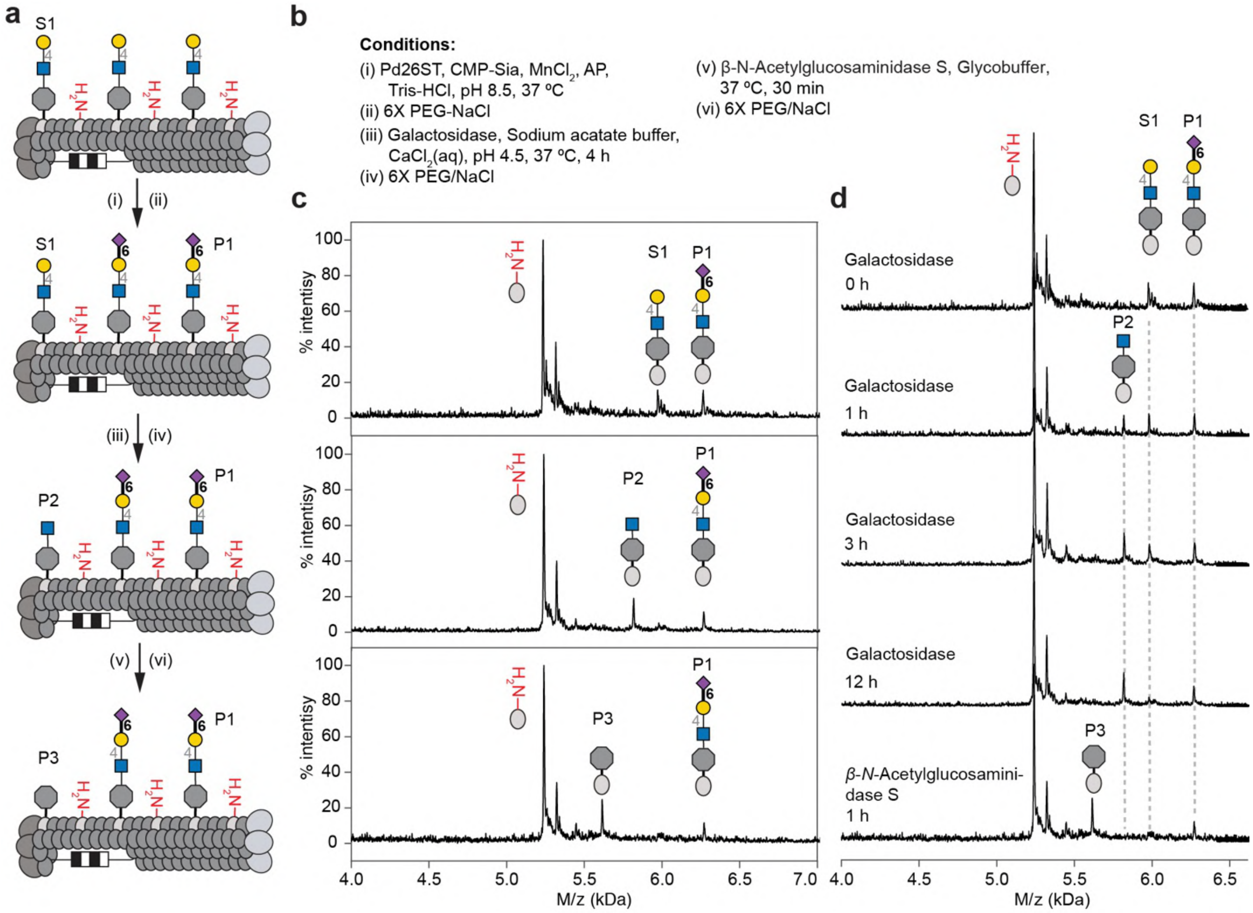

Supplementary Figure. 18: Galactosidase digestion of incomplete Pd26ST-catalyzed sialylation on LacNAc-modified pVIII.

a, Scheme for α-(2→6)-linked sialylation, galactosidase treatment and then β-N-acetylglucosaminidase S treatment of LacNAc-modified pVIII. **b,** Conditions for glycosidase digestion. **c,** MALDI-TOF MS of each enzyme treatment step indicate that S1 was converted to P1 by Pd26ST; the reaction was left incomplete on purpose by incubation for only 1 h. Unreacted S1 can be digested to P2 and then P3 by glycosidase treatment, but the sialylated product P1 was not digested. **d,** Progress of glycosidase treatment by MALDI-TOF MS indicated detailed time course changes.

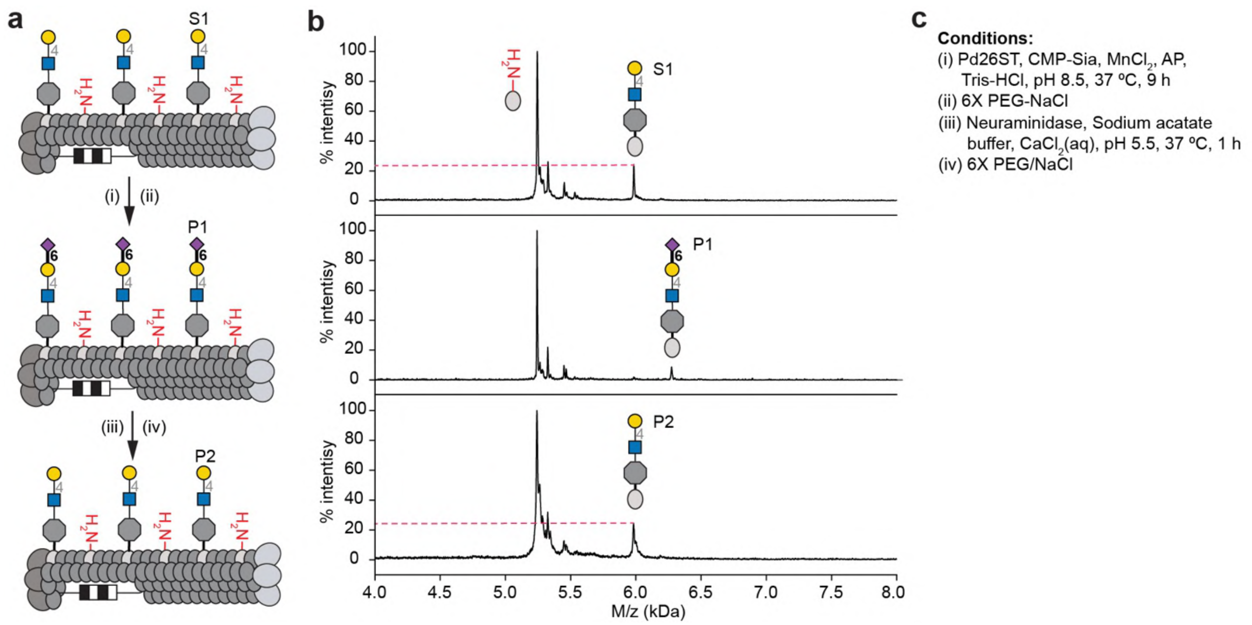

Supplementary Figure. 19: Comparison of the intensity before enzymatic glycan extension and after enzymatic glycan cleavage on pVIII.

a, Scheme for the intensity comparison study. Neuraminidase was used to cleave sialic acid after Pd26ST-catalyzed sialylation on pVIII. **b,** P1 was generated, followed by neuraminidase cleavage to form P2 (Same structure as S1). e MALDI-TOF MS indicated the corresponding intensity of LacNAc-modified peak S1 and P2 were the same. **c,** Conditions for each step.

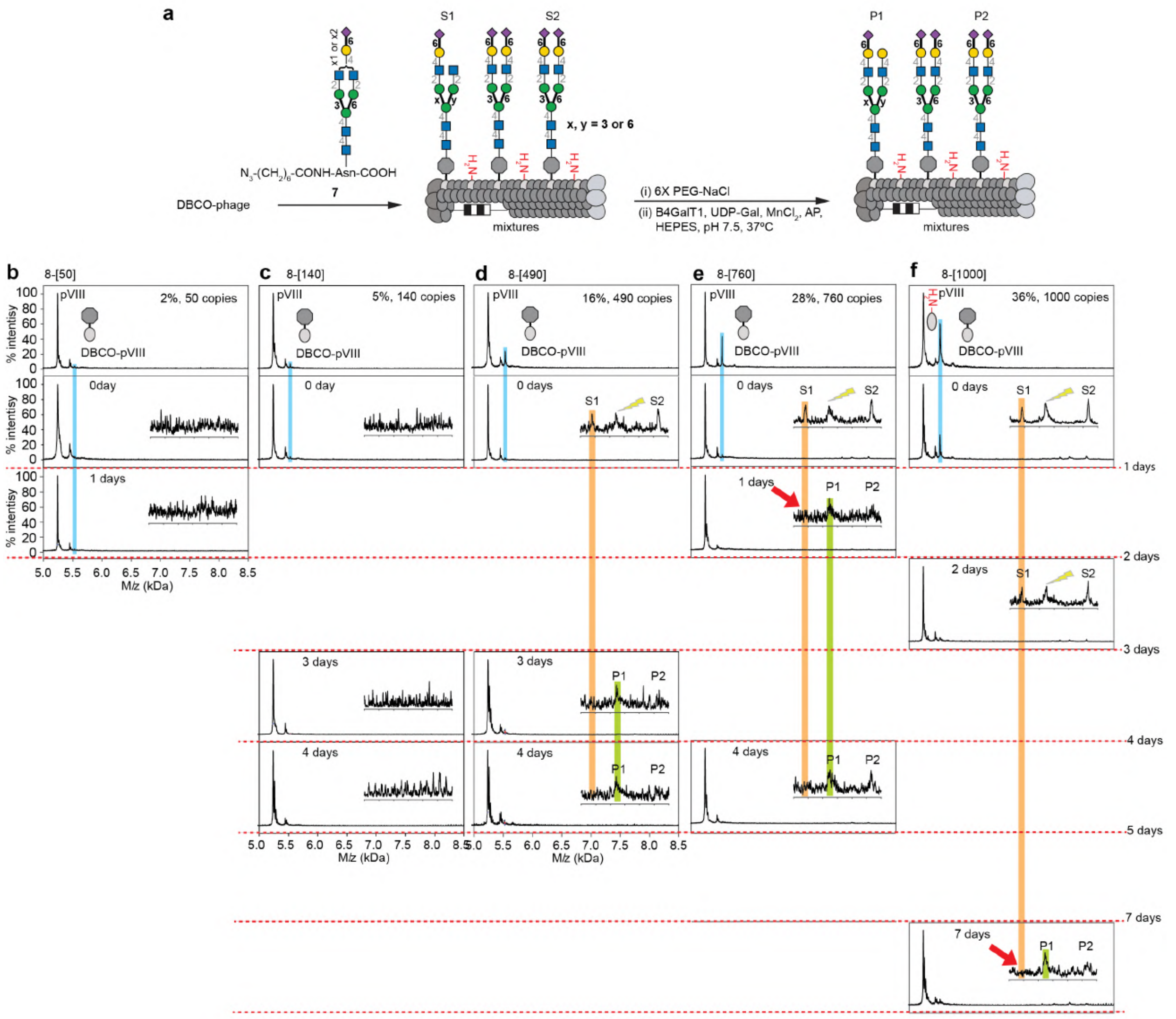

Supplementary Figure. 20: Enzymatic extension of SGP glycan (by B4GalT1) displayed on phage at 5 different densities monitored by MALDI TOF MS.

a, Phage was modified by 2-7 uL of 50 mM DBCO for 4 min-65 min at room temperature to achieve the indicated copy number of DBCO molecules on phage surface (see Supplementary Fig. 25 for details). **b,** After modification of DBCO phage by azido-SGP, B4GalT1 was used to catalyze the transfer of galactose (Gal) from UDP-Gal. Reaction was run for 1-7 days and higher DBCO density required longer completion times as indicated in (**c-f**). In **e-f,** At 750 copies per phage, B4GalT1 converts S1 to P1 in 1 day. The starting material peak S1 was consumed by 1 day and any minor residual amount of S1 observed at day 1 did not change after a prolonged reaction (4-5 days). In contrast, significant S1 peak was observed up to 7 days of B4GalT1 modification at 1000 copies per phage.

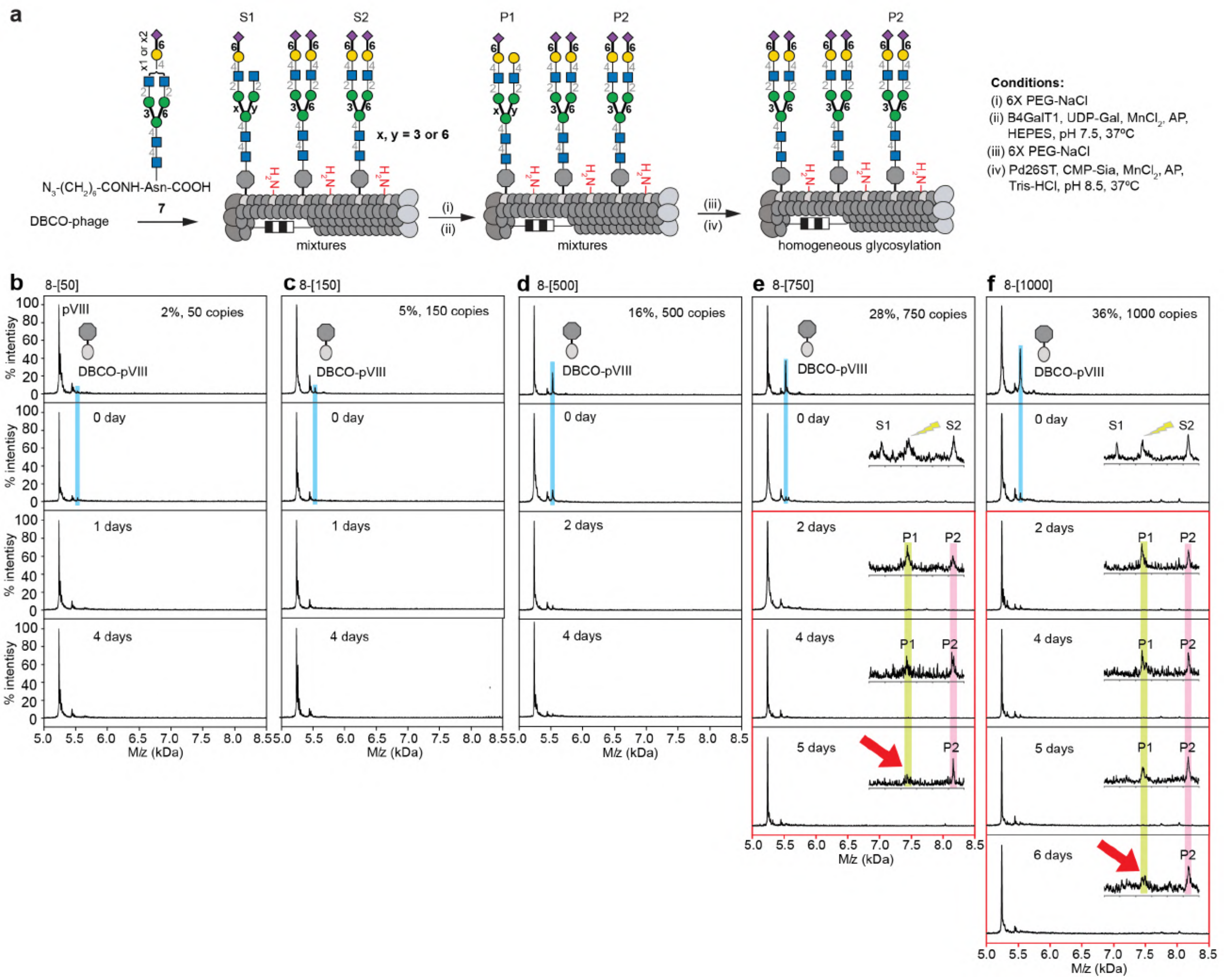

Supplementary Figure. 21: Two steps enzymatic extension (by B4GalT1 and Pd26ST) of SGP glycan displayed on phage at 5 different densities monitored by MALDI TOF MS.

a, Phage was modified by 2-7 uL of 50 mM DBCO for 4 min-65 min at room temperature to achieve the indicated copy number of DBCO molecules on phage surface (see Supplementary Fig. 25 for details for details). **b,** After modification of DBCO phage by azido-SGP, β-1,4-galactosylation by B4GalT1 and α-2,6-linked sialylation by Pd26ST afforded homogeneous sialylated product P2. Reaction was run for 1-6 days; higher DBCO density required longer completion times as indicated in (**c-f**). In **e-f,** At 750 copies per phage, Pd26ST converts P1 to P2 in 3 days. In contrast, P1 peak was observed up to 4 days at 1000 copies per phage.

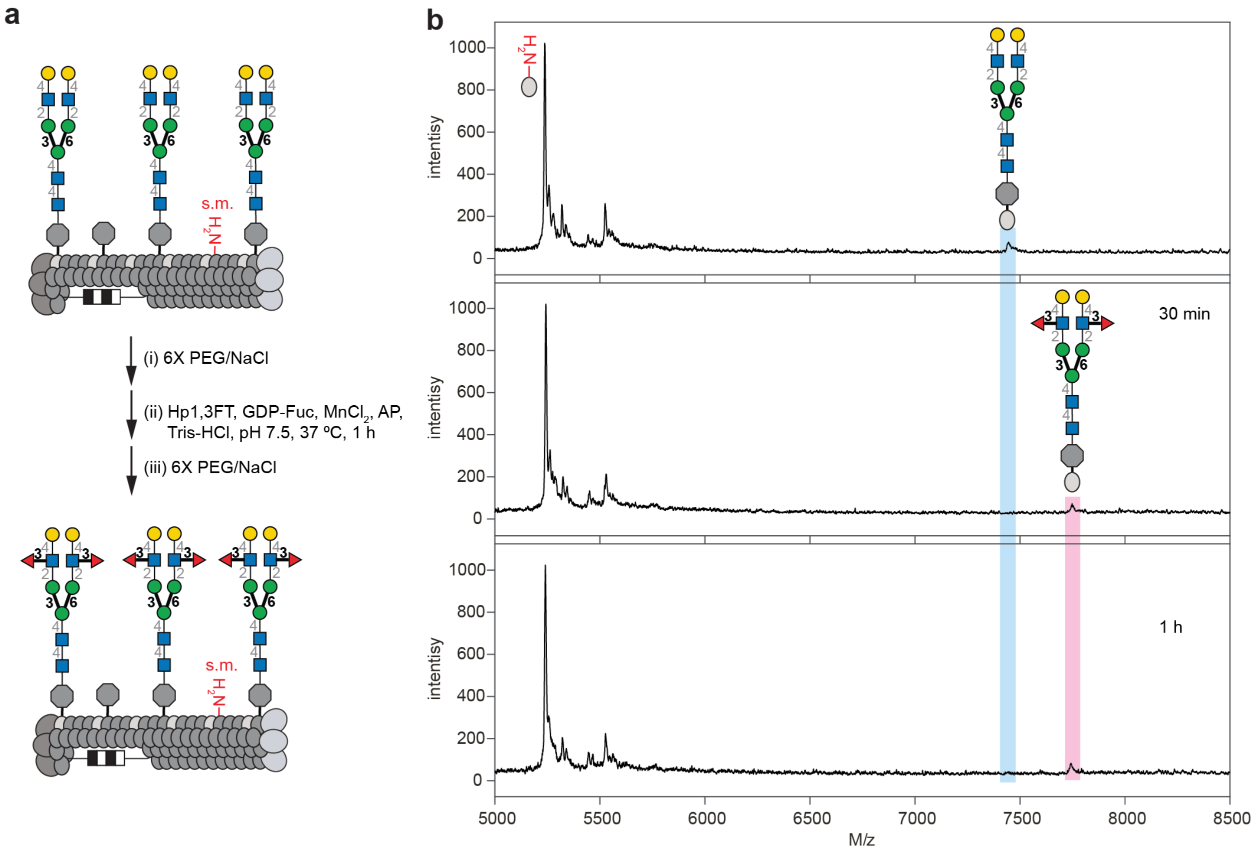

Supplementary Figure. 22: On-phage of Hp1,3FT-catalyzed α-(1 →3) fucosylation of Gal-terminated N-glycan.

a, Scheme of α-(1 →3)-linked fucosylation. **b,** Monitoring progress by MALDI-TOF MS indicated that the reaction was complete after 30 min.

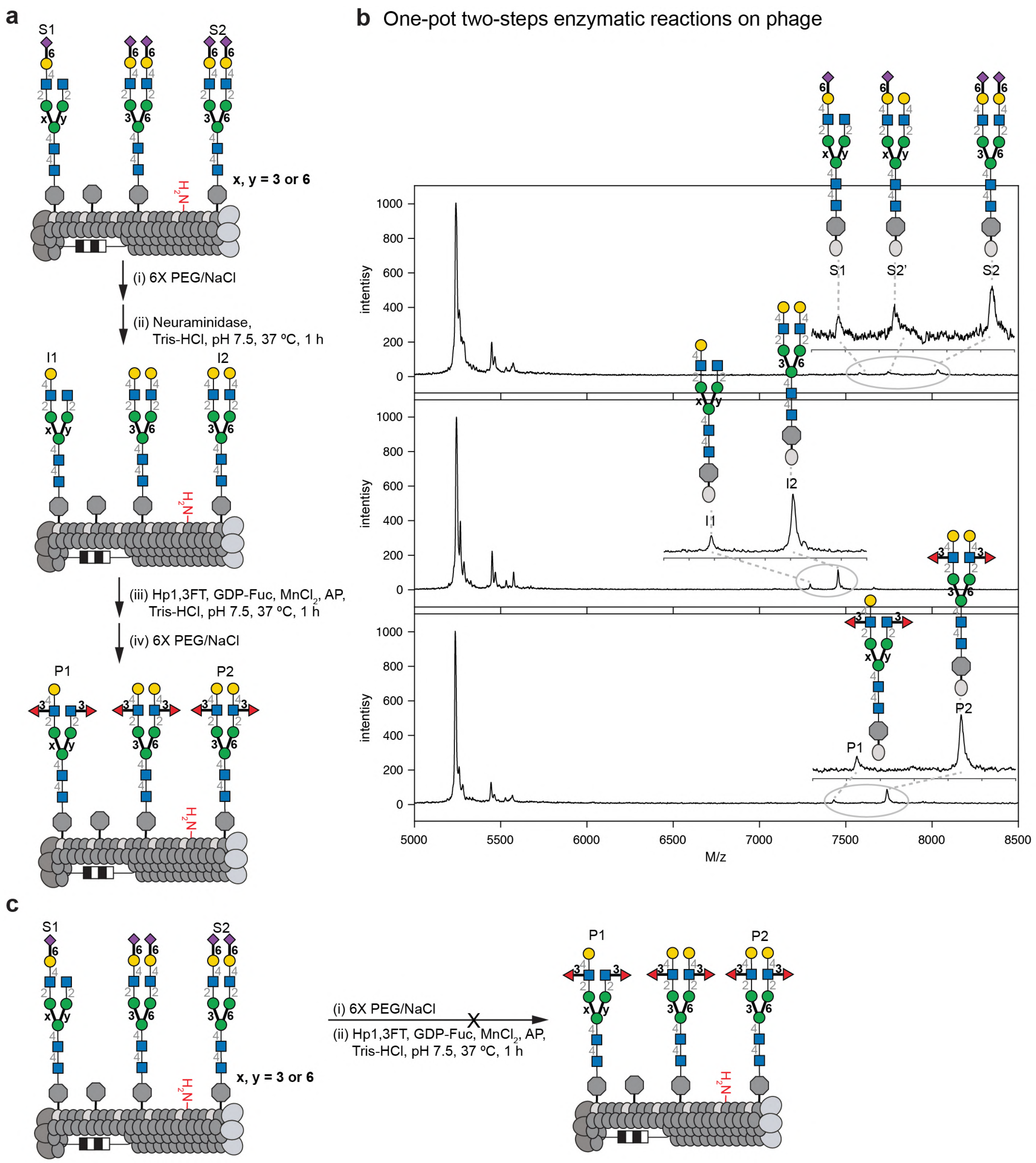

Supplementary Figure. 23: On-phage Hp1,3FT-catalyzed α-(1 →3) fucosylation by one pot two steps enzymatic strategy.

a, Scheme of α-(1 →3)-linked fucosylation. **b,** Cleavage of sialic acid by neuraminidase followed by in situ treatment of Hp1,3FT generated mixtures of P1 and P2 on phage with α-(1 →3)-linked fucose to GlcNAc. The reaction was monitored by MALDI-TOF. **c,** We observed that Hp1,3FT can't tolerate the presence of terminal α-(2 →6)-sialic acid and it does not modify S1 and S2 *N*-glycans on phage.

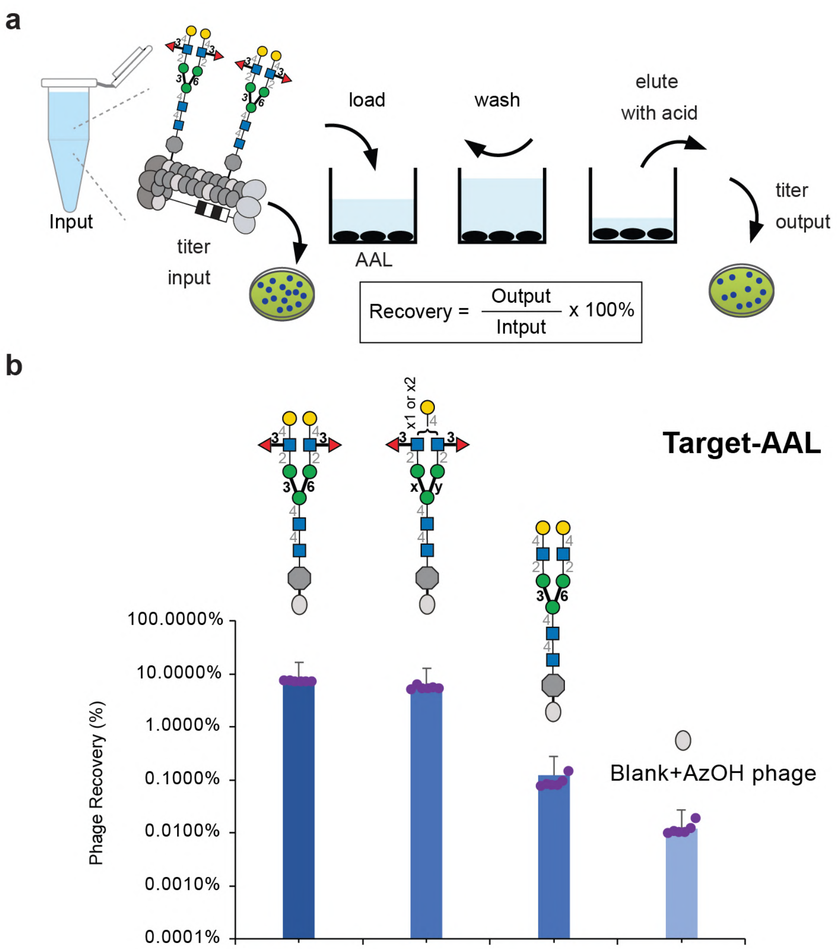

Supplementary Figure. 24: Binding of phage displayed *N*-glycans with α-(1 →3)-linked fucose. **a,** Clonal phage modified with α-(1 →3)-linked fucose (See Supplementary Fig. 22-23) was incubated with well coated by AAL lectin. The wells were washed and treated by 0.1 M HCl to elute the phage. The input and output were tittered in agar and the output/input ratio was used to calculate the recovery. **b,** Fucosylated clones have 50 times higher recovery when compared to parent glycan 1000 times more than blank phage. n = 6 biologically independent experiments binding to AAL. Data is represented as mean+s.d, and the s.d for recovery is propagated from variance of input and output PFU.

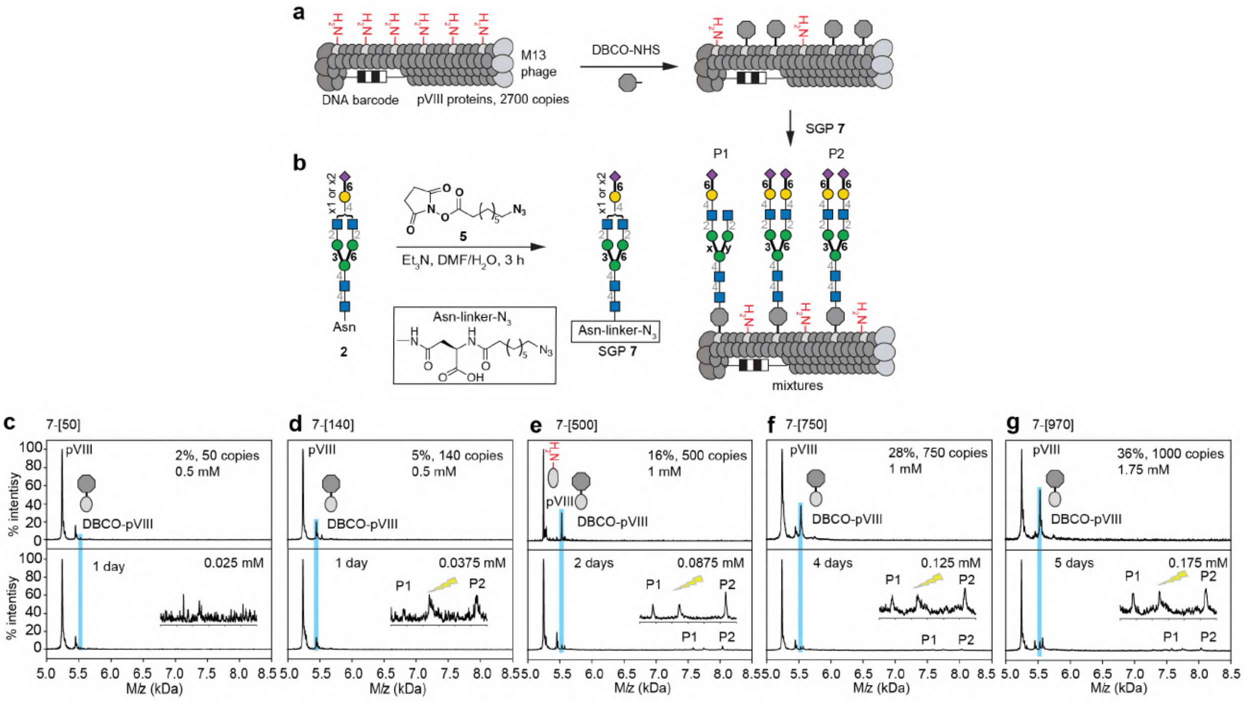

Supplementary Figure. 25: Conjugation of SGP glycan to phage at 5 different densities as monitored by MALDI TOF MS.

a, Phage was modified by 2-7 uL of 50 mM DBCO for 4 min-65 min at room temperature as detailed below to achieve the indicated copy number of DBCO molecules on phage surface. **b,** Modification of DBCO phage by azido-SGP (see Supplementary Fig. 6 for synthesis) resulted in complete (**c-f**) of nearly complete **g,** disappearance of DBCO-pVIII peak in MALDI indicating the completion of the reaction. Reaction was run for 1-5 days and higher DBCO density required longer completion times as indicated in (**c-g**). All conjugates were exposed to azido ethanol to cap any unreacted cyclooctyne residues.

Experimental details:

Combine clonal phage and DBCO in EpiTube according to desired density:
a) 200uL SDB + 2uL DBCO = 2% to 4% density (50 copies p8)
b) 200uL SDB + 2uL DBCO = 4% to 10% density (150 copies p8)
c) 200uL SDB + 4uL DBCO = 16% to 18% density (500 copies p8)
d) 200uL SDB + 4uL DBCO = 26% to 28% density (750 copies p8)
e) 200uL SDB + 7uL DBCO = 36% to 38% density (1000 copies p8)

Incubate at room temperature on shaker for time according to desired density:
a) 4 minutes = 2% to 4% density (50 copies p8)
b) 10 minutes = 4% to 10% density (150 copies p8)
c) 17.5 minutes = 16% to 18% density (500 copies p8)
d) 45 minutes = 26% to 28% density (750 copies p8)
e) 65 minutes = 36% to 38% density (1000 copies p8)

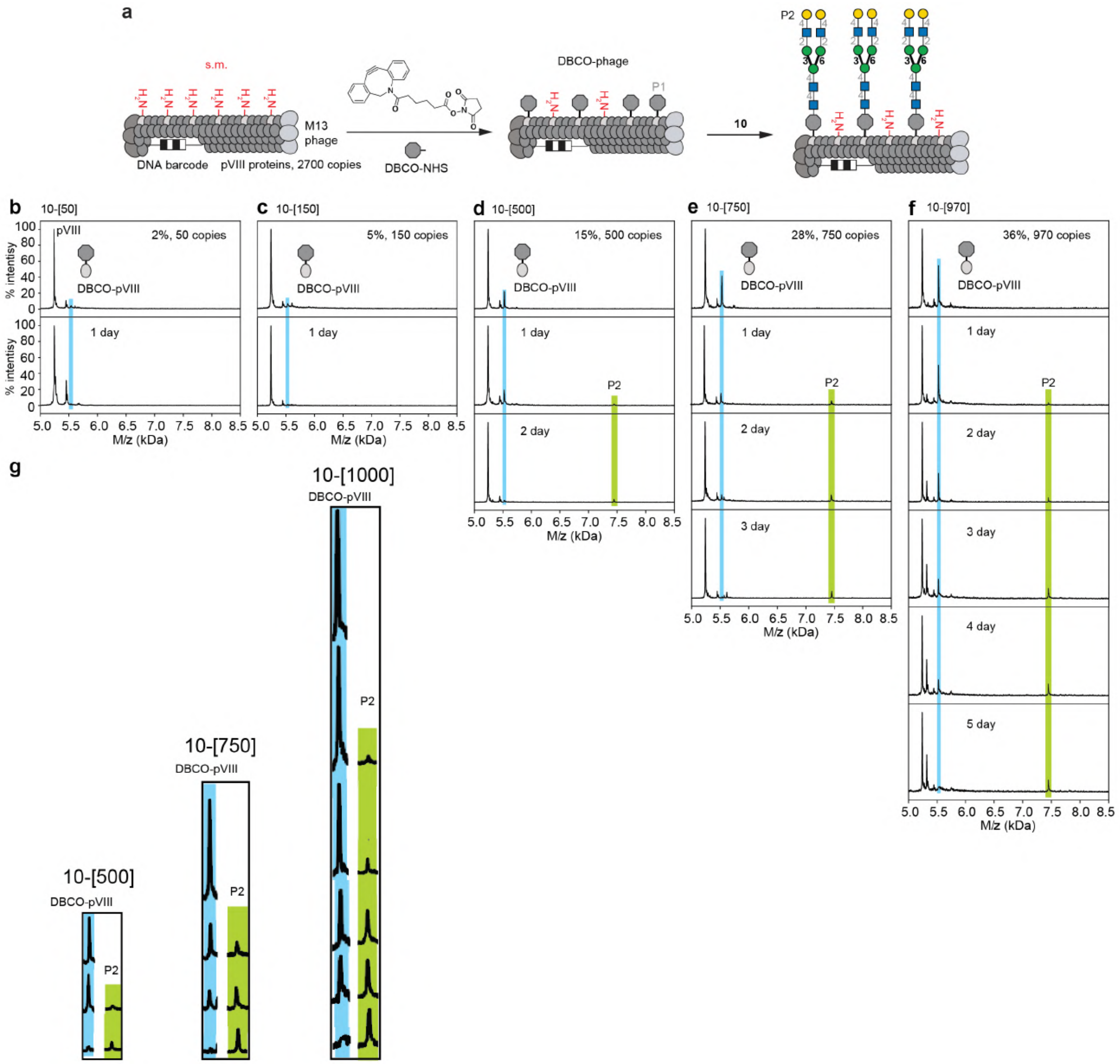

Supplementary Figure. 26: Conjugation of *N*-glycan **10** to phage at 5 different densities as monitored by MALDI TOF MS.

a, Phage was modified by 2-7 uL of 50 mM DBCO for 4 min-65 min at room temperature to achieve the indicated copy number of DBCO molecules on phage surface. **b,** Modification of DBCO phage by *N*-glycan **10** resulted in complete (**b-f**) disappearance of DBCO-pVIII peak in MALDI indicating the completion of the reaction. Reaction was run for 1-5 days; higher DBCO density required longer completion times as indicated in (**b-f**). All conjugates were exposed to azido ethanol to cap any unreacted cyclooctyne residues. **g,** Expanded peaks of DBCO-pVIII and P2 at density of 500, 750 and 1000 copy numbers per phage.

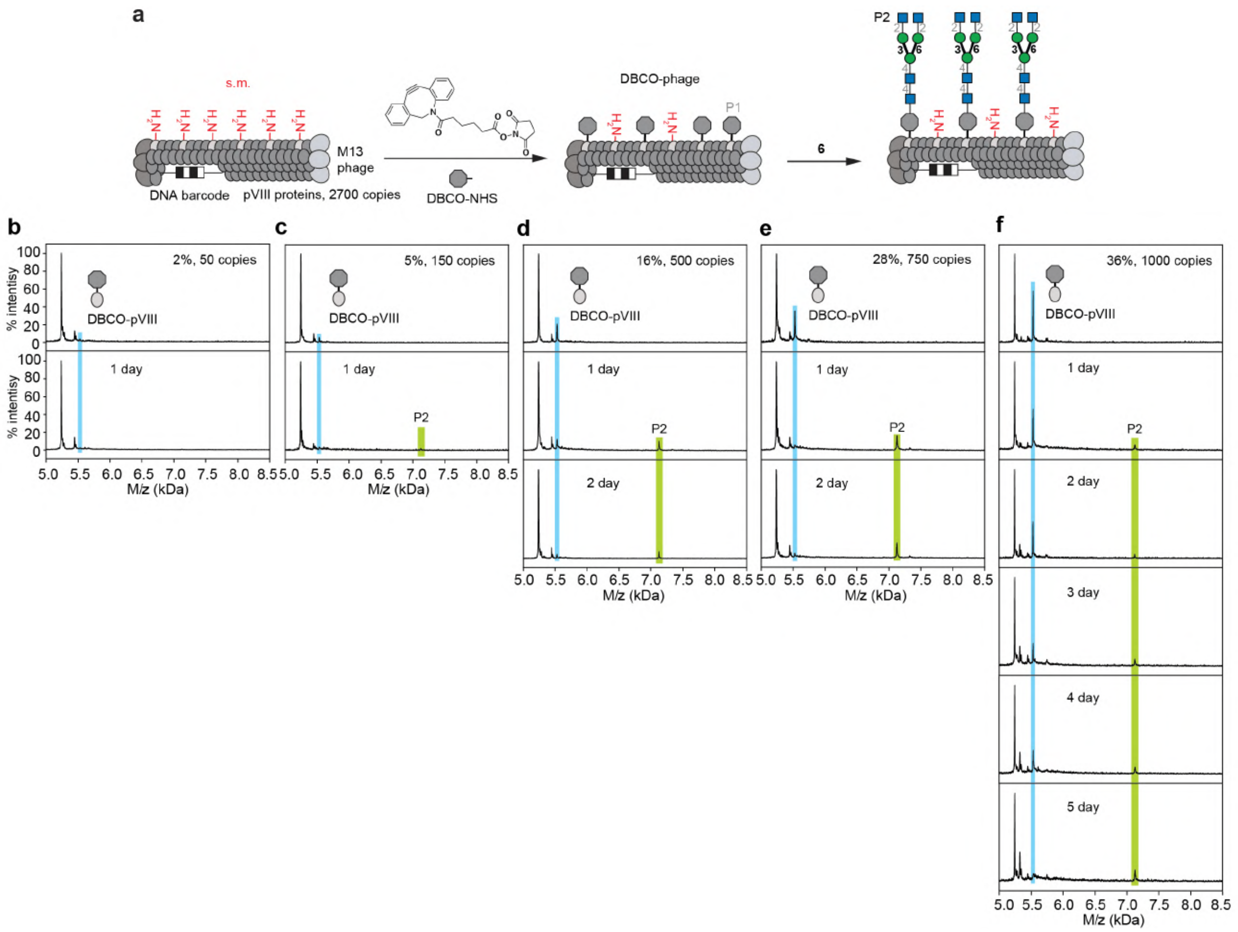

Supplementary Figure. 27: Conjugation of *N*-glycan **6** to phage at 5 different densities as monitored by MALDI TOF MS.

a, Phage was modified by 2-7 uL of 50 mM DBCO for 4 min-65 min at room temperature to achieve the indicated copy number of DBCO molecules on phage surface. **b,** Modification of DBCO phage by N-glycan **6** resulted in complete (**b-f**) disappearance of DBCO-pVIII peak in MALDI indicating the completion of the reaction. Reaction was run for 1-5 days; higher DBCO density required longer completion times as indicated in (**b-f**). All conjugates were exposed to azido ethanol to cap any unreacted cyclooctyne residues.

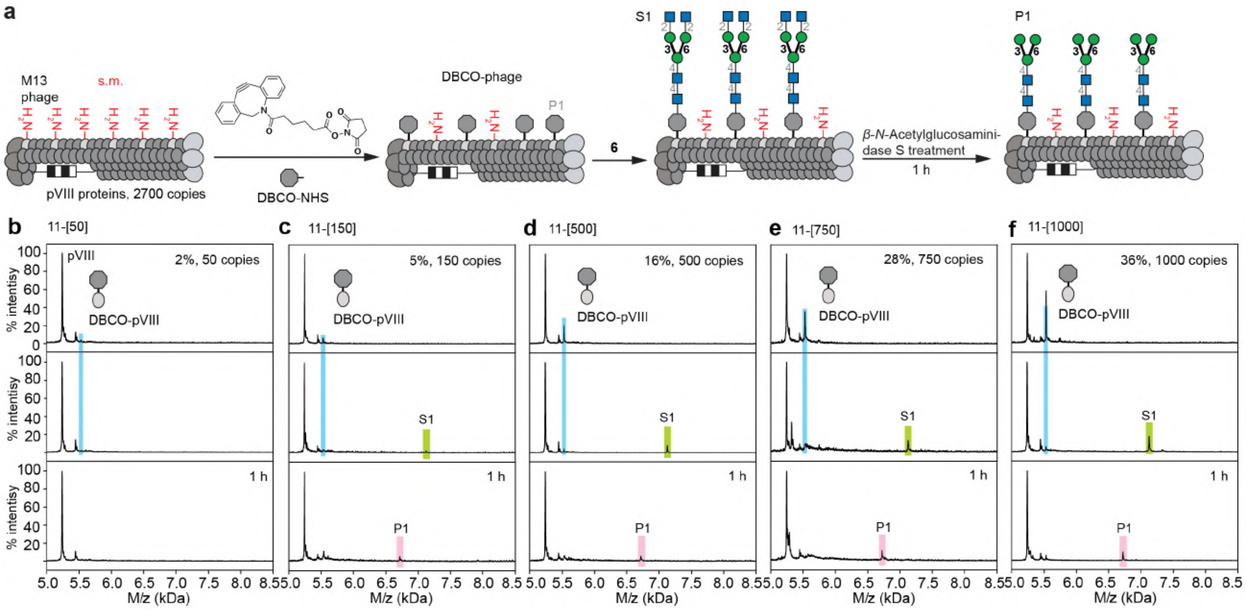

Supplementary Figure. 28: On-phage enzymatic trimming of **6** to generate terminal mannose *N*-glycan ($Man_3GlcNAc_2$) at 5 different densities as monitored by MALDI TOF MS.

a, Phage was modified by 2-7 uL of 50 mM DBCO for 4 min-65 min at room temperature to achieve the indicated copy number of DBCO molecules on phage surface. **b-f,** After modification of DBCO phage by S1 glycan, β-N-Acetylglucosaminidase S treatment of S1 provided desired P1 on pVIII. Each reaction was complete in 1 h.

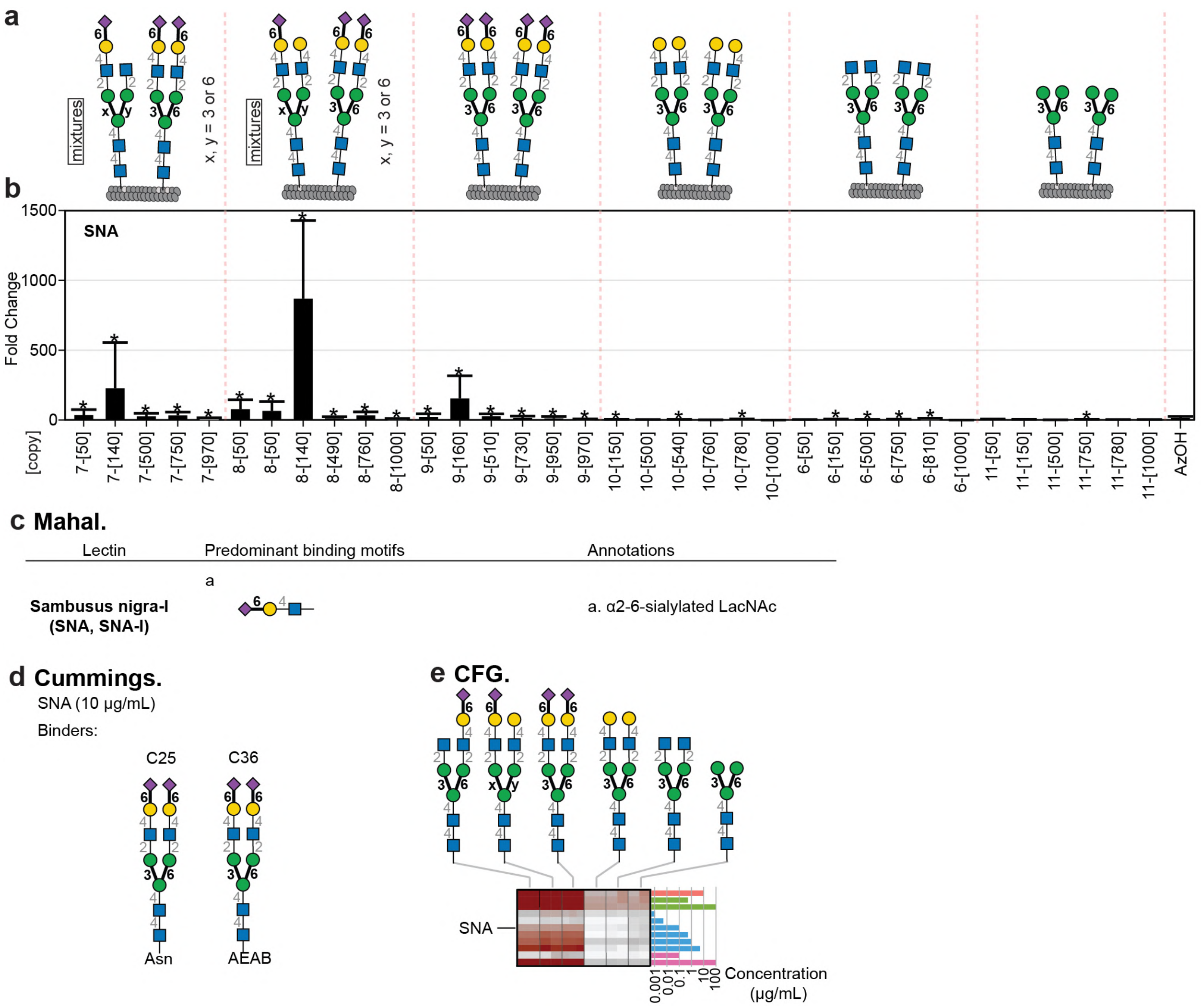

Supplementary Figure. 29: Binding of LiGA6×5 library to SNA-I.

a, LiGA6×5 components, same as Fig. 5. **b,** Bars show binding to SNA-I calculated by edgeR as the best-fit fold change (FC) differential enrichment (DE) of each glycophage clone in SNA-I coated wells when compared to BSA coated wells; error bars represent standard deviation propagated from the variance of the normalized sequencing data. * denotes significantly enriched reads by edgeR's DE analysis with FDR $\leq$ 0.05 (from n = 5 independent SNA binding experiments compared to n = 13 independent BSA-binding control experiments). **c,** Expected SNA-I binding specificities as described by Mahal and co-workers[4].**d,** SNA-I binding to a glass-based *N*-glycan array measured by Cummings and co-workers[5]. **e,** SNA-I binding to *N*-glycans on glass-based arrays produced by the Consortium for Functional Glycomics.

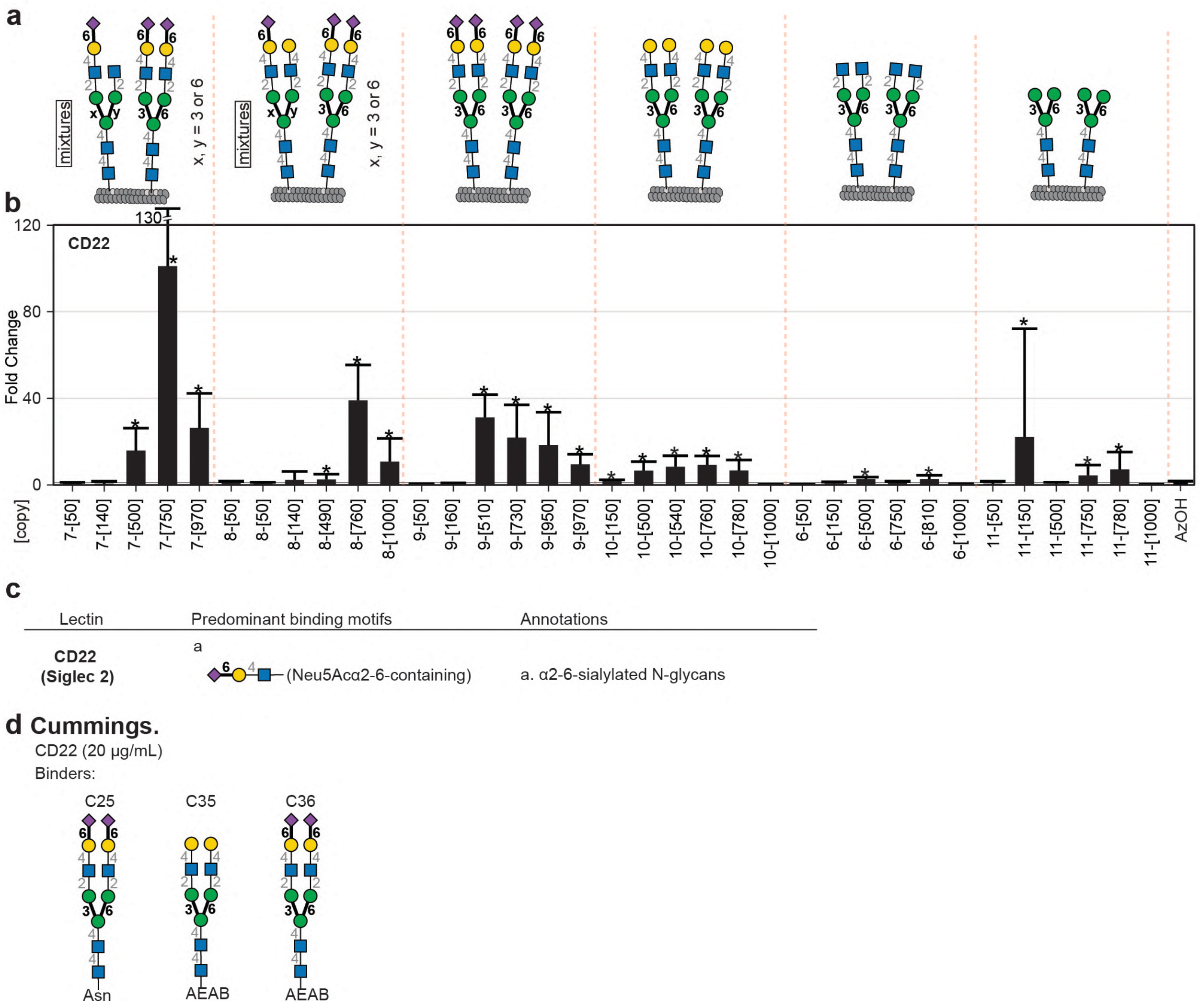

Supplementary Figure. 30: Binding of LiGA6×5 library to CD22 (Siglec-2).
a, The component of LiGA6×5, same as Fig. 5. **b,** Bars show binding to CD22 calculated by edgeR as the best-fit fold change (FC) differential enrichment (DE) of each glycophage clone in CD22 coated wells when compared to BSA coated wells; error bars represent s.d. propagated from the variance of the normalized sequencing data. * denotes significantly enriched reads in DE analysis with FDR ≤ 0.05 (n = 5 (SNA), 13 (BSA) independent binding experiments). **c,** Expected CD22 binding specificities as described by Mahal and co-workers. **d,** CD22 binding to glass-based *N*-glycan array measured by Cummings and co-workers[5].

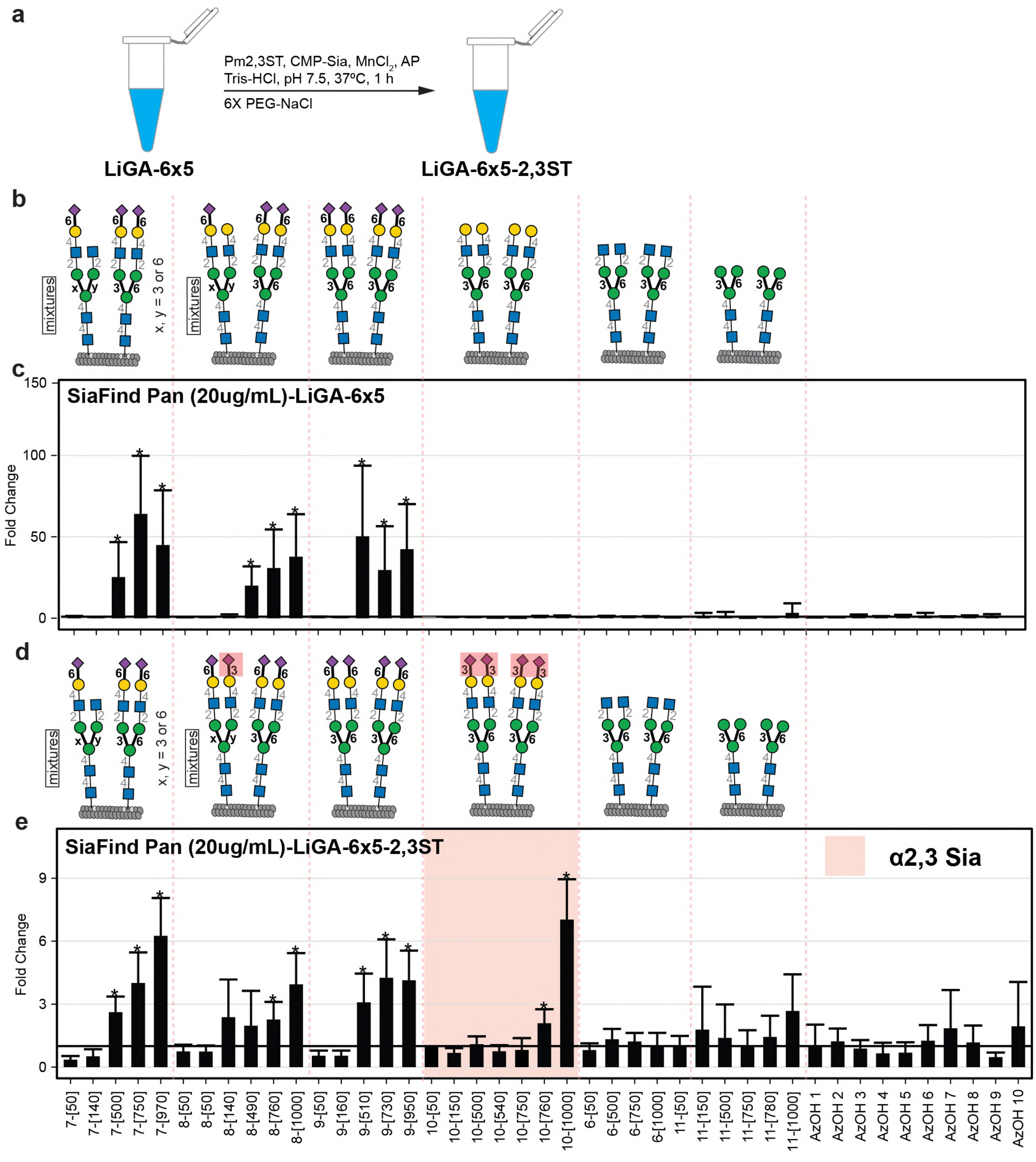

Supplementary Figure. 31: Binding of LiGA6×5 to SiaFind™ Pan-specific Lectenz® before and after remodeling by Pm2,3ST.

a, The remodeling procedure as described in Supplementary Figure 30. **b,** Structures displayed on phage as LiGA6×5. **c,** When there is only α-(2→6)-sialosides of LiGA6×5, we observed binding to Pan-specific Lectenz. Binding to Pan-specific Lectenz was calculated as FC of each glycophage clone in Pan-specific Lectenz coated wells when compared to BSA coated wells. * denotes significantly enriched reads (FDR ≤ 0.05, n = 5 (Pan-specific Lectenz), 5 (BSA) independent

binding experiments). Error bars represent s.d. propagated from the variance of the normalized sequencing data. **d,** Structures displayed on phage of LiGA6×5-2,3ST. **e,** We observed binding of Pan-specific Lectenz towards both to α-(2→6)-sialosides and newly installed α-(2→3)-sialosides. Binding to Pan-specific Lectenz was calculated as FC of each glycophage clone in Pan-specific Lectenz coated wells when compared to BSA coated wells. * denotes significantly enriched reads (FDR ≤ 0.05, n = 5 (Pan-specific Lectenz), 5 (BSA) independent binding experiments). Error bars represent s.d. propagated from the variance of the normalized sequencing data.

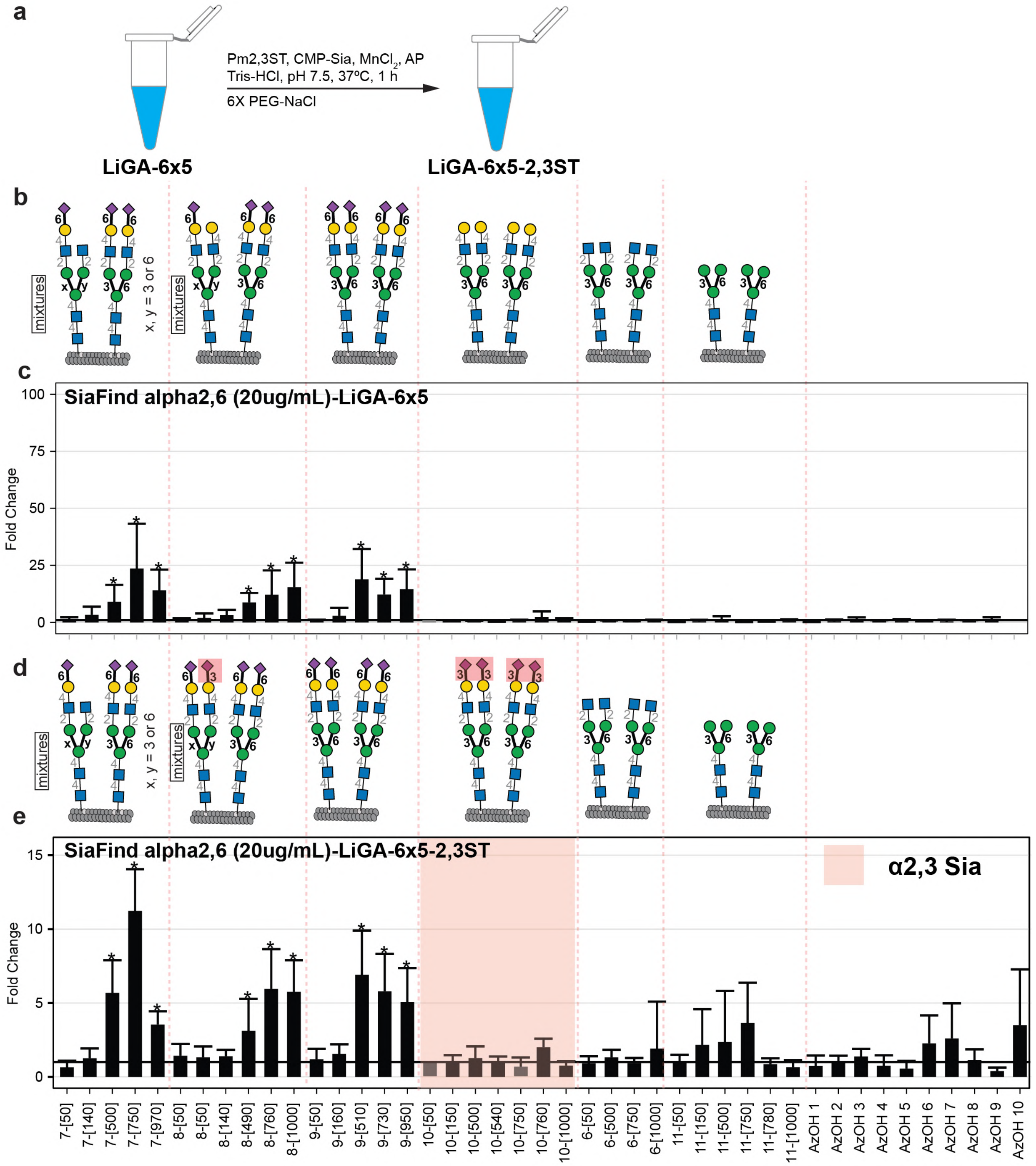

Supplementary Figure. 32: Binding of LiGA6×5 to SiaFind™ α-(2→6)-specific reagent before and after remodeling by Pm2,3ST.

a, The remodeling procedure was described in Supplementary Figure 30. **b,** Structures displayed on phage as LiGA6×5. **c,** We observed binding of α-(2→6)-sialosides to SiaFind™ α-(2→6)-specific reagent. Binding to SiaFind™ α-(2→6)-specific reagent was calculated as FC of each glycophage clone in SiaFind™ α-(2→6)-specific reagent coated wells when compared to BSA coated wells. * denotes significantly enriched reads (FDR ≤ 0.05, n = 5 (SiaFind™ α-(2→6)-

specific reagent), 5 (BSA) independent binding experiments). Error bars represent s.d. propagated from the variance of the normalized sequencing data. **d,** Structures displayed on phage of LiGA6×5-2,3ST. **e,** Unlike SiaFind™ Pan-specific Lectenz® (Supplementary Figure 31) or diCBM40 (Supplementary Figure 30), SiaFind™ α-(2→6)-specific reagent recognized only α-(2→6)-sialosides but newly installed α-(2→3)-sialosides did not show binding. Binding to SiaFind™ α-(2→6)-specific reagent was calculated as FC of each glycophage clone in SiaFind™ α-(2→6)-specific reagent coated wells when compared to BSA coated wells. * denotes significantly enriched reads (FDR ≤ 0.05, n = 5 (SiaFind™ α-(2→6)-specific reagent), 5 (BSA) independent binding experiments). Error bars represent s.d. propagated from the variance of the normalized sequencing data.

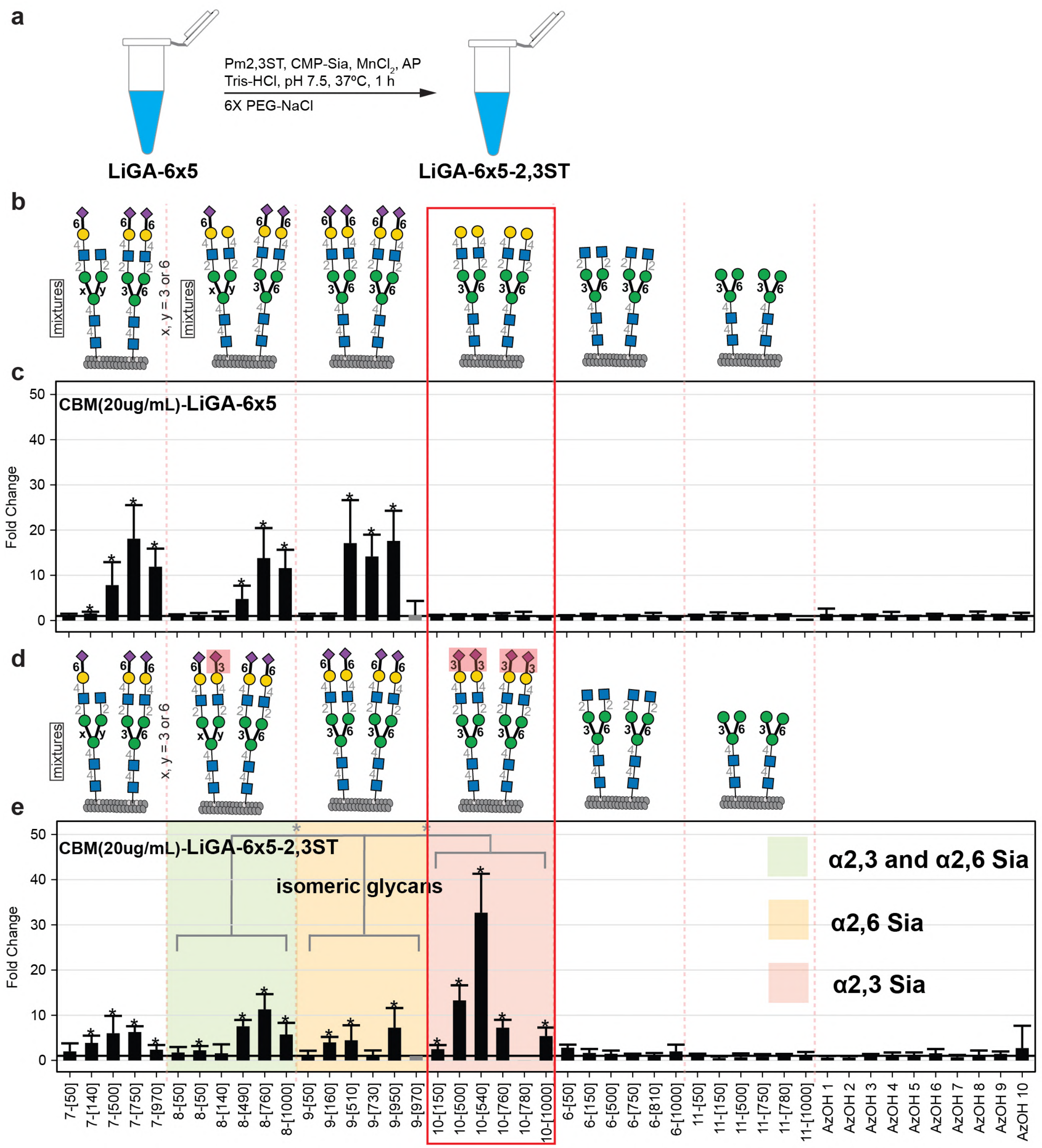

Supplementary Figure. 33: Binding of LiGA6×5 to sialic acid-specific diCBM40 lectin before and after remodeling by Pm2,3ST.

a, The LiGA6×5 was treated with Pm2,3ST to transfer CMP-sialic acid onto Gal-terminal *N*-glycans displayed on phage. After 1 h incubation at 37 °C, PEG/NaCl was added to precipitate the phage. HBS buffer was used to dissolve the phage. Resulting solution, termed "LiGA6×5-2,3ST" was added to diCBM40 coated well to validate the binding of newly introduced α-(2→3)-linked sialic acids to diCBM40 **b,** Structures displayed on phage of LiGA6×5. **c,** Binding of unmodified LiGA6×5 to diCBM40: α-(2→6)-sialosides exhibited enrichment by diCBM40, because

diCBM40 can recognize α-2,6-sialosides with weak affinity[6]. Binding to diCBM40 was calculated as FC of each glycophage clone in diCBM coated wells when compared to BSA coated wells. * denotes significantly enriched reads (FDR ≤ 0.05, n =5 independent binding experiments for binding to diCBM and BSA). Error bars represent s.d. propagated from the variance of the normalized sequencing data. **d,** Structures displayed on phage in LiGA6×5-2,3ST. **e,** Binding of modified LiGA6×5-2,3ST to diCBM40: we observed strong binding of diCBM40 towards α-(2→3)-sialosides on phage that contained only terminal Gal prior to modification (red rectangle). In a set of isomeric glycans, pure α-(2→6) (yellow) binds weaker than pure α-(2→3) (red) and mix of α-(2→3) and α-(2→6)-sialosides (green). Increased binding to α-(2→3) versus α-(2→6)-sialosides is consistent with known preferences of diCBM40 for α-(2→3) linkage. Binding to diCBM40 was calculated as FC of each glycophage clone in diCBM40 coated wells when compared to BSA coated wells. * denotes significantly enriched reads (FDR ≤ 0.05, n =5 independent binding experiments for binding to diCBM and BSA). Error bars represent s.d. propagated from the variance of the normalized sequencing data.

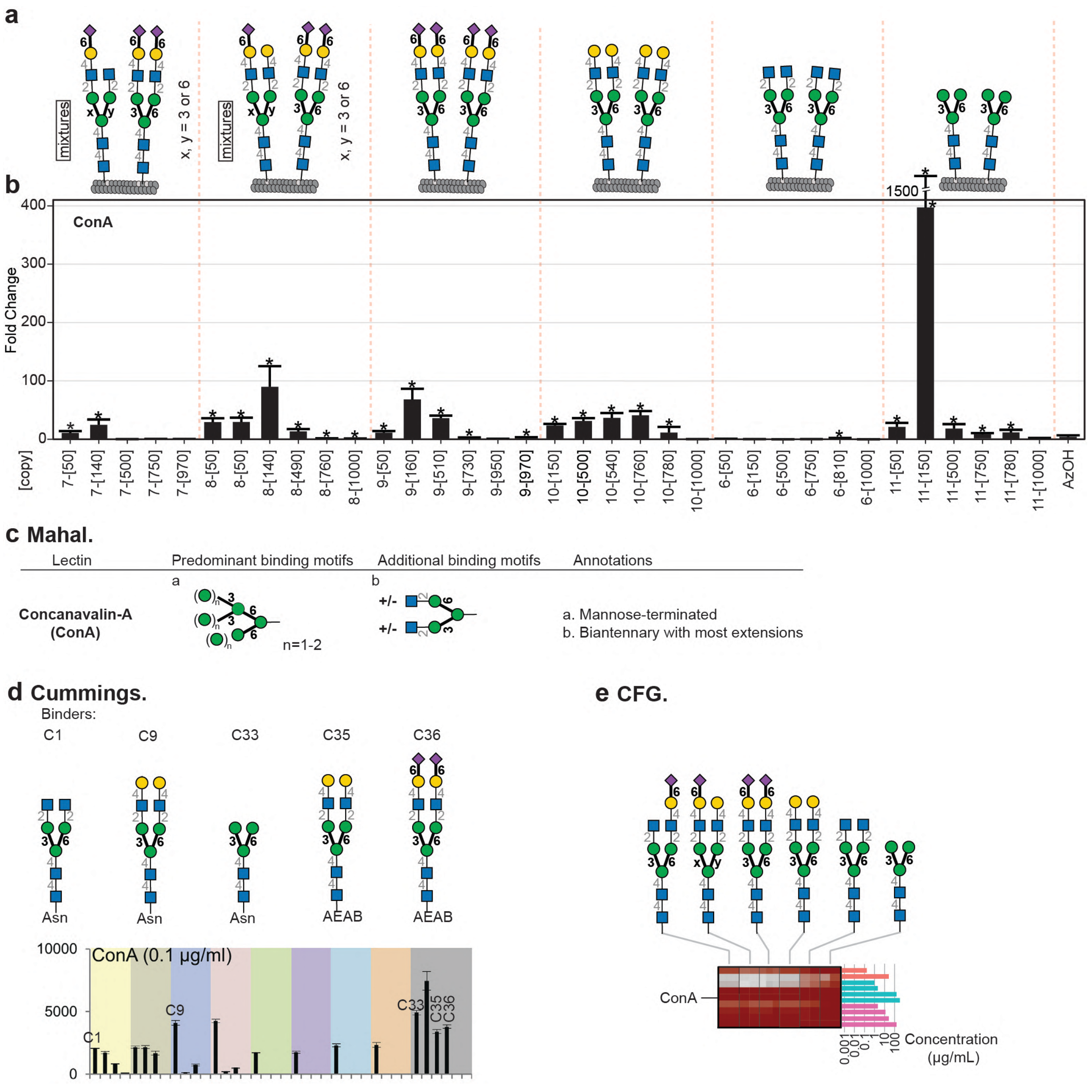

Supplementary Figure. 34: Binding of LiGA6×5 library to ConA.
a, The component of LiGA6×5, same as Fig. 5. **b,** Bars show binding to ConA calculated by edgeR as the best-fit fold change (FC) differential enrichment (DE) of each glycophage clone in ConA coated wells when compared to BSA coated wells; error bars represent s.d. propagated from the variance of the normalized sequencing data. * denotes significantly enriched reads (FDR ≤ 0.05, $n = 8$ (ConA), 13 (BSA) independent binding experiments). **c,** Expected ConA binding specificities as described by Mahal and co-workers[4]. **d,** ConA binding to a glass-based *N*-glycan array measured by Cummings and co-workers[5]. **e,** ConA binding to *N*-glycans on glass-based arrays produced by the Consortium for Functional Glycomics[4].

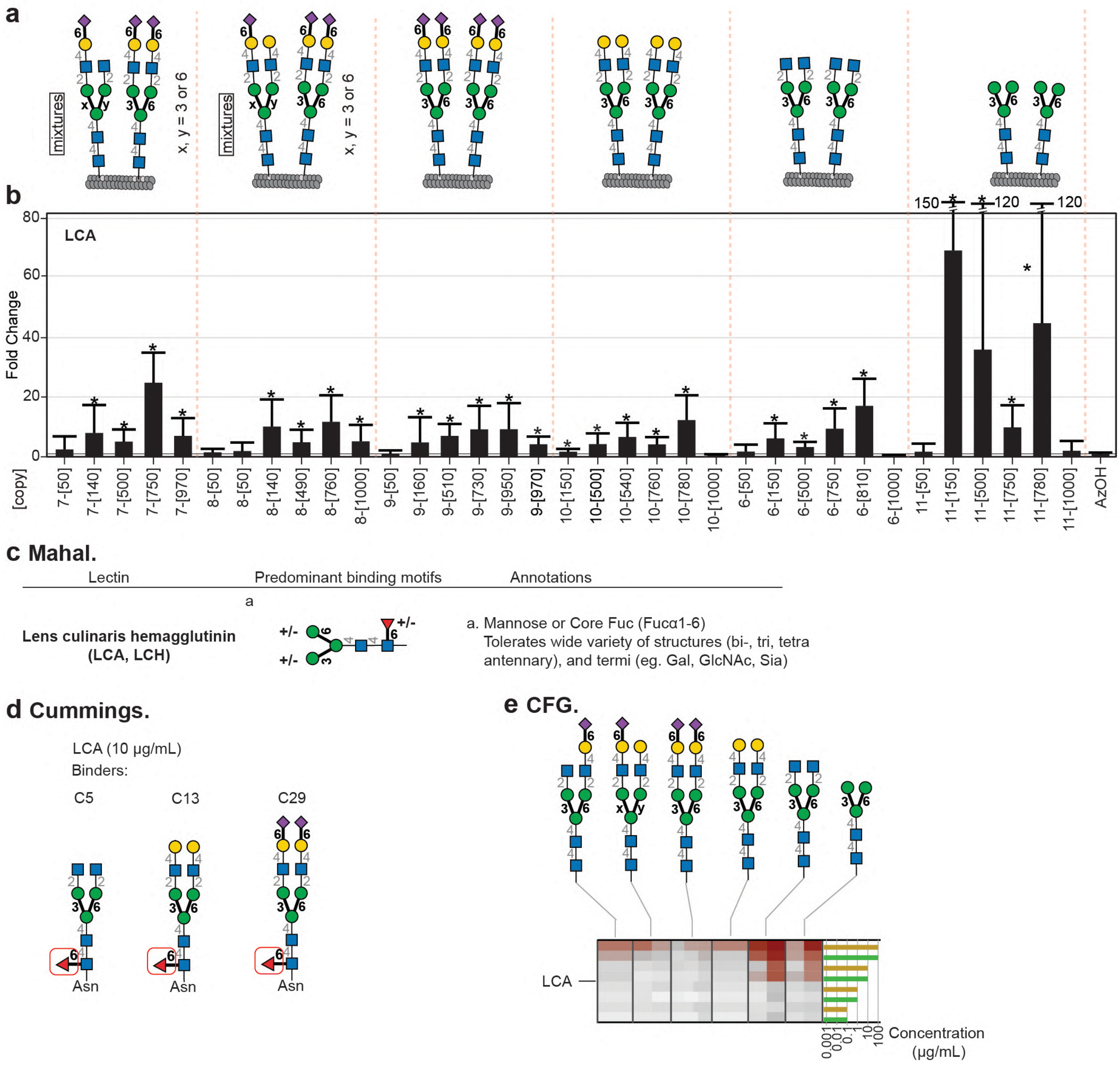

Supplementary Figure. 35: Binding of LiGA6×5 library to LCA.

a, The component of LiGA6×5, same as Fig. 5. **b,** Bars show binding to LCA calculated by edgeR as the best-fit fold change (FC) differential enrichment (DE) of each glycophage clone in LCA coated wells when compared to BSA coated wells; error bars represent s.d. propagated from the variance of the normalized sequencing data. * denotes significantly enriched reads with FDR ≤ 0.05. (n = 6 (LCA), 13 (BSA) independent binding experiments**c,** Expected LCA binding specificities as described by Mahal and co-workers[4]. **d,** LCA binding to a glass-based *N*-glycan array measured by Cummings and co-workers[5]. **e,** LCA binding to *N*-glycans on glass-based arrays produced by the Consortium for Functional Glycomics[4].

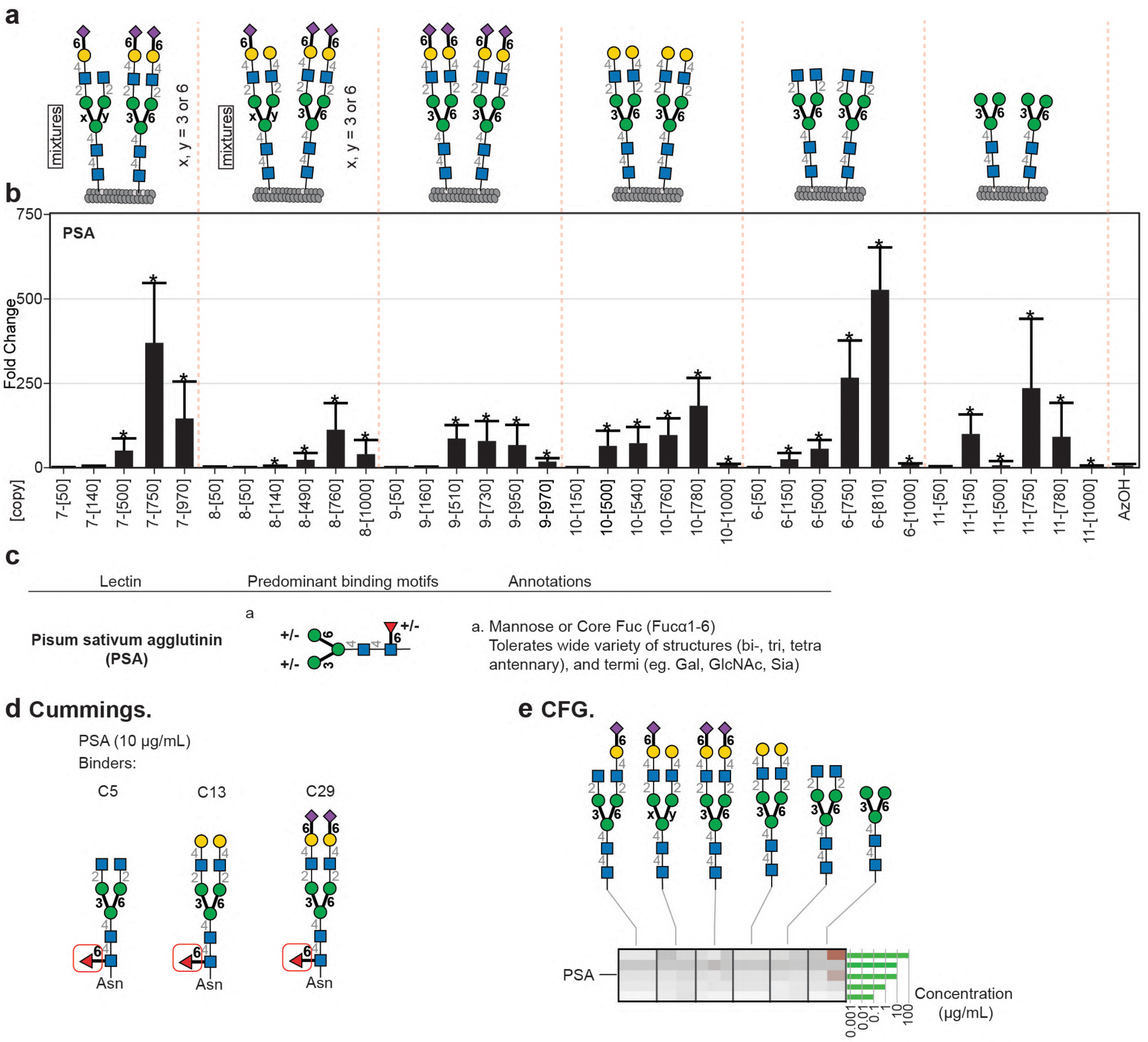

Supplementary Figure. 36: Binding of LiGA6×5 library to PSA.

a, The component of LiGA6×5, same as Fig. 5. **b,** Bars show binding to PSA calculated by edgeR as the best-fit fold change (FC) differential enrichment (DE) of each glycophage clone in PSA coated wells when compared to BSA coated wells. error bars represent s.d. propagated from the variance of the normalized sequencing data. * denotes significantly enriched reads with FDR ≤ 0.05 (n = 5 (PSA), 13 (BSA) independent binding experiments). **c,** Expected PSA binding specificities as described by Mahal and co-workers[4]. **d,** PSA binding to a glass-based *N*-glycan array measured by Cummings and co-workers[5]. **e,** PSA binding to *N*-glycans on glass-based arrays produced by the Consortium for Functional Glycomics[4].

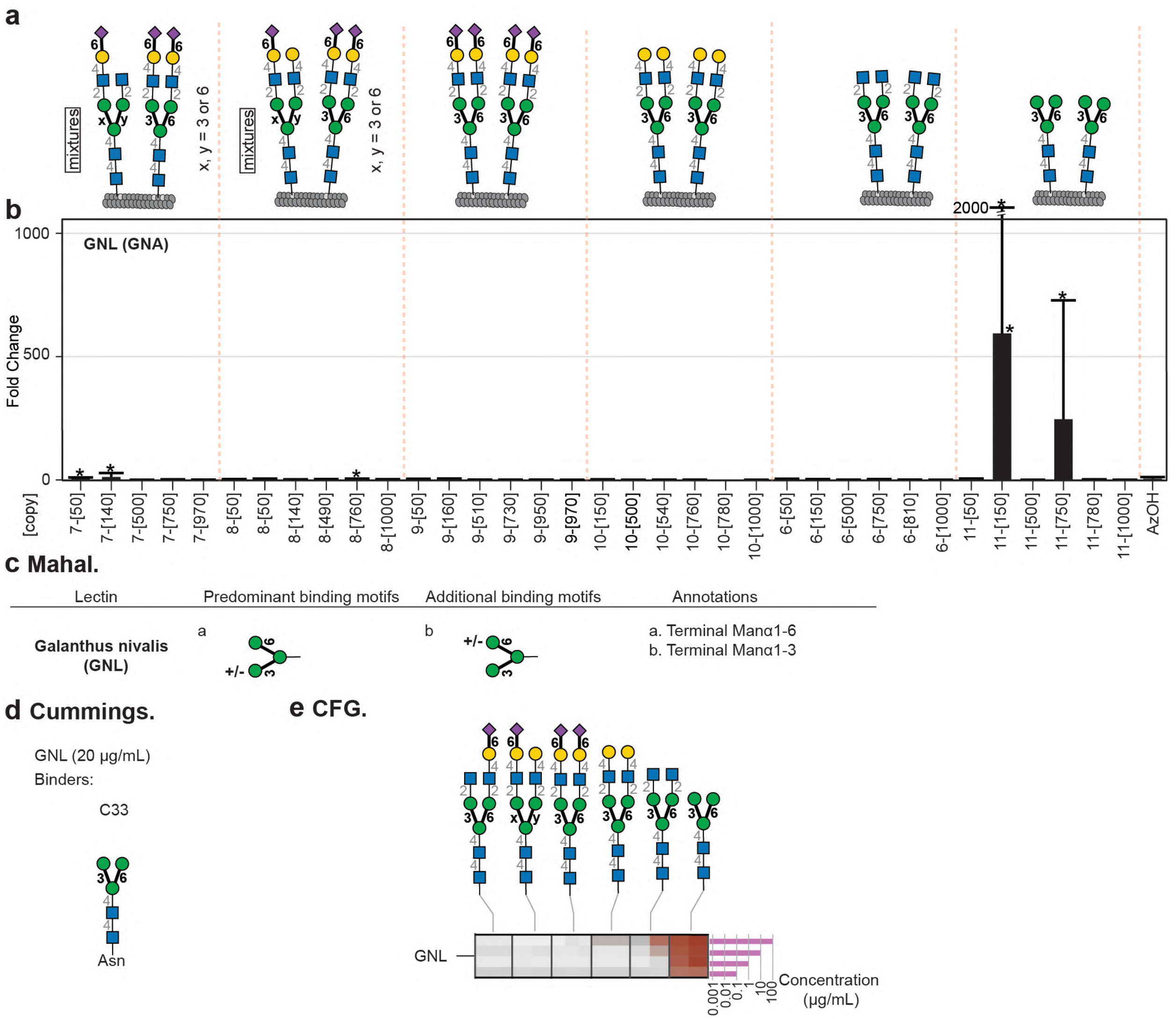

Supplementary Figure. 37: Binding of LiGA6×5 library to GNL (GNA).

a, The component of LiGA6×5, same as Fig. 5. **b,** Bars show binding to GNL calculated by edgeR as the best-fit fold change (FC) differential enrichment (DE) of each glycophage clone in GNL coated wells when compared to BSA coated wells; error bars represent s.d. propagated from the variance of the normalized sequencing data. * denotes significantly enriched reads with FDR ≤ 0.05 (n = 5 (GNL), 13 (BSA) independent binding experiments). **c,** Expected GNL binding specificities as described by Mahal and co-workers[4]. **d,** GNL binding to a glass-based *N*-glycan array measured by Cummings and co-workers[5]. **e,** GNL binding to *N*-glycans on glass-based arrays produced by the Consortium for Functional Glycomics[4].

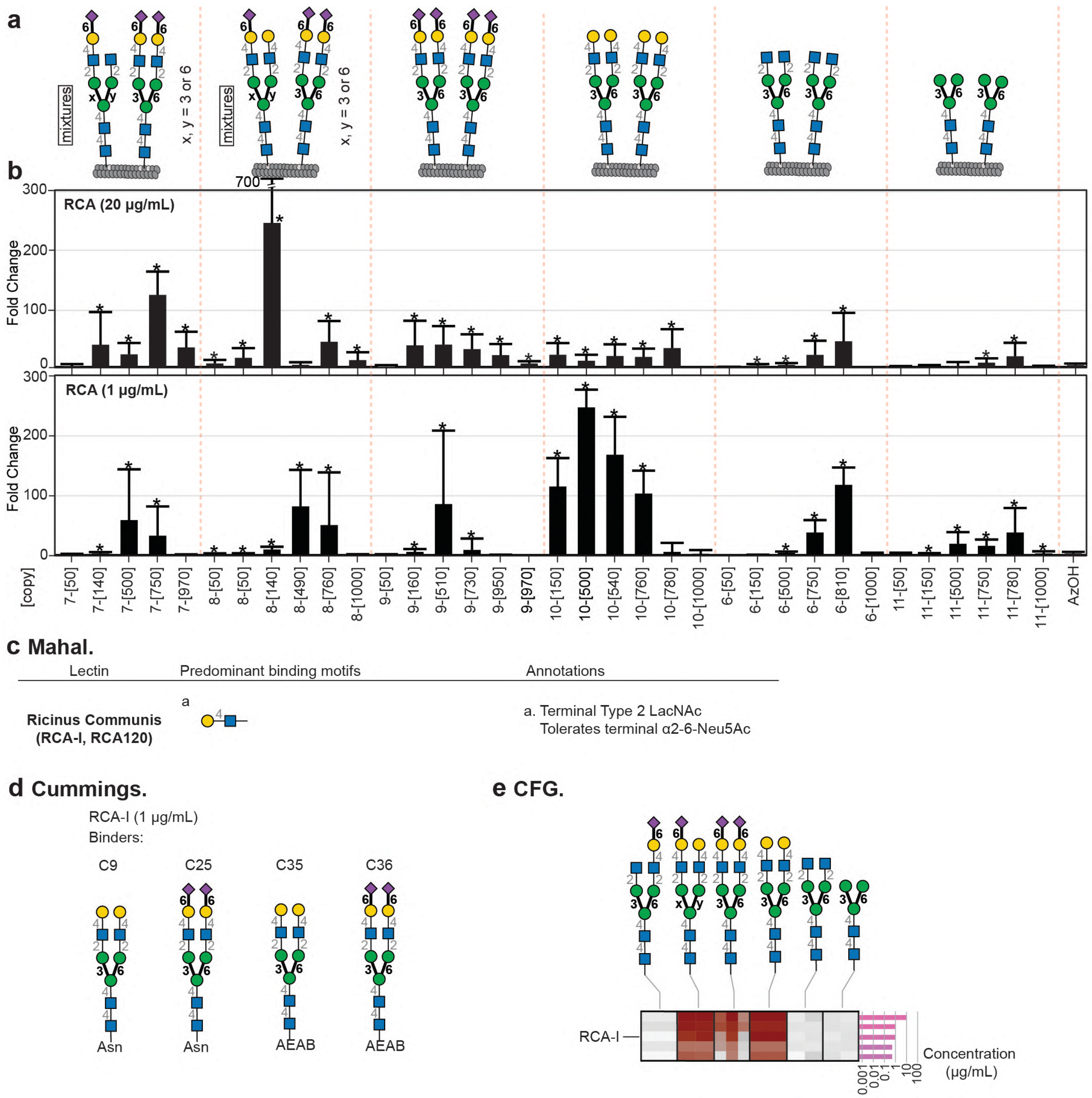

Supplementary Figure. 38: Binding of LiGA6×5 library to RCA-I.

a, The component of LiGA6×5, same as Fig. 5. **b,** Bars show binding to RCA-I calculated by edgeR as the best-fit fold change (FC) differential enrichment (DE) of each glycophage clone in RCA-I coated wells when compared to BSA coated wells; error bars represent s.d. propagated from the variance of the normalized sequencing data. * denotes significantly enriched (black) or depleted (pink) reads in DE analysis with FDR ≤ 0.05 (n = 5 (RCA-I 1 µg/mL) compared to 11 (BSA) and independent binding experiments and 5 (RCA-I 20 µg/mL) compared to 13 (BSA) independent experiments). **c,** Expected RCA-I binding specificities as described by Mahal and co-workers[4]. **d,** RCA-I binding to a glass-based N-glycan array measured by Cummings and co-workers[5]. **e,** RCA-I binding to *N*-glycans on glass-based arrays produced by the Consortium for Functional Glycomics[4].

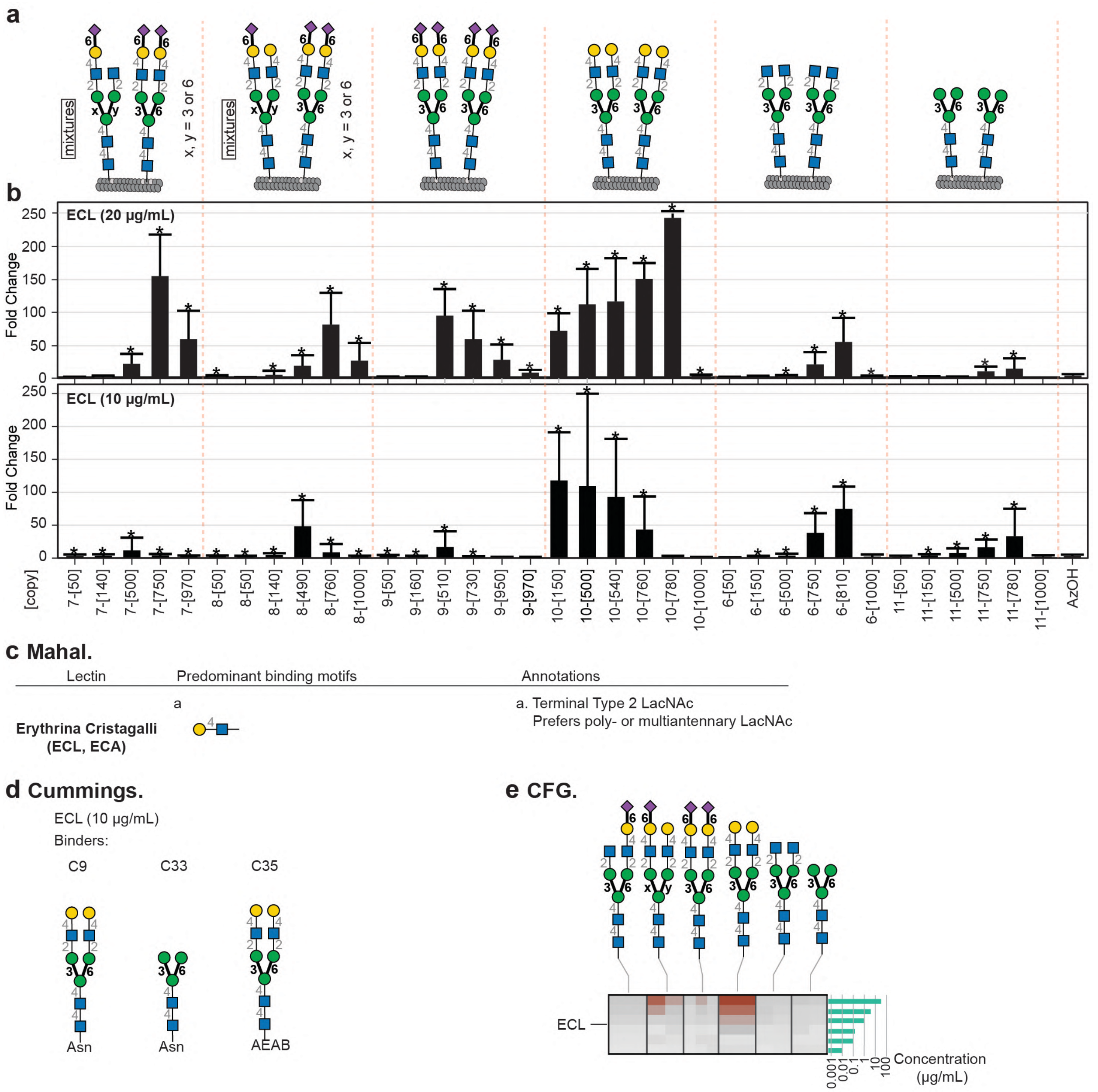

Supplementary Figure. 39: Binding of LiGA6×5 library to ECL.

a, The component of LiGA6×5, same as Fig. 5. **b,** Bars show binding to ECL calculated by edgeR as the best-fit fold change (FC) differential enrichment (DE) of each glycophage clone in ECL coated wells when compared to BSA coated wells; error bars represent s.d. propagated from the variance of the normalized sequencing data. * denotes significantly enriched reads with FDR ≤ 0.05 (n = 5 (ECL 10 µg/mL) compared to n = 11 (BSA) and n = 5 (ECL 20 µg/mL) compared to n = 13 (BSA) - all independent binding experiments). **c,** Expected ECL binding specificities as described by Mahal and co-workers[4]. **d,** ECL binding to a glass-based N-glycan array measured by Cummings and co-workers[5]. **e,** ECL binding to *N*-glycans on glass-based arrays produced by the Consortium for Functional Glycomics[4].

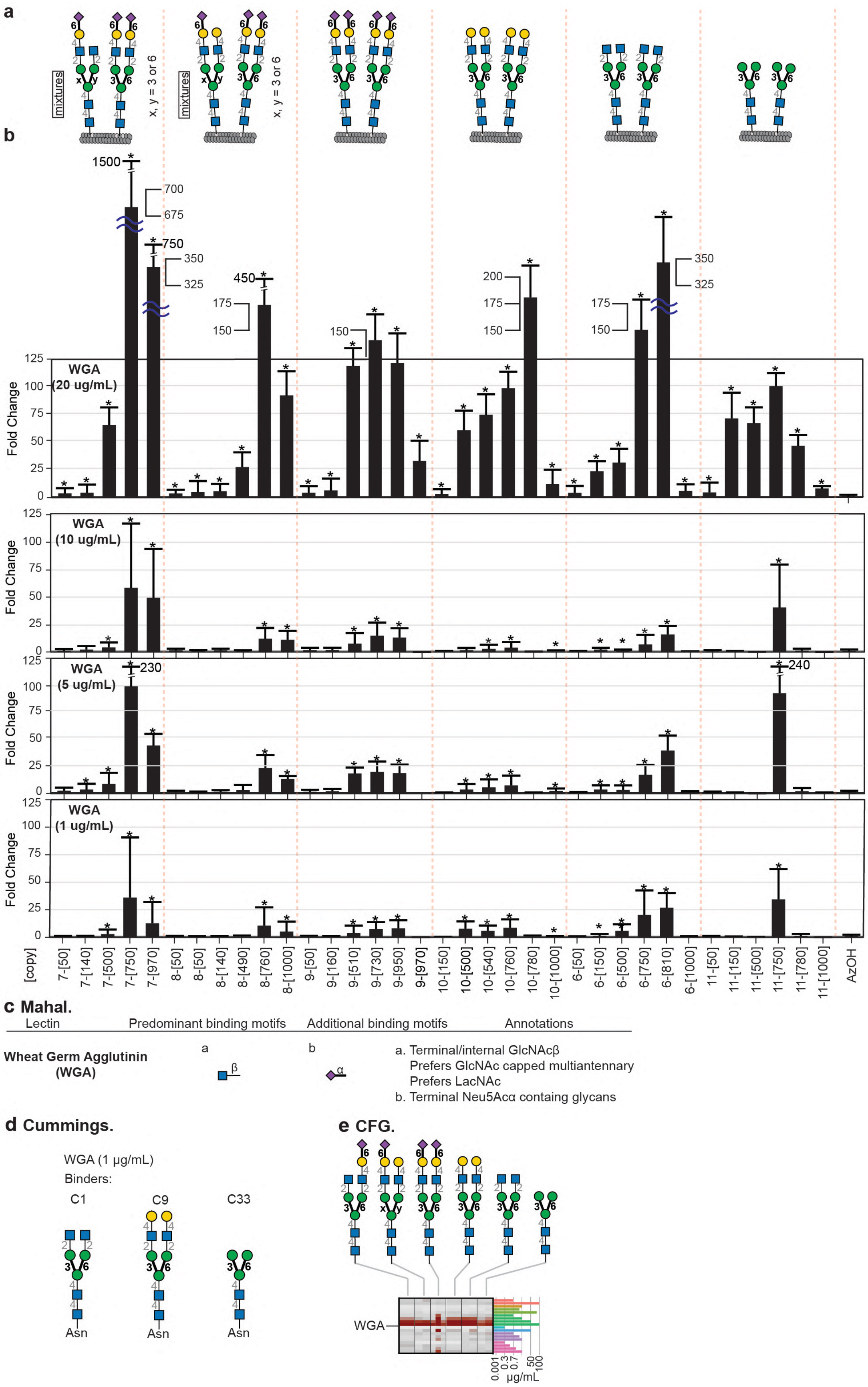

a
b
mixtures
x, y = 3 or 6
WGA (20 ug/mL)
WGA (10 ug/mL)
WGA (5 ug/mL)
WGA (1 ug/mL)
Fold Change
[copy]
7-[50] 7-[140] 7-[500] 7-[750] 7-[970] 8-[50] 8-[50] 8-[140] 8-[490] 8-[760] 8-[1000] 9-[50] 9-[160] 9-[510] 9-[730] 9-[950] 9-[970] 10-[150] 10-[500] 10-[540] 10-[760] 10-[780] 10-[1000] 6-[50] 6-[150] 6-[500] 6-[750] 6-[810] 6-[1000] 11-[50] 11-[150] 11-[500] 11-[750] 11-[780] 11-[1000] AzOH
c Mahal.
Lectin
Predominant binding motifs
Additional binding motifs
Annotations
Wheat Germ Agglutinin (WGA)
a. Terminal/internal GlcNAcβ
Prefers GlcNAc capped multiantennary
Prefers LacNAc
b. Terminal Neu5Acα containg glycans
d Cummings.
WGA (1 μg/mL)
Binders:
C1
C9
C33
Asn
e CFG.
WGA
μg/mL

(legend on the next page)

Supplementary Figure. 40: Binding of LiGA6×5 library to WGA.

a, The component of LiGA6×5, same as Fig. 5. **b,** Bars show binding to WGA calculated by edgeR as the best-fit fold change (FC) differential enrichment (DE) of each glycophage clone in WGA coated wells when compared to BSA coated wells; error bars represent s.d. propagated from the variance of the normalized sequencing data. * denotes significantly enriched reads with FDR ≤ 0.05 (n = 5 (WGA other), 11 (WGA 20 μg/mL), 13 (BSA) independent binding experiments). **c,** Expected WGA binding specificities as described by Mahal and co-workers[4]. **d,** WGA binding to a glass-based *N*-glycan array measured by Cummings and co-workers[5]. **e,** WGA binding to *N*-glycans on glass-based arrays produced by the Consortium for Functional Glycomics[4].

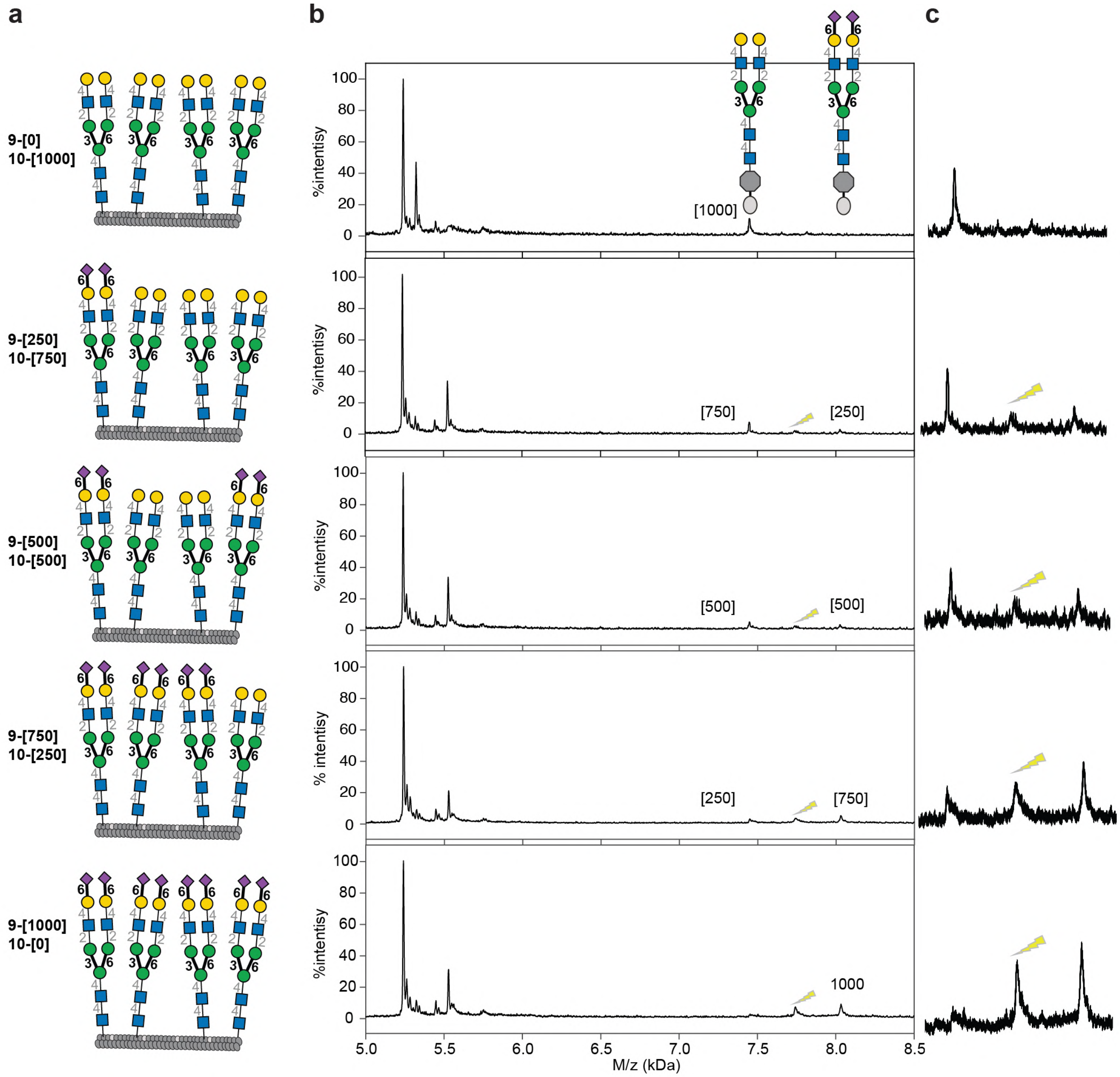

Supplementary Figure. 41: MALDI-TOF MS results of five phage conjugates for the study of steric occlusions.

a, Five different phage clones that contain different ratios of α-(2 →6)-sialylated and galactose-terminated *N*-glycans on the same phage viron. **b,** 1000 copy numbers of five DBCO modified phage clones were reacted with different ratio of α-(2 →6)-sialylated and galactose-terminated *N*-glycans. **c,** Expanded figure of MALDI-TOF MS analysis from **b.**

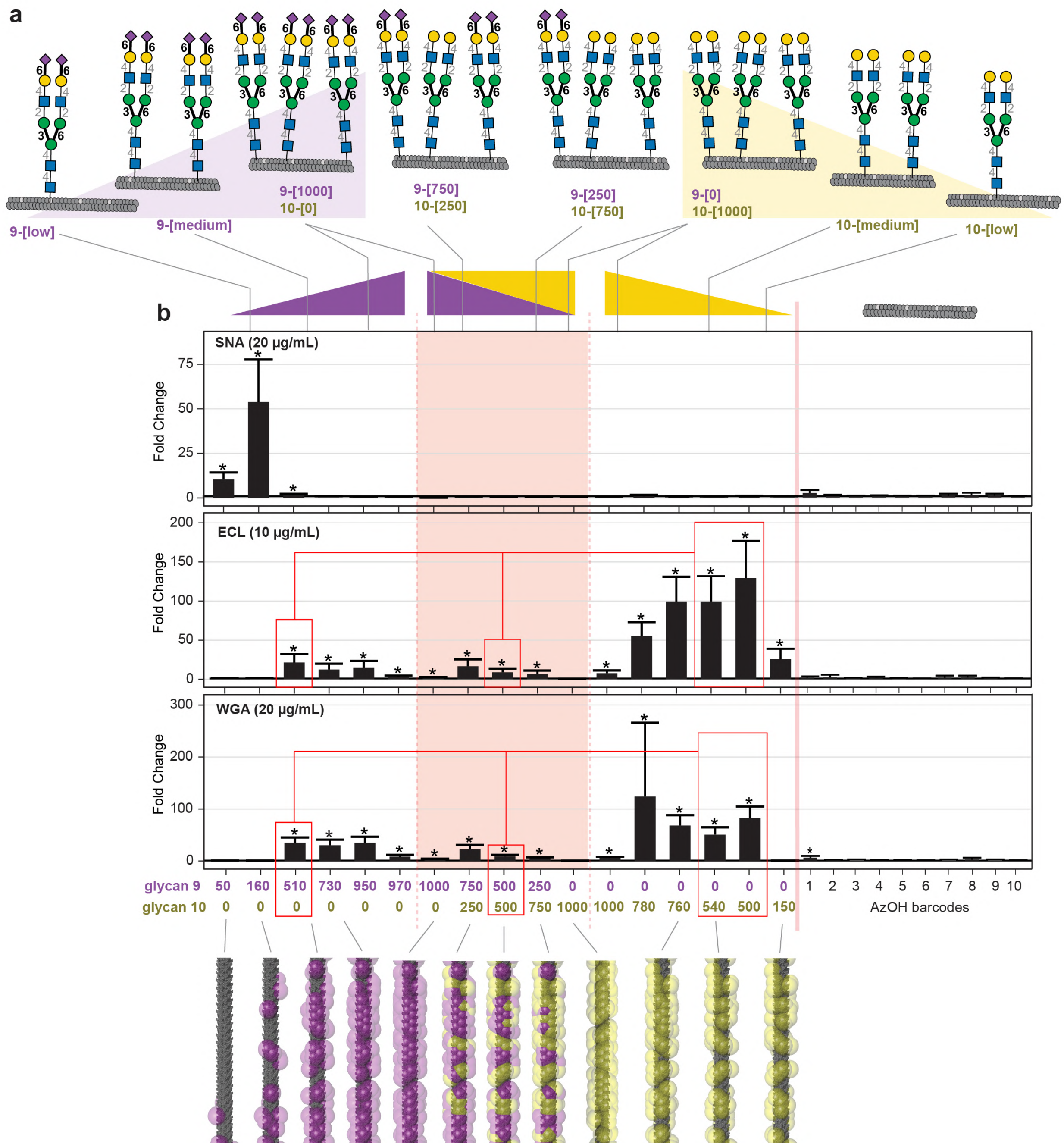

Supplementary Figure. 42: Steric occlusions in the interaction between lectins and *N*-glycans on phage.

a, To test the steric occlusion hypothesis, we produced LiGA-SO in which phage displayed either different densities of *N*-glycans **9** and **10** or mixtures of glycans **9** and **10** on the same phage. In the latter mixture, the total number of glycans remained 1000 glycans per phage but the ratio between **9** and **10** varied (see Supplementary Figure 35). **b,** Binding of the LiGA-SO to ECL, SNA and WGA demonstrated that binding of all lectins ceased when total density of glycans reached 1000 copies. Specifically, 500 copies of glycan **9** on phage exhibited strong binding to ECL and

WGA, 500 copies of glycan **10** exhibited only minor binding to the same lectins but when 500 copies of **9** and 500 copies of **10** were present on the same phage simultaneously, the binding was abrogated. Also, within the populations of phage that displayed 1000 copies of **9** and **10**, the strongest binding was for phage that displayed 750:250 ratio of **9** and **10** (Supplementary Figure 36b). Introduction of non-binding glycans on phage decreases the access to the productive glycans. This experiment confirmed that steric occlusions played a role in the decreased binding of lectins to high density of glycan-modified phages. Binding to each lectin (SNA, ECL, WGA) was calculated as FC of each glycophage clone in each lectin coated wells when compared to BSA coated wells. * denotes significantly enriched reads with FDR ≤ 0.05 (n = 5 independent binding experiments for binding to SNA, ECL, WGA and BSA). Error bars represent s.d. propagated from the variance of the normalized sequencing data.

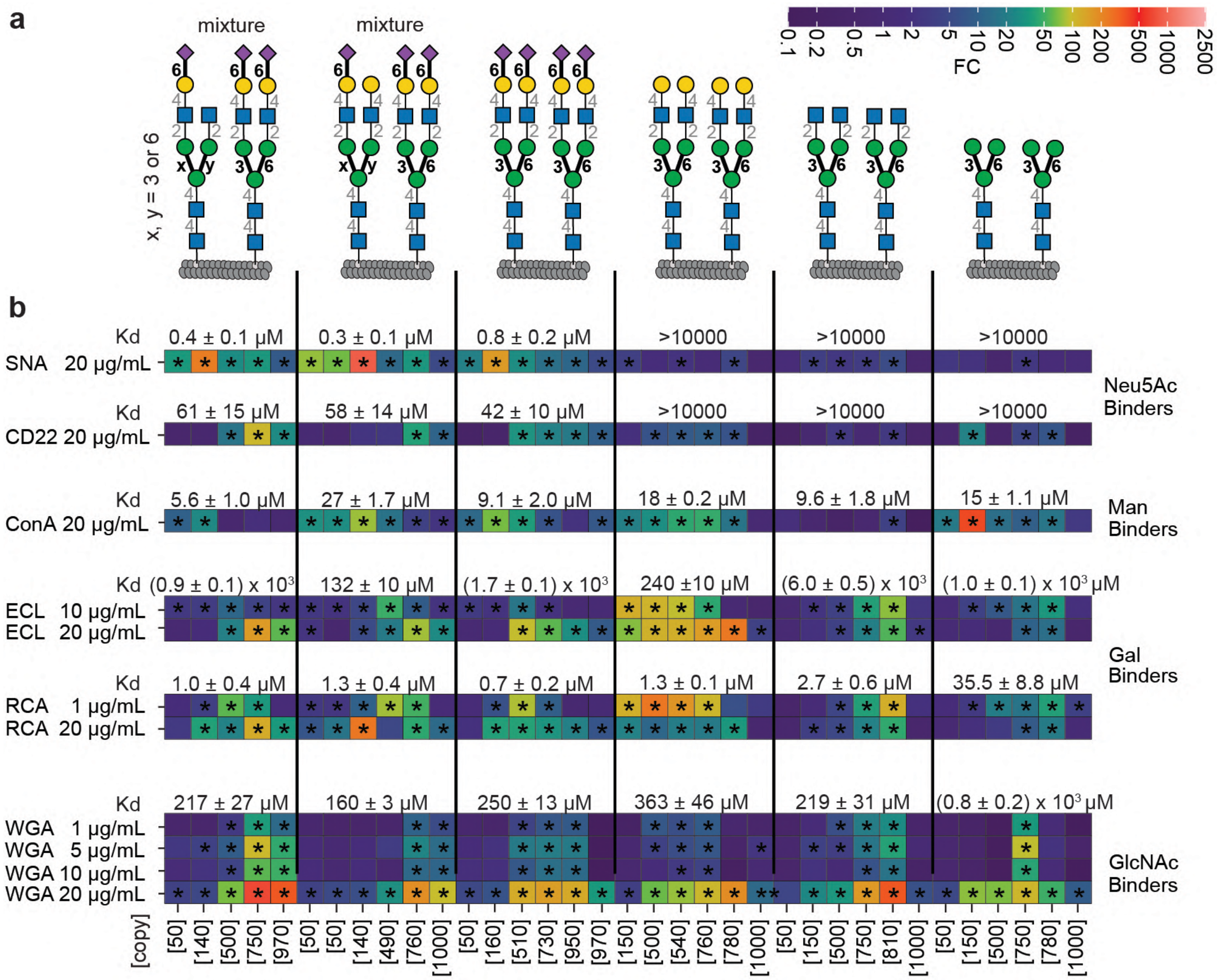

Supplementary Figure. 43: Binding measurements of each *N*-glycan with 6 lectins by Mass-spectrometry.

Synthetic *N*-glycans were used to measure the K_d valuesbetween monovalent *N*-glycan and a GBP binding site by ESI-MS. **a,** The structure of six synthetic *N*-glycans used in this measurement. **b,** The obtained K_d values of all *N*-glycans with each lectin was shown. (The unit of all K_d values is μM)

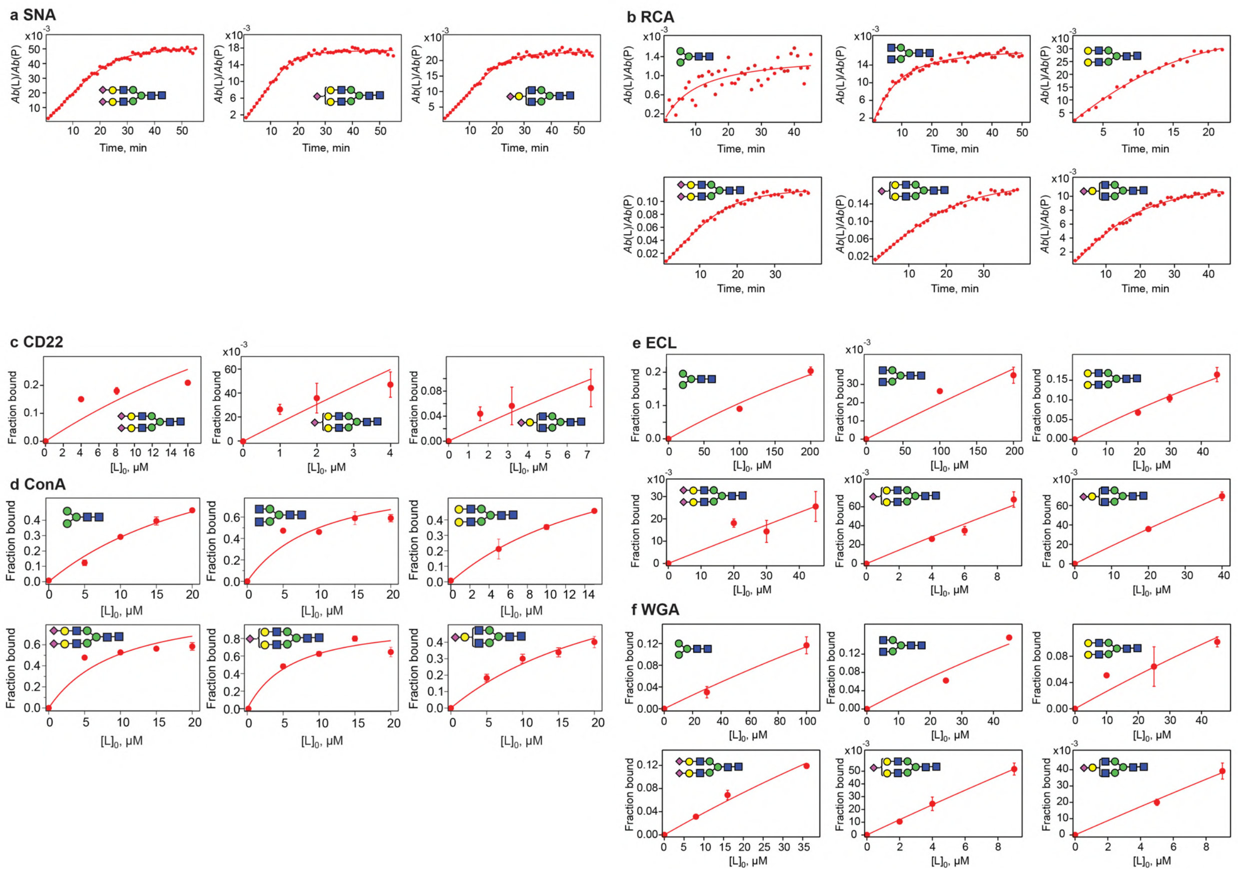

Supplementary Figure. 44: Titration curve data measured by Mass Spectrometry.
Time-resolved abundance ratio of the released glycan ligand ions to protein ion measured by COIN-CaR-nMS for **a,** SNA. **b,** RCA. Concentration-dependence of fraction of glycan ligand-bound protein measured by ESI-MS for **c,** CD22. **d,** ConA. **e,** ECL. **f,** WGA. **c-f,** All measurements were performed on solutions of aqueous ammonium acetate (200 µM, pH 7.0). Three technical replicates were performed (n = 3). Values represent the mean +/- SD.

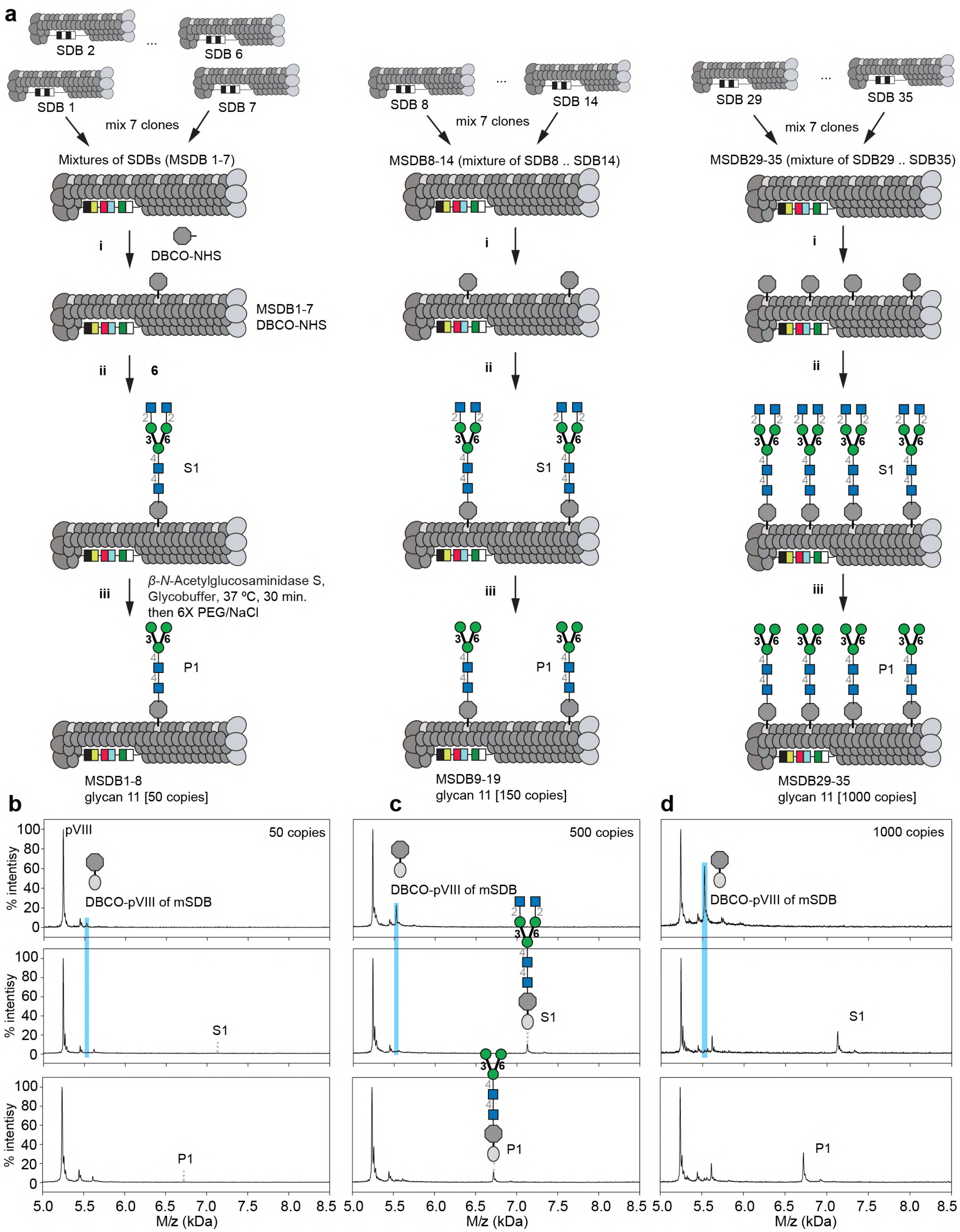
a
SDB 2
SDB 6
SDB 1
SDB 7
mix 7 clones
Mixtures of SDBs (MSDB 1-7)
i
DBCO-NHS
MSDB1-7
DBCO-NHS
ii
6
S1
iii
β-N-Acetylglucosaminidase S,
Glycobuffer, 37 ºC, 30 min.
then 6X PEG/NaCl
P1
MSDB1-8
glycan 11 [50 copies]
SDB 8
SDB 14
mix 7 clones
MSDB8-14 (mixture of SDB8 .. SDB14)
MSDB9-19
glycan 11 [150 copies]
SDB 29
SDB 35
mix 7 clones
MSDB29-35 (mixture of SDB29 .. SDB35)
MSDB29-35
glycan 11 [1000 copies]
b
c
d
pVIII
50 copies
500 copies
1000 copies
DBCO-pVIII of mSDB
% intentisy
M/z (kDa)

Supplementary Figure. 45: The workflow of creating MSDB glycoconjugates.
a-d, Each set of MSDB consisted of 7 phage clone with a unique DNA barcode and conjugated individually to DBCO-NHS. Low to high densities of DBCO-MSDB phage were created and conjugated to biantennary *N*-glycan **6**. After MALDI showed full conversion of conjugation to generate peak S1, *β-N-acetylglucosaminidase S* was added to cleave terminal GlcNAc yielding low to high densities of MSDB paucimannose-conjugates P1. These individual MSDB glycoconjugates were mixed with LiGA6×5 for binding experiments as shown in Supplementary Fig. 46, 48, and 49.

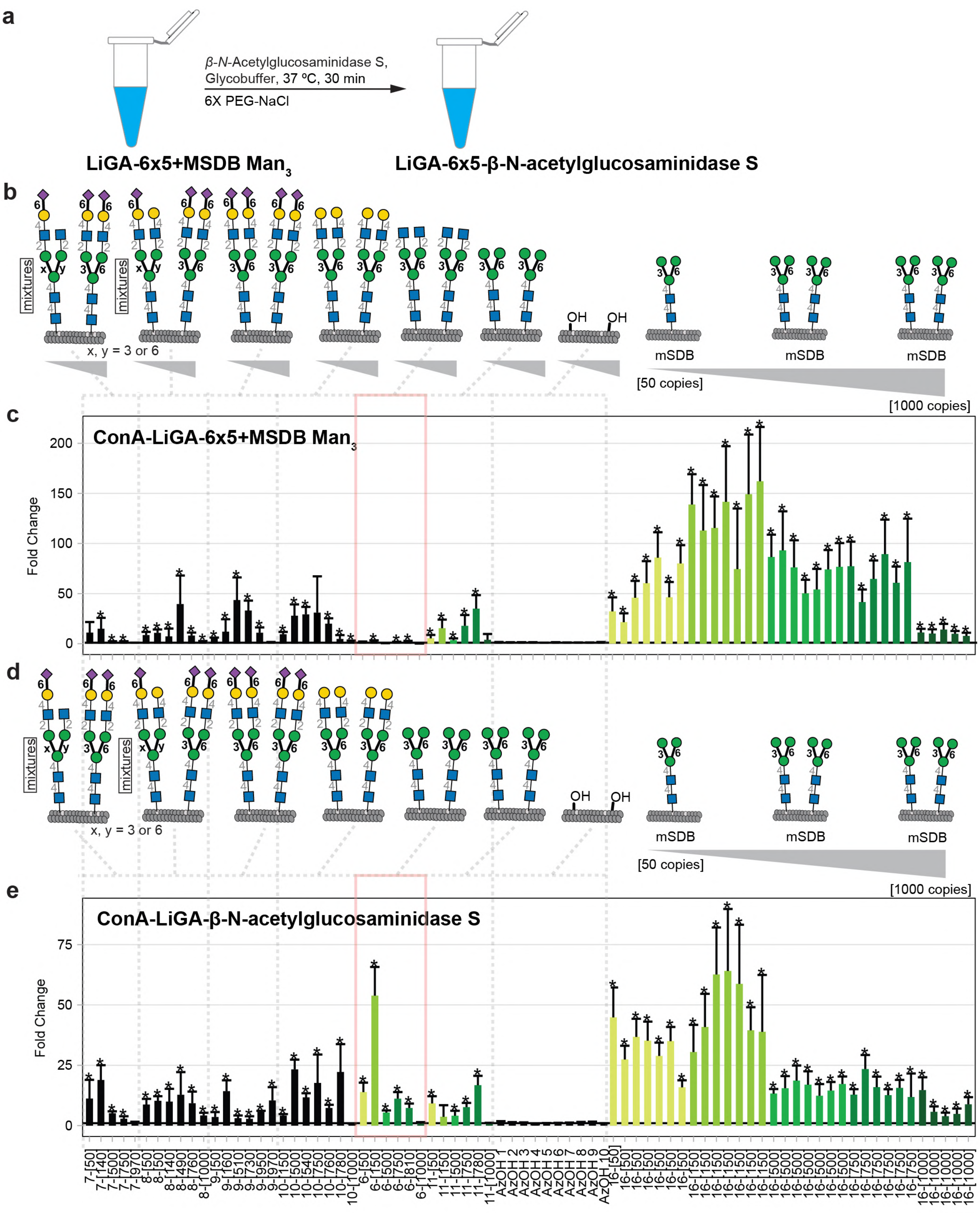

Supplementary Figure. 46: Binding of ConA to MSDB-paucimannose conjugates and LiGA6×5. **a,** The remodeling procedure was described in Supplementary Figure 30. **b,** We used the same mixing procedure as described in Supplementary Figure 38. then measured binding with ConA. **c,**

This MSDB-Man3 scan showed that binding of ConA to phage displaying 150 glycans is better than 50 or 500 and binding to any construct with 50–750 glycans per phage are better than binding to phage displaying 1000 glycans. Also, we again observed the phage clones that displayed 6 showed no binding, which is consistent with the results shown in Supplementary Figure 37. **d,** Structures displayed on phage of LiGA6×5- *β-N-acetylglucosaminidase S*. **e,** When the LiGA was treated with *β-N-acetylglucosaminidase S*, the non-binding clones that used to carry glycan 6 exhibited strong binding, due to removal of GlcNAc. In **c** and **e**, Binding to ConA was calculated as FC of each glycophage clone in ConA coated wells when compared to BSA coated wells. * denotes significantly enriched reads with FDR ≤ 0.05 (n = 5 independent binding experiments to ConA and BSA). Error bars represent s.d. propagated from the variance of the normalized sequencing data.

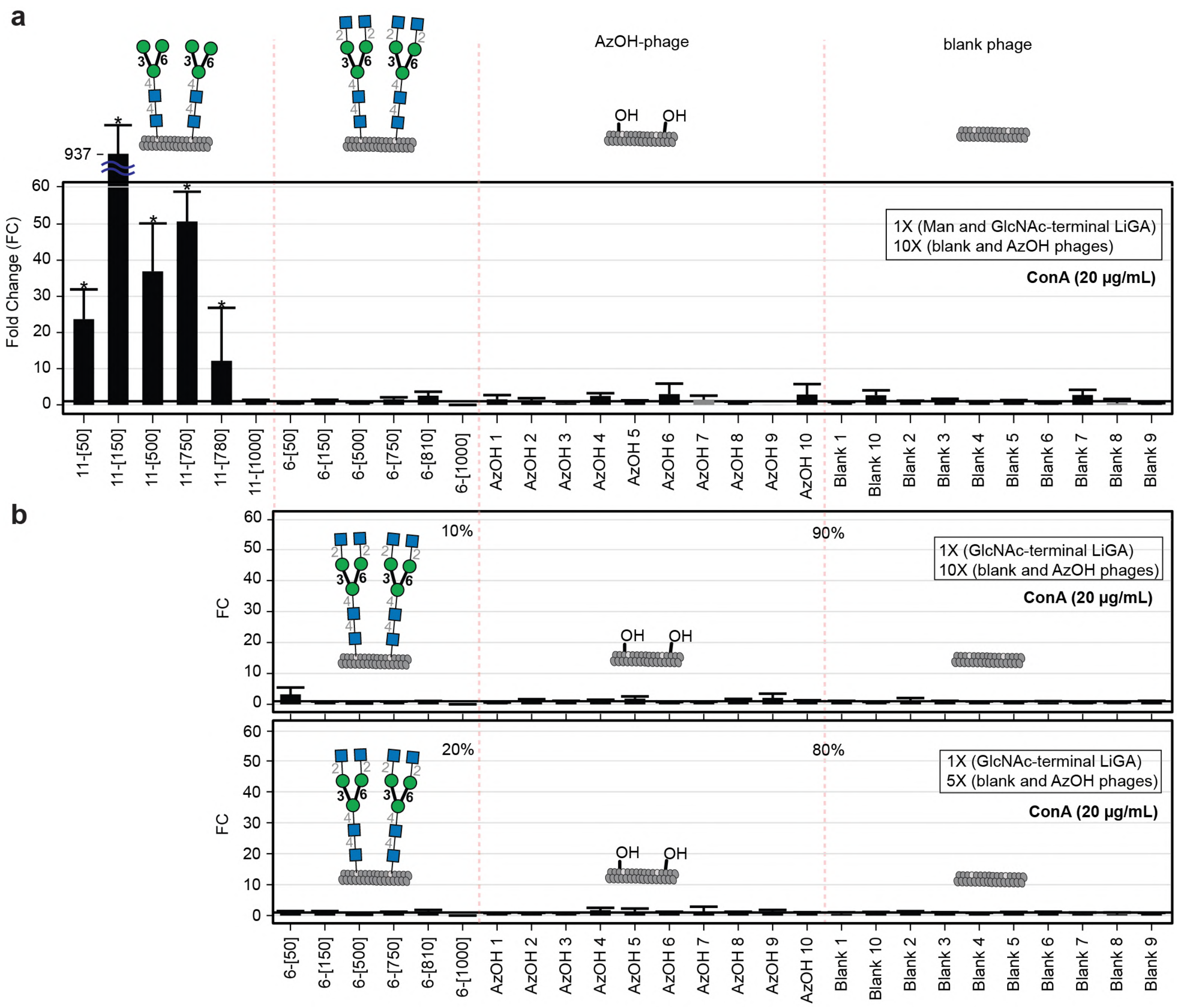

Supplementary Figure. 47: Comparison of LiGA-mixtures with and without binding paucimannose *N*-glycans.

a, We created LiGA2×6 which contained paucimannose biantennary *N*-glycans on phage with terminal GlcNAc (glycan **6**) and paucimannose alone (glycan **11**). The LiGA2×6 was screened against ConA. Only Man-terminated conjugates were enriched. **b,** To test whether presence of strong binder interferes with binding of weaker binder **6** on phage, we made another LiGA mixture without paucimannose. Glycan **6** on phage did not bind to ConA even when glycan **11** on phage was not present. We tested two different ratios between phage displaying glycan **6** and control phage. Both results showed no binding. In **a** and **b**, Binding to ConA was calculated as FC of each glycophage clone in ConA coated wells when compared to BSA coated wells. * denotes significantly enriched reads with FDR ≤ 0.05 (n = 5 independent binding experiments to ConA and BSA). Error bars represent s.d. propagated from the variance of the normalized sequencing data.

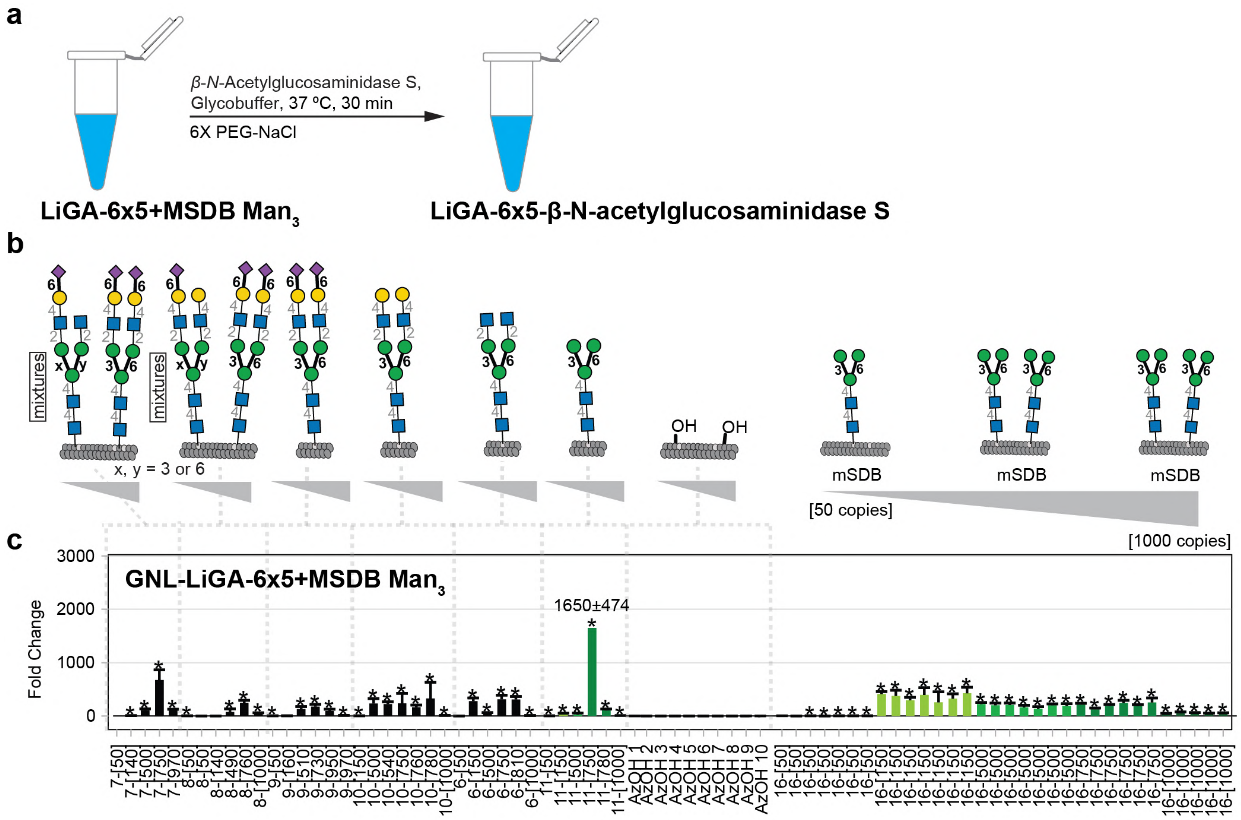

Supplementary Figure. 48: Binding of GNL by to MSDB-phage paucimannose conjugates and LiGA6×5.

a, The remodeling procedure was describe in Supplementary Fig. 33. **b,** We mixed 5 densities of MSDB-phage paucimannose conjugates with LiGA6×5, and measured binding with GNL. (Azidoethanol phage were used as control) **c,** Detailed study of using MSDB-Man3 showed that GNL bound paucimannose displayed on phage with intermediate density (150-750 copies). Binding to GNL was calculated as FC of each glycophage clone in GNL coated wells when compared to BSA coated wells. * denotes significantly enriched reads with FDR ≤ 0.05 (n = 5 independent binding experiments to GNL and BSA). Error bars represent s.d. propagated from the variance of the normalized sequencing data.

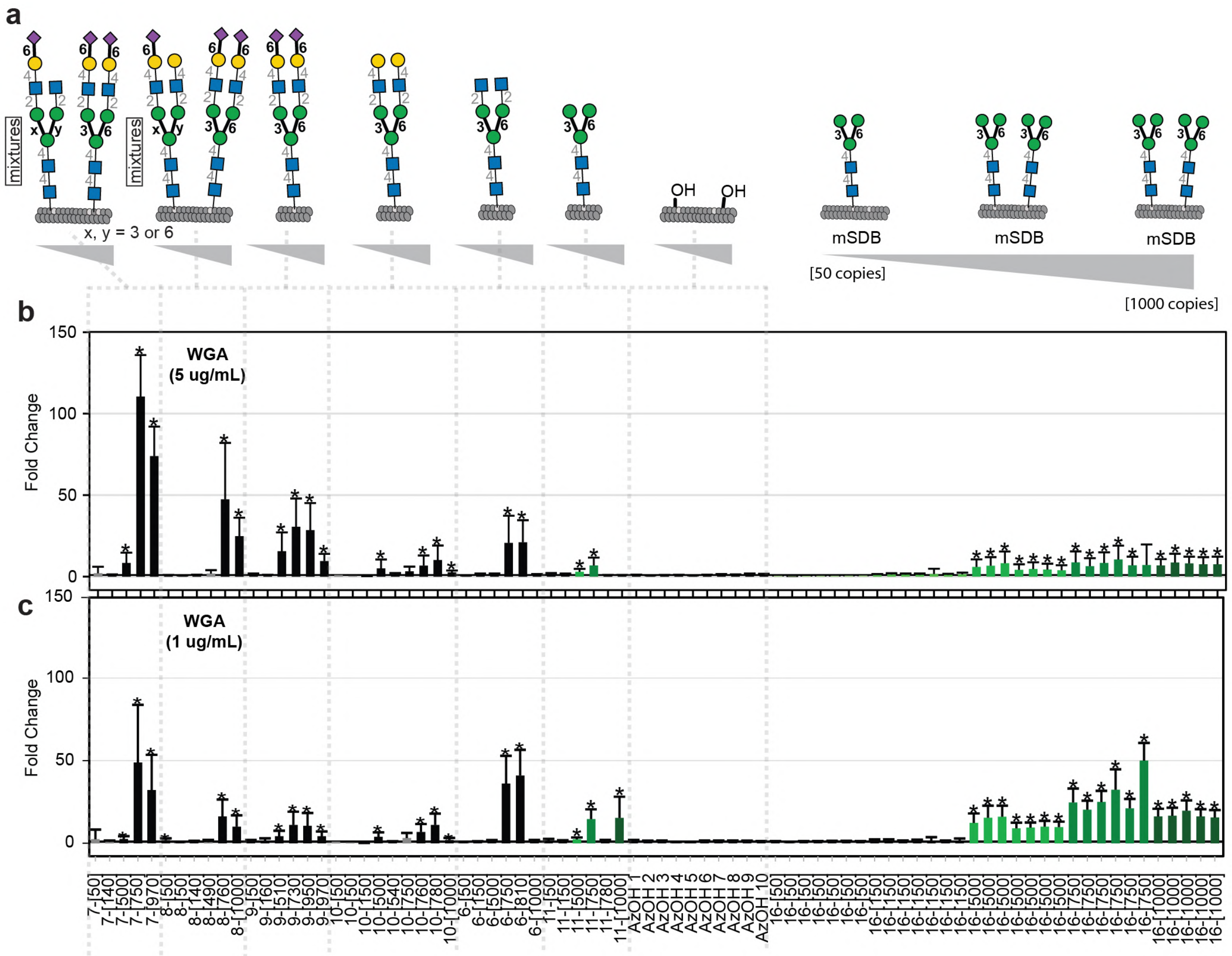

Supplementary Figure. 49: Binding of WGA by to MSDB-paucimannose conjugates and LiGA6×5.

a, We used the same procedure as described in Supplementary Fig. 45 then measured binding with WGA. **b-c,** We tested the significance of density dependent binding of WGA to paucimannose on phage by using MSDB-Man_3. We observed binding of WGA to MSDB-Man_3 at densities between 500 to 1000 glycans per phage. No binding occurred at densities of 50 and 150 copies of paucimannose. Binding to WGA was calculated as FC of each glycophage clone in WGA coated wells when compared to BSA coated wells. * denotes significantly enriched reads with FDR ≤ 0.05 (n = 5 independent binding experiments to WGA and BSA). Error bars represent s.d. propagated from the variance of the normalized sequencing data.

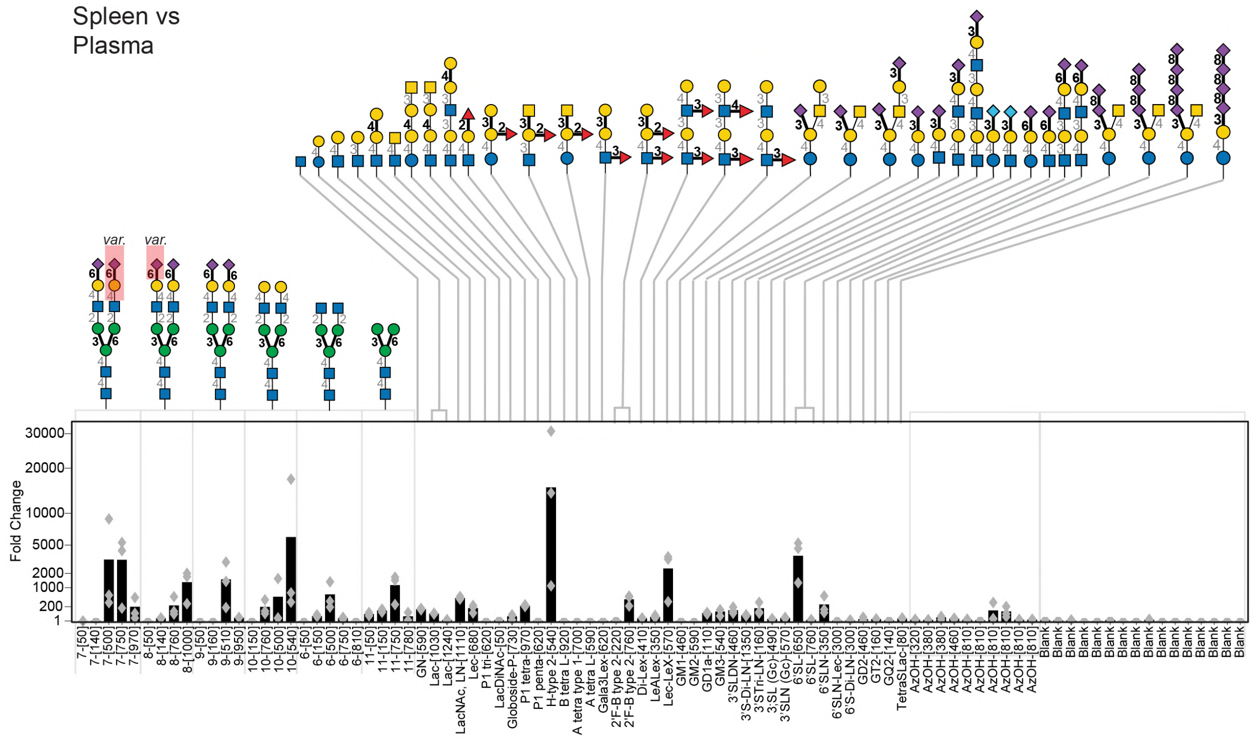

Supplementary Figure. 50: Summary of LiGA interaction with spleen *in-vivo* described as fold change enrichment in spleen with respect to plasma from the same animal.

The injection and processing of LiGA is described in Fig. 6 (main text); the *N*-glycan and blank (unmodified) phage data is mirrored from Figure 6b for consistency and replotted as a bar chart describing the mean FC across n = 3 mice with an overlaid grey points showing the FCs of the individual mice. The 6'SL-[650] is considerably enriched higher in liver, spleen and kidney compared to 6'SL-[760] (Supplementary Fig. 50-53) because of the relative low reads in the plasma samples.

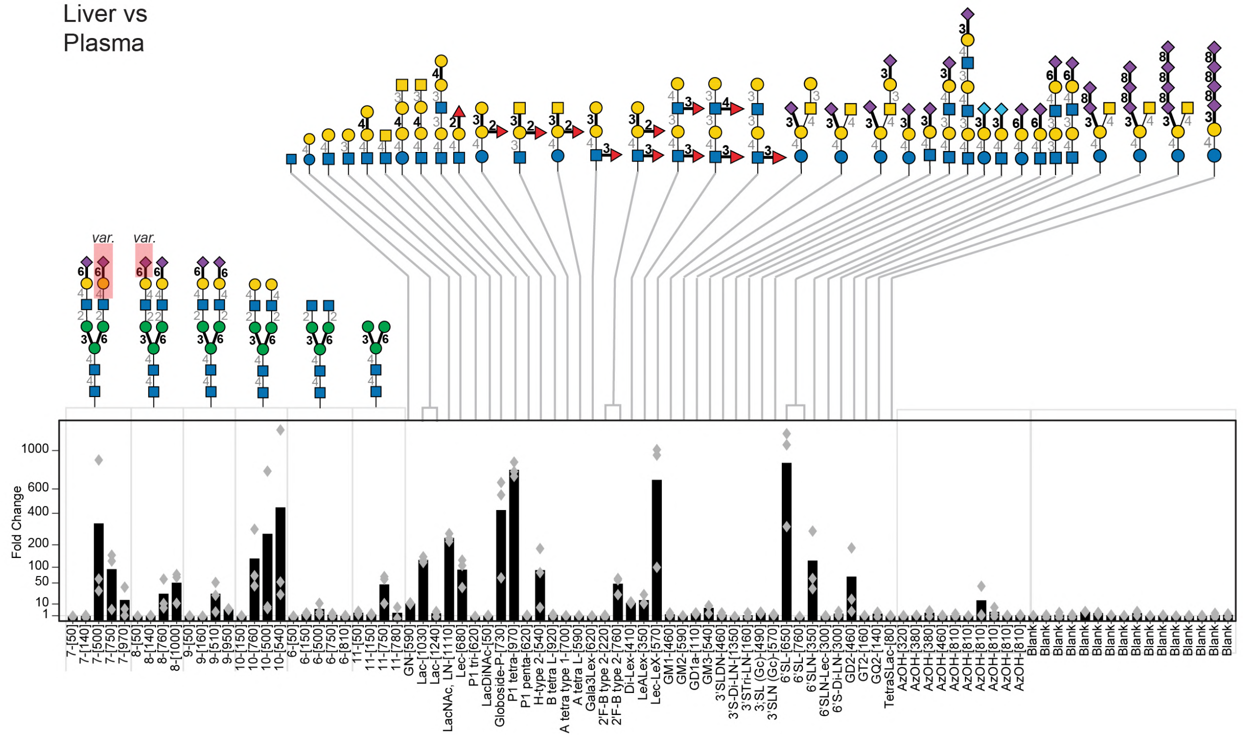

Supplementary Figure. 51: Summary of LiGA interaction with liver *in-vivo* described as fold change (FC) enrichment in liver with respect to plasma from the same animal.

The injection and processing of LiGA is described in Fig. 6 (main text); the *N*-glycan and blank (unmodified) phage data is the same as used to create Fig. 6b but analyzed separately for each mouse (n = 3). The grey diamonds show the fold change of each mouse and the mean of the three plotted as black bars.

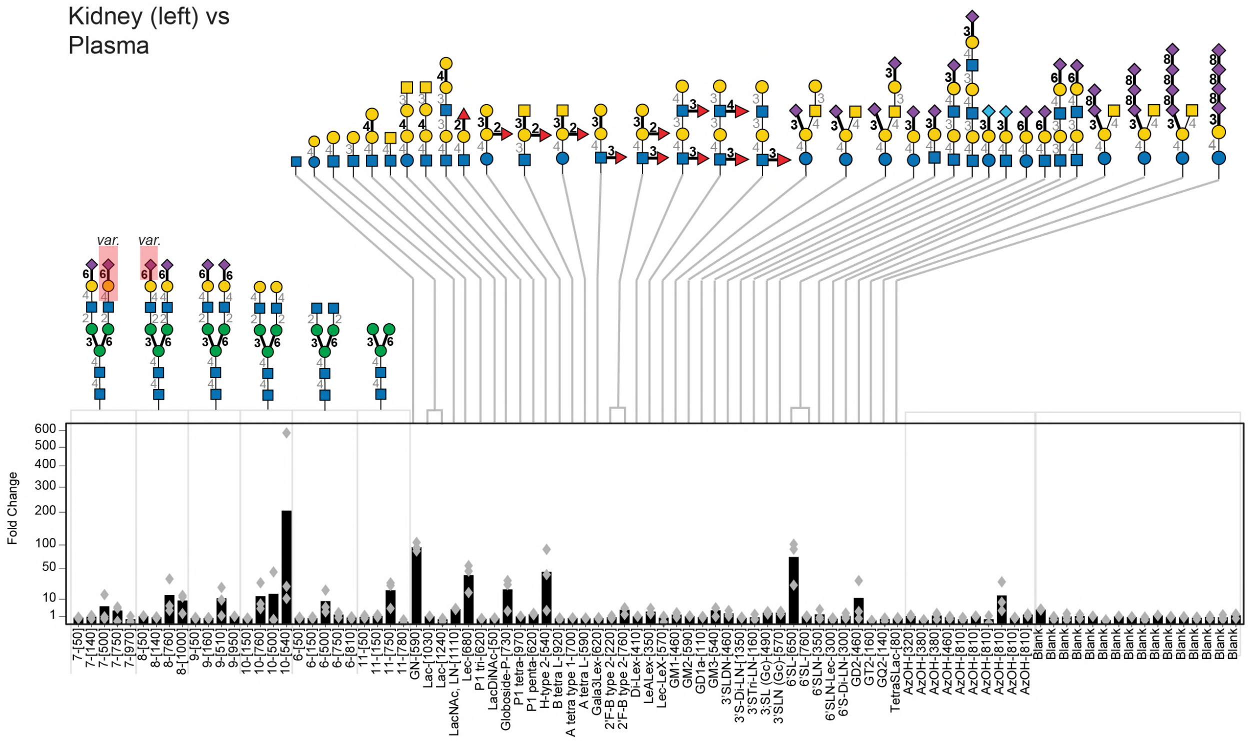

Supplementary Figure. 52: Summary of LiGA interaction with left kidney *in-vivo* described as fold change (FC) enrichment in left kidney with respect to plasma from the same animal.
The injection and processing of LiGA is described in Fig. 6 (main text); the *N*-glycan and blank (unmodified) phage data is the same as used to create Fig. 6b but analyzed separately for each mouse (n = 3). The grey diamonds show the fold change of each mouse and the mean of the three plotted as black bars.

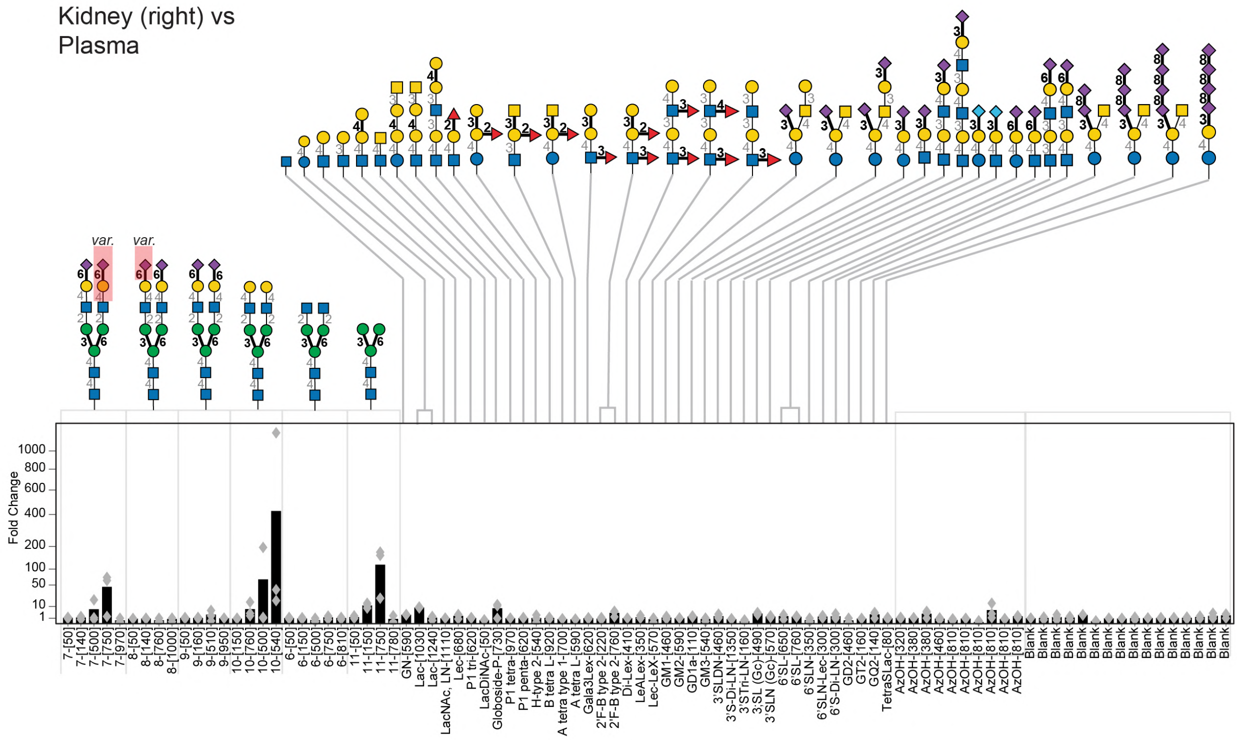

Supplementary Figure. 53: Summary of LiGA interaction with right kidney *in-vivo* described as fold change (FC) enrichment in right kidney with respect to plasma from the same animal.
The injection and processing of LiGA is described in Fig. 6 (main text); the *N*-glycan and blank (unmodified) phage data is the same as used to create Fig. 6b but analyzed separately for each mouse (n = 3). The grey diamonds show the fold change of each mouse and the mean of the three plotted as black bars.

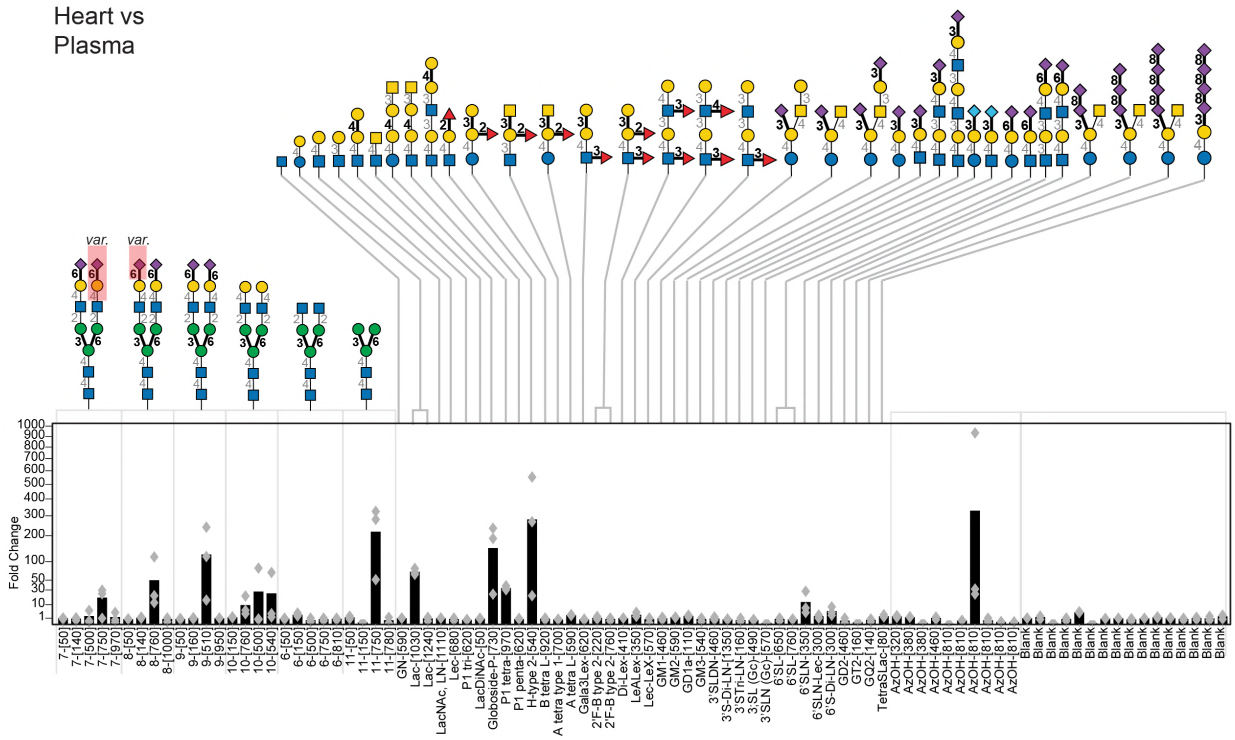

Supplementary Figure. 54: Summary of LiGA interaction with heart *in-vivo* described as fold change (FC) enrichment in heart with respect to plasma from the same animal.
The injection and processing of LiGA is described in Fig. 6 (main text); the *N*-glycan and blank (unmodified) phage data is the same as used to create Fig. 6b but analyzed separately for each mouse (n = 3). The grey diamonds show the fold change of each mouse and the mean of the three plotted as black bars.

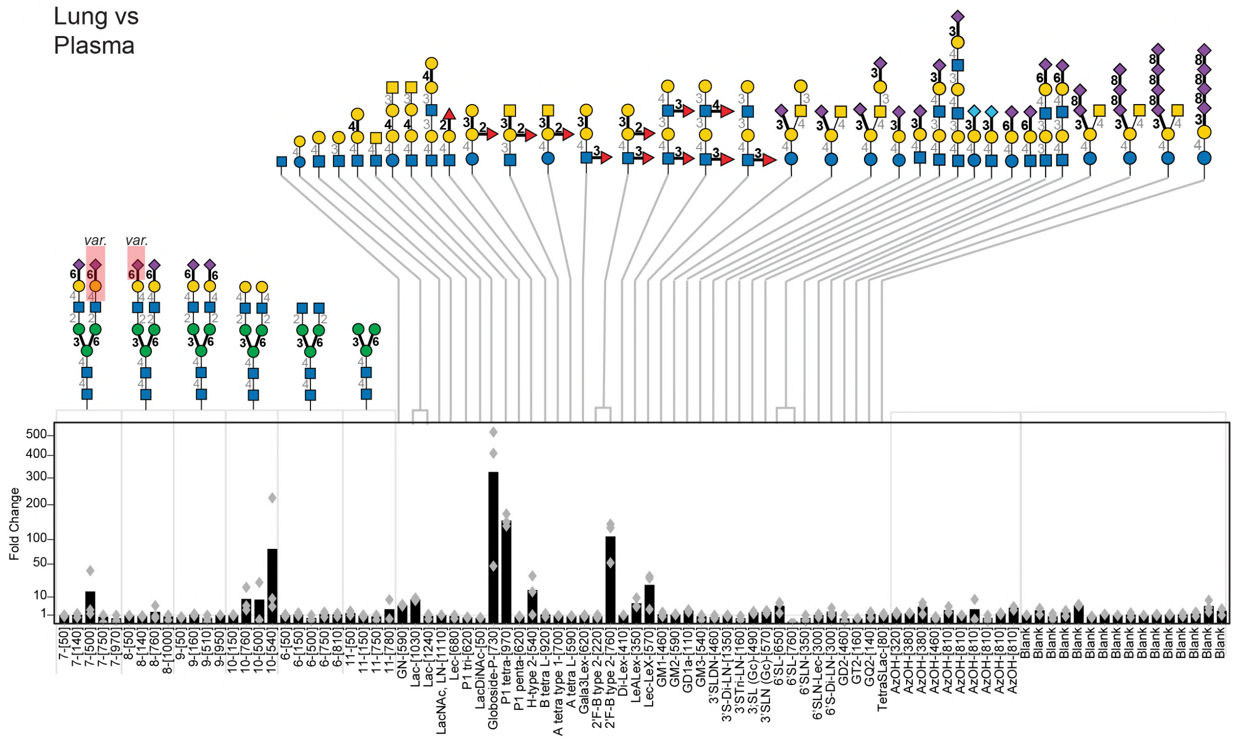

Supplementary Figure. 55: Summary of LiGA interaction with lungs *in-vivo* described as fold change (FC) enrichment in lungs with respect to plasma from the same animal.
The injection and processing of LiGA is described in Fig. 6 (main text); the *N*-glycan and blank (unmodified) phage data is the same as used to create Fig. 6b but analyzed separately for each mouse (n = 3). The grey diamonds show the fold change of each mouse and the mean of the three plotted as black bars.

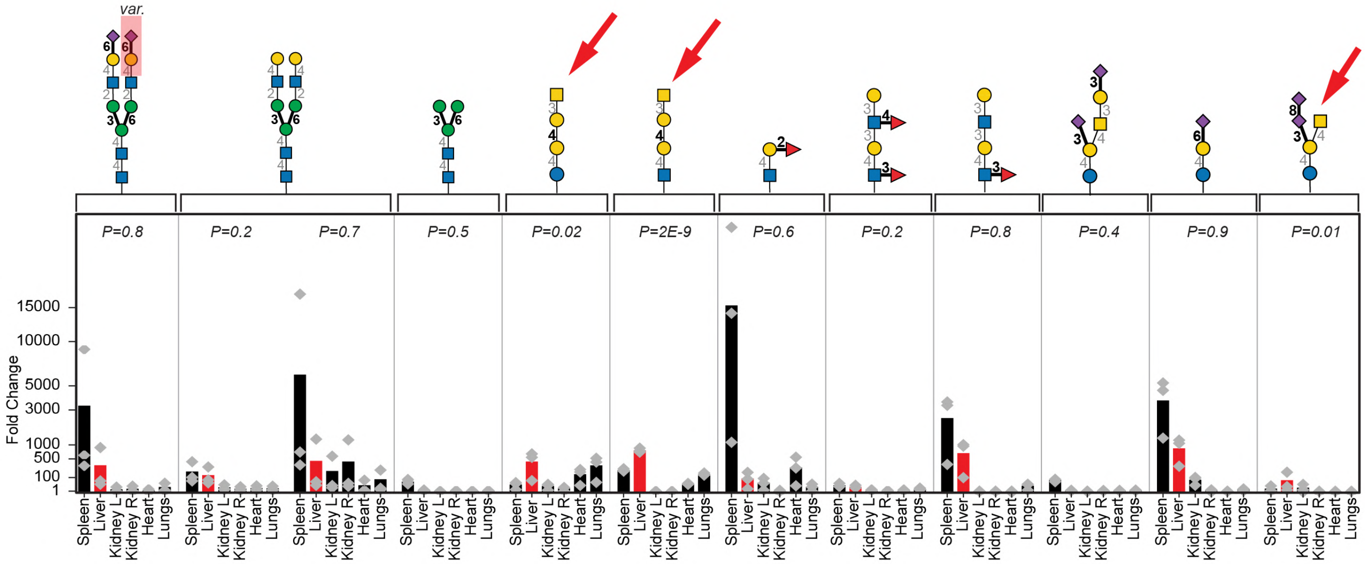

Supplementary Figure. 56: Summary of glycans enriched in liver compared to plasma. Among the glycans enriched in liver compared to plasma, we observed 11 glycans (12 glycan-density pairs) with FDR ≤ 0.05. The fold change for terminal GalNAc containing glycans (Globoside-P, P1-tetra and GD2) was highest and statistically significant compared to other organs. Thus highlighting that GalNAc in LiGA can be used for liver targeting, analogous to the many of the GalNAc-siRNA drug candidates in clinical trials[7]. For each of the glycan density-pairs in the plot, whether the fold change is significantly different in liver compared to the other tissues was assessed by a one-way ANOVA test (assumes normal distribution - implemented in R v. 3.5.2). This figure replots a subset of the data presented in Supplementary Fig. 50-55 and as in those figures the grey diamonds show the fold change of each mouse (n = 3) and the mean of the three is plotted as bars.

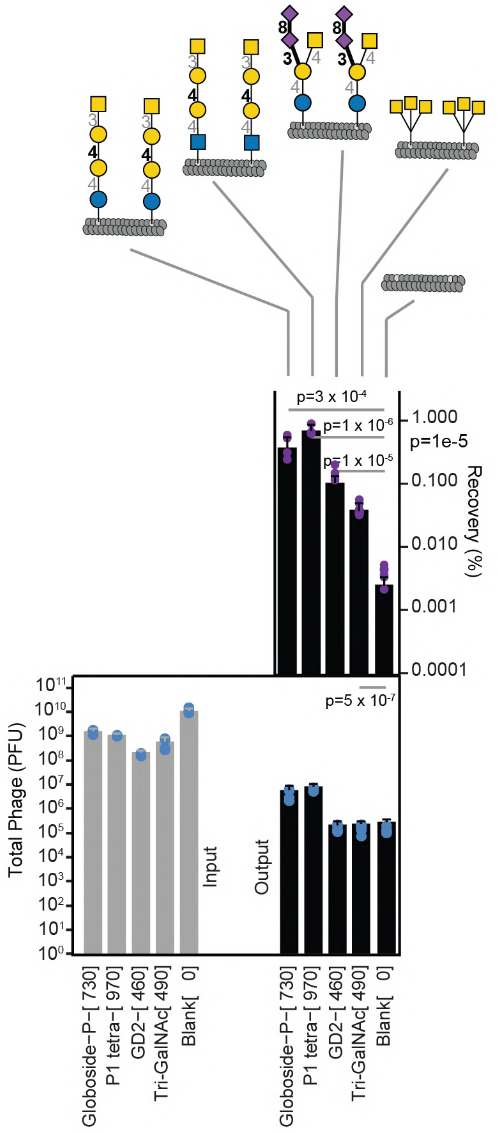

Supplementary Figure. 57: Summary of clonal binding of 6 glycans to HepG2 cells that expressed ASGPR.

Terminal GalNAc containing glycans were significantly enriched by ASPGR expressing HepG2 cells. Tri-GalNAc, a known ASGPR ligand was used as a positive control for binding. The GalNAc containing glycans were significantly enriched compared to blank phage (non-modified phages). Recovery of Globoside-P and P1-Tetra ~100 fold compared to blank phage suggesting specificity of GalNAc to ASGPR protein. The binding of GD2 and Tri-GalNAc was comparable based on the recovery of each clonal phages. n = 3 (Globoside-P, P-Tetra 1, GD2, Tri-GalNAc), 4 (Tri-Gal), 5 (Blank) independent biological replicates with *p*-values from two-tailed *t*-tests. Data is represented as mean+s.d, and the s.d for recovery is propagated from variance of input and output PFU.

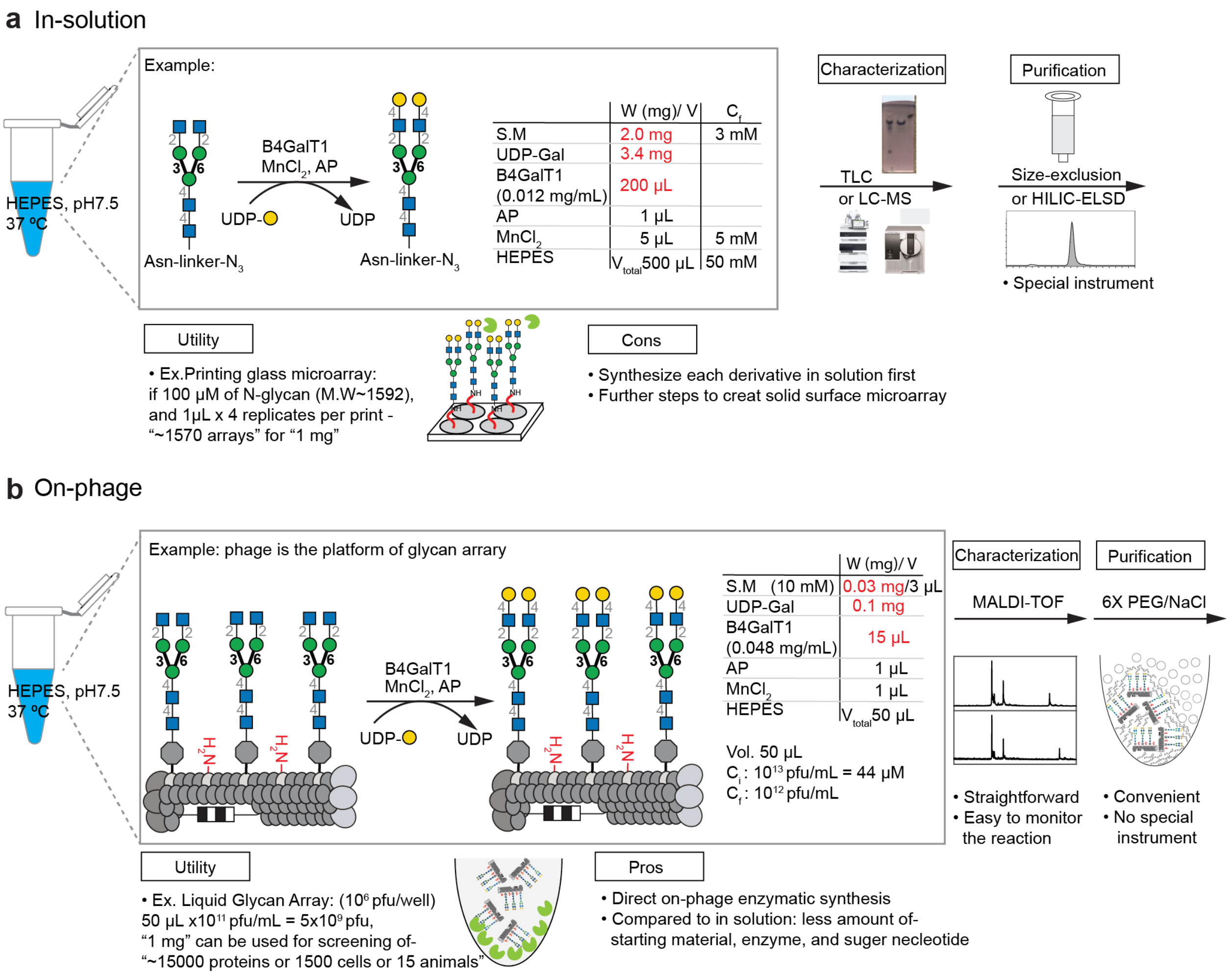

Supplementary Figure. 58: Comparison of "on-phage" enzymatic synthesis of liquid glycan array
(**a**) with in solution enzymatic synthesis of glycans used to construct glass-based glycan arrays (**b**). Both **a** and **b** have end point utility section which describes how many arrays can be generated from a fixed amount of each glycan (e.g., 1 mg of glycans can be used to print 1570 glass-based arrays). **b,** Example of on-phage enzymatic synthesis with detailed reaction conditions. The utility describes the amount of glycans needed to generate DNA-coded glycan needed to conduct a certain number of LiGA experiments (e.g., 1 mg of glycan yields enough LiGA for 15,000 protein binding experiments). While **a** and **b** are only an "order of magnitude"-type estimates, they illustrate that LiGA manufacturing requires an order of magnitude less material than the traditional glass-based glycan microarrays.

Supplementary References

1 Sun, B. *et al.* A simplified procedure for gram-scale production of sialylglycopeptide (SGP) from egg yolks and subsequent semi-synthesis of Man3GlcNAc oxazoline. *Carbohydr. Res.* **396**, 62-69 (2014).

2 Fraser, B. H. *et al.* Synthesis of 1, 4-triazole linked zanamivir dimers as highly potent inhibitors of influenza A and B. *Medchemcomm.* **4**, 383-386 (2013).

3 Sojitra, M. *et al.* Genetically encoded multivalent liquid glycan array displayed on M13 bacteriophage. *Nat. Chem. Biol.*, 1-11 (2021).

4 Bojar, D. *et al.* A useful guide to lectin binding: machine-learning directed annotation of 57 unique lectin specificities. *ACS Chem. Biol.* **17**, 2993-3012 (2022).

5 Gao, C. *et al.* Unique binding specificities of proteins toward isomeric asparagine-linked glycans. *Cell Chem. Biol.* **26**, 535-547. e534 (2019).

6 Ribeiro, J. P. *et al.* Characterization of a high-affinity sialic acid-specific CBM40 from Clostridium perfringens and engineering of a divalent form. *Biochem. J.* **473**, 2109-2118 (2016).

7 Springer, A. D. & Dowdy, S. F. GalNAc-siRNA Conjugates: Leading the Way for Delivery of RNAi Therapeutics. *Nucleic Acid Ther.* **28**, 109-118, doi:10.1089/nat.2018.0736 (2018).
