# Supplementary material for: Chemoenzymatic synthesis of genetically-encoded multivalent liquid N-glycan arrays
Source: Nat Commun. 2023 Aug 28;14:5237. doi: 10.1038/s41467-023-40900-y (PMC10462762; doi:10.1038/s41467-023-40900-y)
Supplement: Supplementary file 4 — Description of Additional Supplementary files [file 41467_2023_40900_MOESM4_ESM.pdf]

# Description of Additional Supplementary files

Supplementary Data 1: All MALDI Data.
Description: Two word documents that contain MALDI spectra for characterization of glycophage constructs used in Fig. 5 and 6, and a folder that contains this raw MALDI data as 70 separate text files.

Supplementary Data 2: Raw data for Fig.5.
Description: Sixteen excel documents that contain raw and normalized counts for next generation sequencing data used to generate bar charts and heatmap in Fig. 5.

Supplementary Data 3: Raw data for Fig.6.
Description: Seven excel documents that contain raw and normalized counts for next generation sequencing data used to generate bar charts and heatmap in Fig. 6.

Supplementary Data 4: LiGA Dictionaries.
Description: Two excel files, which describe 1 to 1 correspondence between glycan, density and DNA barcodes used in Fig. 5 and 6.

Supplementary Data 5: List of all glycans used in this paper.
Description: Information of all glycans used in this work to build LiGA components.

Supplementary Data 6: Illumina Sequence of Primers.
Description: Sequence of all primers used in this work are provided in one excel file.

Supplementary Data 7: ASGPR (HepG2) Clonal for Supplementary Fig. 57.
Description: Plaque count used to generate data for Supplementary Fig. 57.
