# Supplementary material for: Chemoenzymatic synthesis of genetically-encoded multivalent liquid N-glycan arrays
Source: Nat Commun. 2023 Aug 28;14:5237. doi: 10.1038/s41467-023-40900-y (PMC10462762; doi:10.1038/s41467-023-40900-y)
Supplement: Supplementary file 8 — Supplementary Dataset 4 [file 41467_2023_40900_MOESM8_ESM.zip › Supplementary Data 1_All MALDI Data/All MALDI Data for Fig.5.docx]

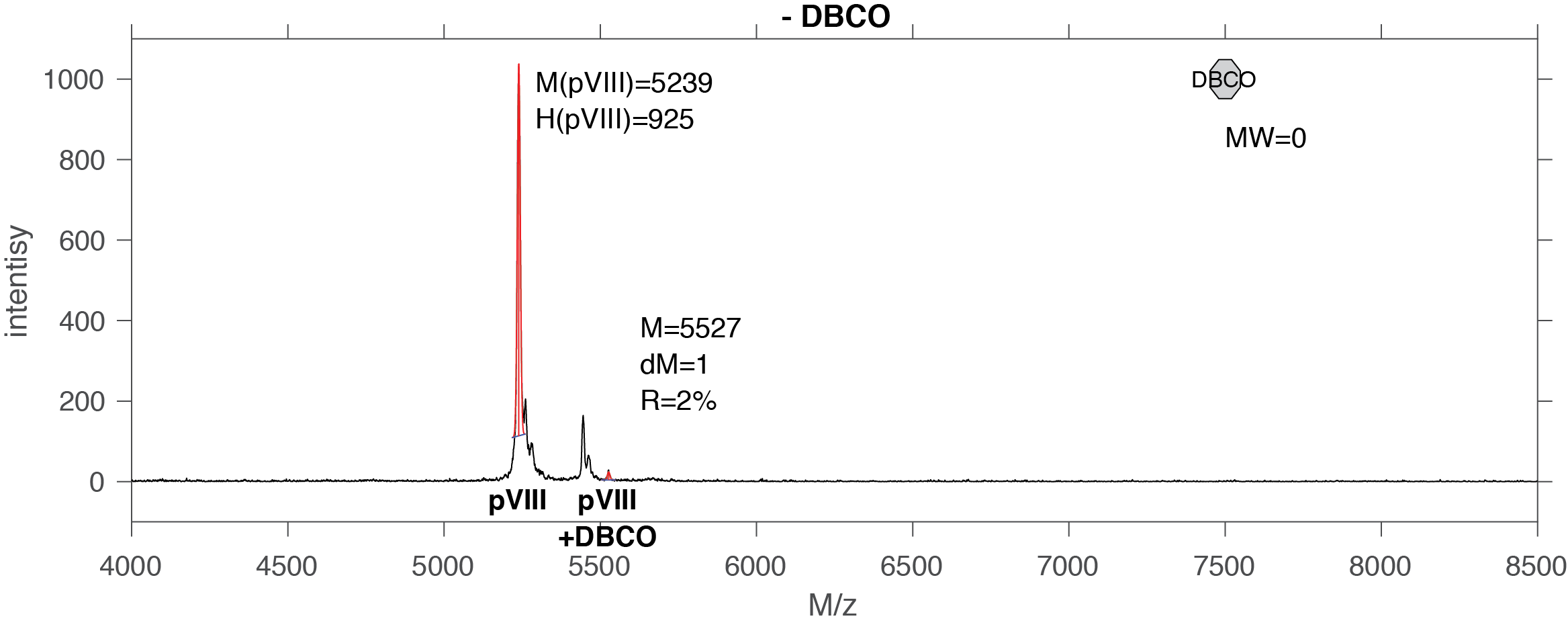


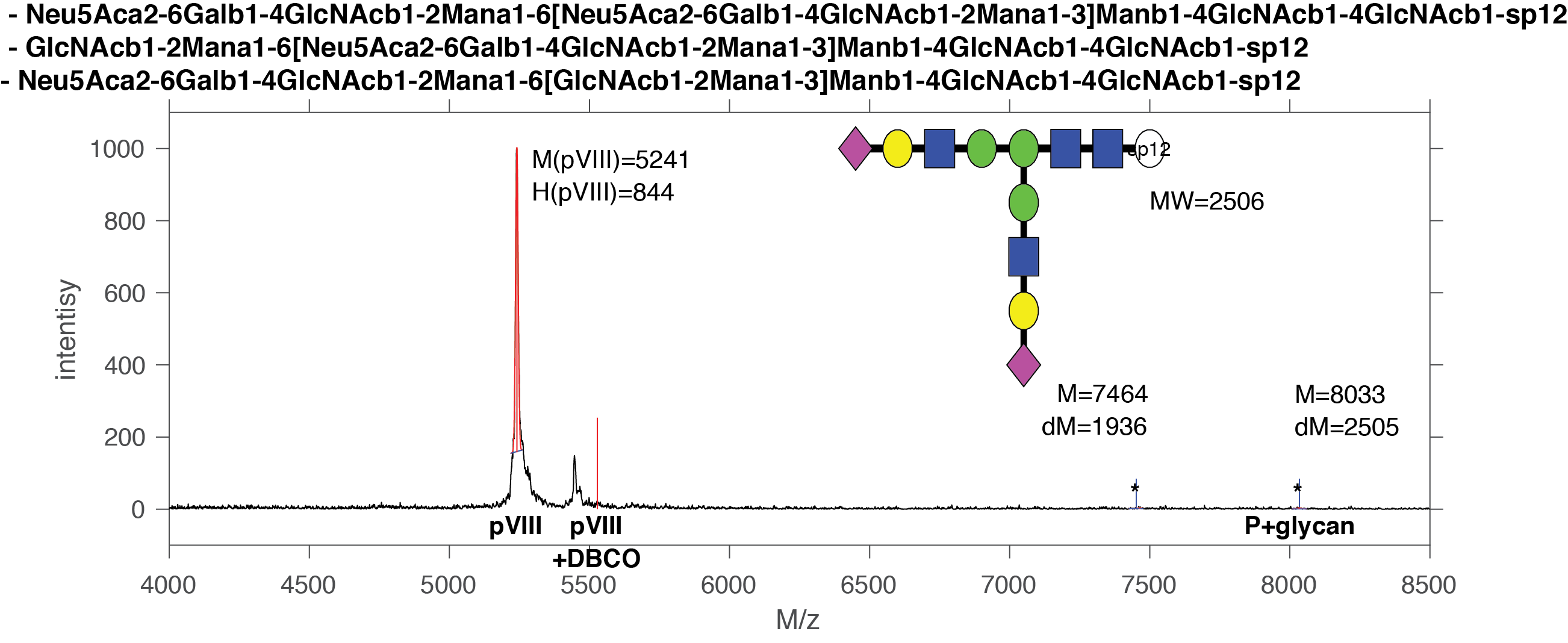


**SDB Number:** SDB103

**Barcode:** TTATTATTCGCAATTCCTTTAAGTGTTGAAAAAAACGACCAGAAGACTTATCACGCGGGTGGGGGG

**Axis Name:** 7-[50]

**IUPAC :** Neu5Ac(a2-6)Gal(b1-4)GlcNAc(b1-2)Man(a1-6)[Neu5Ac(a2-6)Gal(b1-4)GlcNAc(b1-2)Man(a1-3)]Man(b1-4)GlcNAc(b1-4)GlcNAc(b1-Sp;GlcNAc(b1-2)Man(a1-6)[Neu5Ac(a2-6)Gal(b1-4)GlcNAc(b1-2)Man(a1-3)]Man(b1-4)GlcNAc(b1-4)GlcNAc(b1-Sp;Neu5Ac(a2-6)Gal(b1-4)GlcNAc(b1-2)Man(a1-6)[GlcNAc(b1-2)Man(a1-3)]Man(b1-4)GlcNAc(b1-4)GlcNAc(b1-Sp

**Maldi File:** TL-IV-65-DBCO-SDB103c_0004.txt and TL-IV-71-0607_0006.txt

**Density:** based on DBCO intensity was 2%


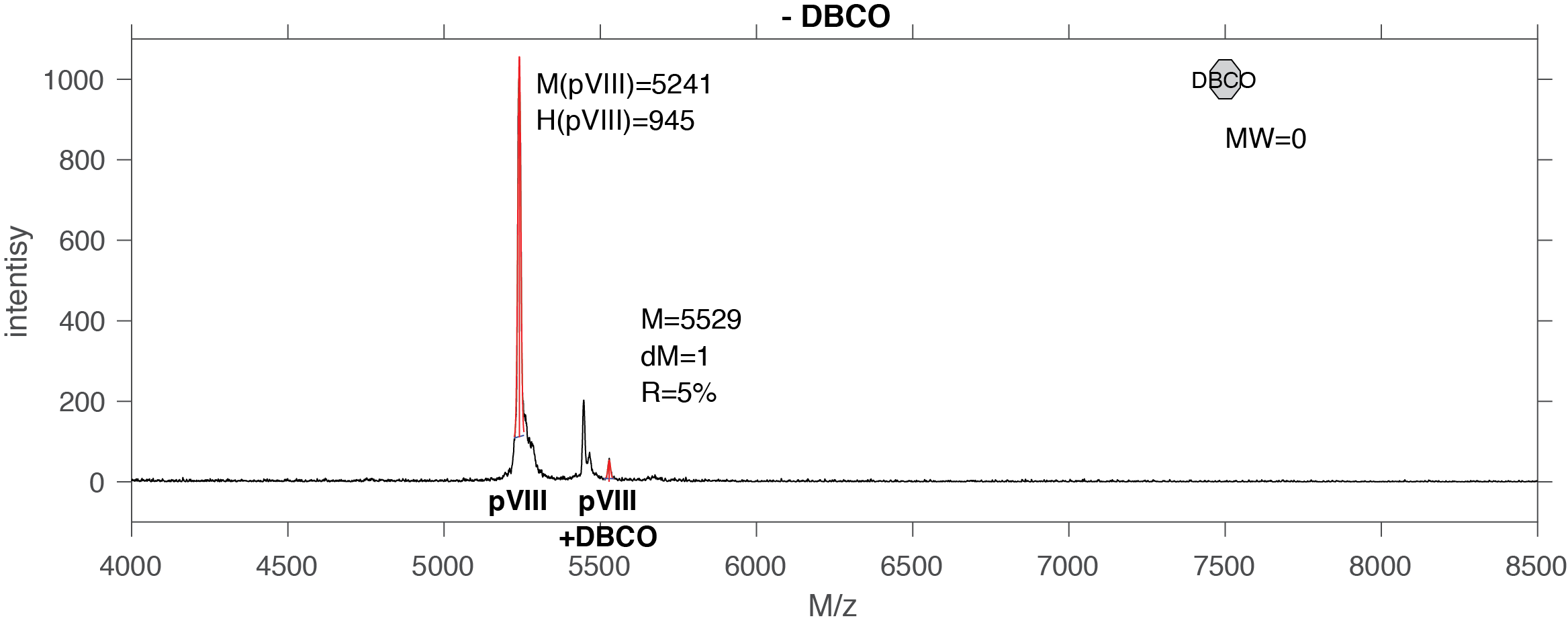


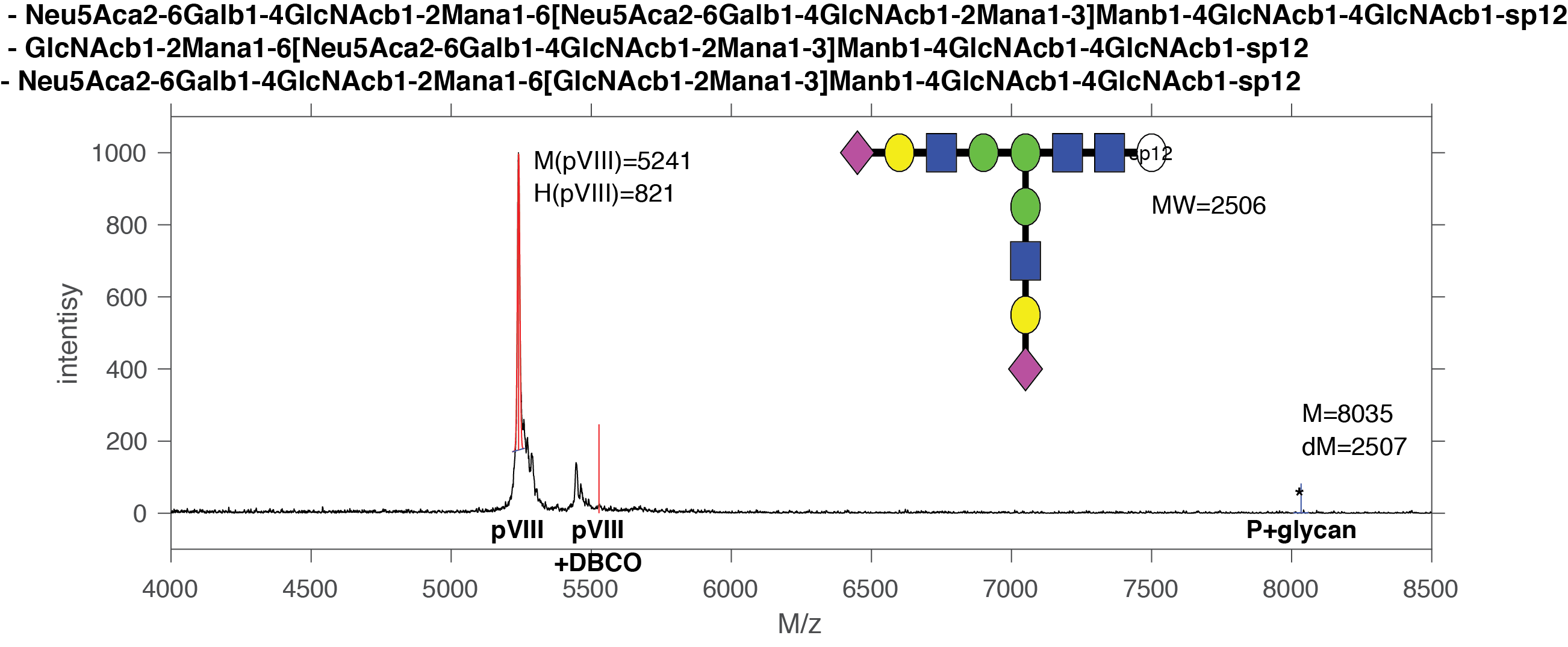


**SDB Number:** SDB191

**Barcode:** TTATTATTCGCAATTCCTTTAAGCGTAGAGAAGAATGATCAAAAAACATATCACGCTGGTGGTGGT

**Axis Name:** 7-[140]

**IUPAC :** Neu5Ac(a2-6)Gal(b1-4)GlcNAc(b1-2)Man(a1-6)[Neu5Ac(a2-6)Gal(b1-4)GlcNAc(b1-2)Man(a1-3)]Man(b1-4)GlcNAc(b1-4)GlcNAc(b1-Sp;GlcNAc(b1-2)Man(a1-6)[Neu5Ac(a2-6)Gal(b1-4)GlcNAc(b1-2)Man(a1-3)]Man(b1-4)GlcNAc(b1-4)GlcNAc(b1-Sp;Neu5Ac(a2-6)Gal(b1-4)GlcNAc(b1-2)Man(a1-6)[GlcNAc(b1-2)Man(a1-3)]Man(b1-4)GlcNAc(b1-4)GlcNAc(b1-Sp

**Maldi File:** TL-IV-59-SDB191-8min_0002.txt and TL-IV-61-0607_0003.txt

**Density:** based on DBCO intensity was 5%


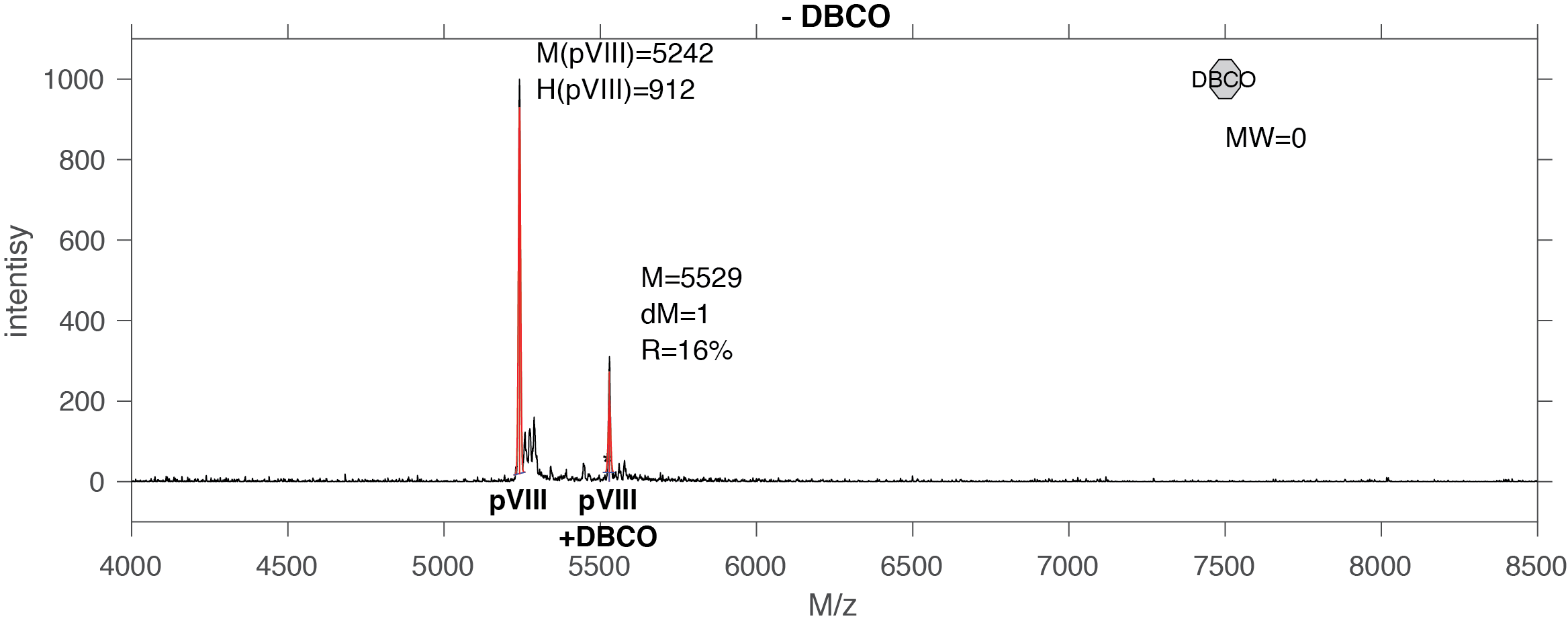


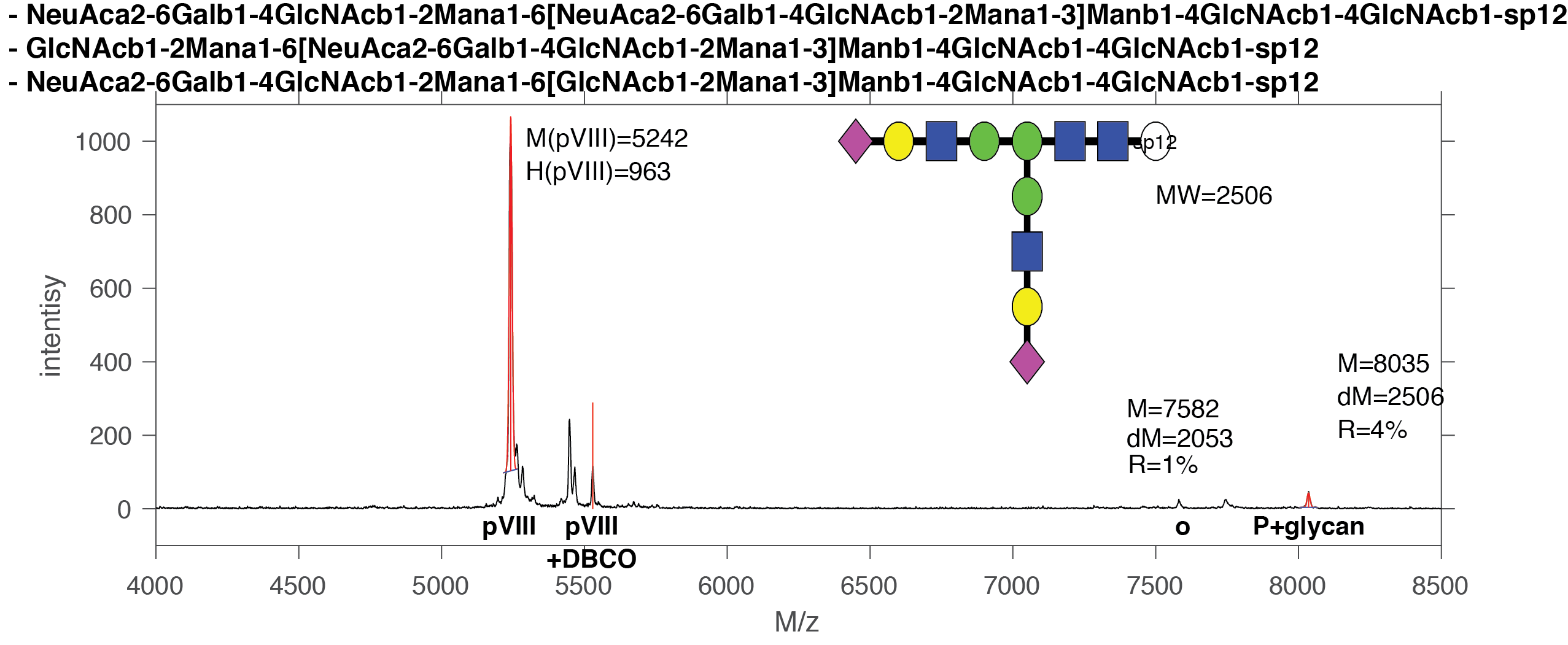


**SDB Number:** SDB120

**Barcode:** CTGCTGTTTGCTATTCCACTAAGTGTTGAAAAAAACGATCAGAAGACTTATCATGCTGGGGGAGGA

**Axis Name:** 7-[500]

**IUPAC :** Neu5Ac(a2-6)Gal(b1-4)GlcNAc(b1-2)Man(a1-6)[Neu5Ac(a2-6)Gal(b1-4)GlcNAc(b1-2)Man(a1-3)]Man(b1-4)GlcNAc(b1-4)GlcNAc(b1-Sp;GlcNAc(b1-2)Man(a1-6)[Neu5Ac(a2-6)Gal(b1-4)GlcNAc(b1-2)Man(a1-3)]Man(b1-4)GlcNAc(b1-4)GlcNAc(b1-Sp;Neu5Ac(a2-6)Gal(b1-4)GlcNAc(b1-2)Man(a1-6)[GlcNAc(b1-2)Man(a1-3)]Man(b1-4)GlcNAc(b1-4)GlcNAc(b1-Sp

**Maldi File:** TL-III-111-DBCO-120_0003.txt and TL-III-111-2nd day_0005.txt

**Density:** based on DBCO density was 16%


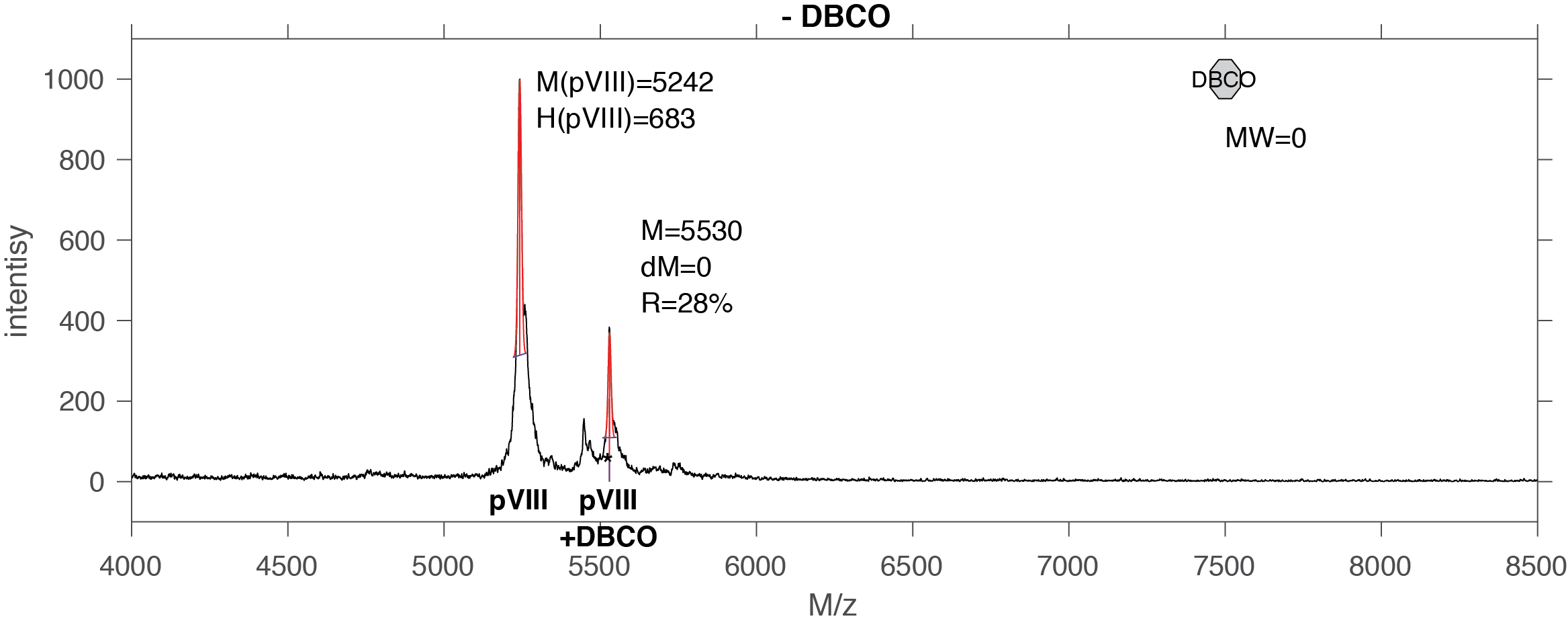


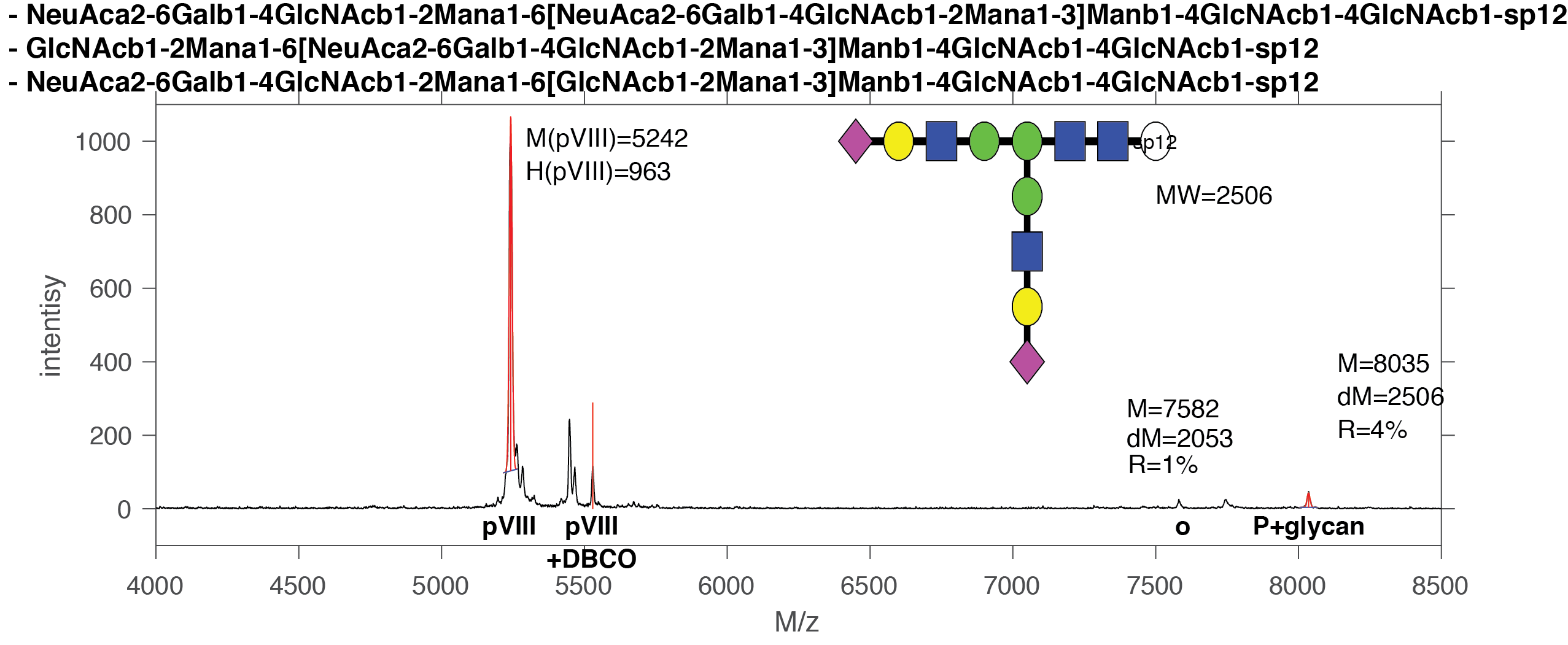


**SDB Number:** SDB28

**Barcode:** CTACTCTTCGCCATTCCACTGAGTGTGGAGAAGAATGATCAGAAGACTTATCATGCGGGTGGAGGT

**Axis Name:** 7-[750]

**IUPAC :** Neu5Ac(a2-6)Gal(b1-4)GlcNAc(b1-2)Man(a1-6)[Neu5Ac(a2-6)Gal(b1-4)GlcNAc(b1-2)Man(a1-3)]Man(b1-4)GlcNAc(b1-4)GlcNAc(b1-Sp;GlcNAc(b1-2)Man(a1-6)[Neu5Ac(a2-6)Gal(b1-4)GlcNAc(b1-2)Man(a1-3)]Man(b1-4)GlcNAc(b1-4)GlcNAc(b1-Sp;Neu5Ac(a2-6)Gal(b1-4)GlcNAc(b1-2)Man(a1-6)[GlcNAc(b1-2)Man(a1-3)]Man(b1-4)GlcNAc(b1-4)GlcNAc(b1-Sp

**Maldi File:** TL-IV-43-DBCO-SDB28_0001.txt and TL-IV-45_0531_0006.txt

**Density:** 4%, based on DBCO intensity was 28%

**
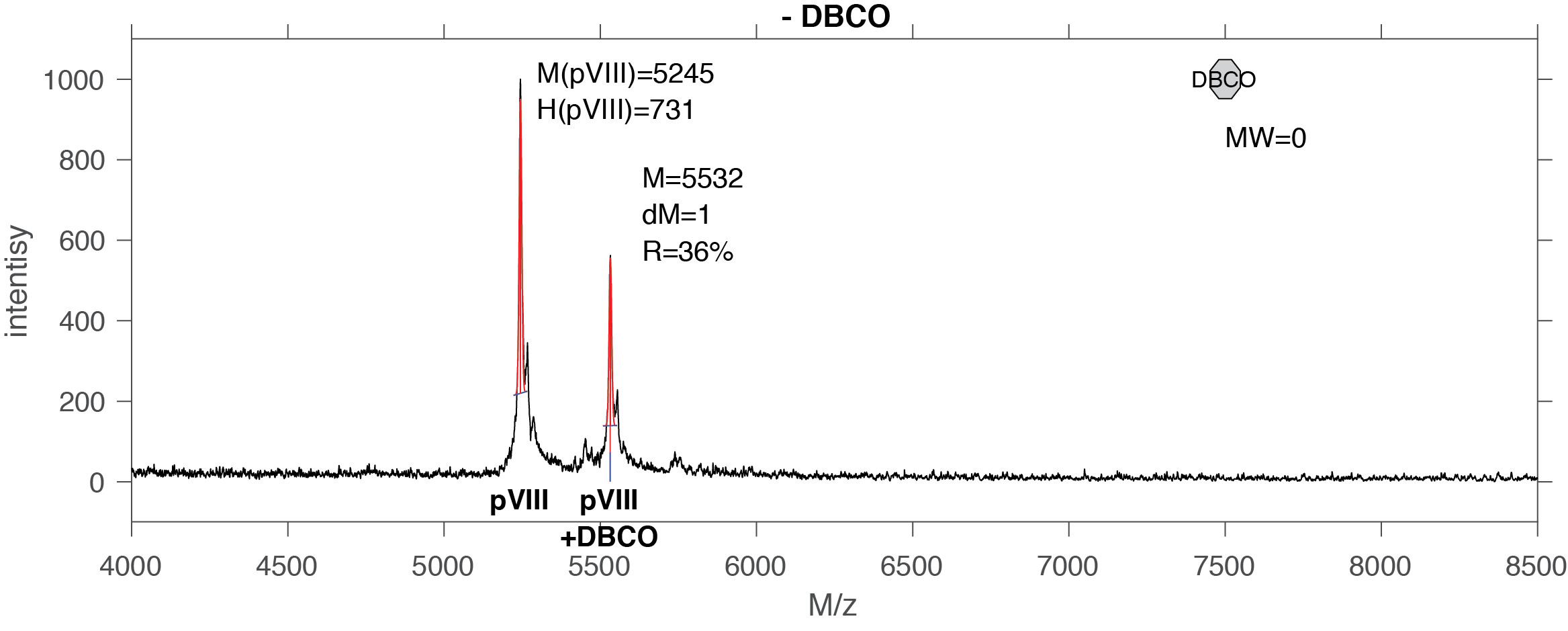
**

**
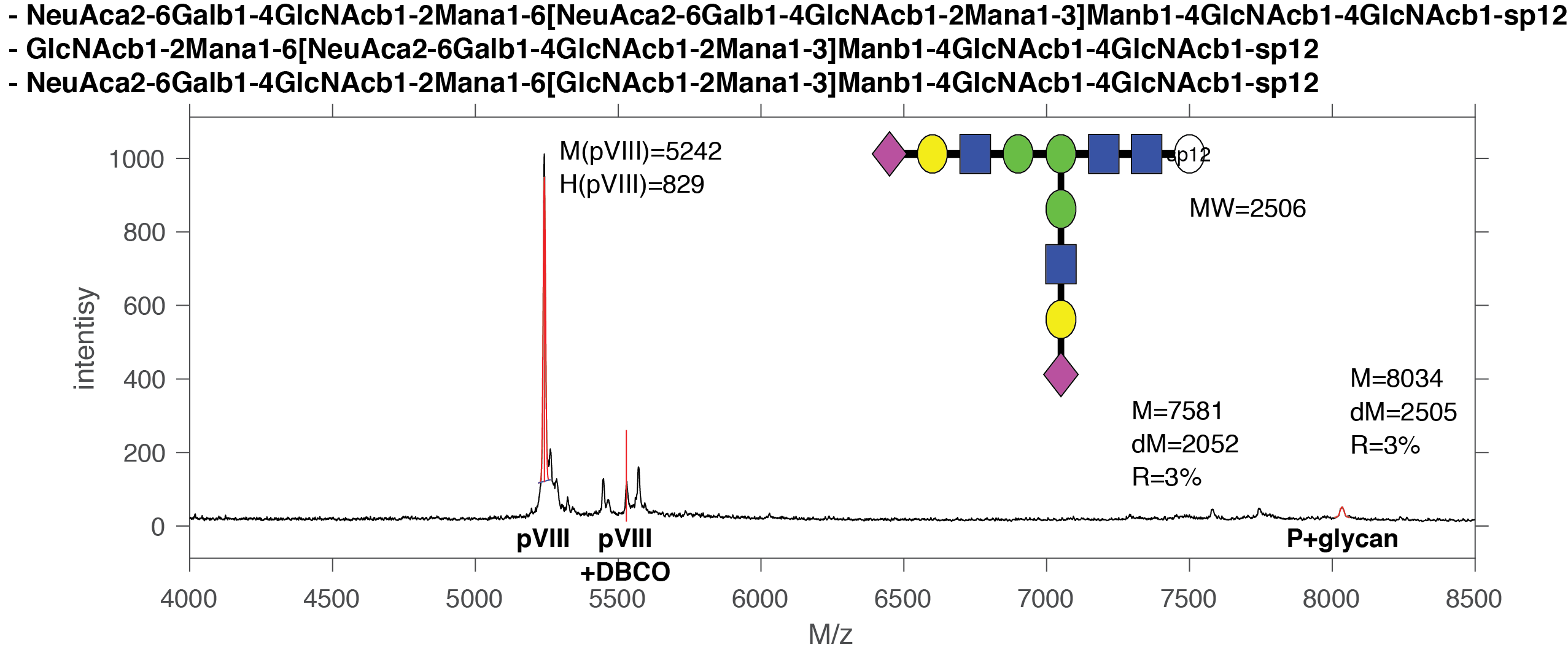
**

**SDB Number:** SDB186

**Barcode:** CTACTGTTCGCAATCCCGCTAAGTGTTGAGAAAAACGATCAAAAGACTTATCATGCAGGCGGAGGA

**Axis Name:** 7-[970]

**IUPAC :** Neu5Ac(a2-6)Gal(b1-4)GlcNAc(b1-2)Man(a1-6)[Neu5Ac(a2-6)Gal(b1-4)GlcNAc(b1-2)Man(a1-3)]Man(b1-4)GlcNAc(b1-4)GlcNAc(b1-Sp;GlcNAc(b1-2)Man(a1-6)[Neu5Ac(a2-6)Gal(b1-4)GlcNAc(b1-2)Man(a1-3)]Man(b1-4)GlcNAc(b1-4)GlcNAc(b1-Sp;Neu5Ac(a2-6)Gal(b1-4)GlcNAc(b1-2)Man(a1-6)[GlcNAc(b1-2)Man(a1-3)]Man(b1-4)GlcNAc(b1-4)GlcNAc(b1-Sp

**Maldi File:** TL-IV-41-DBCO-SDB191-60min-2nd_0001.txt and TL-IV-47_0531_0004.txt

**Density:** based on DBCO intensity was 36%


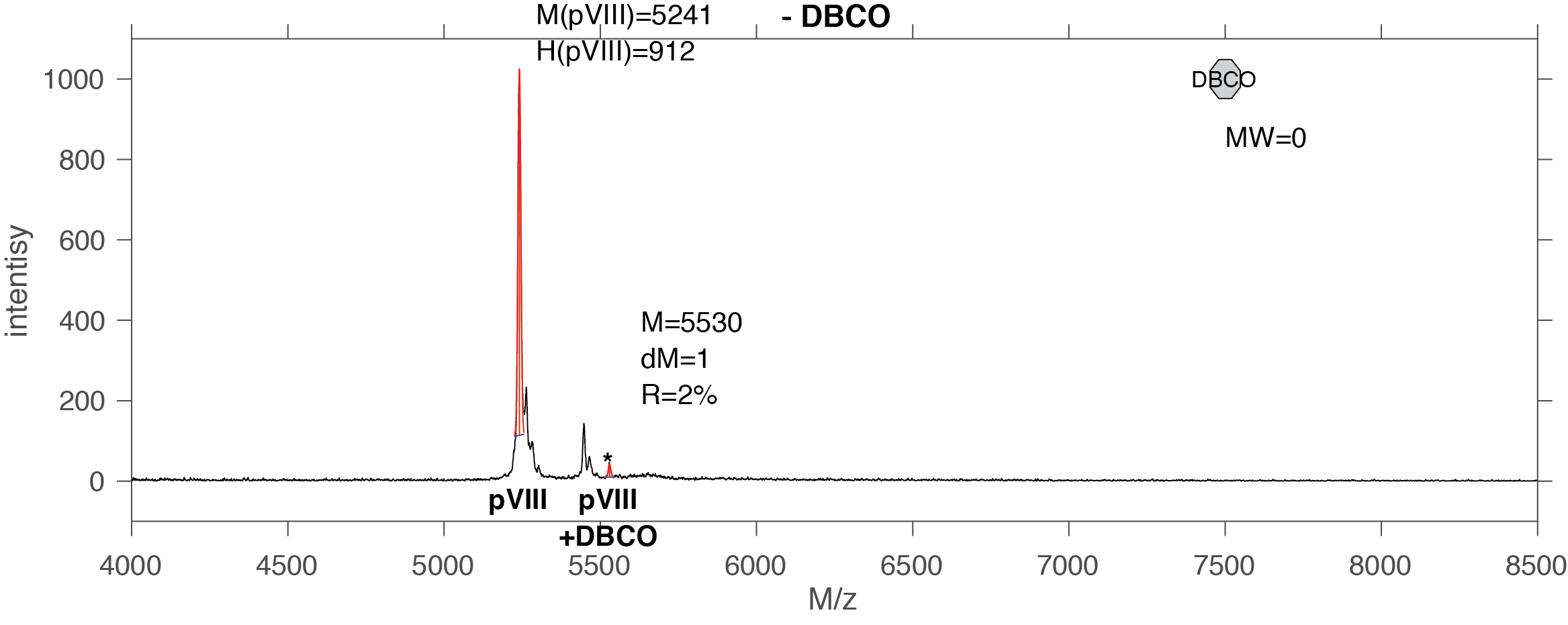


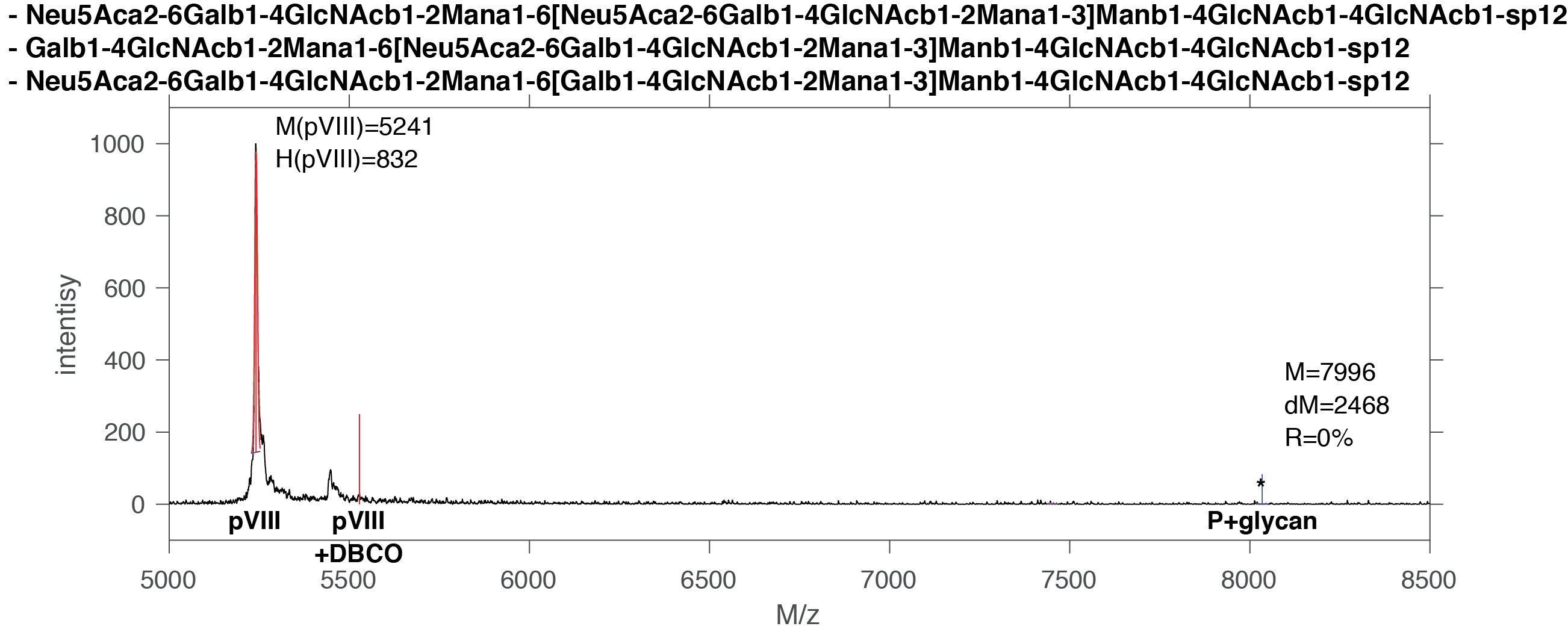


**SDB Number:** SDB206

**Barcode:** CTTCTATTTGCTATTCCTCTAAGTGTTGAAAAAAACGACCAAAAAACATATCATGCGGGGGGCGGA

**Axis Name:** 8-[50]

**IUPAC :** Neu5Ac(a2-6)Gal(b1-4)GlcNAc(b1-2)Man(a1-6)[Neu5Ac(a2-6)Gal(b1-4)GlcNAc(b1-2)Man(a1-3)]Man(b1-4)GlcNAc(b1-4)GlcNAc(b1-Sp; Gal(b1-4)GlcNAc(b1-2)Man(a1-6)[Neu5Ac(a2-6)Gal(b1-4)GlcNAc(b1-2)Man(a1-3)]Man(b1-4)GlcNAc(b1-4)GlcNAc(b1-Sp; Neu5Ac(a2-6)Gal(b1-4)GlcNAc(b1-2)Man(a1-6)[Gal(b1-4)GlcNAc(b1-2)Man(a1-3)]Man(b1-4)GlcNAc(b1-4)GlcNAc(b1-Sp

**Maldi File:** TL-IV-93-DBCO-SDB206_0008.txt and TL-IV-93_0002.txt

**Density:** based on DBCO density was 2%


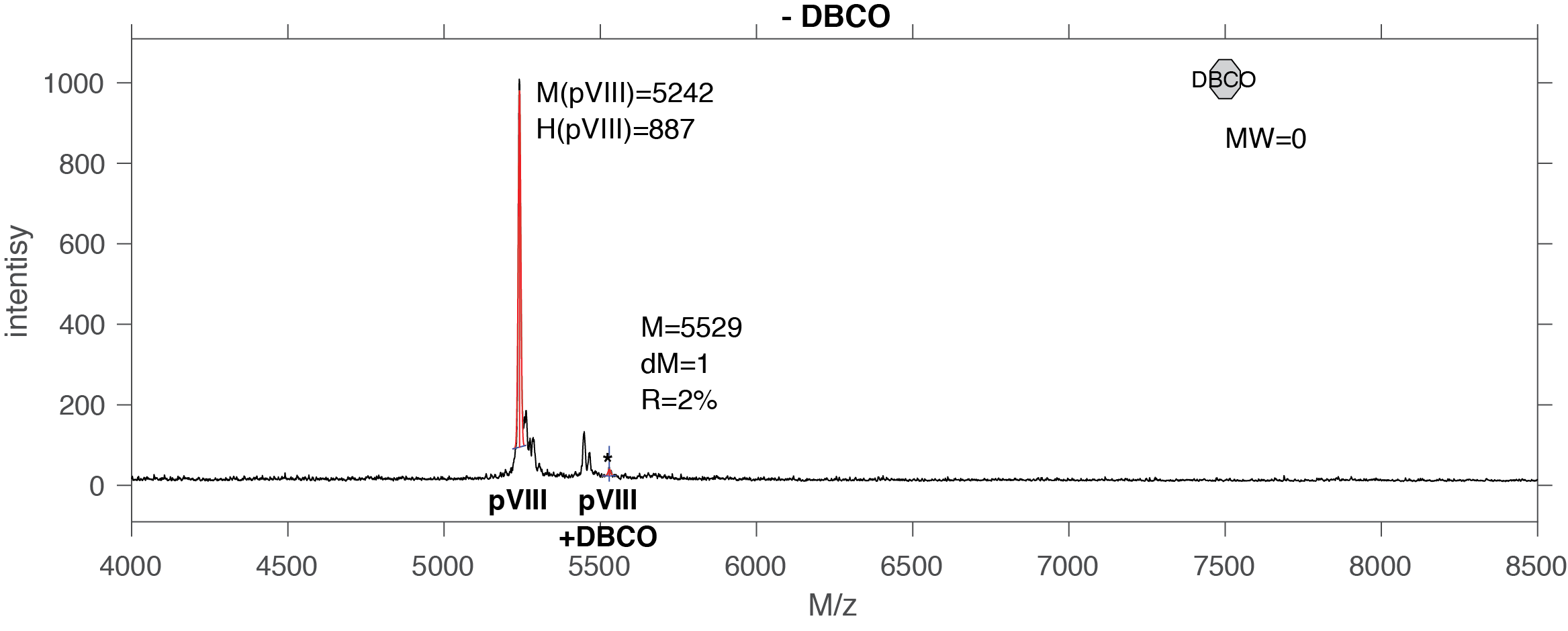


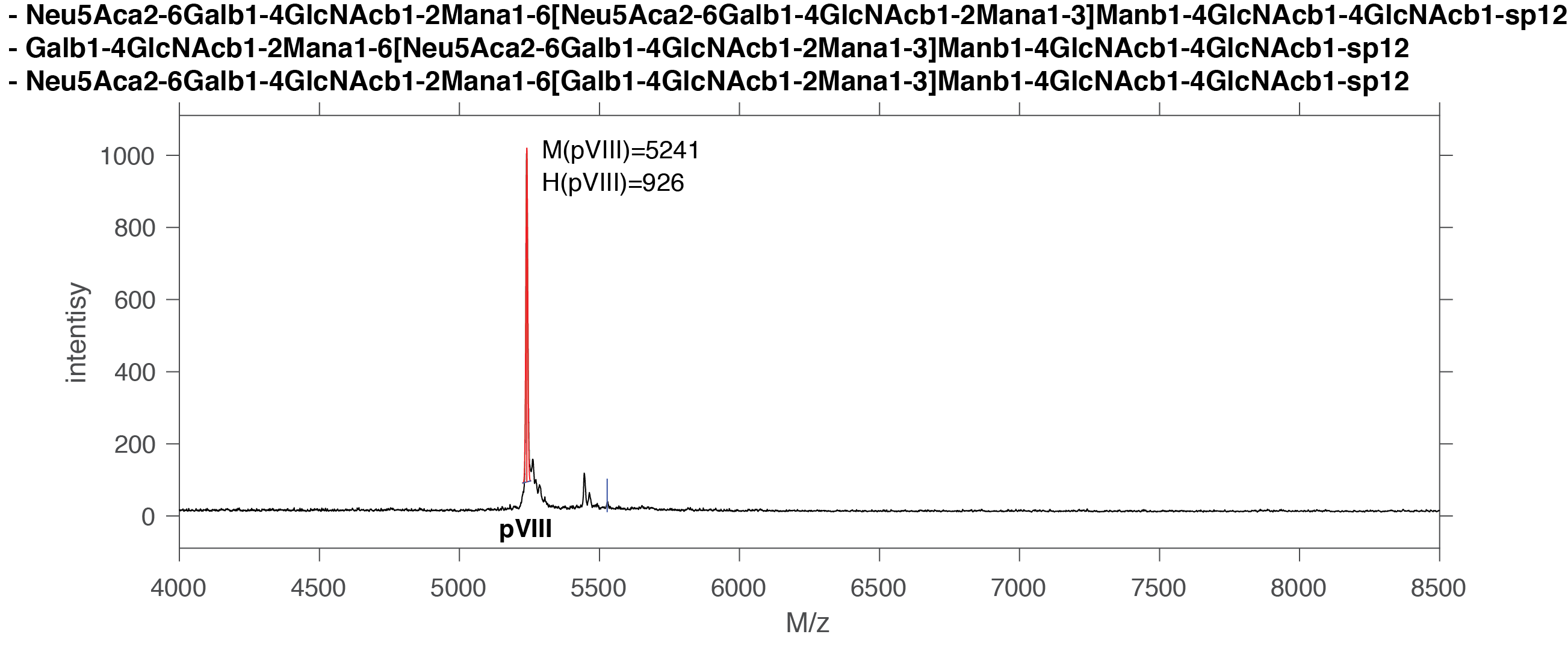


**SDB Number:** SDB36

**Barcode:** CTGCTCTTCGCGATCCCTCTGAGTGTGGAGAAGAATGATCAGAAGACTTATCATGCGGGTGGAGGT

**Axis Name:** 8-[50]

**IUPAC :** Neu5Ac(a2-6)Gal(b1-4)GlcNAc(b1-2)Man(a1-6)[Neu5Ac(a2-6)Gal(b1-4)GlcNAc(b1-2)Man(a1-3)]Man(b1-4)GlcNAc(b1-4)GlcNAc(b1-Sp; Gal(b1-4)GlcNAc(b1-2)Man(a1-6)[Neu5Ac(a2-6)Gal(b1-4)GlcNAc(b1-2)Man(a1-3)]Man(b1-4)GlcNAc(b1-4)GlcNAc(b1-Sp; Neu5Ac(a2-6)Gal(b1-4)GlcNAc(b1-2)Man(a1-6)[Gal(b1-4)GlcNAc(b1-2)Man(a1-3)]Man(b1-4)GlcNAc(b1-4)GlcNAc(b1-Sp

**Maldi File:**. TL-IV-115-DBCO-SDB36_0004.txt and TL-IV-115_0001.txt

**Density:** based on DBCO density was 2%

**
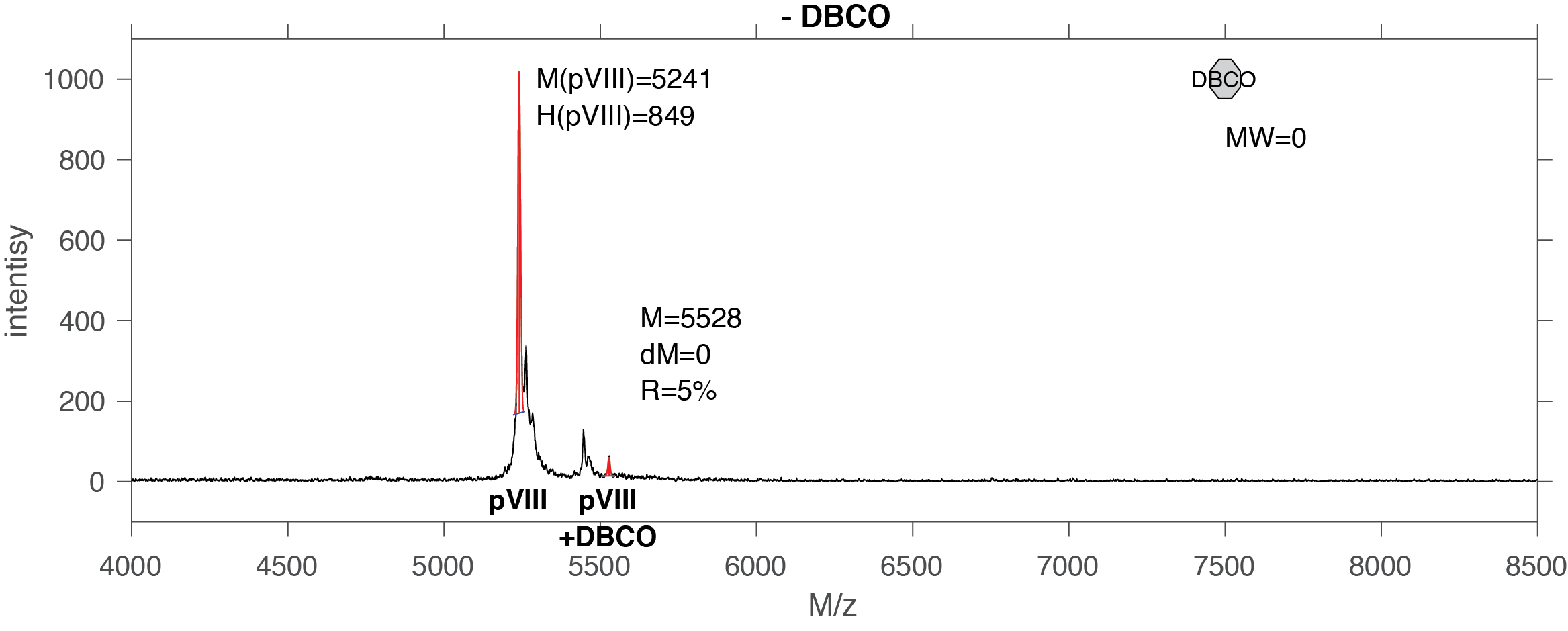
**


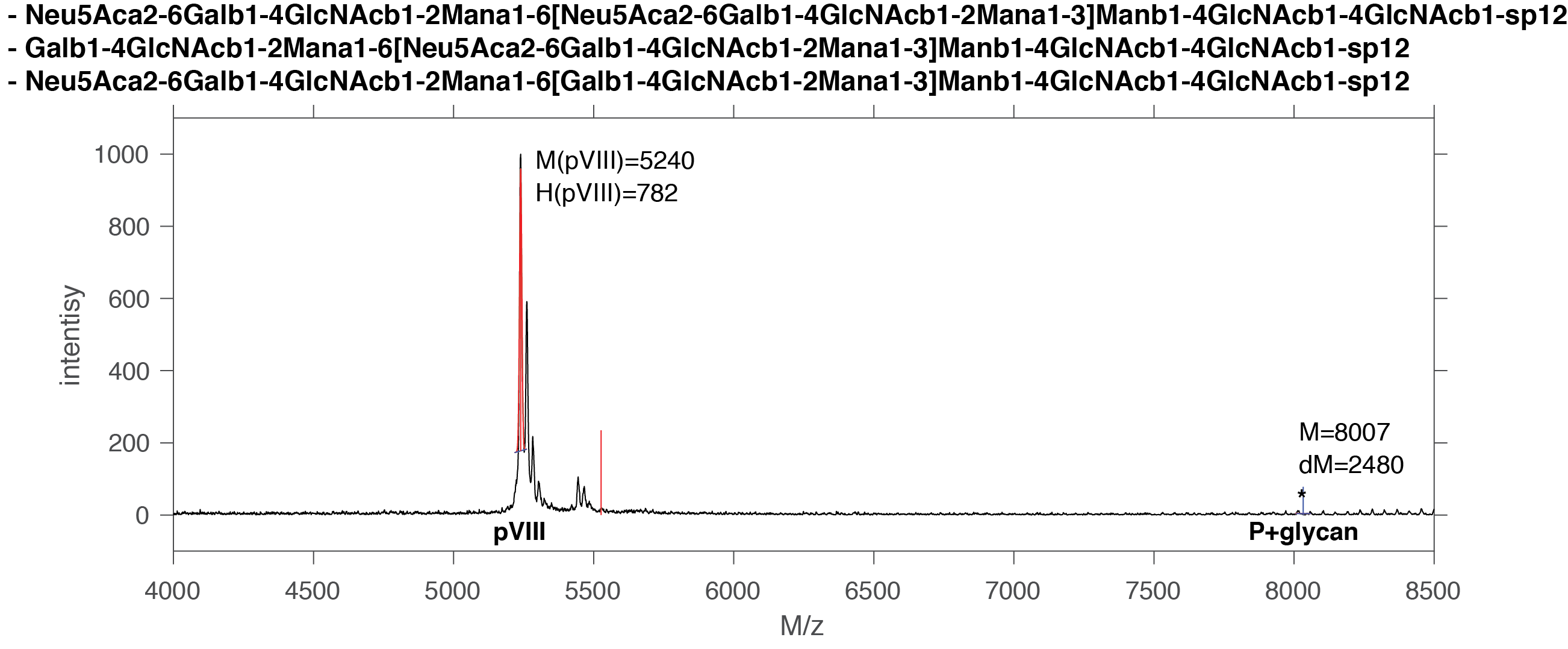


**SDB Number:** SDB163

**Barcode:** CTGCTGTTTGCGATACCCCTGAGCGTGGAGAAAAATGACCAAAAGACTTACCATGCTGGAGGAGGA

**Axis Name:** 8-[140]

**IUPAC :** Neu5Ac(a2-6)Gal(b1-4)GlcNAc(b1-2)Man(a1-6)[Neu5Ac(a2-6)Gal(b1-4)GlcNAc(b1-2)Man(a1-3)]Man(b1-4)GlcNAc(b1-4)GlcNAc(b1-Sp; Gal(b1-4)GlcNAc(b1-2)Man(a1-6)[Neu5Ac(a2-6)Gal(b1-4)GlcNAc(b1-2)Man(a1-3)]Man(b1-4)GlcNAc(b1-4)GlcNAc(b1-Sp; Neu5Ac(a2-6)Gal(b1-4)GlcNAc(b1-2)Man(a1-6)[Gal(b1-4)GlcNAc(b1-2)Man(a1-3)]Man(b1-4)GlcNAc(b1-4)GlcNAc(b1-Sp

**Maldi File:** TL-IV-59-SDB163-8min_0011.txt and TL-IV-75-0611_0001.txt

**Density:** based on DBCO density was 5%

**
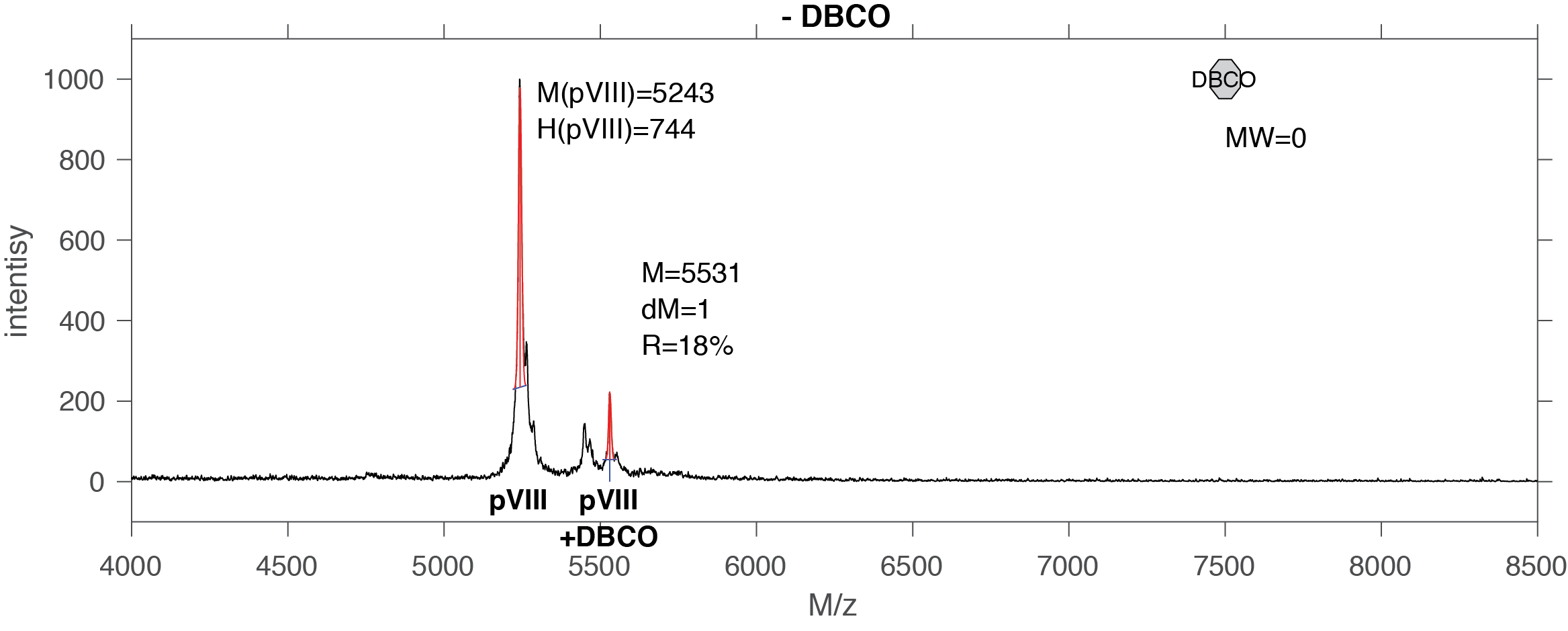
**


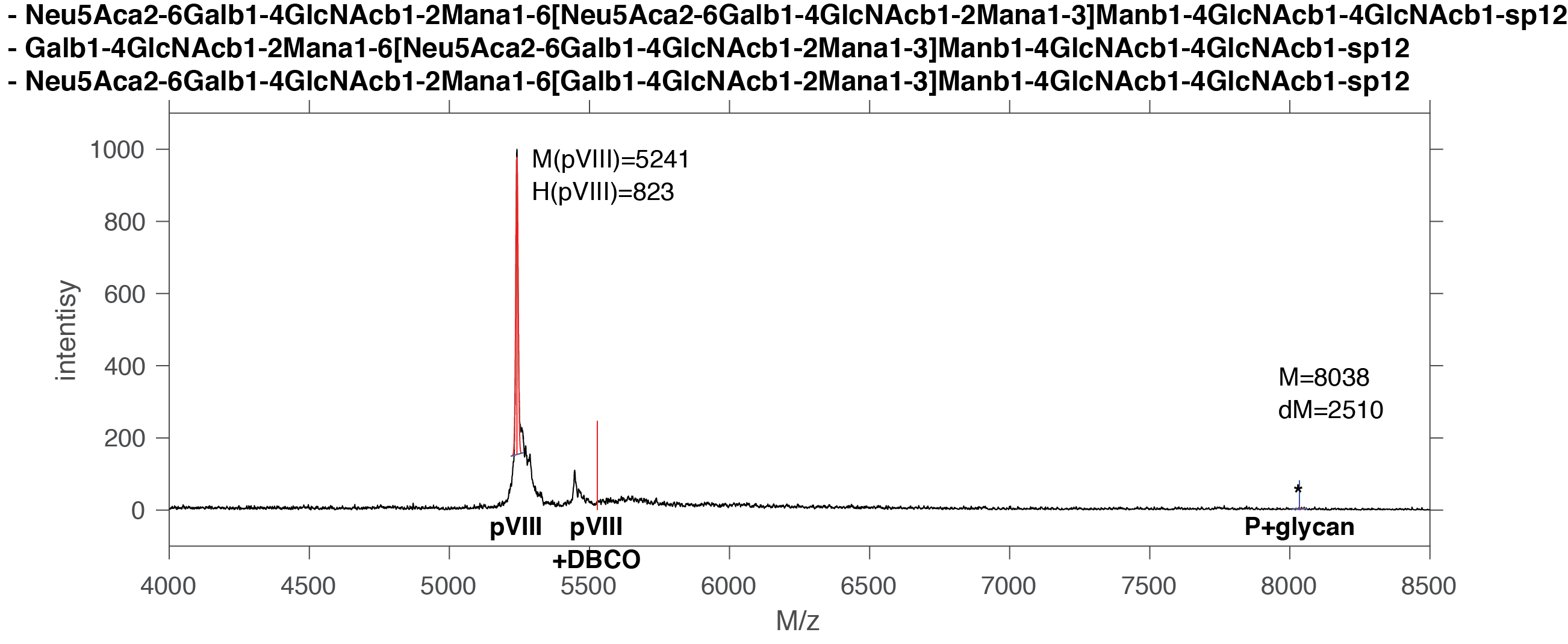


**SDB Number:** SDB170

**Barcode:** TTATTATTCGCAATTCCTTTAAGCGTGGAGAAAAATGACCAAAAAACGTATCATGCGGGGGGGGGT

**Axis Name:** 8-[490]

**IUPAC :** Neu5Ac(a2-6)Gal(b1-4)GlcNAc(b1-2)Man(a1-6)[Neu5Ac(a2-6)Gal(b1-4)GlcNAc(b1-2)Man(a1-3)]Man(b1-4)GlcNAc(b1-4)GlcNAc(b1-Sp; Gal(b1-4)GlcNAc(b1-2)Man(a1-6)[Neu5Ac(a2-6)Gal(b1-4)GlcNAc(b1-2)Man(a1-3)]Man(b1-4)GlcNAc(b1-4)GlcNAc(b1-Sp; Neu5Ac(a2-6)Gal(b1-4)GlcNAc(b1-2)Man(a1-6)[Gal(b1-4)GlcNAc(b1-2)Man(a1-3)]Man(b1-4)GlcNAc(b1-4)GlcNAc(b1-Sp

**Maldi File:** TL-IV-51-DBCO-SDB170_0006.txt and TL-IV-67-0610_0004.txt

**Density:** based on DBCO density was 18%


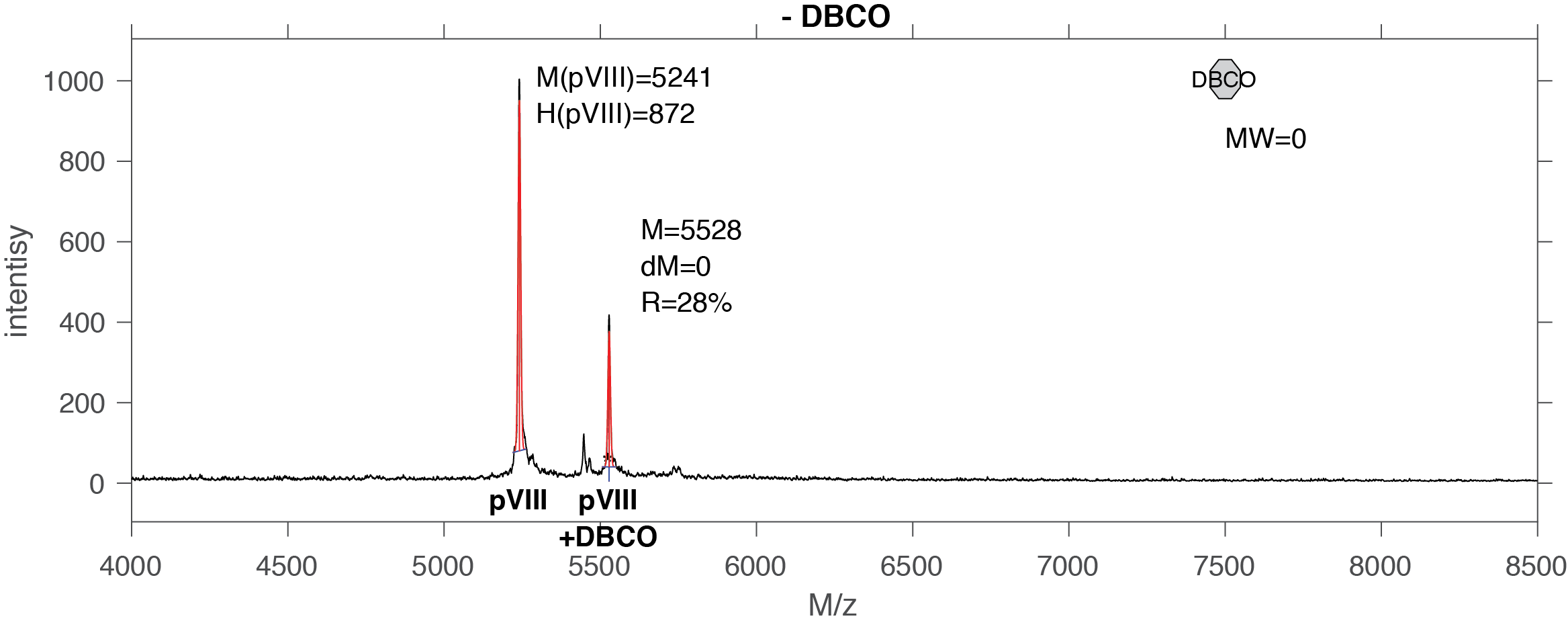


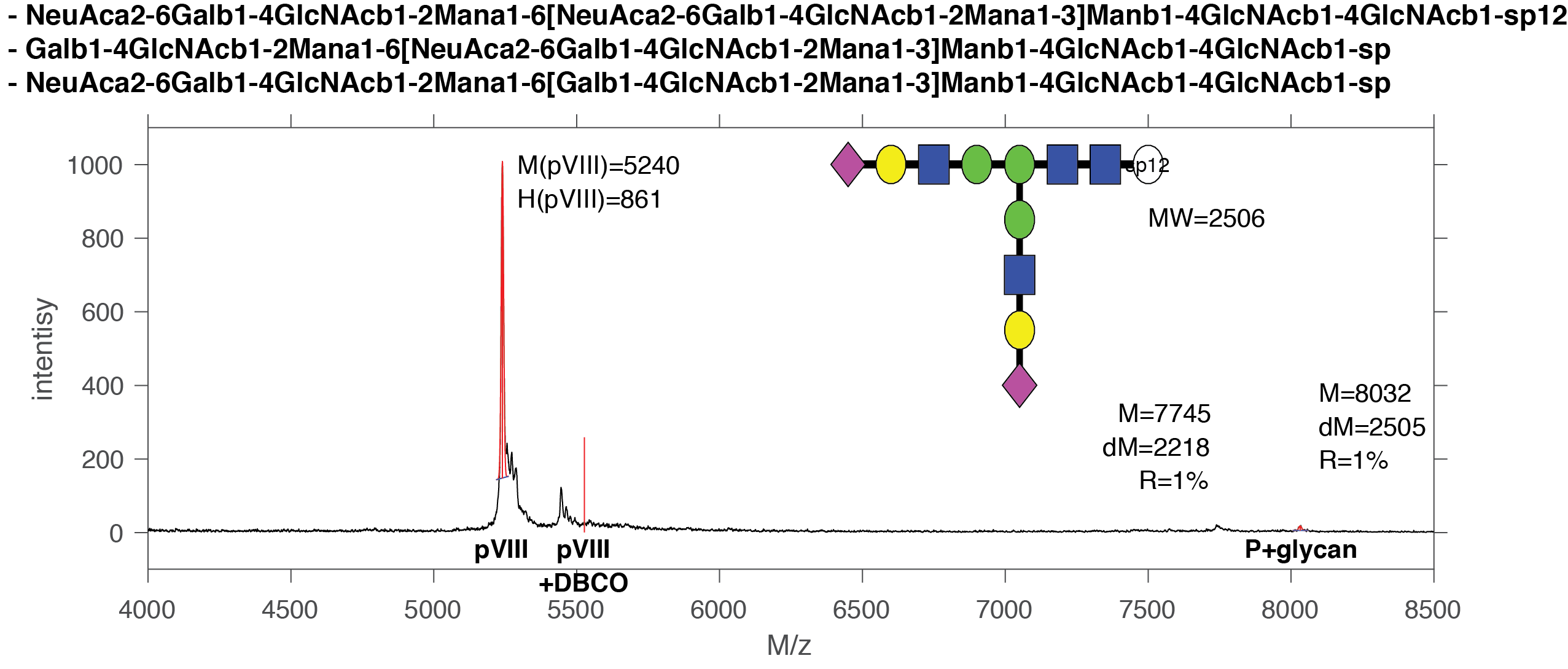


**SDB Number:** SDB43

**Barcode:** CTTCTGTTTGCTATTCCGCTCAGTGTGGAGAAGAATGATCAAAAAACCTACCATGCCGGGGGAGGG

**Axis Name:** 8-[760]

**IUPAC :** Neu5Ac(a2-6)Gal(b1-4)GlcNAc(b1-2)Man(a1-6)[Neu5Ac(a2-6)Gal(b1-4)GlcNAc(b1-2)Man(a1-3)]Man(b1-4)GlcNAc(b1-4)GlcNAc(b1-Sp; Gal(b1-4)GlcNAc(b1-2)Man(a1-6)[Neu5Ac(a2-6)Gal(b1-4)GlcNAc(b1-2)Man(a1-3)]Man(b1-4)GlcNAc(b1-4)GlcNAc(b1-Sp; Neu5Ac(a2-6)Gal(b1-4)GlcNAc(b1-2)Man(a1-6)[Gal(b1-4)GlcNAc(b1-2)Man(a1-3)]Man(b1-4)GlcNAc(b1-4)GlcNAc(b1-Sp

**Maldi File:** TL-III-127-DBCO-SDB43_0002.txt and TL-III-155-1_0322_0003.txt

**Density:** based on DBCO density was 28%


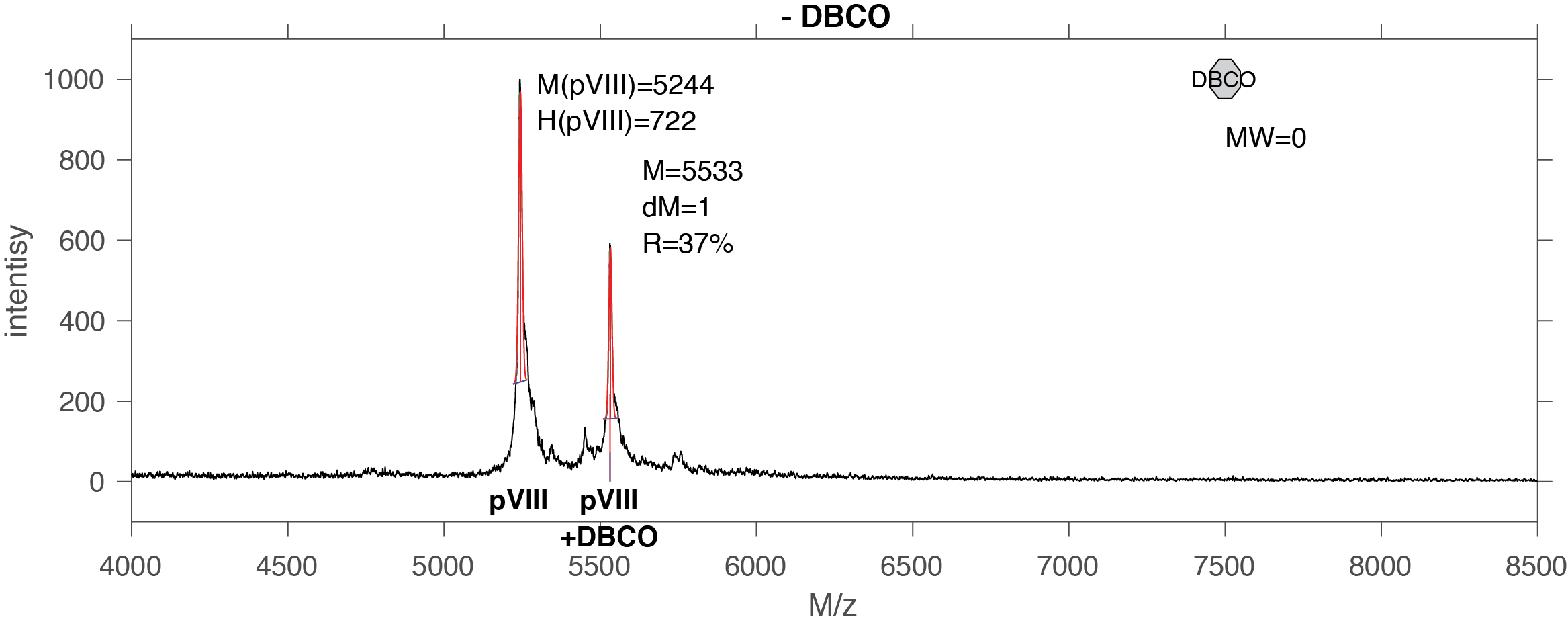


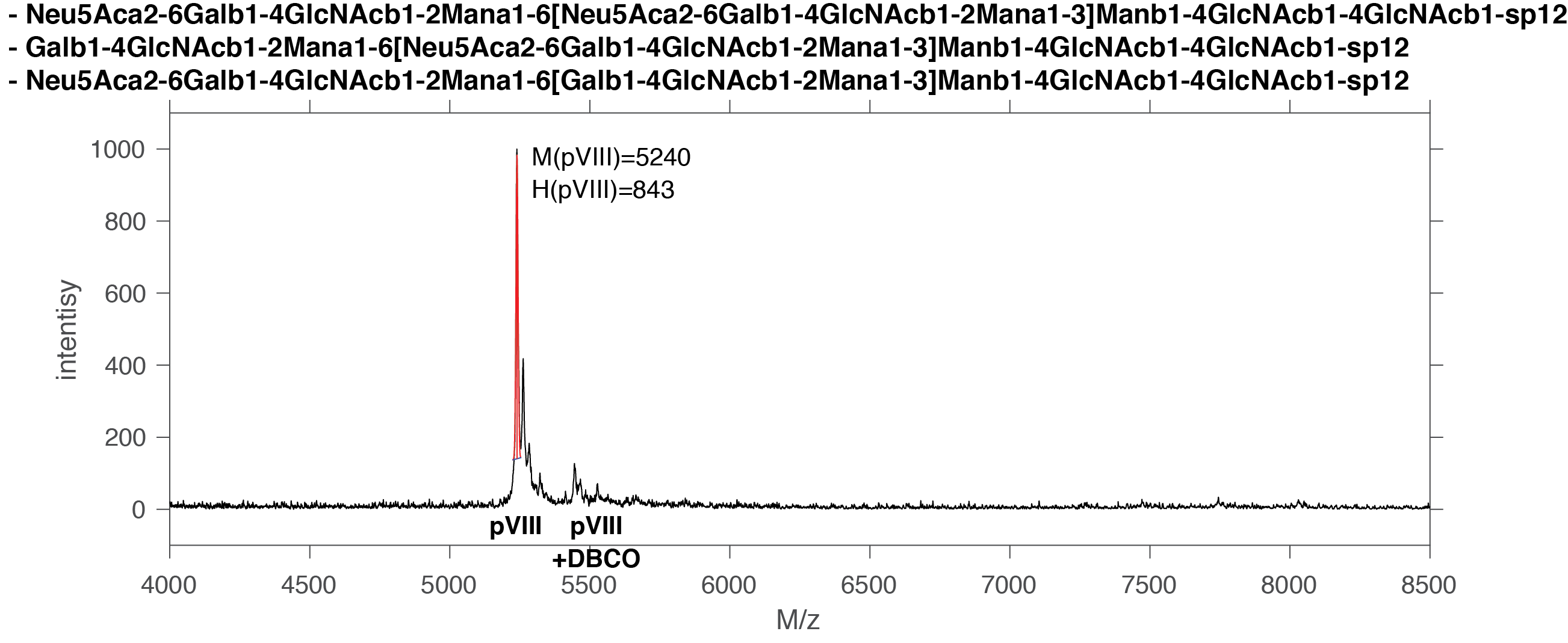


**SDB Number:** SDB171

**Barcode:** CTTCTCTTTGCAATCCCGCTGAGCGTCGAGAAGAATGACCAGAAGACATACCACGCTGGTGGCGGC

**Axis Name:** 8-[1000]

**IUPAC :** Neu5Ac(a2-6)Gal(b1-4)GlcNAc(b1-2)Man(a1-6)[Neu5Ac(a2-6)Gal(b1-4)GlcNAc(b1-2)Man(a1-3)]Man(b1-4)GlcNAc(b1-4)GlcNAc(b1-Sp; Gal(b1-4)GlcNAc(b1-2)Man(a1-6)[Neu5Ac(a2-6)Gal(b1-4)GlcNAc(b1-2)Man(a1-3)]Man(b1-4)GlcNAc(b1-4)GlcNAc(b1-Sp; Neu5Ac(a2-6)Gal(b1-4)GlcNAc(b1-2)Man(a1-6)[Gal(b1-4)GlcNAc(b1-2)Man(a1-3)]Man(b1-4)GlcNAc(b1-4)GlcNAc(b1-Sp

**Maldi File:** TL-IV-53-DBCO-SDB171_0004.txt and TL-IV-69-0608_0004.txt

**Density:** based on DBCO density was 37%


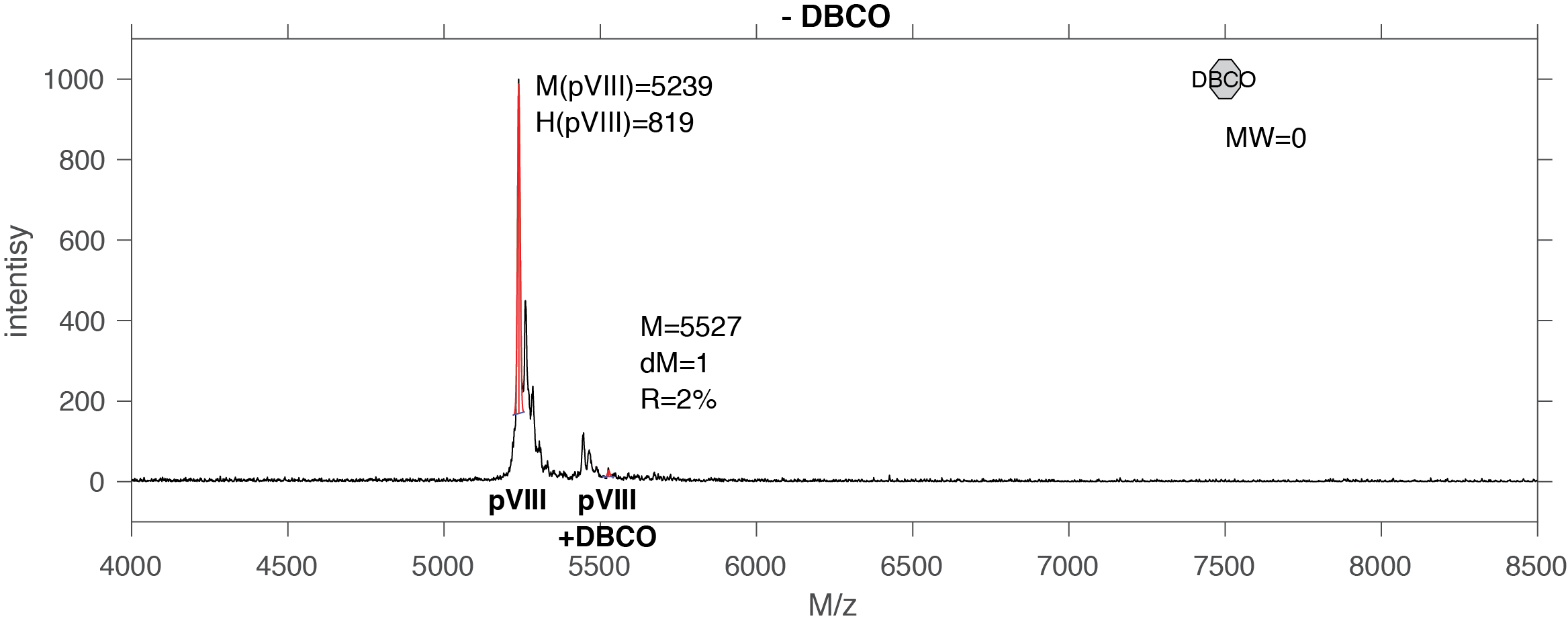


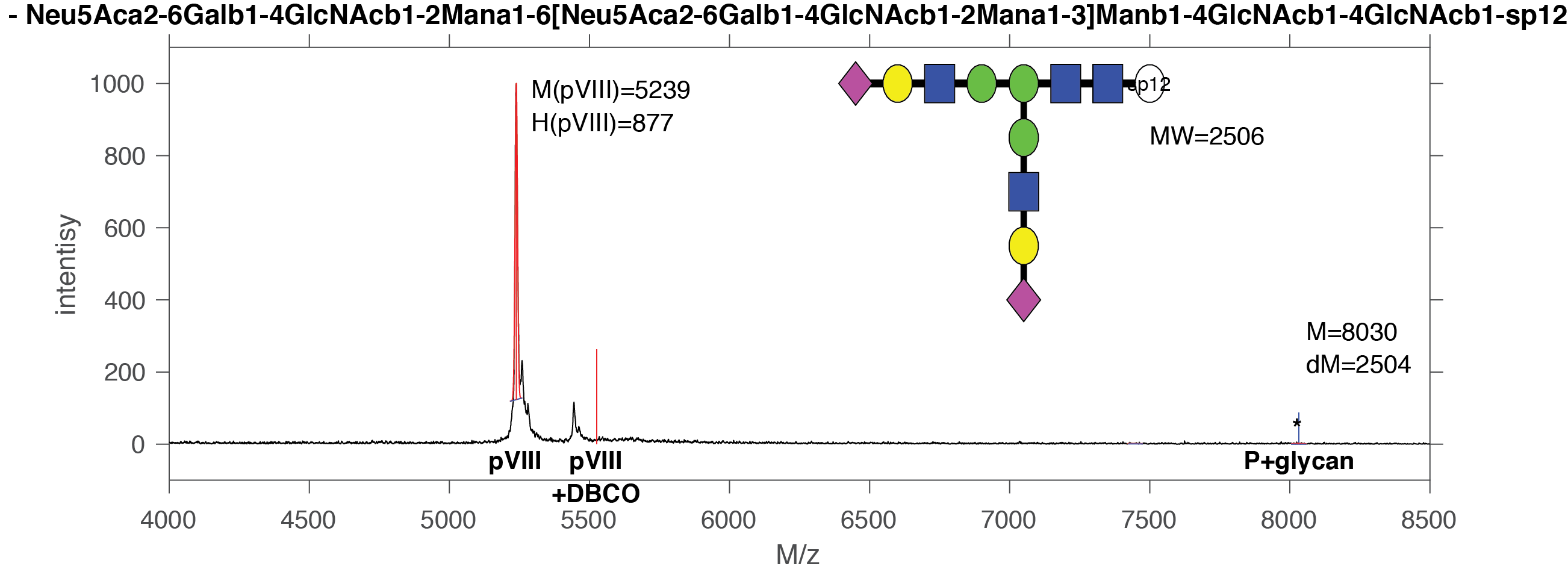


**SDB Number:** SDB108

**Barcode:** CTTCTGTTCGCGATCCCCCTAAGTGTGGAGAAAAATGACCAAAAGACTTATCATGCTGGGGGAGGT

**Axis Name:** 9-[50]

**IUPAC:** Neu5Ac(a2-6)Gal(b1-4)GlcNAc(b1-2)Man(a1-6)[Neu5Ac(a2-6)Gal(b1-4)GlcNAc(b1-2)Man(a1-3)]Man(b1-4)GlcNAc(b1-4)GlcNAc(b1-Sp

**Maldi File:** TL-IV-65-DBCO-SDB108_0002.txt and TL-IV-89-0614_0002.txt

**Density:** based on DBCO density was 2%


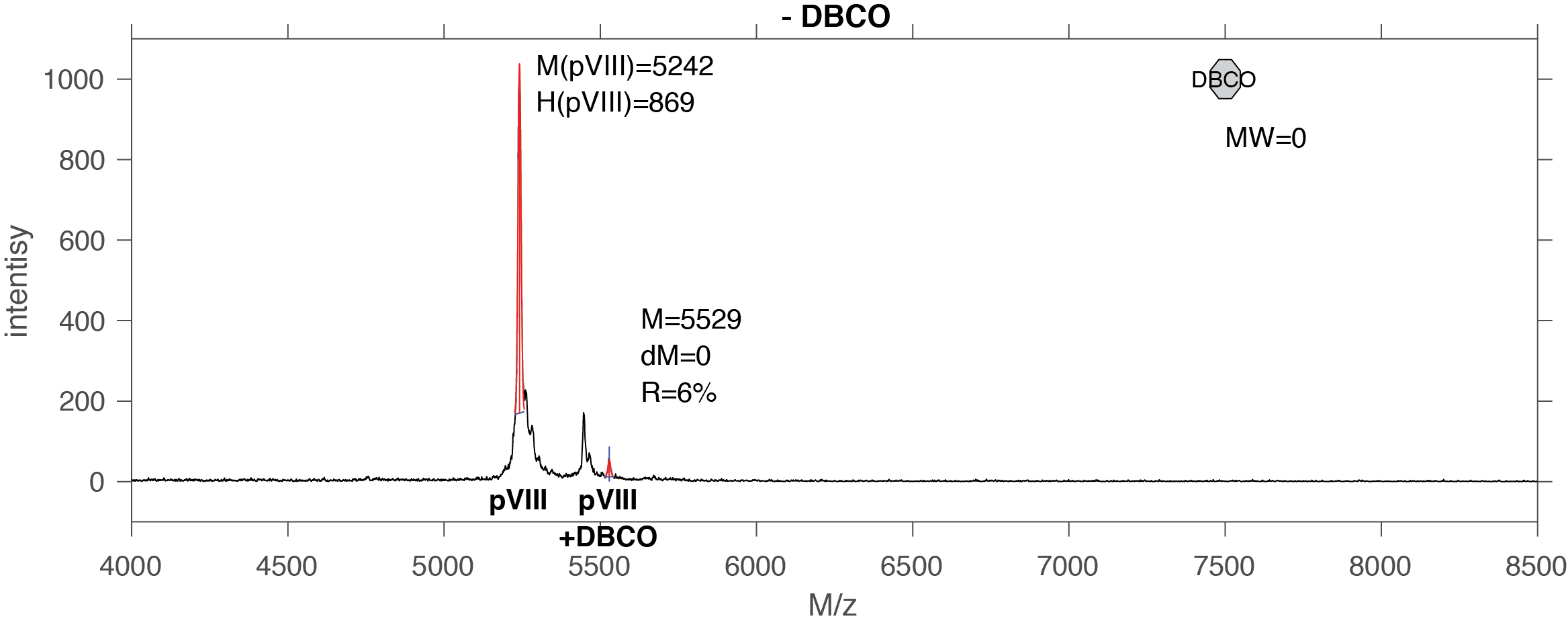


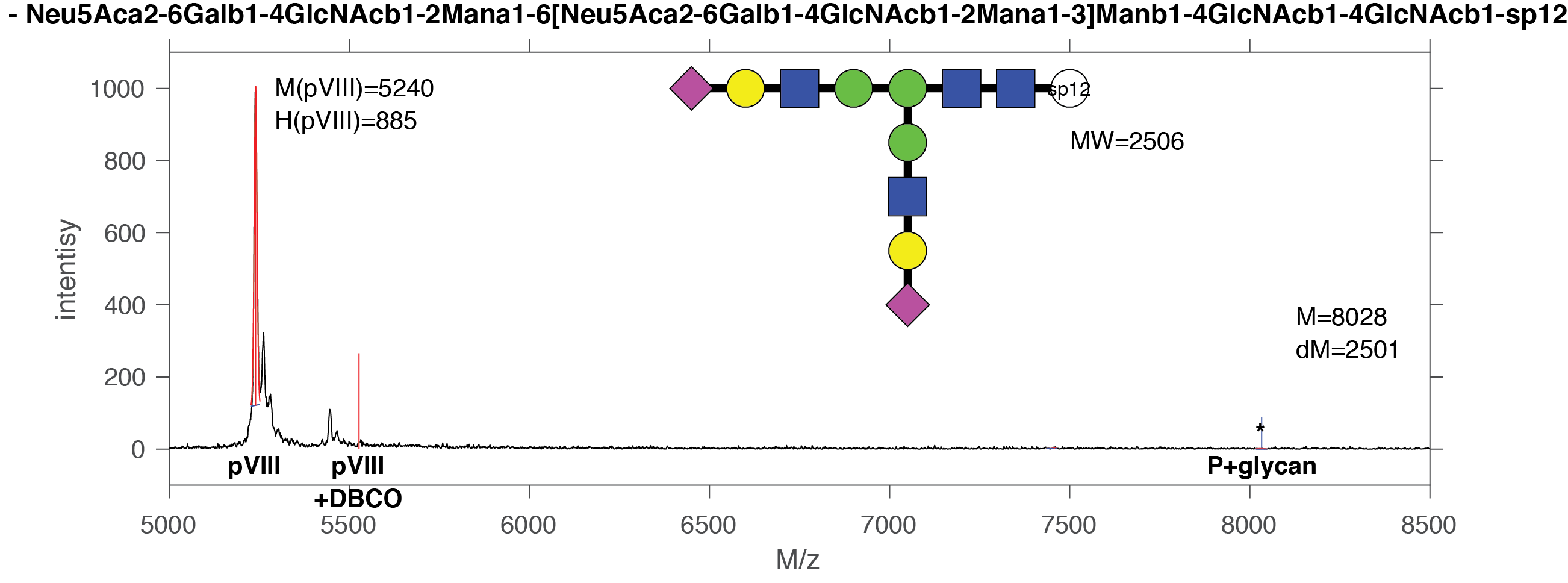


**SDB Number:** SDB219

**Barcode:** TTATTATTCGCAATTCCTTTAAGTGTGGAAAAGAACGATCAAAAAACTTATCACGCGGGTGGGGGC

**Axis Name:** 9-[160]

**IUPAC:** Neu5Ac(a2-6)Gal(b1-4)GlcNAc(b1-2)Man(a1-6)[Neu5Ac(a2-6)Gal(b1-4)GlcNAc(b1-2)Man(a1-3)]Man(b1-4)GlcNAc(b1-4)GlcNAc(b1-Sp

**Maldi File:** TL-IV-59-SDB219-8min_0003.txt and TL-IV-87-0614_0002.txt

**Density:** based on DBCO density was 6%


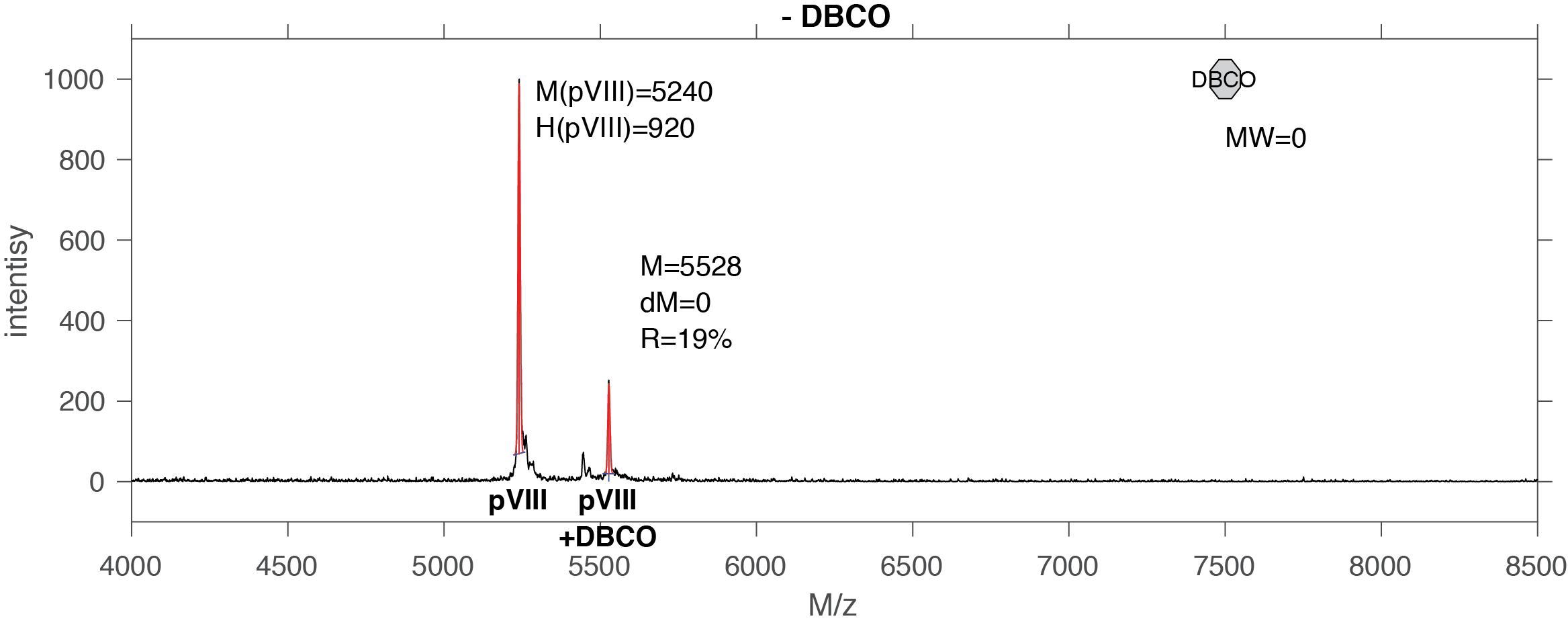


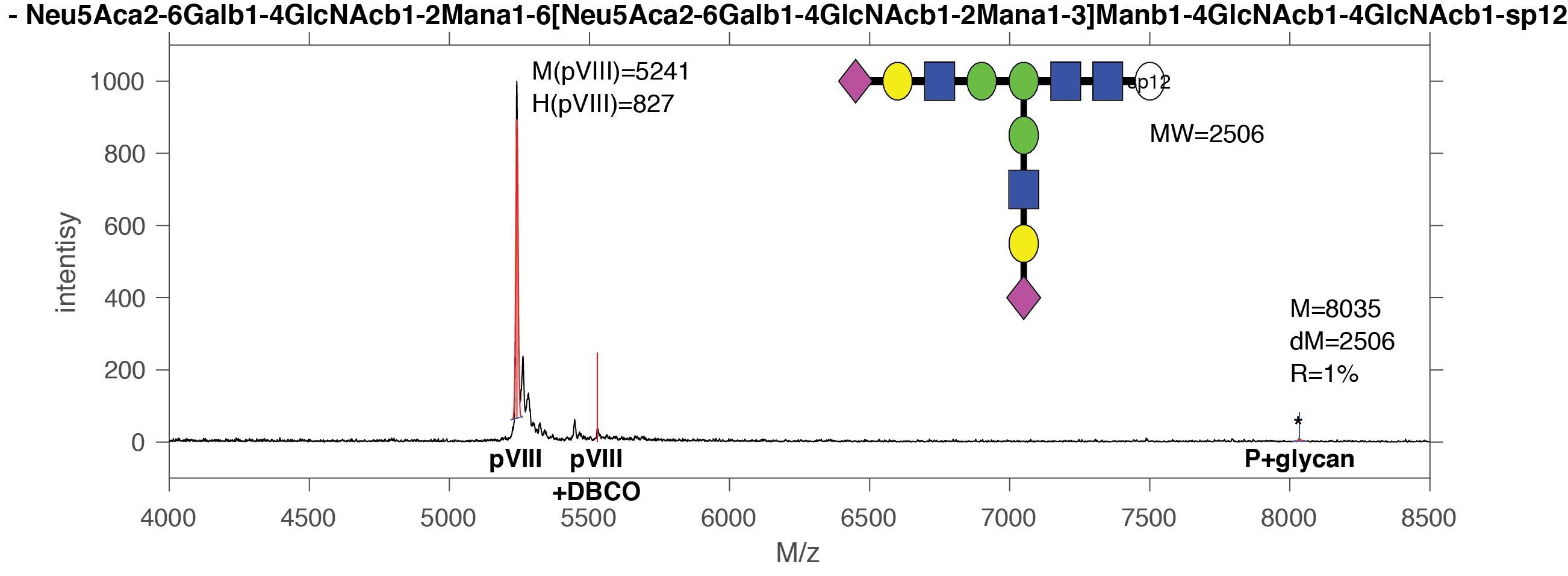


**SDB Number:** SDB110

**Barcode:** CTGCTGTTTGCGATCCCGCTAAGCGTGGAAAAGAACGATCAGAAGACGTACCATGCGGGAGGGGGG

**Axis Name:** 9-[510]

**IUPAC:** Neu5Ac(a2-6)Gal(b1-4)GlcNAc(b1-2)Man(a1-6)[Neu5Ac(a2-6)Gal(b1-4)GlcNAc(b1-2)Man(a1-3)]Man(b1-4)GlcNAc(b1-4)GlcNAc(b1-Sp

**Maldi File:** TL-IV-79-DBCO-SDB110_0003.txt and TL-IV-81-0614_0007.txt

**Density:** based on DBCO density was 19%


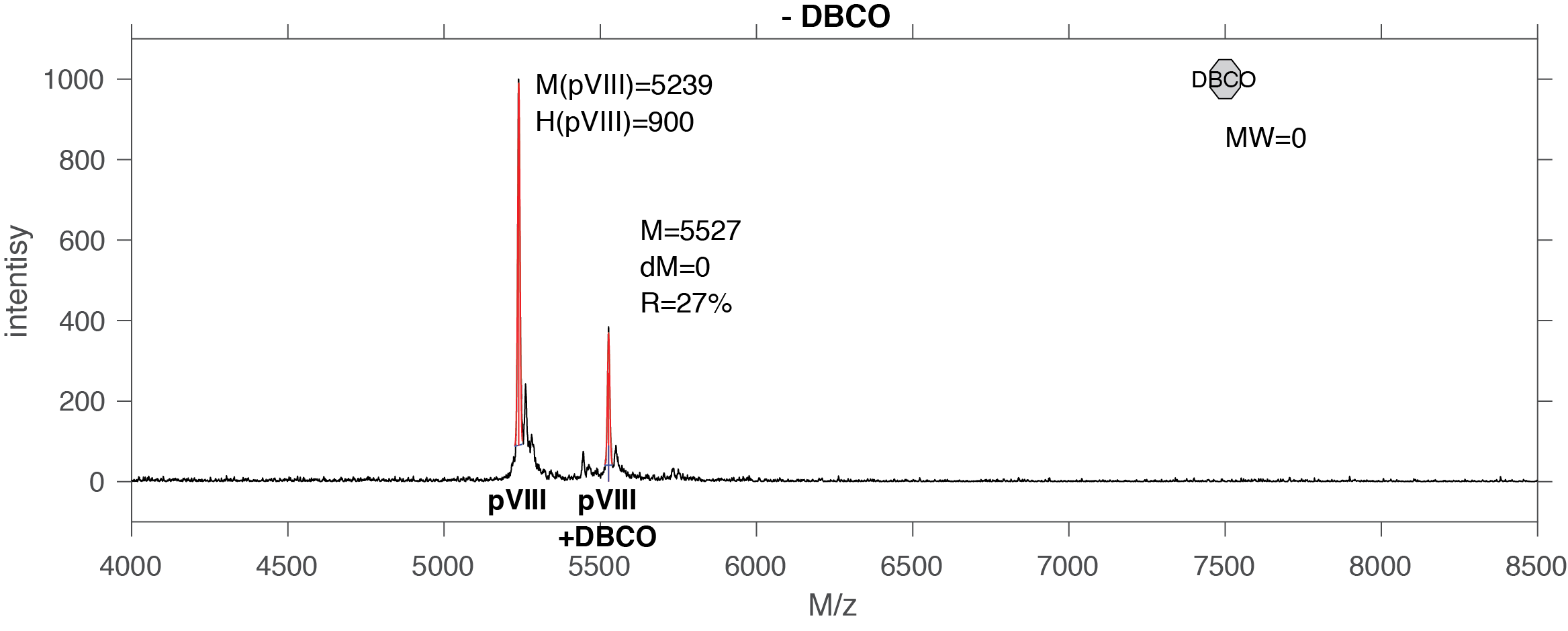


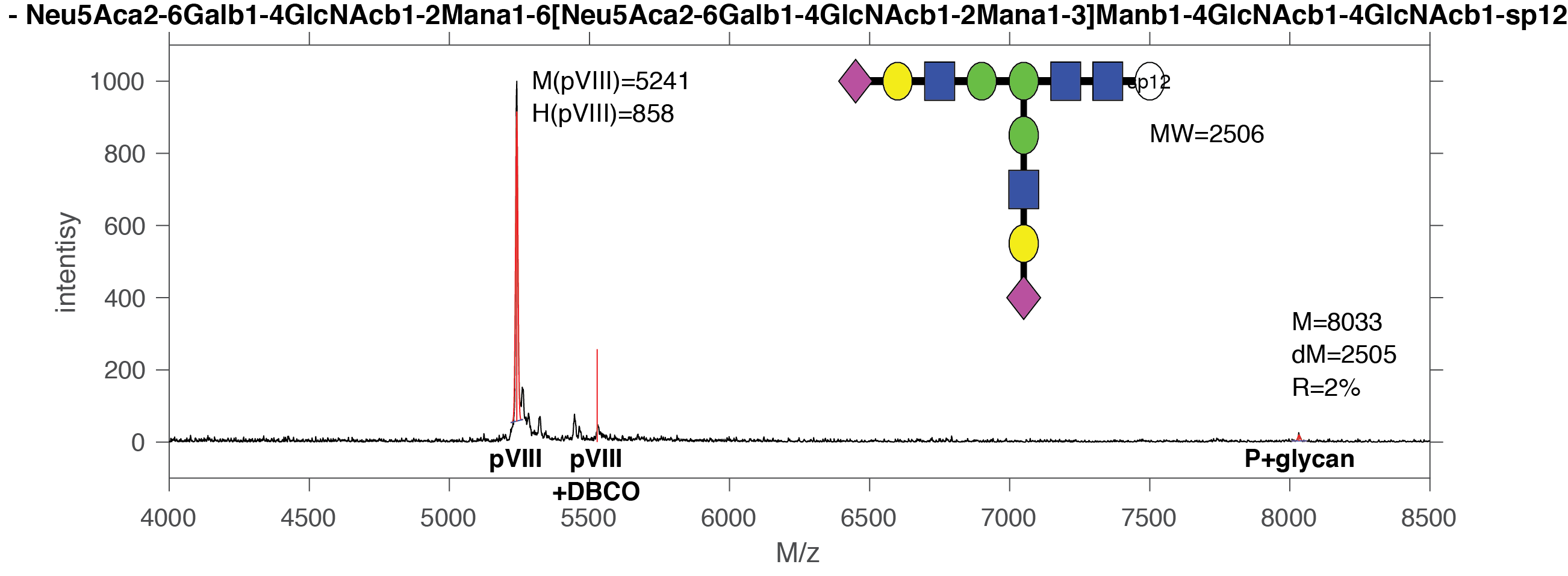


**SDB Number:** SDB158

**Barcode:** CTACTTTTTGCTATTCCGCTGAGCGTGGAGAAGAATGATCAGAAGACATACCATGCGGGTGGAGGG

**Axis Name:** 9-[730]

**IUPAC:** Neu5Ac(a2-6)Gal(b1-4)GlcNAc(b1-2)Man(a1-6)[Neu5Ac(a2-6)Gal(b1-4)GlcNAc(b1-2)Man(a1-3)]Man(b1-4)GlcNAc(b1-4)GlcNAc(b1-Sp

**Maldi File:** TL-IV-79-DBCO-SDB158-30min_0004.txt and TL-IV-85-0614_0002.txt

**Density:** based on DBCO density was 27%


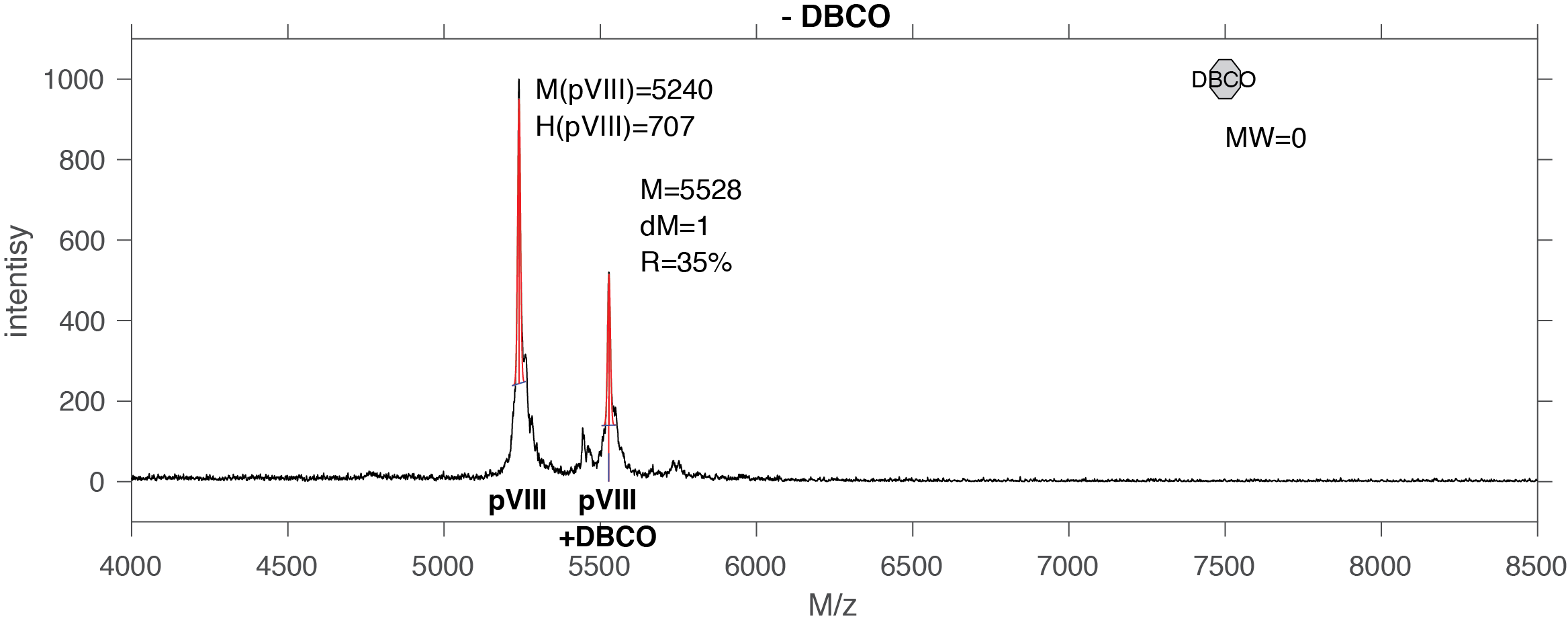


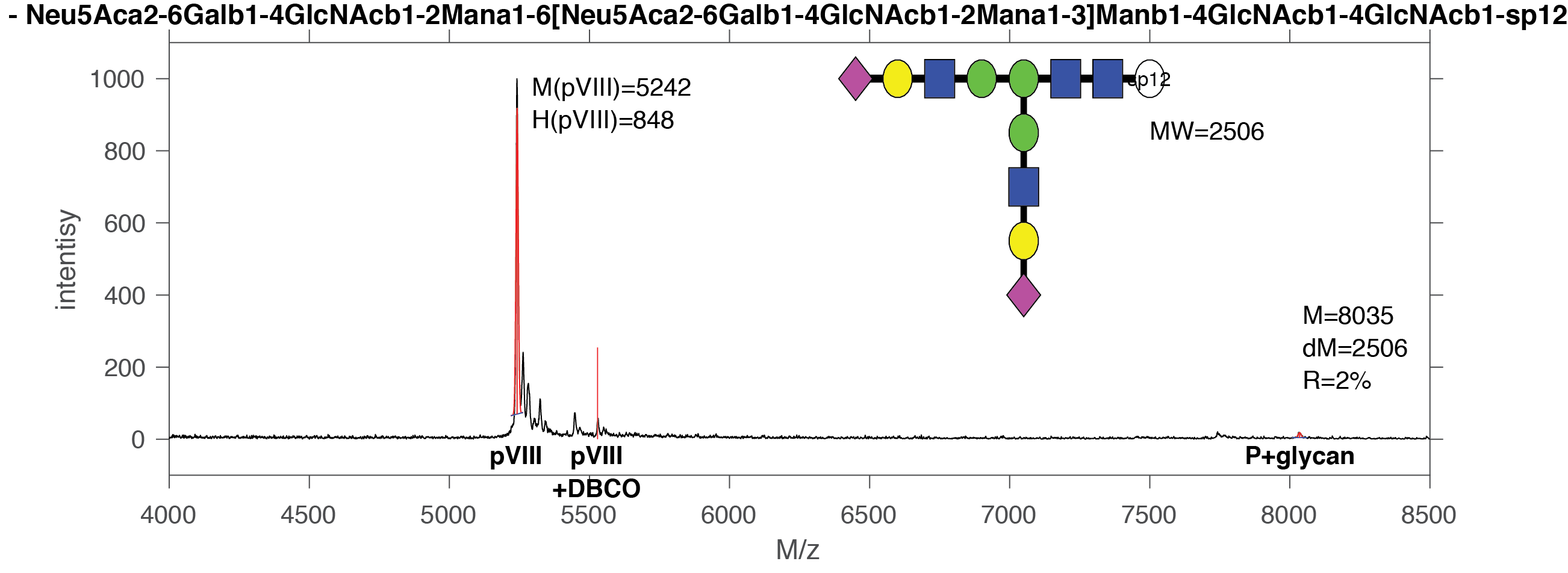


**SDB Number:** SDB156

**Barcode:** TTATTATTCGCAATTCCTTTAAGTGTCGAGAAAAACGATCAAAAGACCTACCACGCAGGGGGTGGT

**Axis Name:** 9-[950]

**IUPAC:** Neu5Ac(a2-6)Gal(b1-4)GlcNAc(b1-2)Man(a1-6)[Neu5Ac(a2-6)Gal(b1-4)GlcNAc(b1-2)Man(a1-3)]Man(b1-4)GlcNAc(b1-4)GlcNAc(b1-Sp

**Maldi File:** TL-IV-79-DBCO-SDB156_0005.txt and TL-IV-83-0614_0004.txt and

**Density:** based on DBCO density was 35%


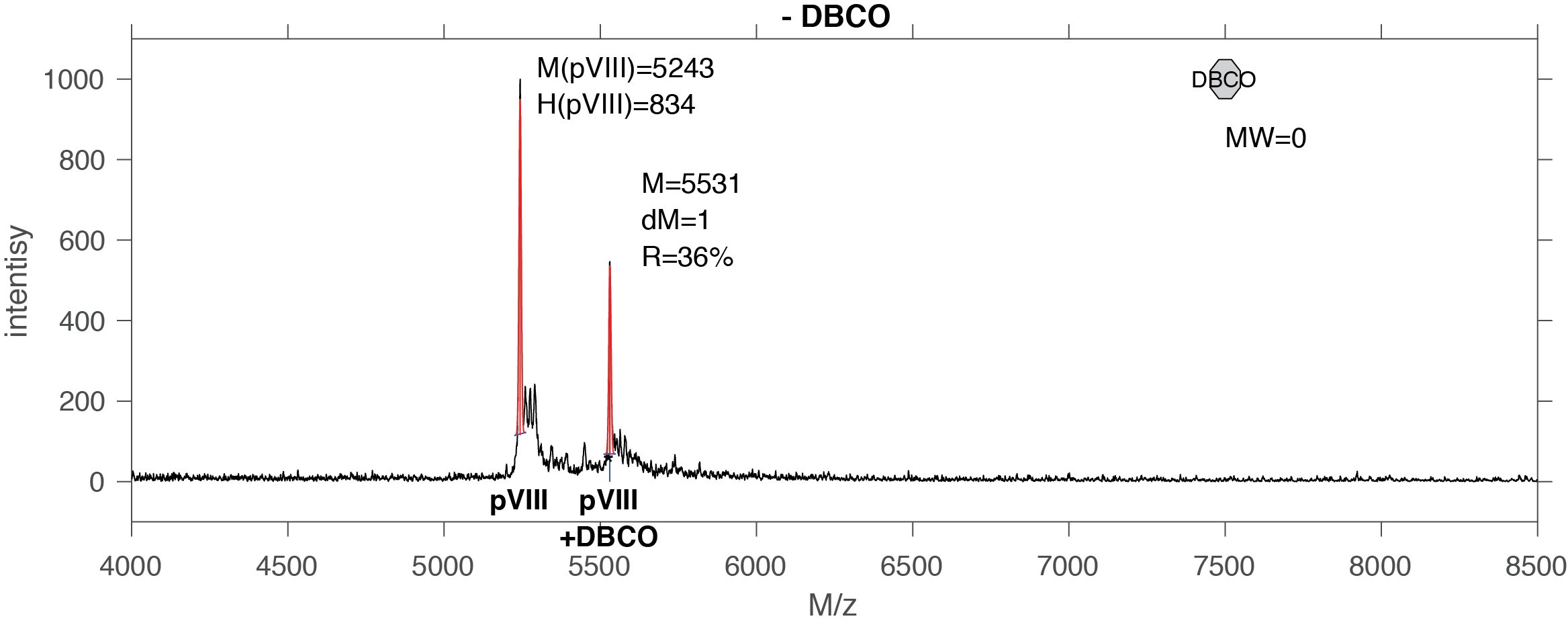


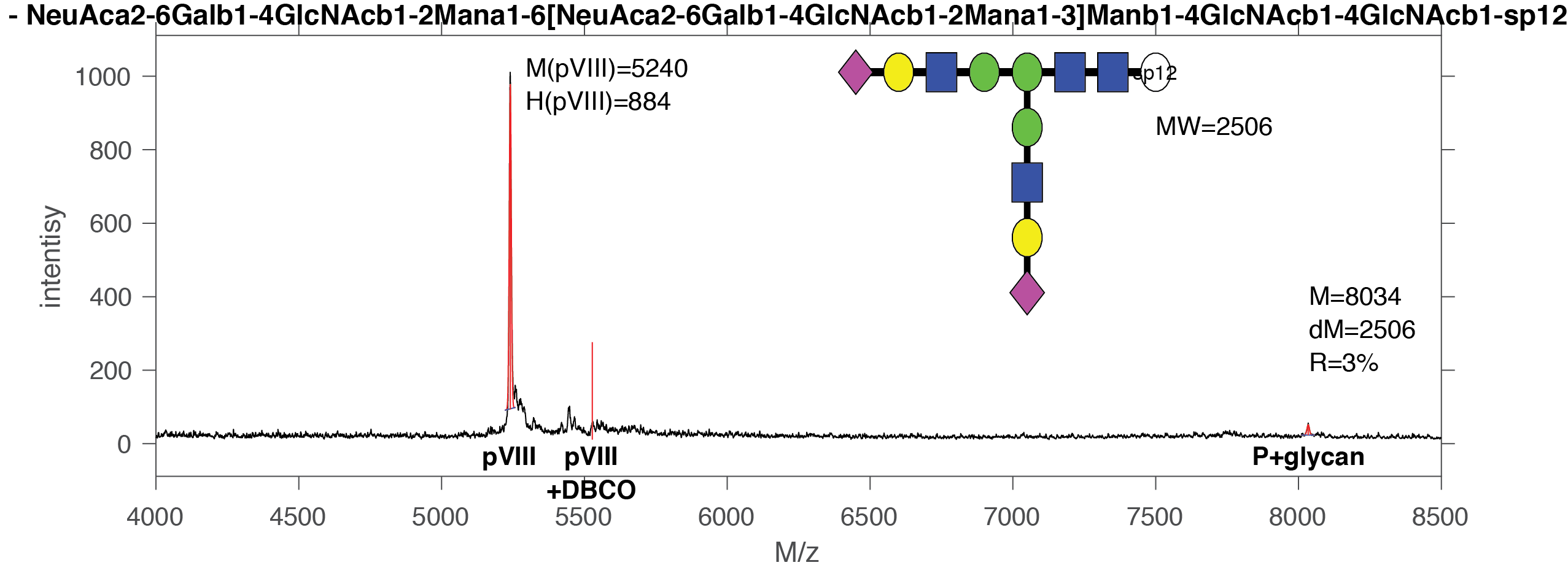


**SDB Number:** SDB147

**Barcode:** CTACTGTTCGCAATCCCGCTAAGCGTTGAGAAGAACGATCAAAAAACATACCACGCAGGTGGGGGA

**Axis Name:** 9-[970]

**IUPAC:** Neu5Ac(a2-6)Gal(b1-4)GlcNAc(b1-2)Man(a1-6)[Neu5Ac(a2-6)Gal(b1-4)GlcNAc(b1-2)Man(a1-3)]Man(b1-4)GlcNAc(b1-4)GlcNAc(b1-Sp

**Maldi File:** TL-III-157-DBCO-SDB147_0001.txt and TL-III-189-1-afterPEG_0010.txt

**Density:** based on DBCO density was 36%


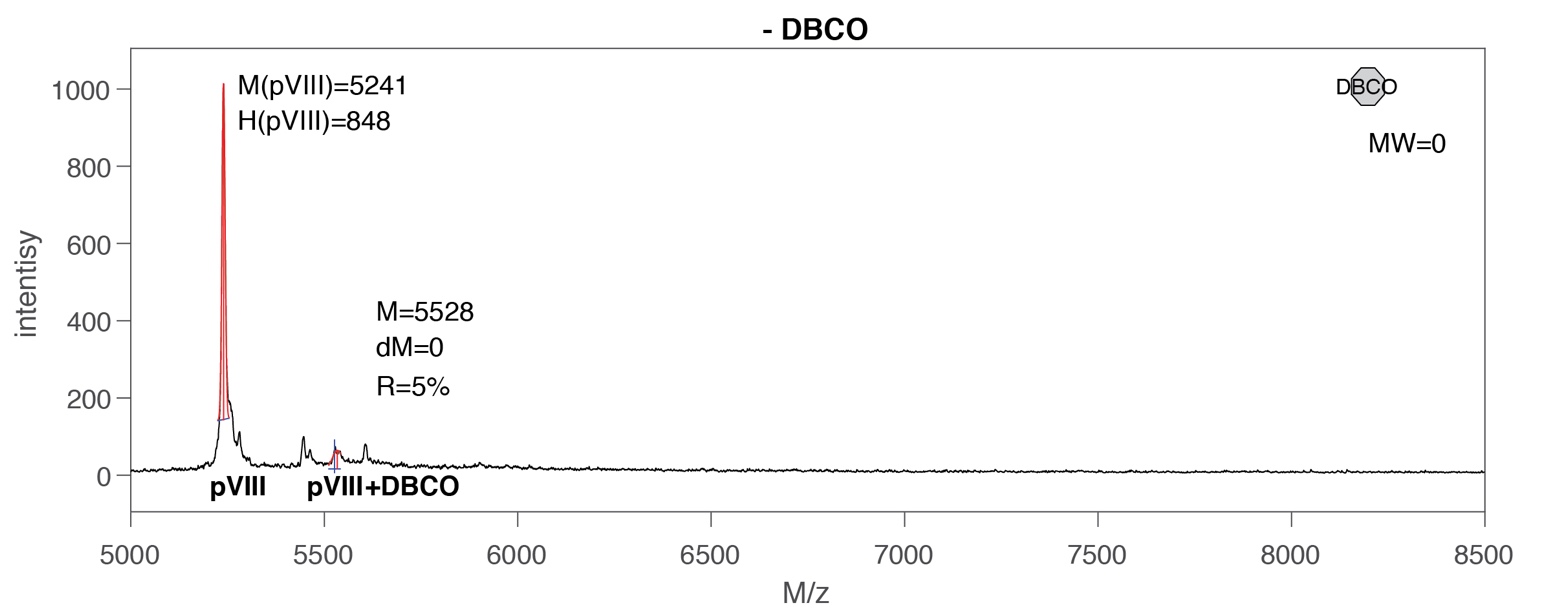


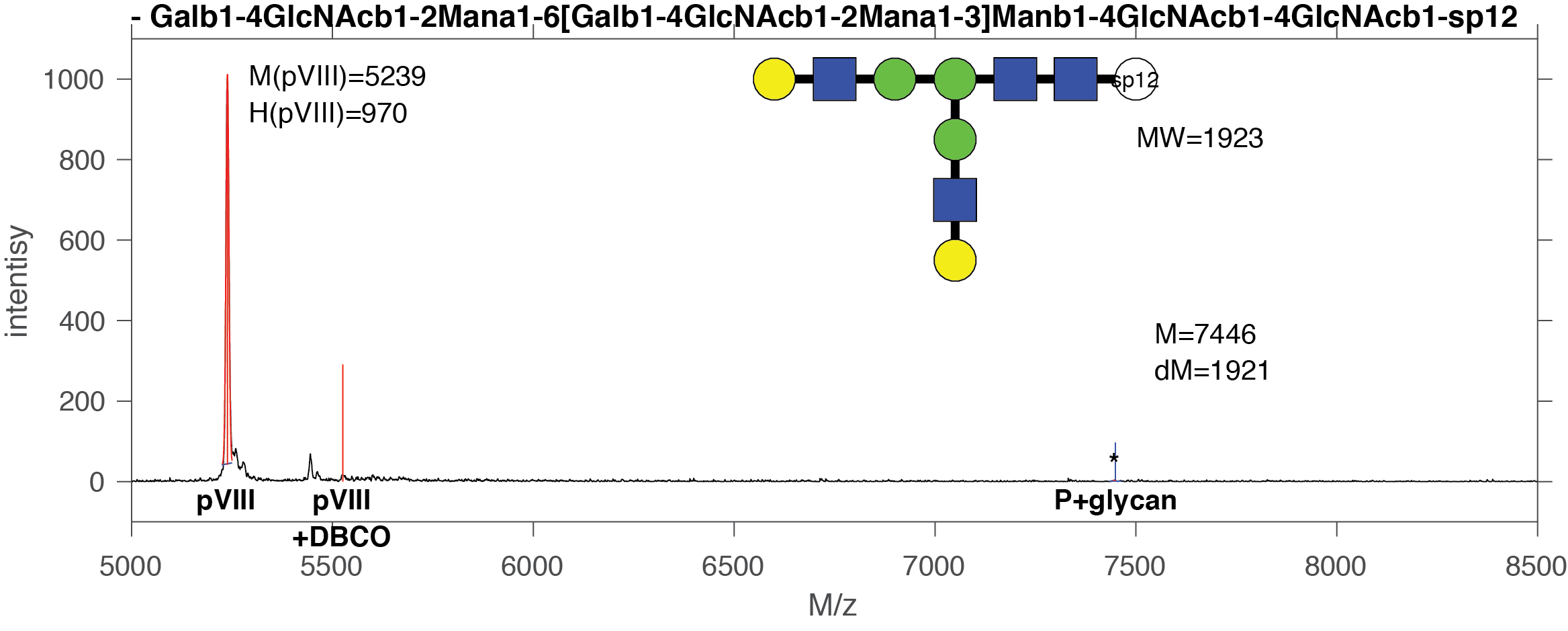


**SDB Number:** SDB101

**Barcode:** CTACTTTTTGCTATTCCTCTCAGTGTTGAAAAAAACGATCAGAAGACTTACCATGCGGGTGGGGGT

**Axis Name:** 10-[150]

**IUPAC:** Gal(b1-4)GlcNAc(b1-2)Man(a1-6)[Gal(b1-4)GlcNAc(b1-2)Man(a1-3)]Man(b1-4)GlcNAc(b1-4)GlcNAc(b1-Sp

**Maldi File:** TL-IV-135-SDB101_0001.txt and TL-IV-136-SDB101_0003.txt

**Density:** based on DBCO density was 5%


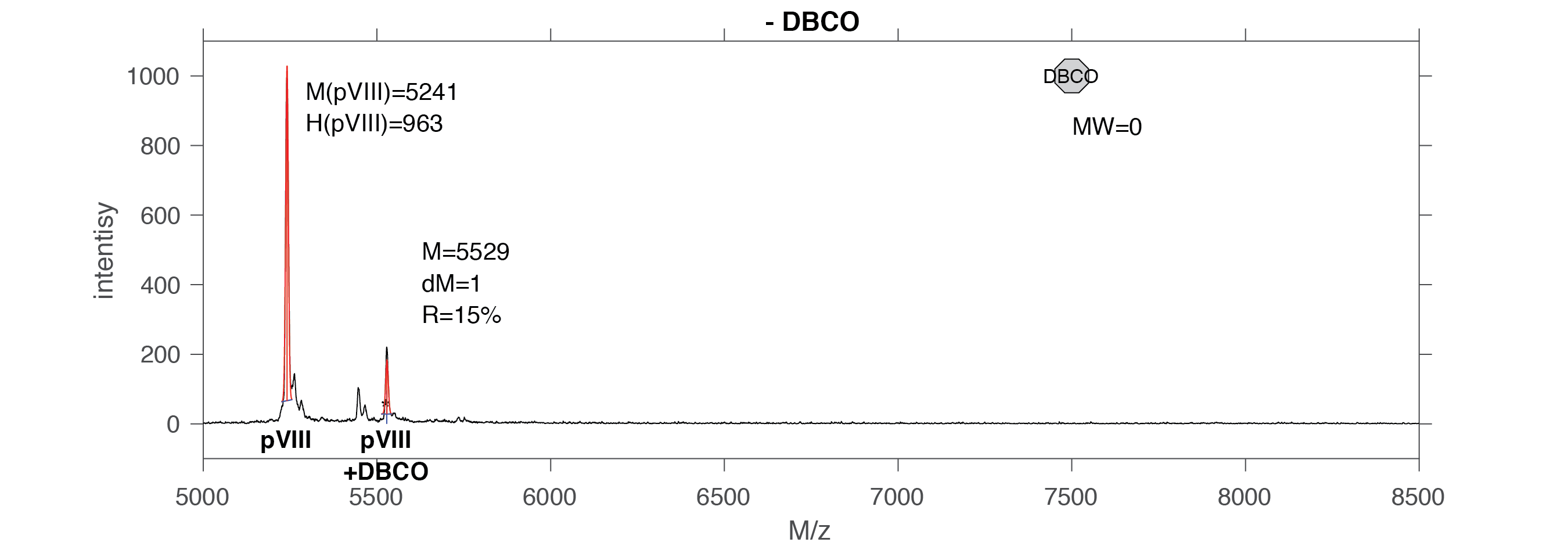


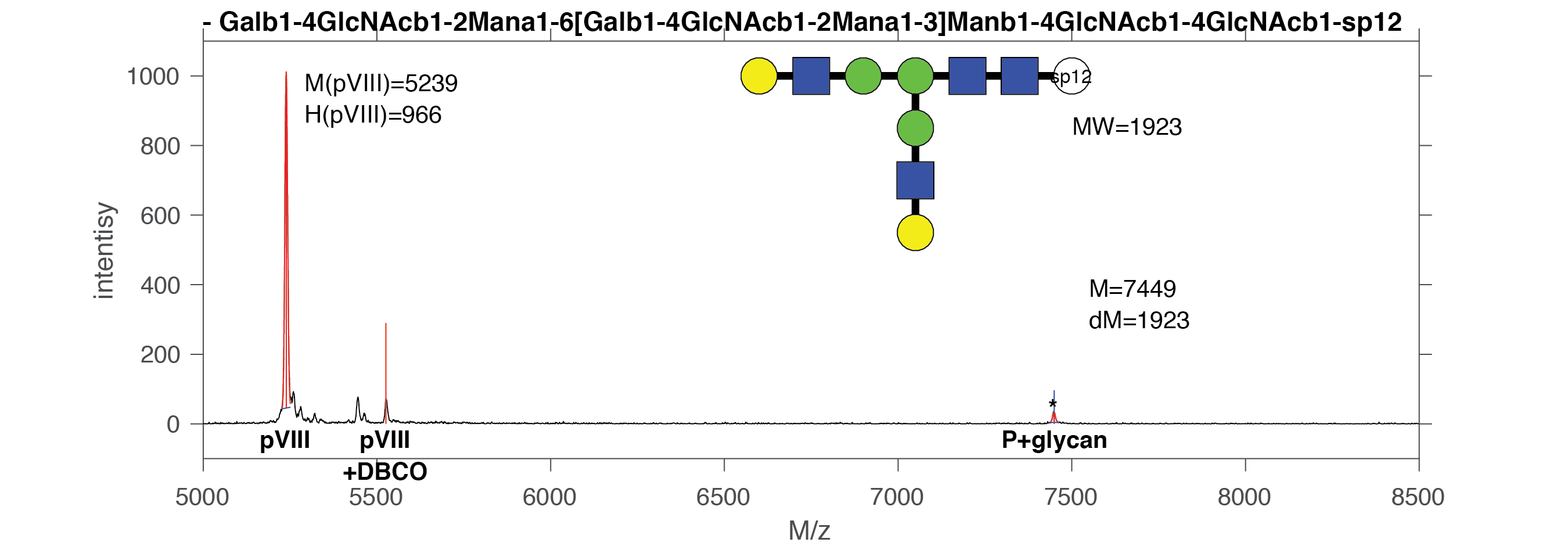


**SDB Number:** SDB119

**Barcode:** CTGCTTTTTGCCATCCCTCTTAGTGTCGAGAAAAACGACCAGAAAACATACCACGCCGGAGGGGGT

**Axis Name:** 10-[500]

**IUPAC:** Gal(b1-4)GlcNAc(b1-2)Man(a1-6)[Gal(b1-4)GlcNAc(b1-2)Man(a1-3)]Man(b1-4)GlcNAc(b1-4)GlcNAc(b1-Sp

**Maldi File:**  TL-IV-131-DBCO-SDB119-17.5min_0003.txt and TL-IV-136-SDB119_0003.txt

**Density:** based on DBCO density was 15%


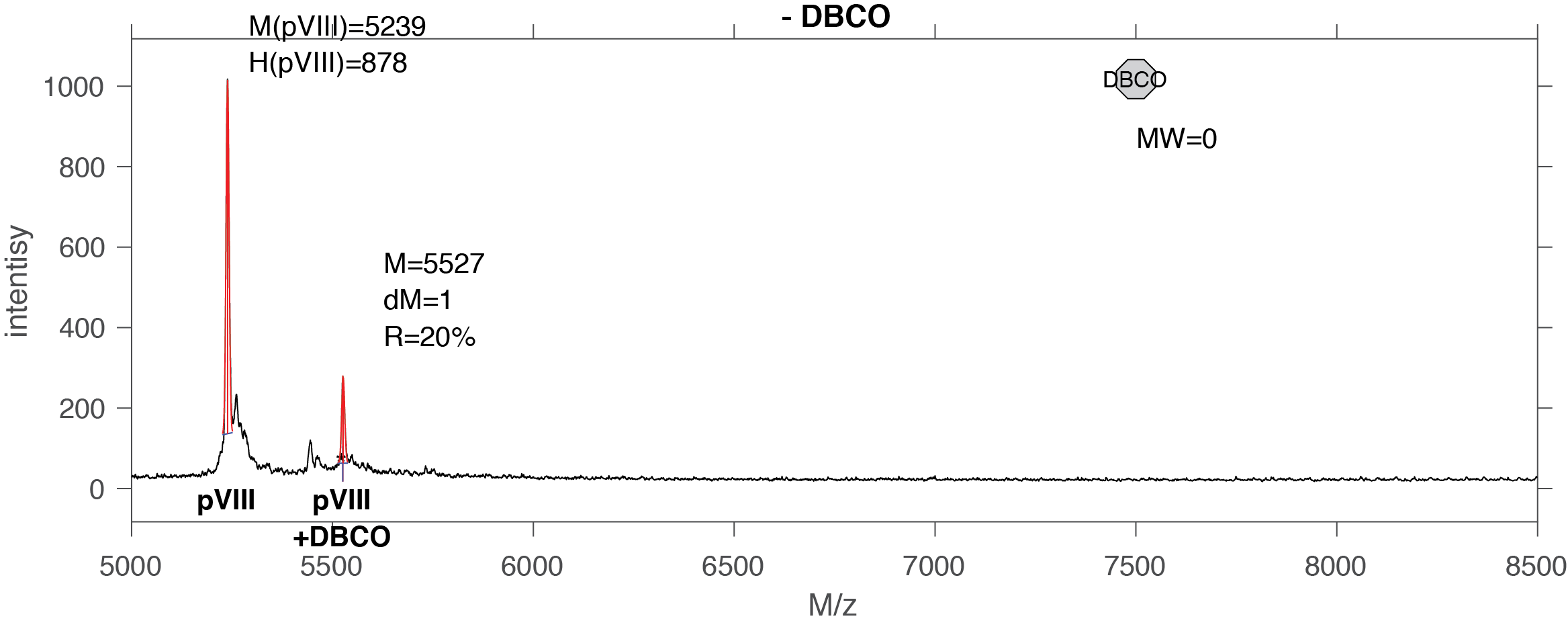


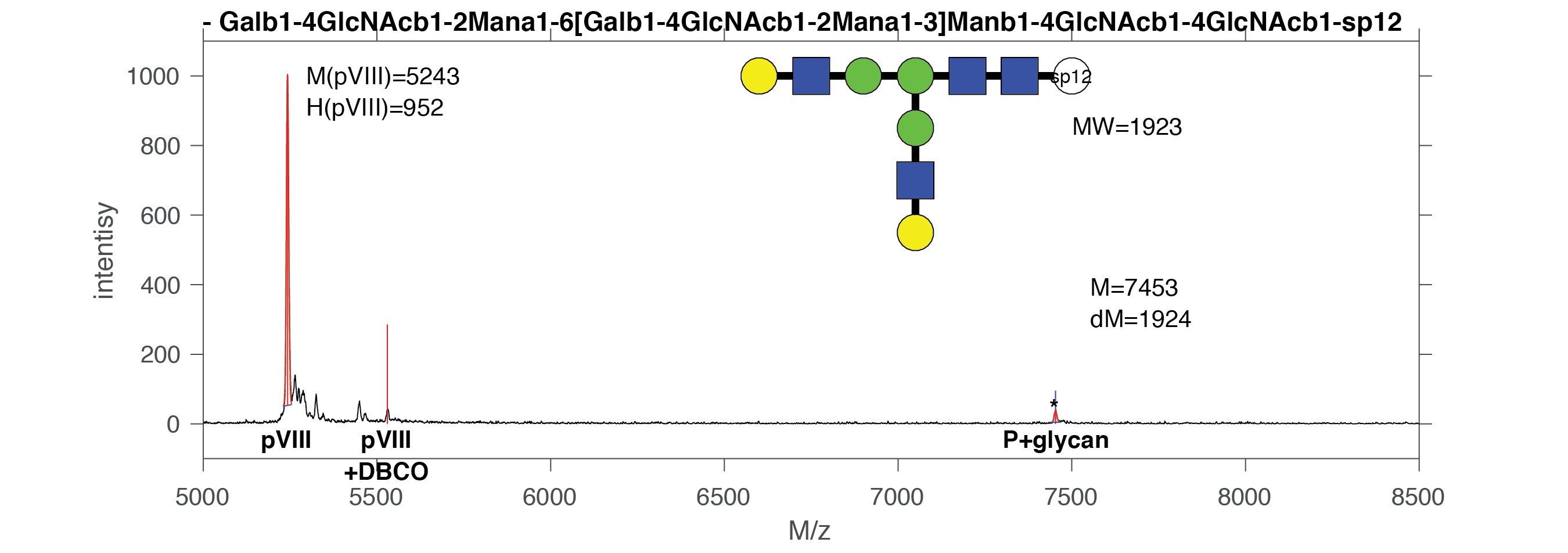


**SDB Number:** SDB37

**Barcode:** CTTCTTTTTGCCATCCCGCTCAGTGTGGAGAAGAATGATCAGAAGACTTATCATGCGGGTGGAGGT

**Axis Name:** 10-[540]

**IUPAC:** Gal(b1-4)GlcNAc(b1-2)Man(a1-6)[Gal(b1-4)GlcNAc(b1-2)Man(a1-3)]Man(b1-4)GlcNAc(b1-4)GlcNAc(b1-Sp

**Maldi File:**  TL-IV-117-DBCO-SDB37_0003.txt and TL-IV-117_0004.txt

**Density:** based on DBCO density was 20%


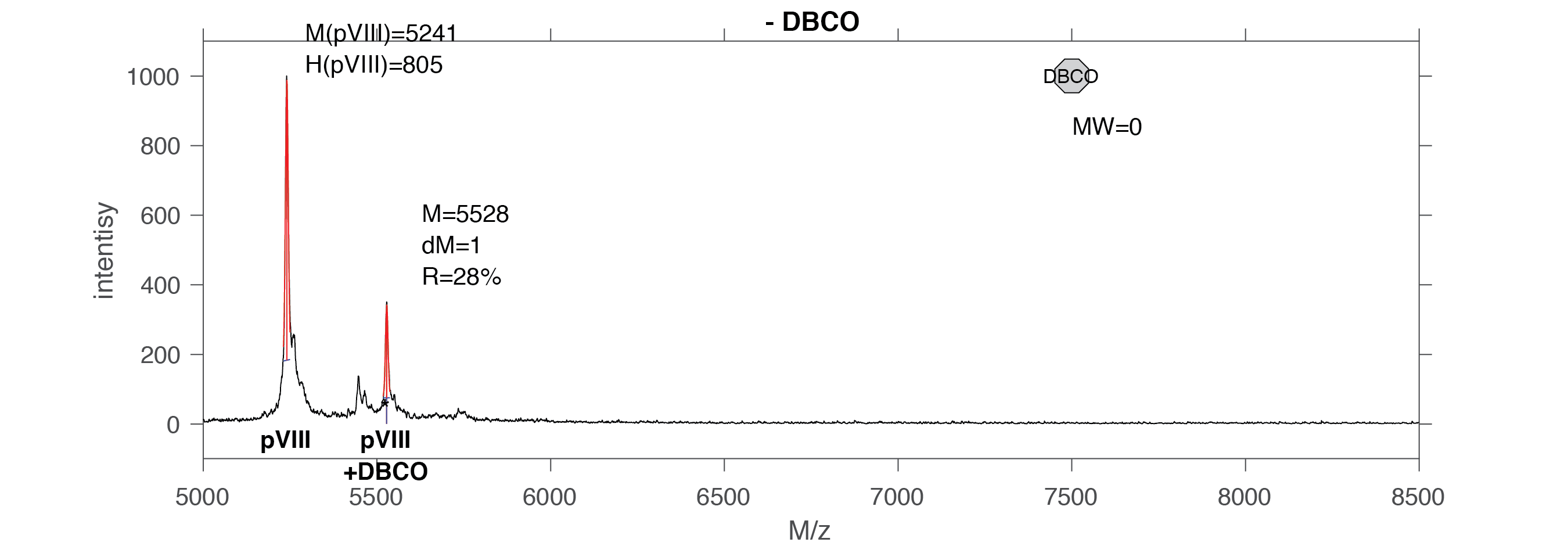


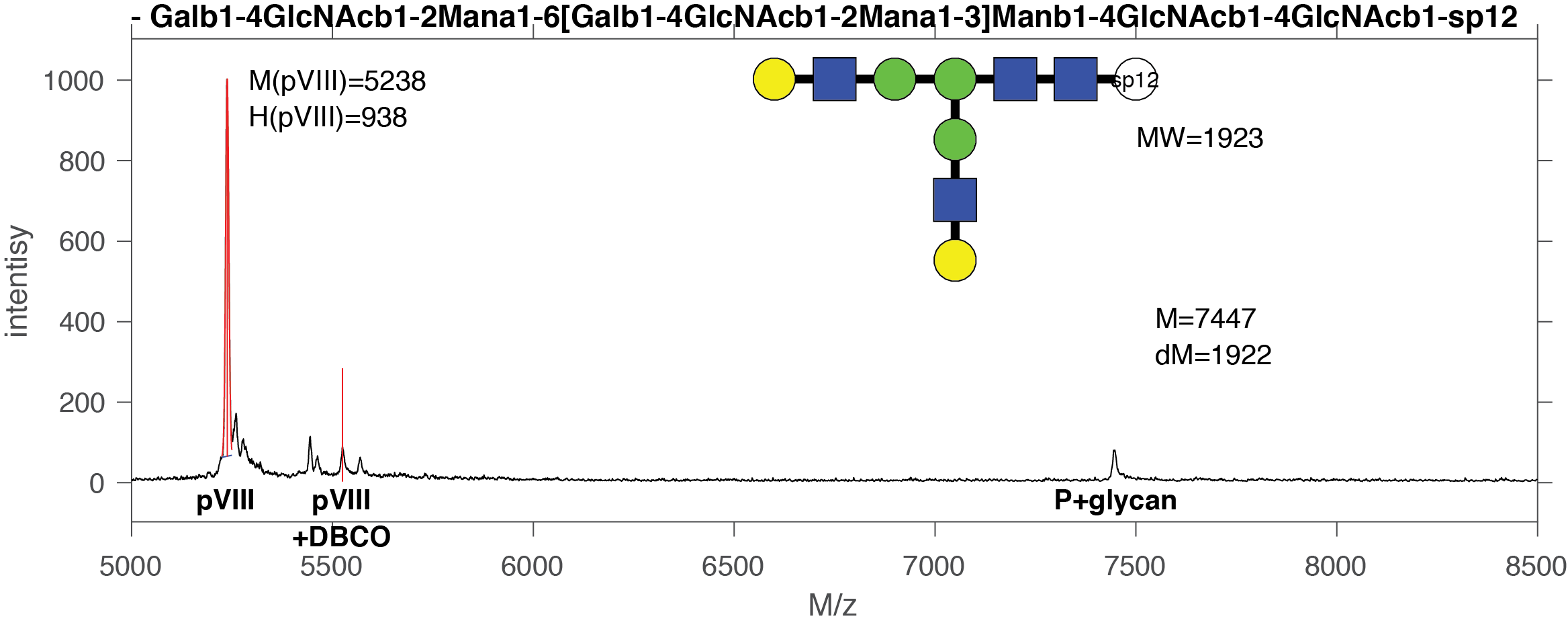


**SDB Number:** SDB93

**Barcode:** TTATTATTCGCAATTCCTTTAAGTGTAGAAAAGAACGATCAAAAGACTTATCATGCGGGGGGGGGA

**Axis Name:** 10-[750]

**IUPAC:** Gal(b1-4)GlcNAc(b1-2)Man(a1-6)[Gal(b1-4)GlcNAc(b1-2)Man(a1-3)]Man(b1-4)GlcNAc(b1-4)GlcNAc(b1-Sp

**Maldi File:**  TL-IV-131-DBCO-SDB93_0004.txt and TL-IV-138_0002.txt

**Density:** based on DBCO density was 28%

**
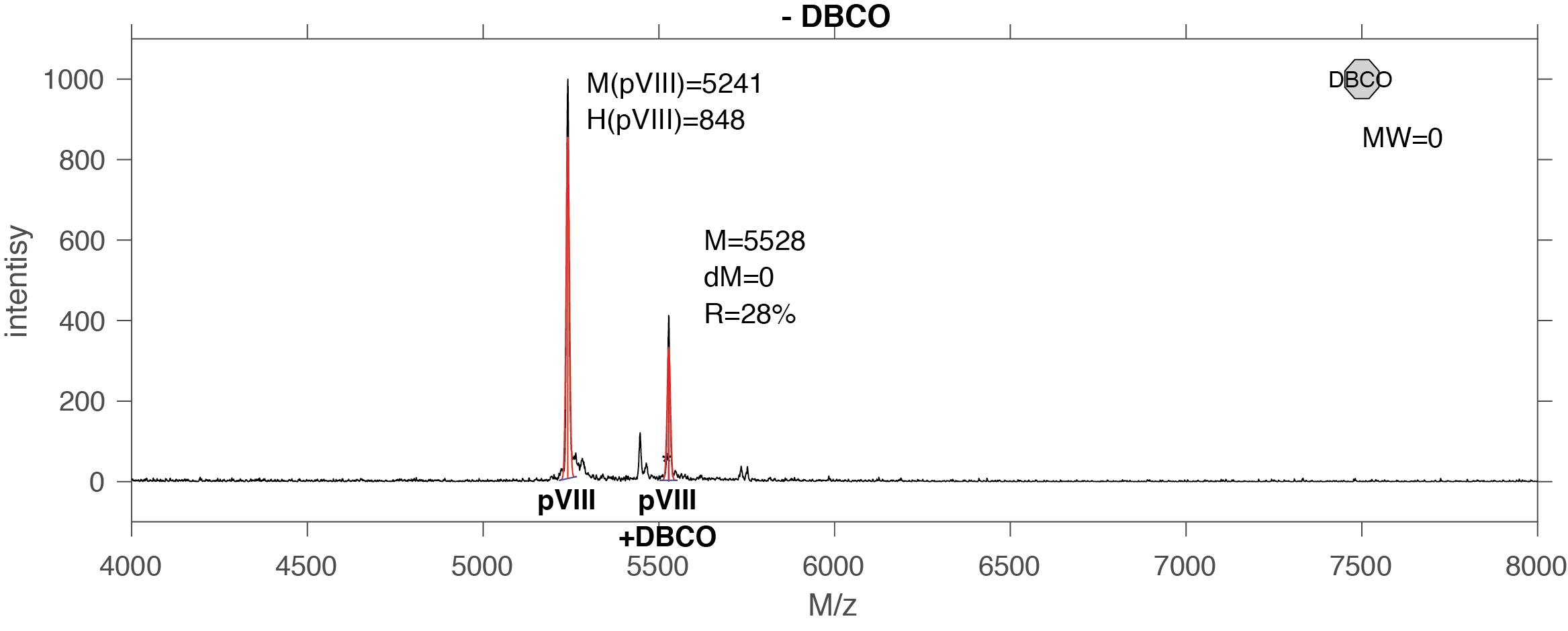
**

**
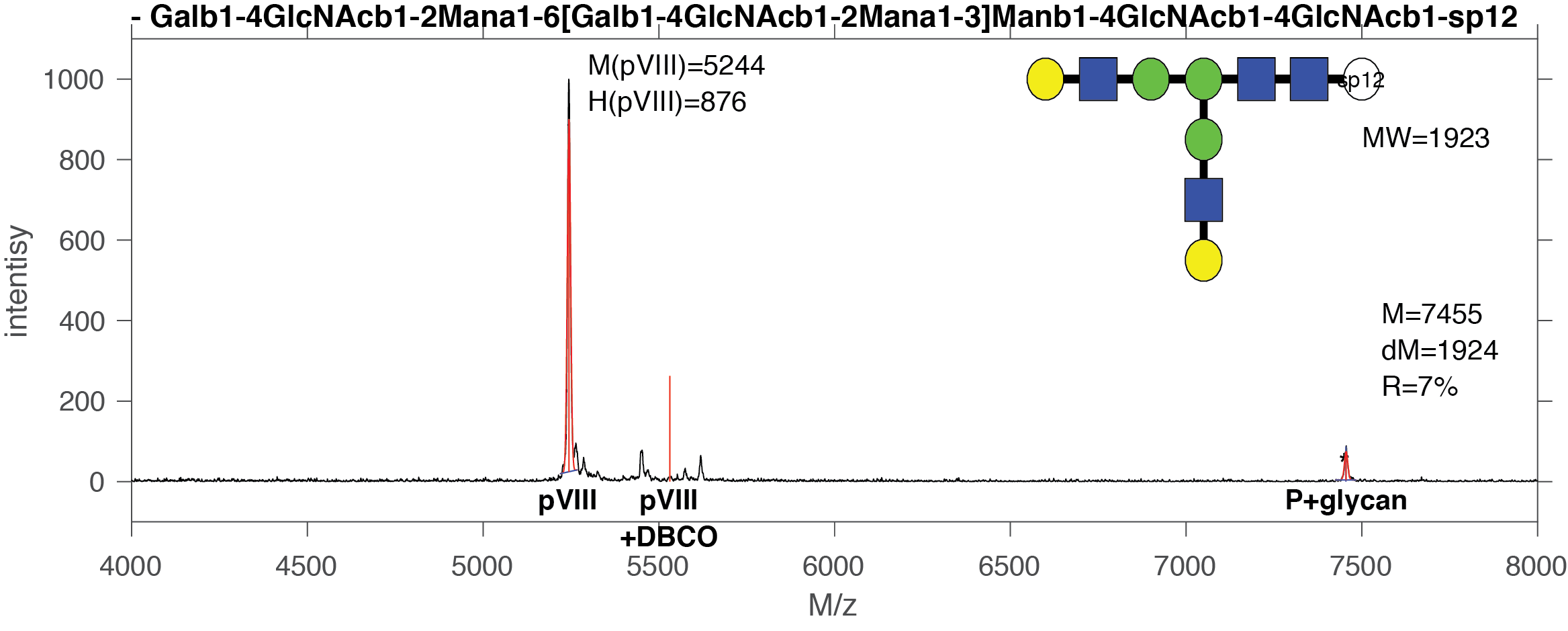
**

**SDB Number:** SDB13

**Barcode:** CTACTTTTCGCAATTCCTCTGAGTGTGGAGAAGAATGATCAGAAGACTTATCATGCGGGTGGAGGT

**Axis Name:** 10-[760]

**IUPAC:** Gal(b1-4)GlcNAc(b1-2)Man(a1-6)[Gal(b1-4)GlcNAc(b1-2)Man(a1-3)]Man(b1-4)GlcNAc(b1-4)GlcNAc(b1-Sp

**Maldi File:** TL-III-103-2-DBCO-SDB13_0002.txt and TL-III-107-2nd day+AzOH_0001.txt

**Density:** based on DBCO density was 28%


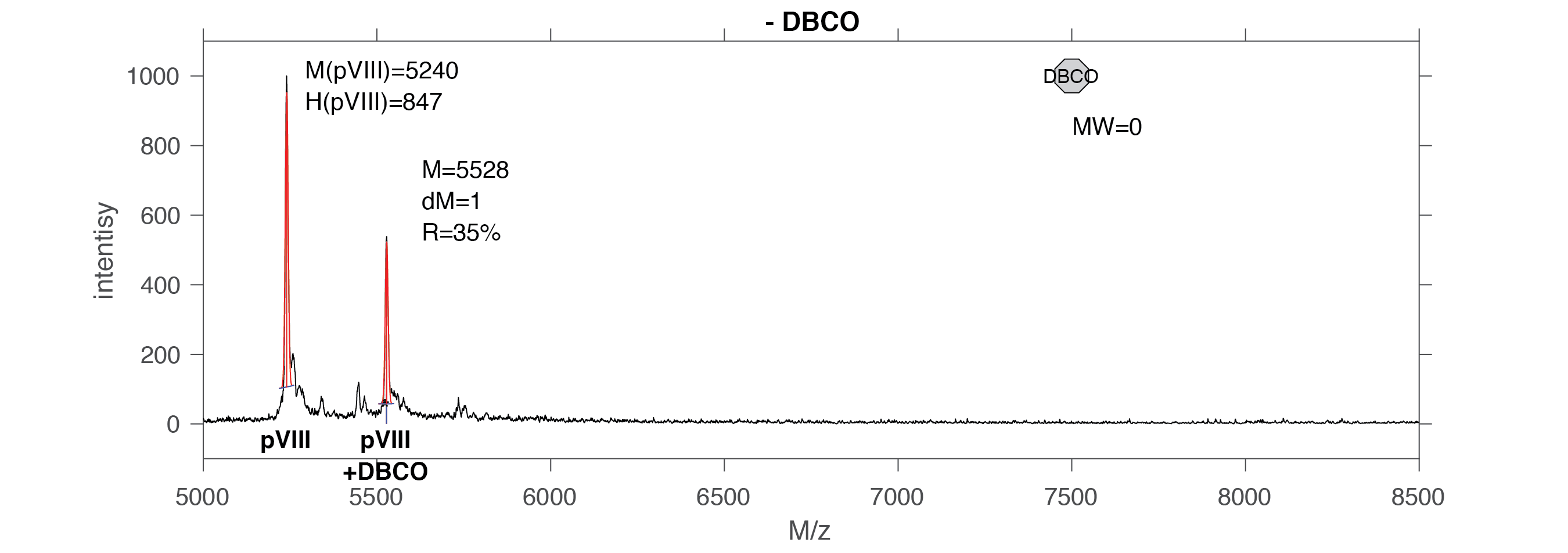


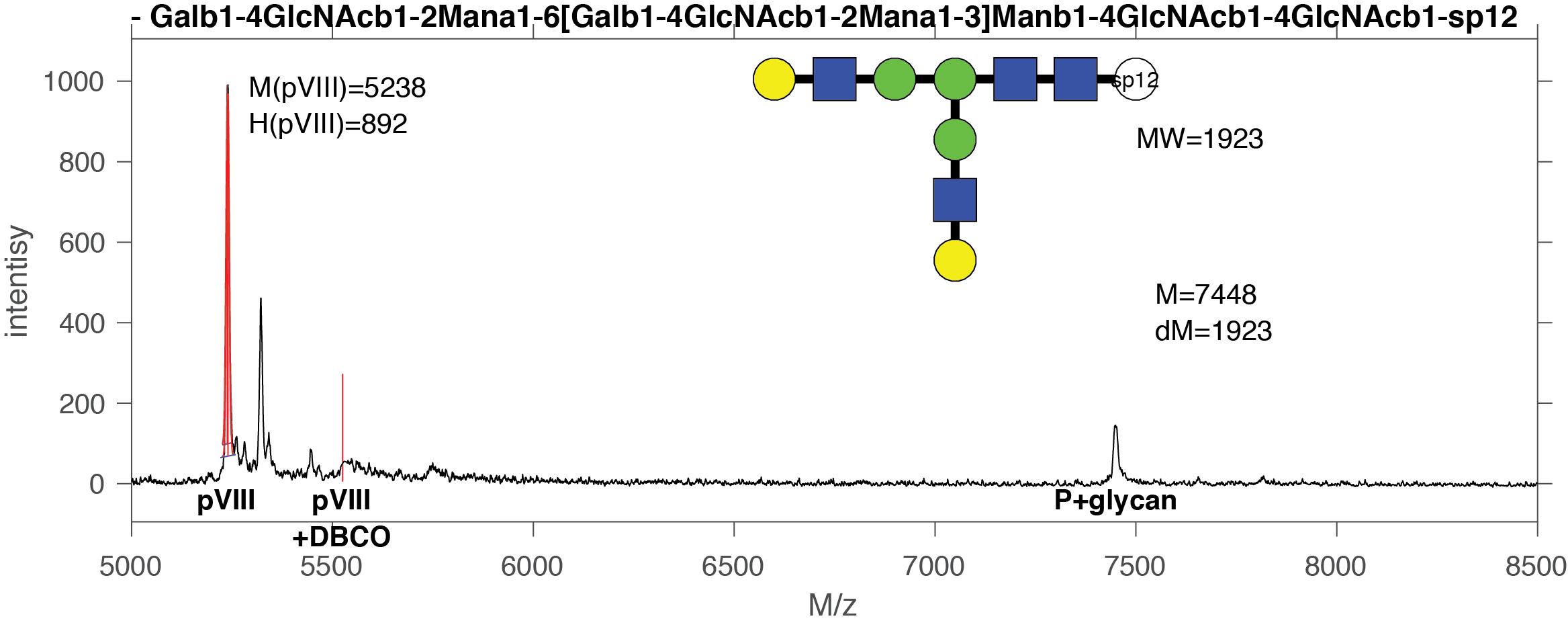


**SDB Number:** SDB221

**Barcode:** TTATTATTCGCAATTCCTTTAAGCGTGGAGAAAAATGACCAAAAAACGTACCACGCTGGGGGGGGG

**Axis Name:** 10-[1000]

**IUPAC:** Gal(b1-4)GlcNAc(b1-2)Man(a1-6)[Gal(b1-4)GlcNAc(b1-2)Man(a1-3)]Man(b1-4)GlcNAc(b1-4)GlcNAc(b1-Sp

**Maldi File:**  TL-IV-131-DBCO-SDB22160min_0002.txt and TL-IV-136-SDB221_1118_0002.txt

**Density:** based on DBCO density was 36%


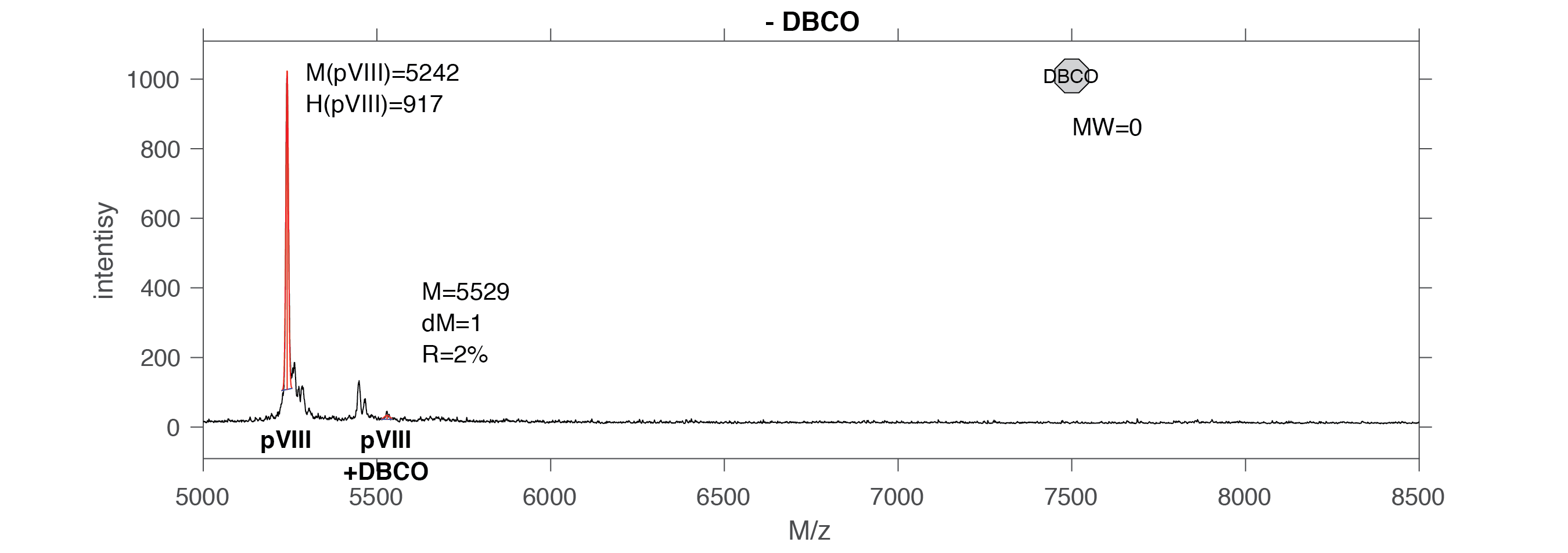


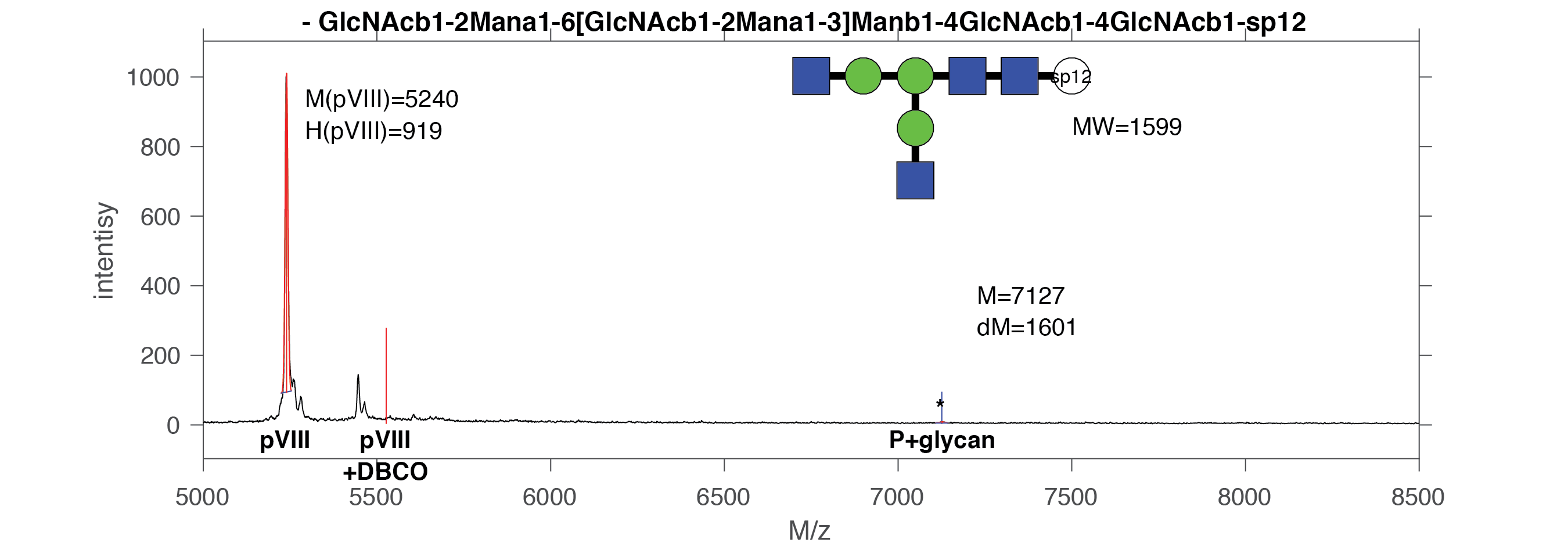


**SDB Number:** SDB90

**Barcode:** CTTCTATTTGCGATTCCGCTGAGTGTTGAAAAAAACGATCAAAAAACTTACCACGCCGGTGGGGGC

**Axis Name:** 6-[50]

**IUPAC:** GlcNAc(b1-2)Man(a1-6)[GlcNAc(b1-2)Man(a1-3)]Man(b1-4)GlcNAc(b1-4)GlcNAc(b1-Sp

**Maldi File:** TL-IV-135-DBCO-SDB90_0003.txt and TL-IV-135-SDB90_0001.txt

**Density:** based on DBCO density was 2%


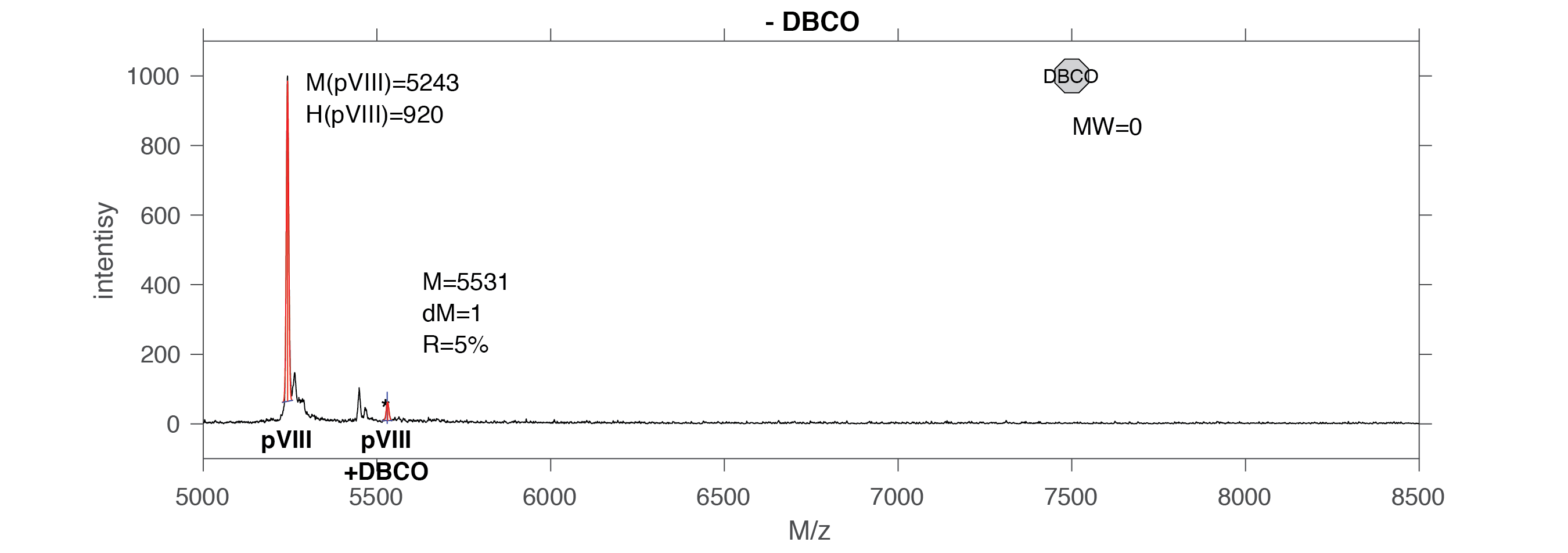


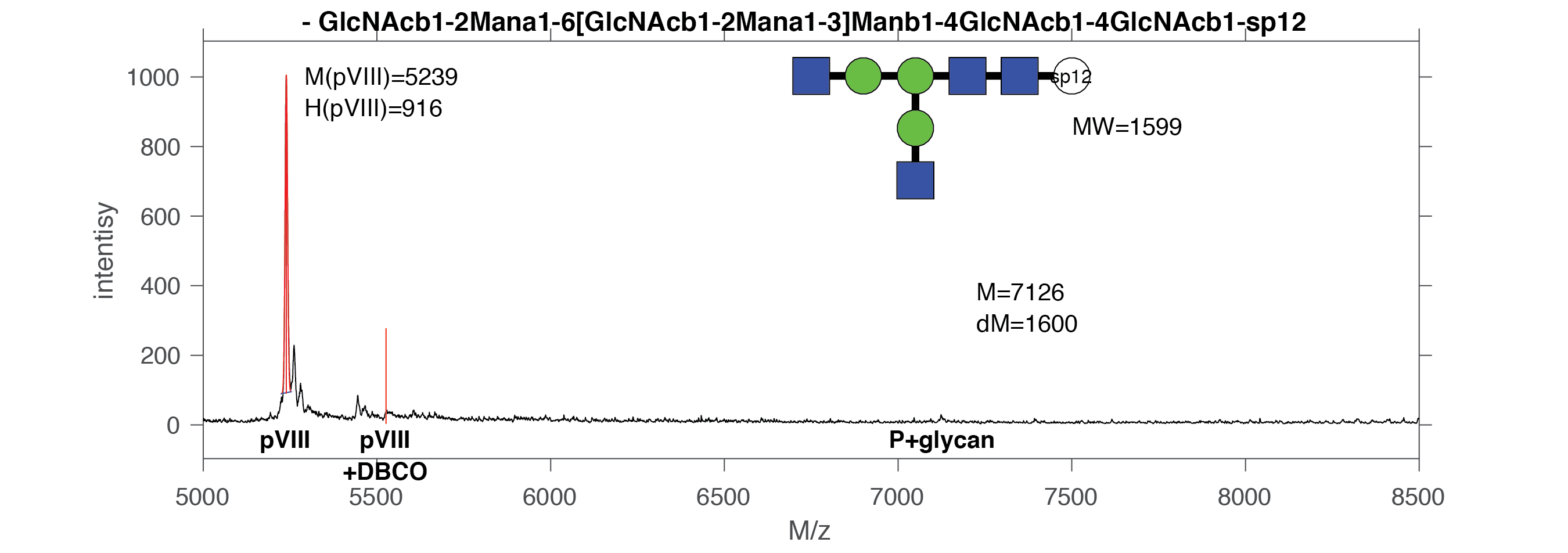


**SDB Number:** SDB102

**Barcode:** CTACTCTTTGCTATCCCTCTAAGTGTCGAGAAGAATGATCAGAAAACATACCATGCCGGGGGGGGT

**Axis Name:** 6-[150]

**IUPAC:** GlcNAc(b1-2)Man(a1-6)[GlcNAc(b1-2)Man(a1-3)]Man(b1-4)GlcNAc(b1-4)GlcNAc(b1-Sp

**Maldi File:** TL-IV-131-DBCO-SDB102_0003.txt and TL-IV-135-SDB102_0001.txt

**Density:** based on DBCO density was 5%


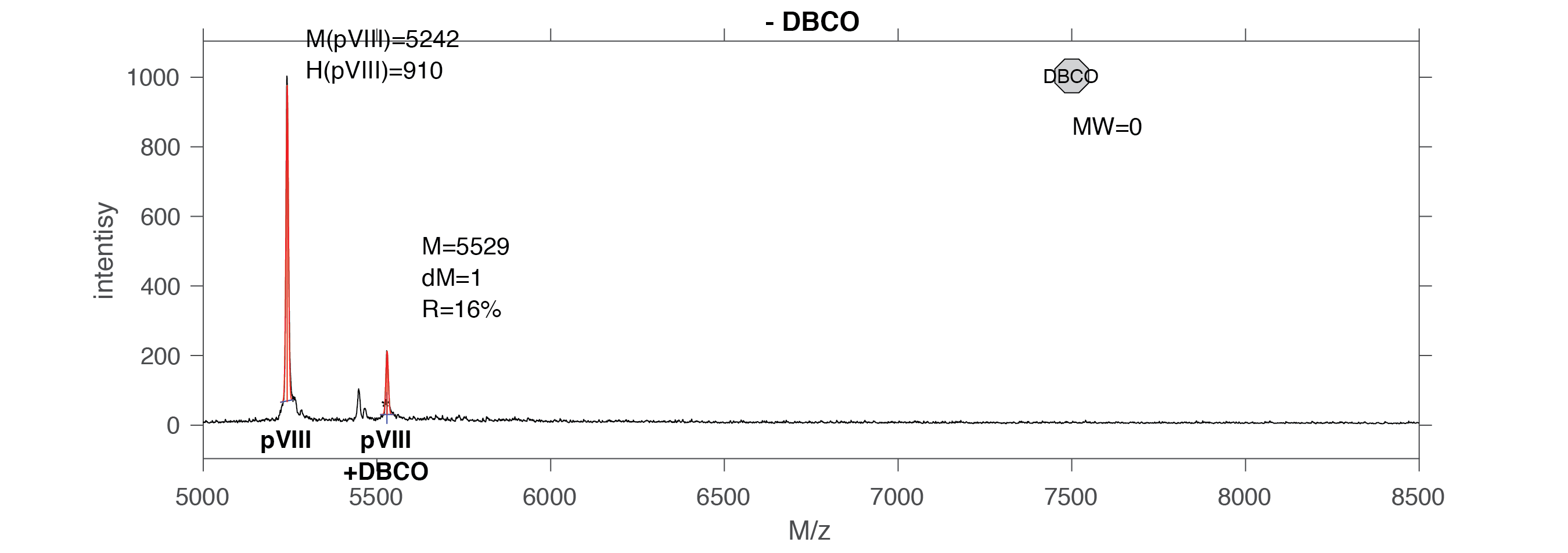


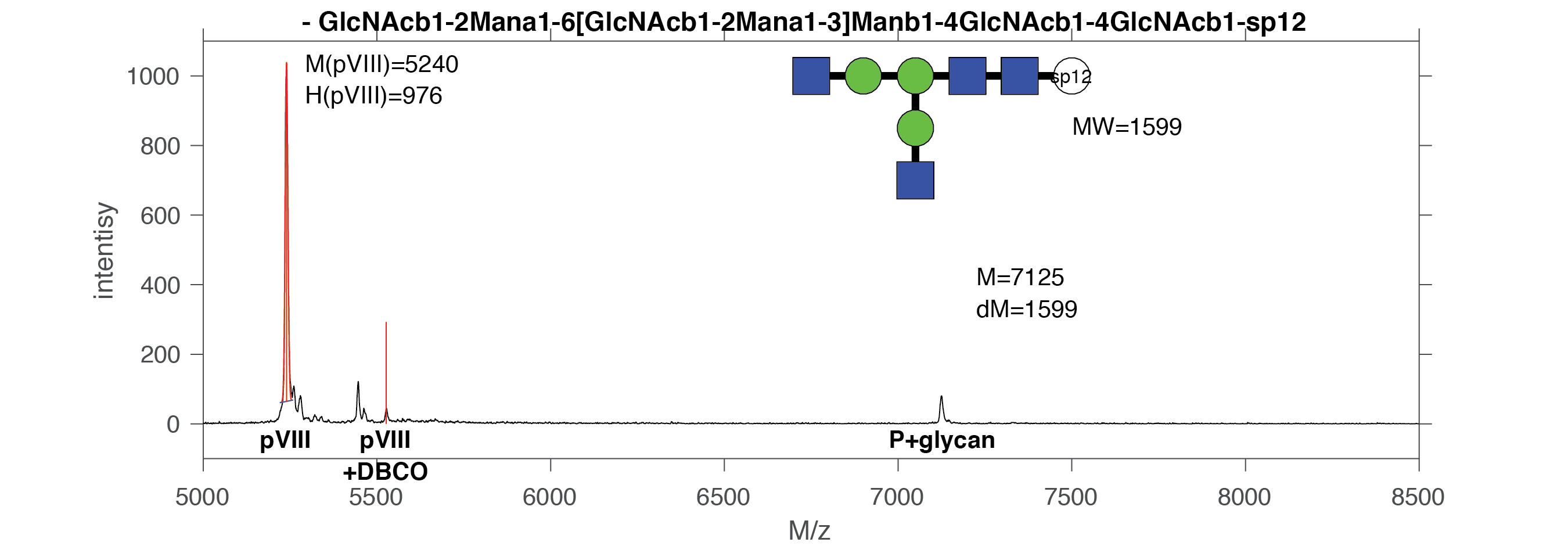


**SDB Number:** SDB155

**Barcode:** TTATTATTCGCAATTCCTTTAAGTGTGGAGAAGAATGATCAAAAGACGTACCATGCAGGCGGAGGT

**Axis Name:** 6-[500]

**IUPAC:** GlcNAc(b1-2)Man(a1-6)[GlcNAc(b1-2)Man(a1-3)]Man(b1-4)GlcNAc(b1-4)GlcNAc(b1-Sp

**Maldi File:** TL-IV-131-DBCO-SDB155-2nd_0002.txt and TL-IV-136-SDB155_0002.txt

**Density:** based on DBCO density was 15%


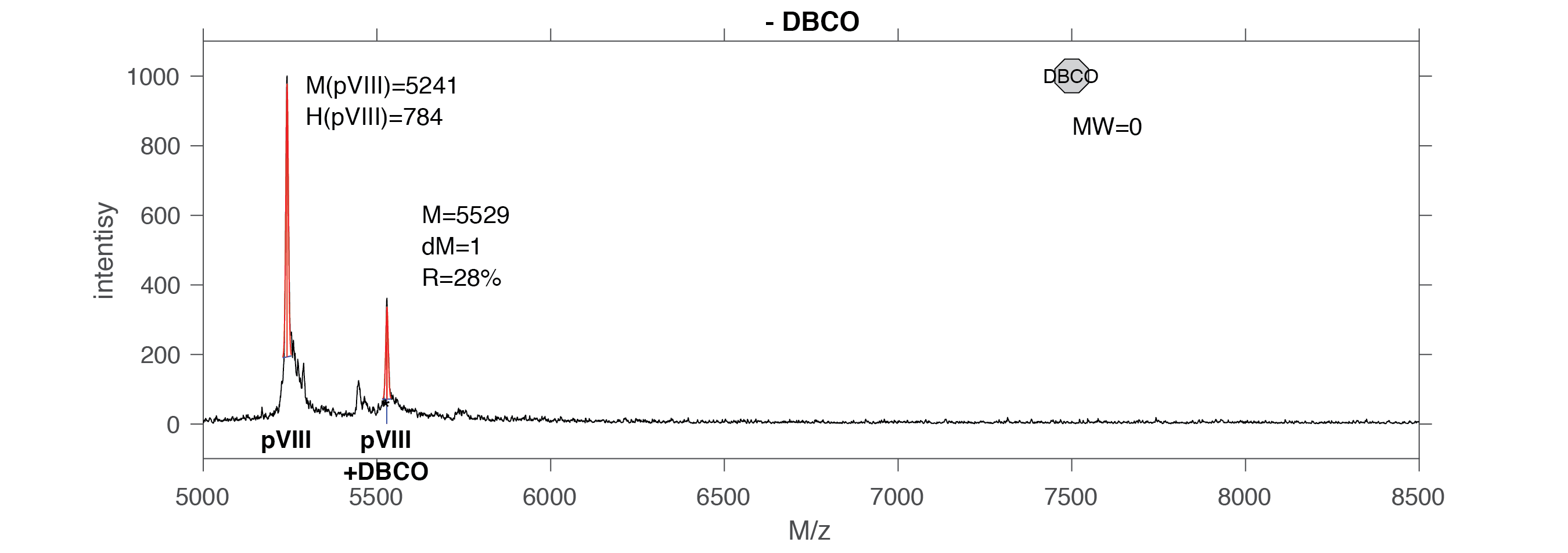


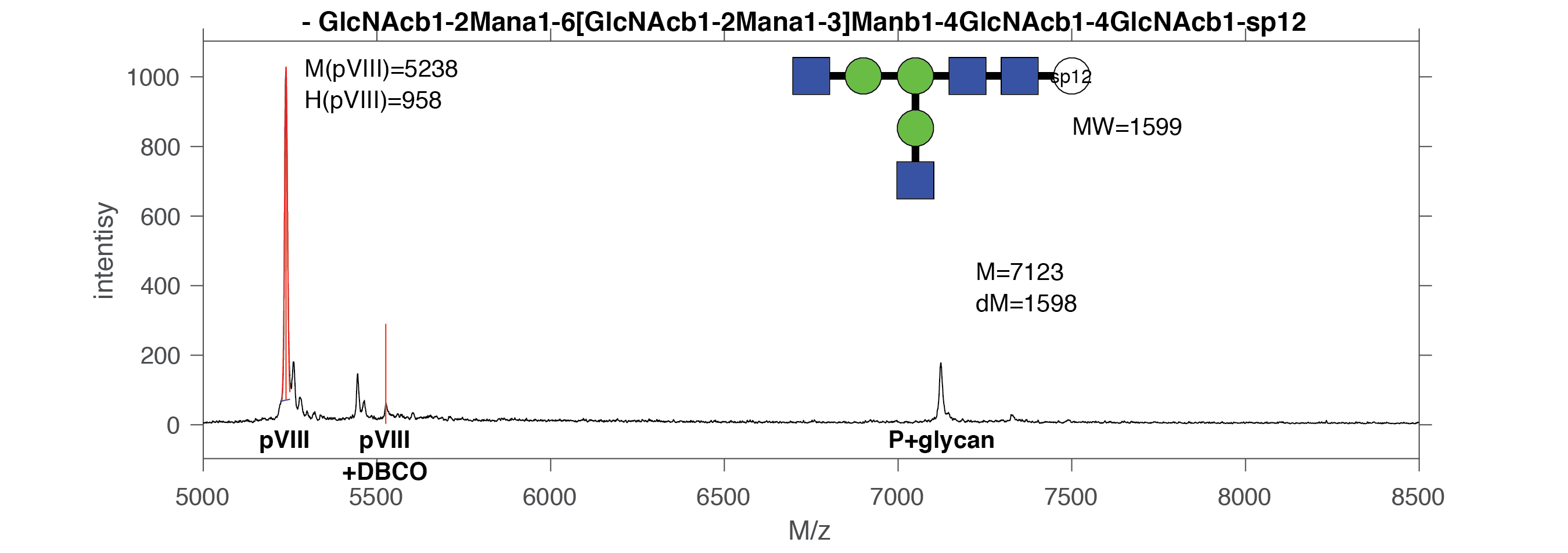


**SDB Number:** SDB94

**Barcode:** CTACTCTTTGCTATCCCTCTAAGTGTAGAAAAGAATGATCAAAAAACATATCATGCGGGTGGGGGT

**Axis Name:** 6-[750]

**IUPAC:** GlcNAc(b1-2)Man(a1-6)[GlcNAc(b1-2)Man(a1-3)]Man(b1-4)GlcNAc(b1-4)GlcNAc(b1-Sp

**Maldi File:** TL-IV-131-DBCO-SDB94_0006.txt and TL-IV-135-SDB94_0002.txt

**Density:** based on DBCO density was 28%


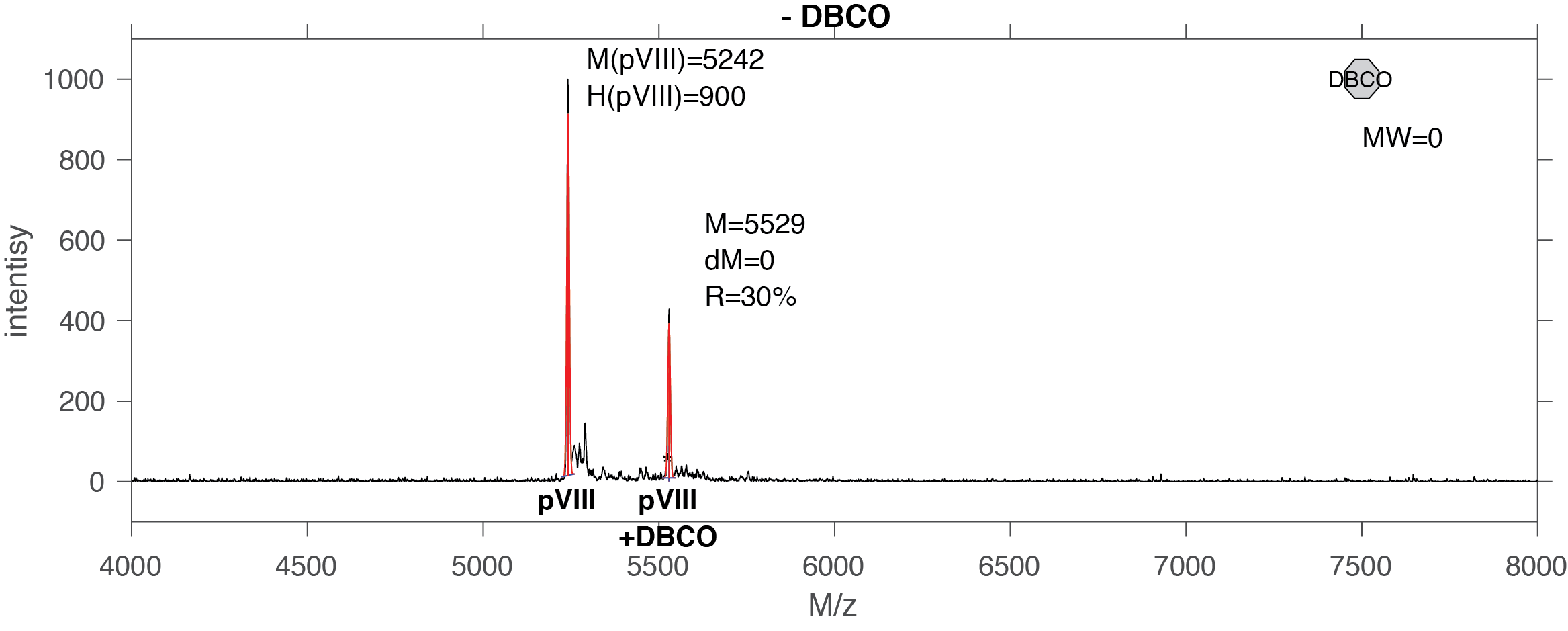


**
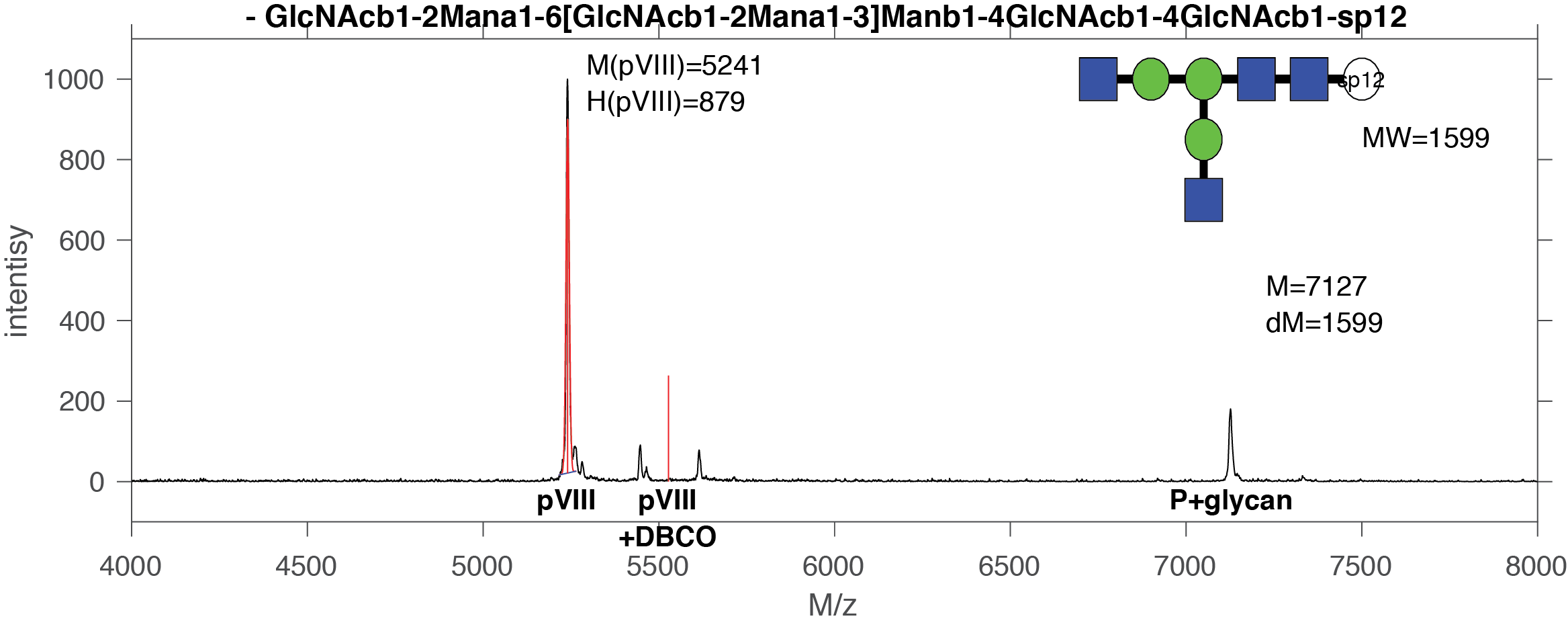
**

**SDB Number:** SDB29

**Barcode:** CTGCTATTTGCGATCCCGCTGAGTGTGGAGAAGAATGATCAGAAGACTTATCATGCGGGTGGAGGT

**Axis Name:** 6-[810]

**IUPAC:** GlcNAc(b1-2)Man(a1-6)[GlcNAc(b1-2)Man(a1-3)]Man(b1-4)GlcNAc(b1-4)GlcNAc(b1-Sp

**Maldi File:** TL-III-113-DBCO-SDB29_0002.txt and TL-III-113+AzOH_0001.txt

**Density:** based on DBCO density was 30%


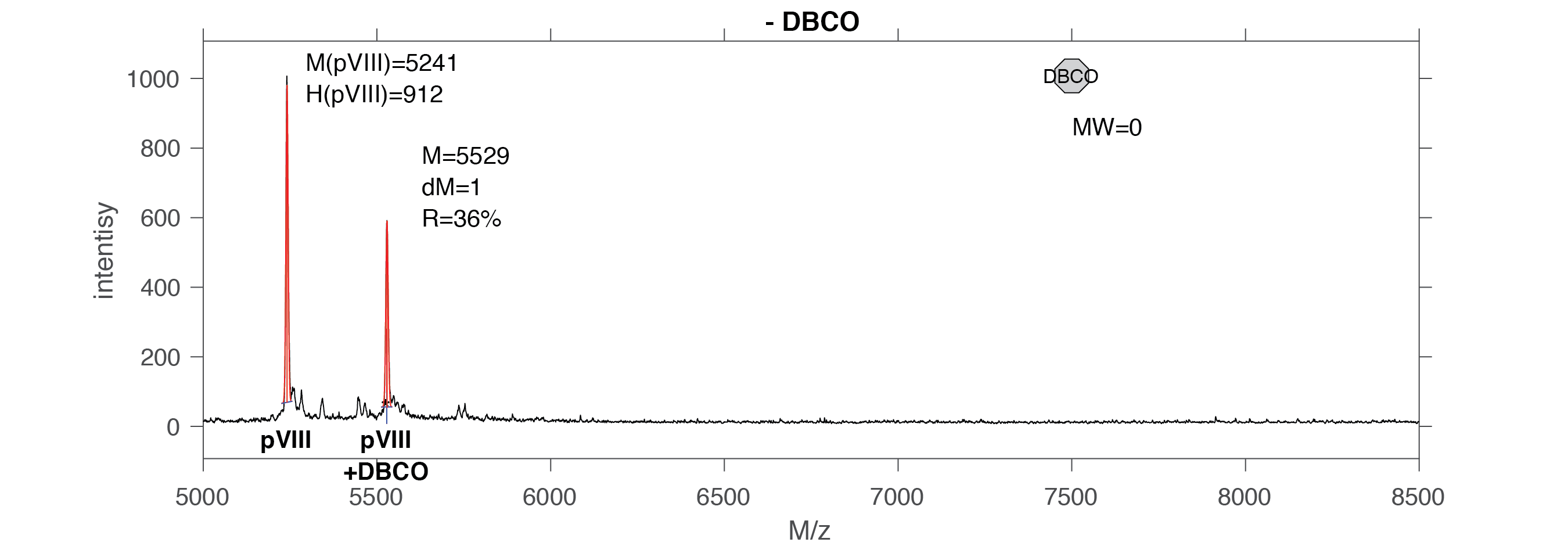


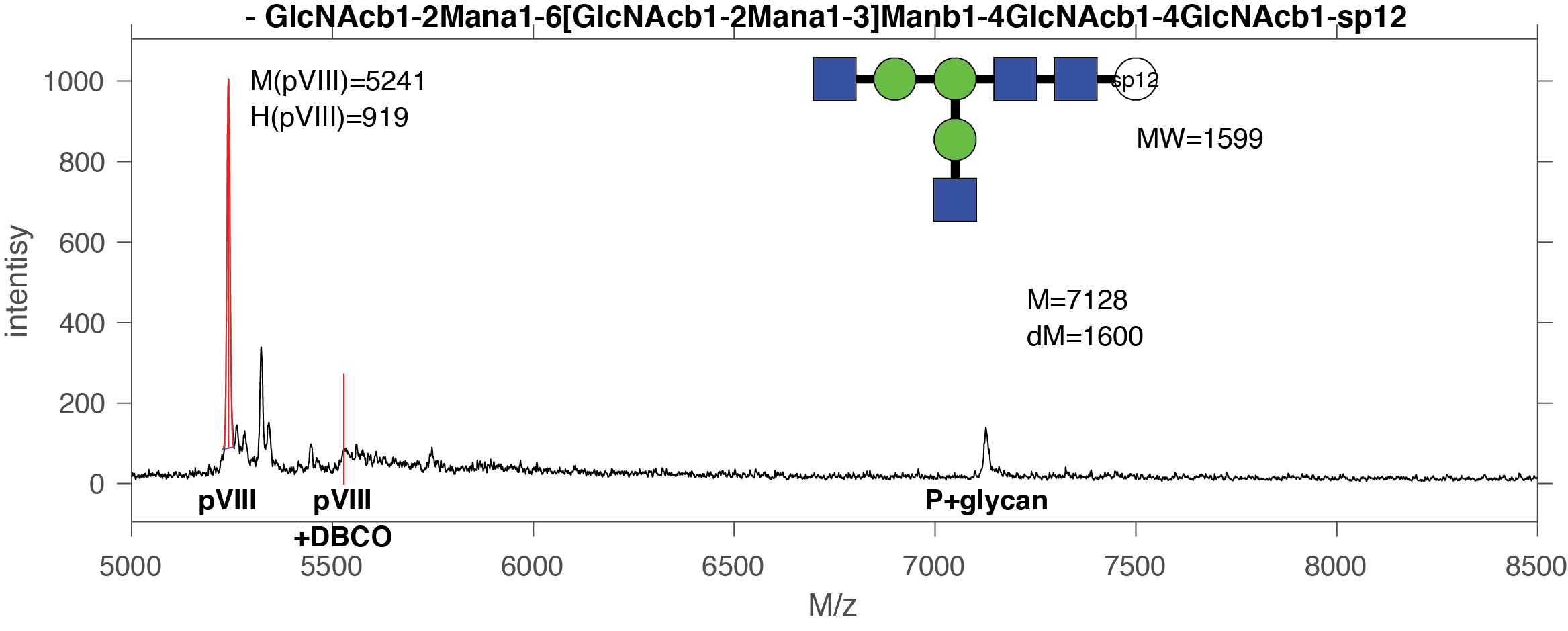


**SDB Number:** SDB225

**Barcode:** CTTCTCTTTGCTATTCCTCTTAGCGTGGAAAAAAACGATCAAAAAACTTACCATGCCGGGGGTGGT

**Axis Name:** 6-[1000]

**IUPAC:** GlcNAc(b1-2)Man(a1-6)[GlcNAc(b1-2)Man(a1-3)]Man(b1-4)GlcNAc(b1-4)GlcNAc(b1-Sp

**Maldi File:** TL-IV-131-DBCO-SDB225-2nd_0001.txt and TL-IV-136-SDB225_1118_0002.txt

**Density:** based on DBCO density was 36%


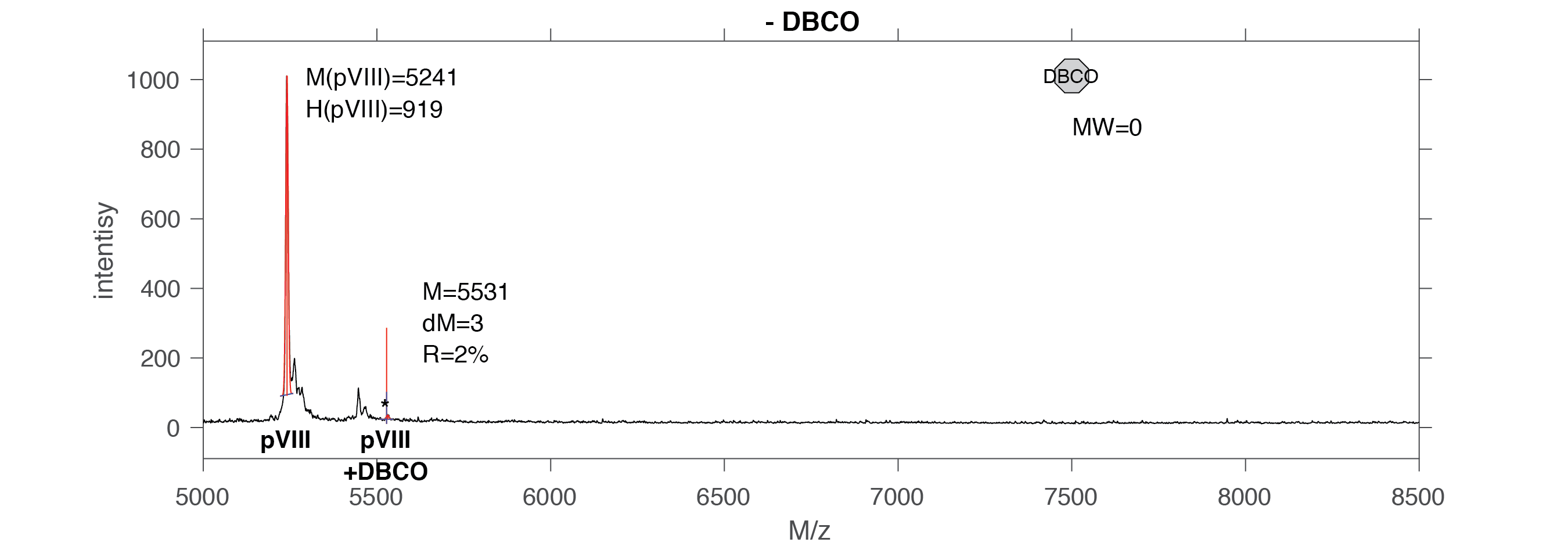


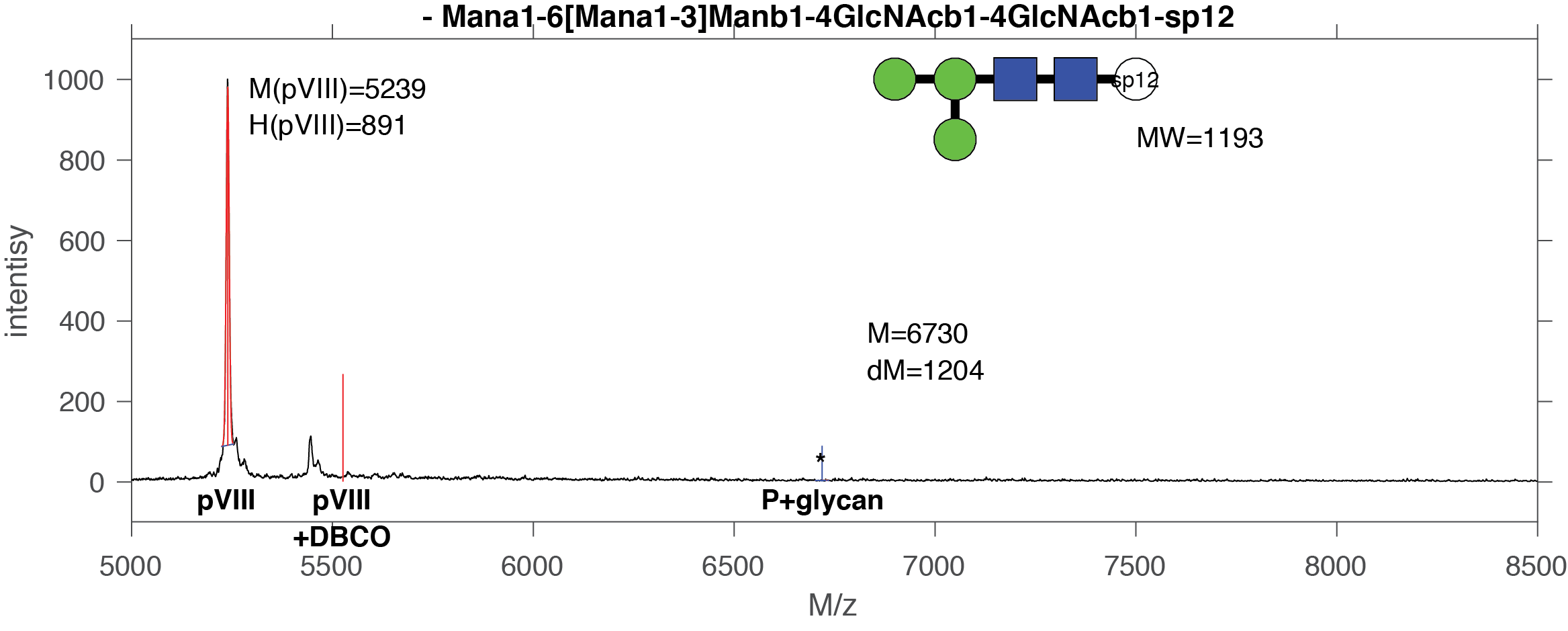


**SDB Number:** SDB224

**Barcode:** TTATTATTCGCAATTCCTTTAAGTGTGGAGAAGAACGATCAGAAGACCTATCACGCCGGGGGTGGA

**Axis Name:** 11-[50]

**IUPAC:** Man(a1-6)[Man(a1-3)]Man(b1-4)GlcNAc(b1-4)GlcNAc(b1-Sp

**Maldi File:** TL-IV-150-DBCO-SDB224_0001.txt and TL-IV-135-SDB224_0001.txt

**Density:** based on DBCO density was 2%


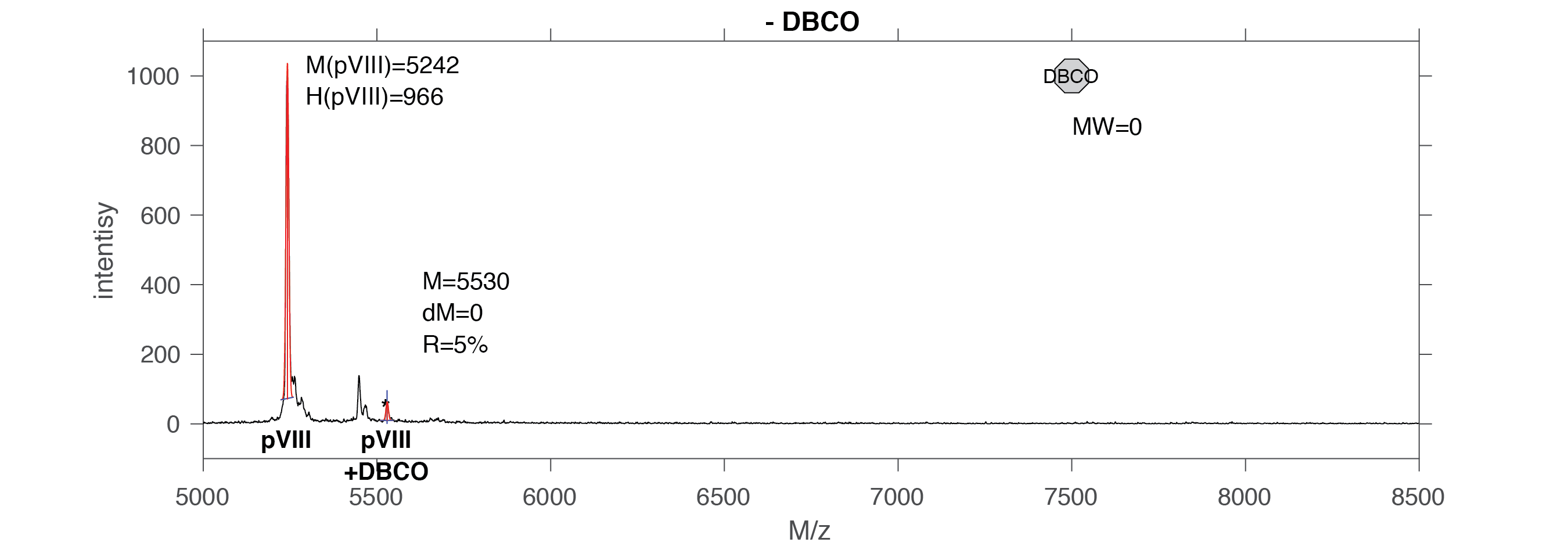


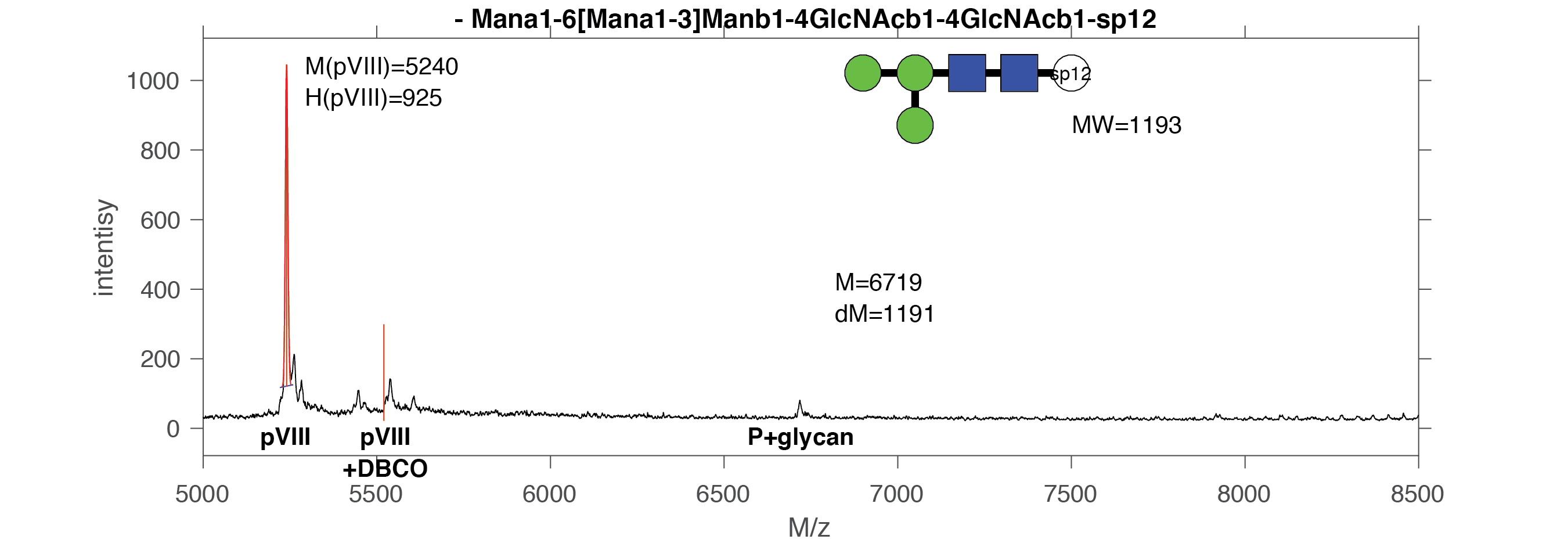


**SDB Number:** SDB104

**Barcode:** CTGCTATTTGCCATCCCACTAAGTGTGGAGAAAAATGATCAGAAAACTTATCATGCCGGAGGTGGG

**Axis Name:** 11-[150]

**IUPAC:** Man(a1-6)[Man(a1-3)]Man(b1-4)GlcNAc(b1-4)GlcNAc(b1-Sp

**Maldi File:** TL-IV-131-DBCO-SDB104_0004.txt and TL-IV-151_0002.txt

**Density:** based on DBCO density was 5%


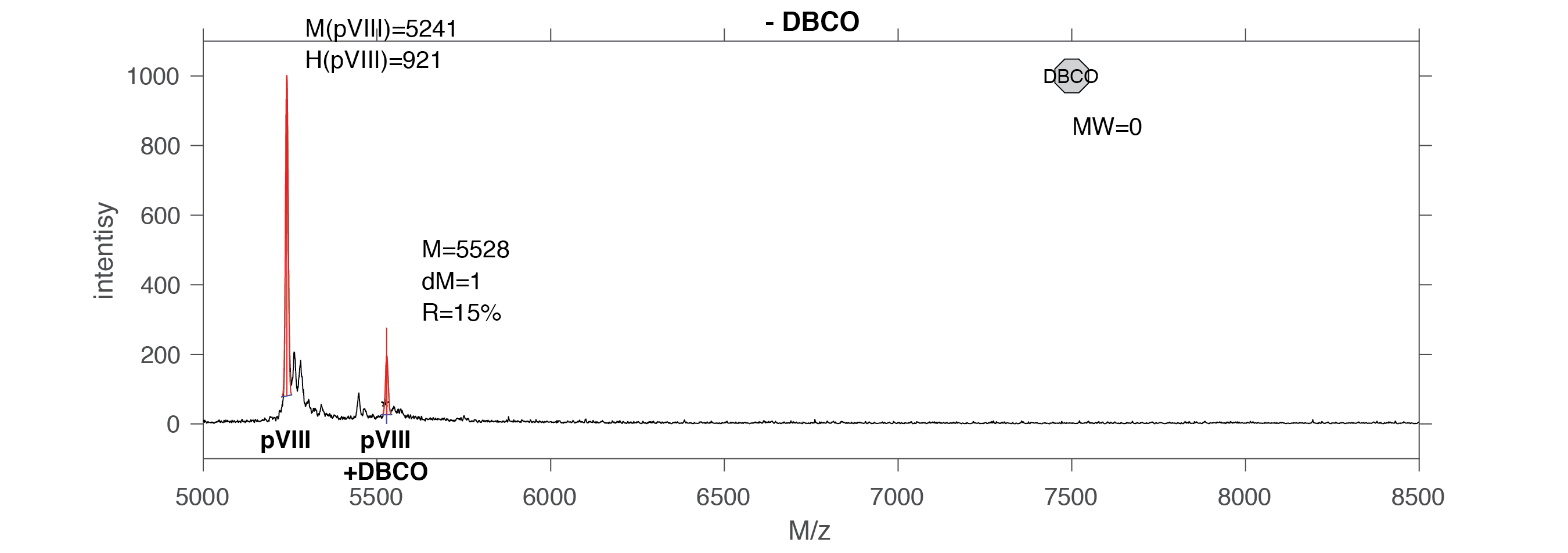


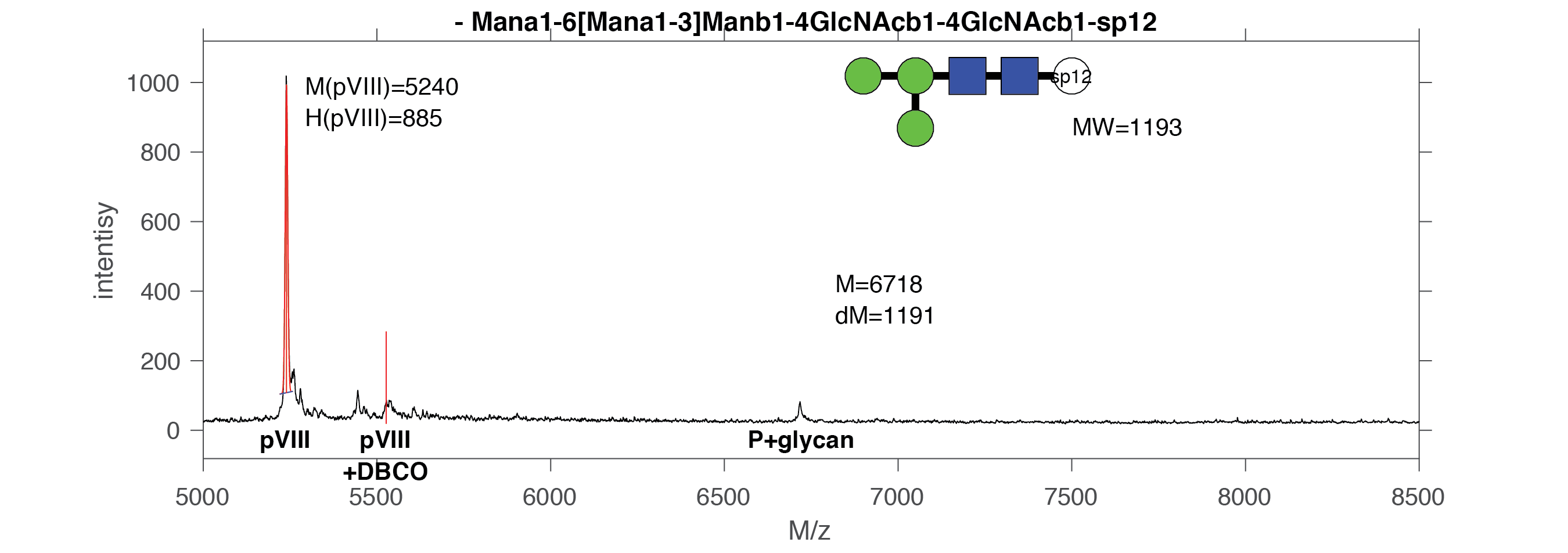


**SDB Number:** SDB136

**Barcode:** CTTCTTTTTGCGATTCCGCTCAGTGTAGAGAAGAACGATCAAAAGACGTATCATGCTGGAGGAGGT

**Axis Name:** 11-[500]

**IUPAC:** Man(a1-6)[Man(a1-3)]Man(b1-4)GlcNAc(b1-4)GlcNAc(b1-Sp

**Maldi File:** TL-IV-131-DBCO-SDB136_0002.txt and TL-IV-152_0001.txt

**Density:** based on DBCO density was 15%


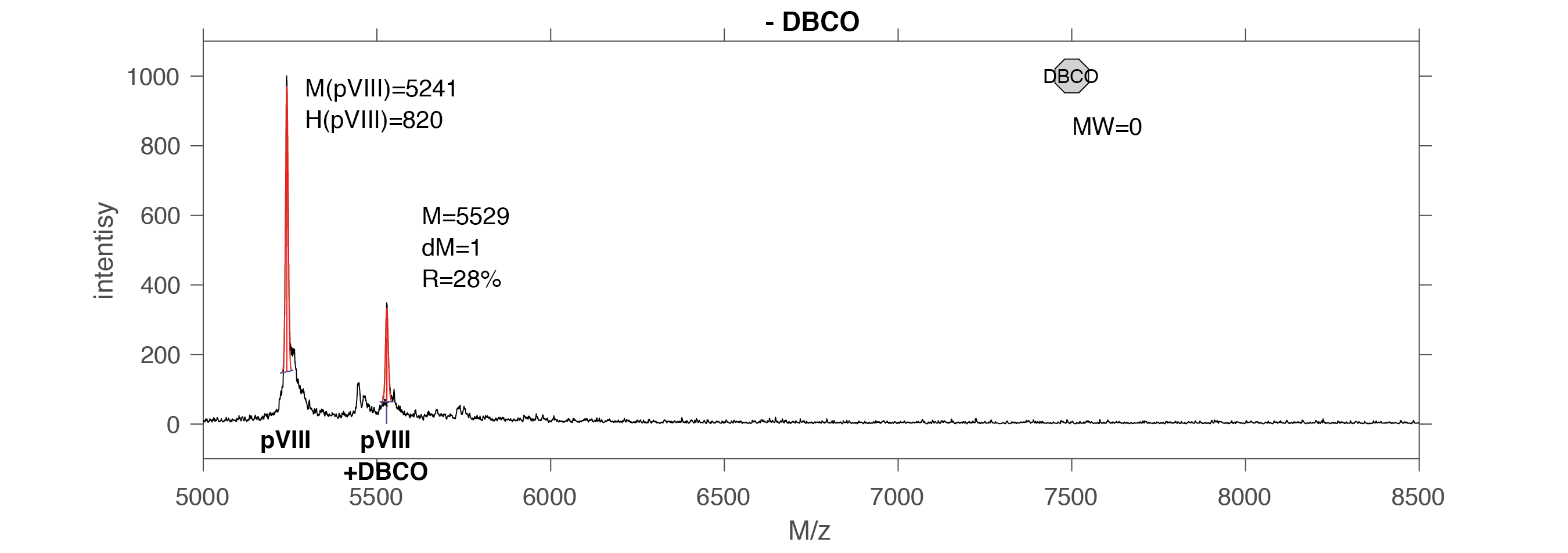


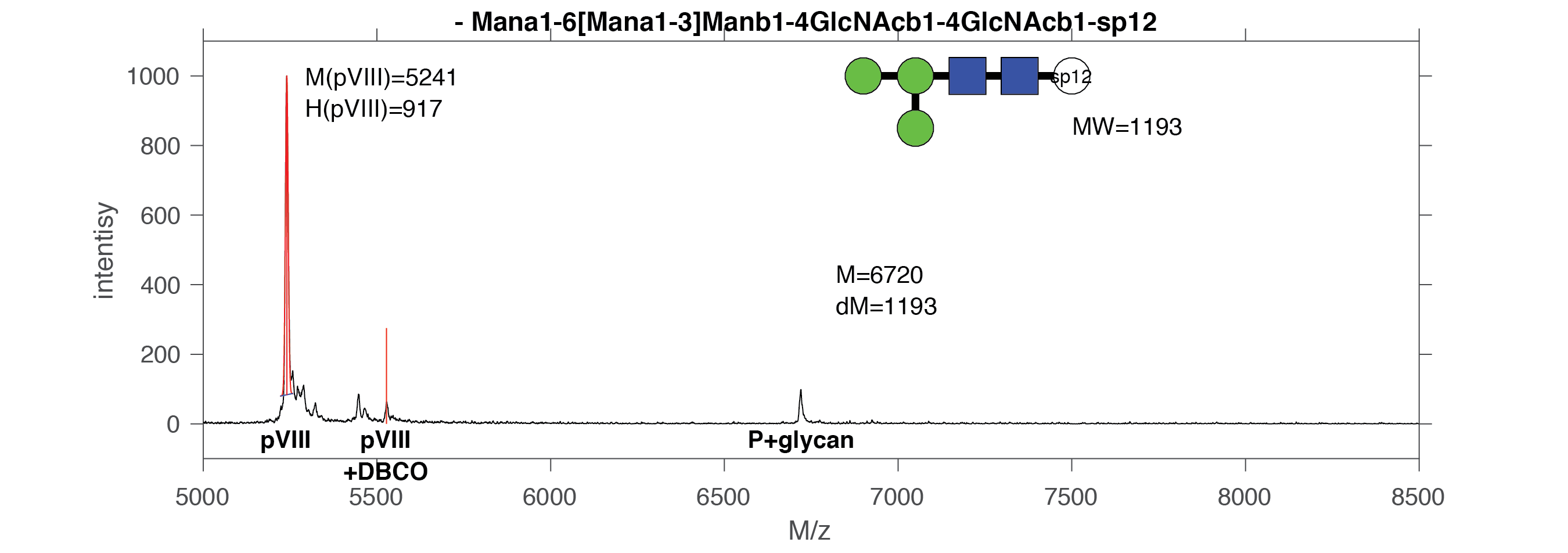


**SDB Number:** SDB95

**Barcode:** CTTCTCTTTGCGATACCGCTAAGTGTAGAAAAGAACGACCAAAAGACCTATCACGCGGGTGGTGGG

**Axis Name:** 11-[750]

**IUPAC:** Man(a1-6)[Man(a1-3)]Man(b1-4)GlcNAc(b1-4)GlcNAc(b1-Sp

**Maldi File:** TL-IV-131-DBCO-SDB95_0004.txt and TL-IV-153_0002.txt

**Density:** based on DBCO density was 28%


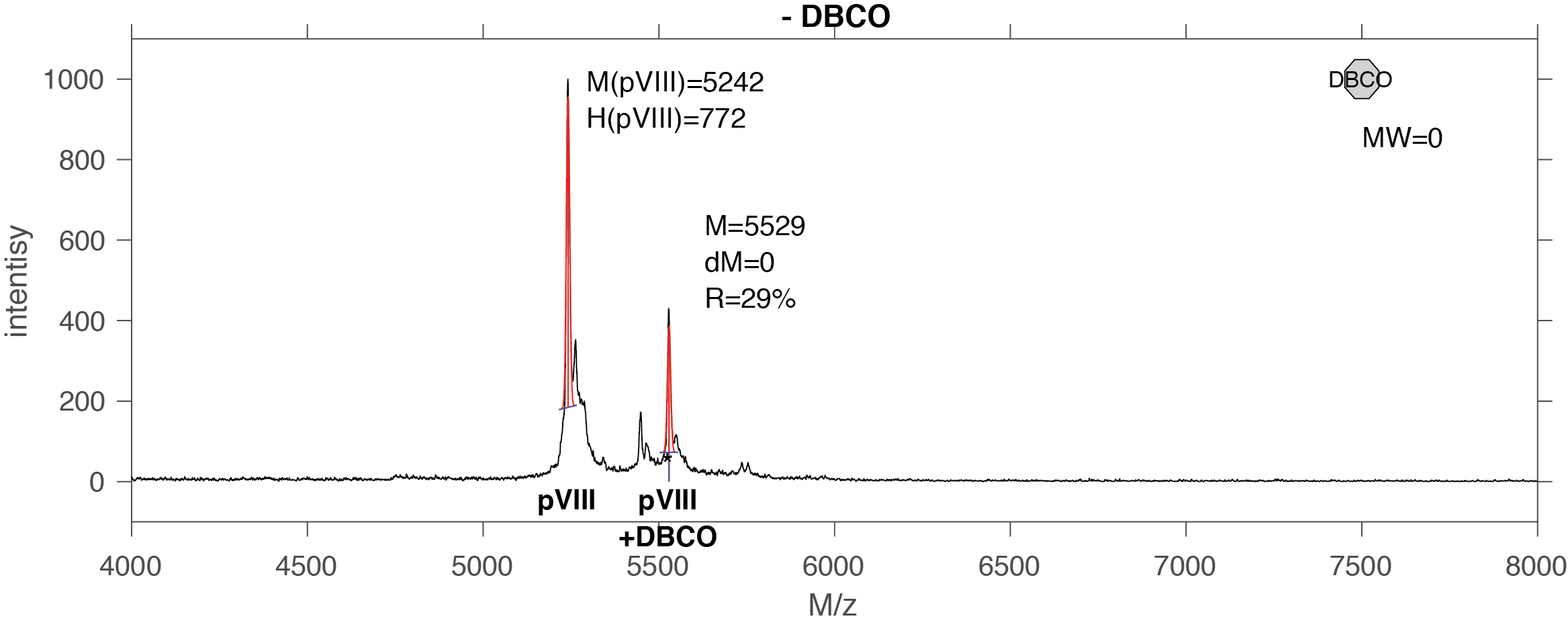


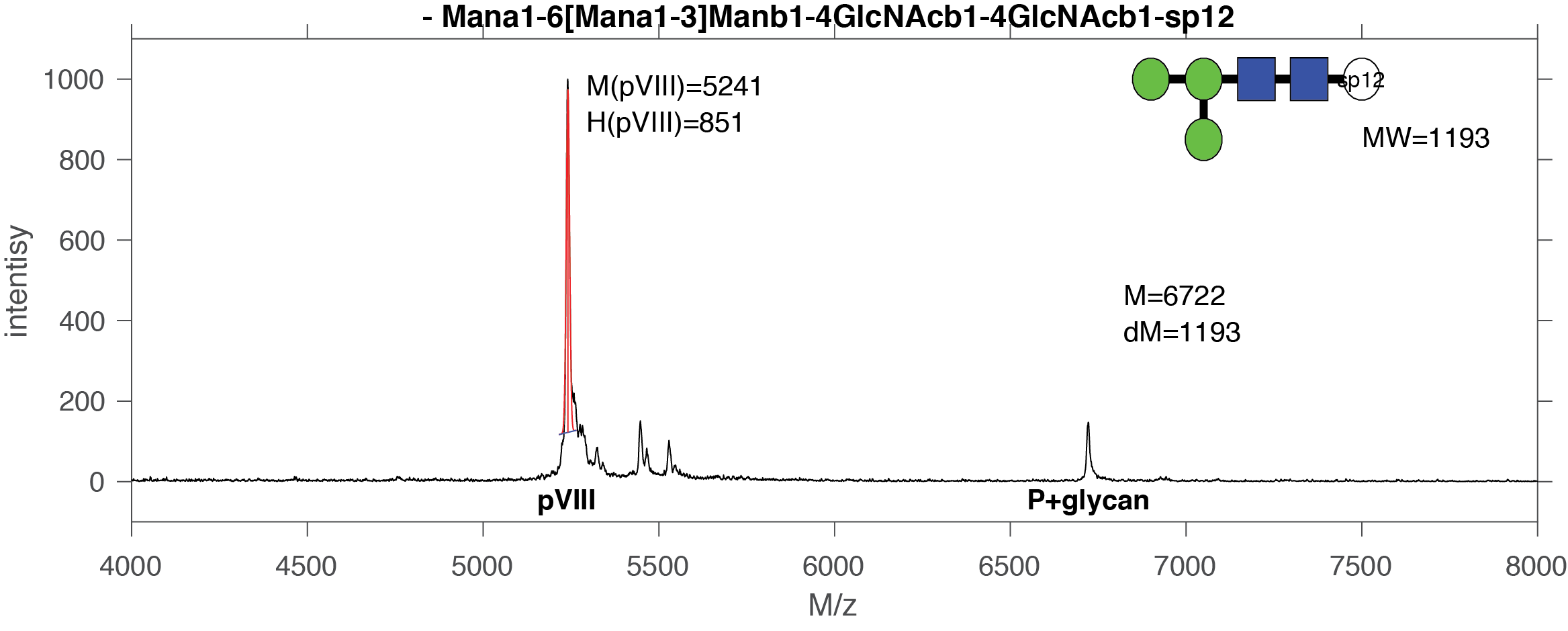


**SDB Number:** SDB20

**Barcode:** CTGCTCTTTGCCATCCCGCTTAGTGTGGAGAAGAATGATCAGAAGACTTATCATGCGGGTGGAGGT

**Axis Name:** 11-[780]

**IUPAC:** Man(a1-6)[Man(a1-3)]Man(b1-4)GlcNAc(b1-4)GlcNAc(b1-Sp

**Maldi File:** TL-III-103-3-DBCO-SDB20_0002.txt and TL-III-143_0001.txt

**Density:** based on DBCO density was 29%


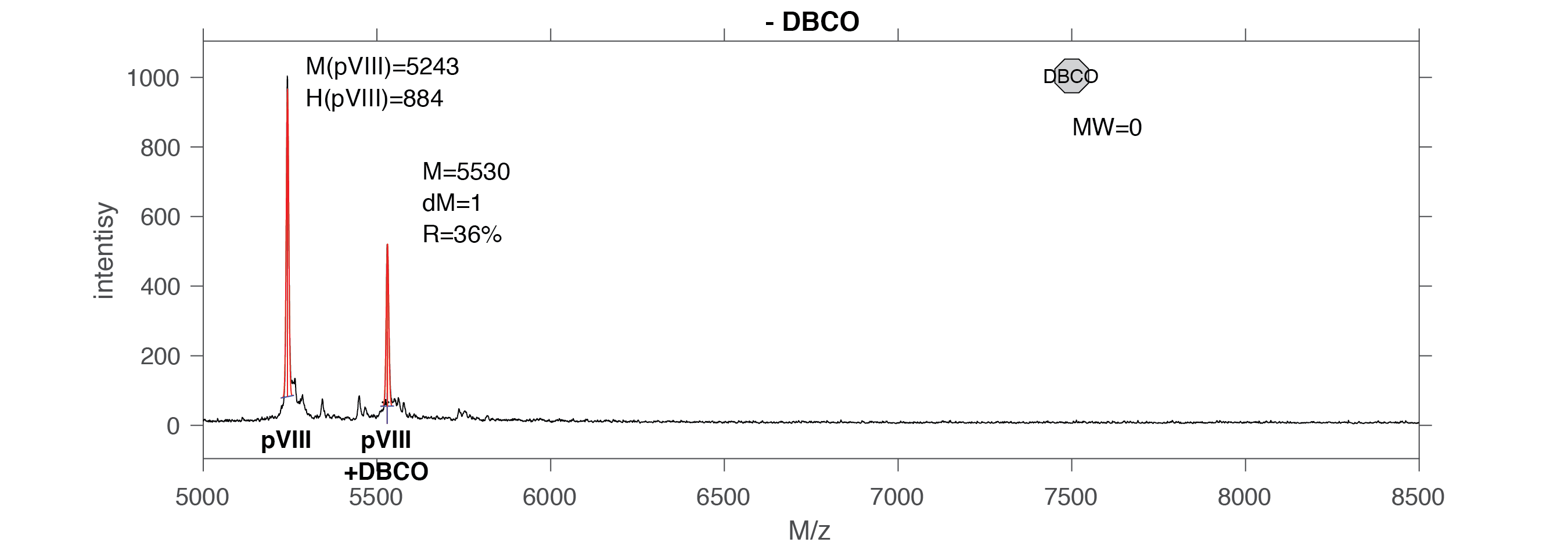


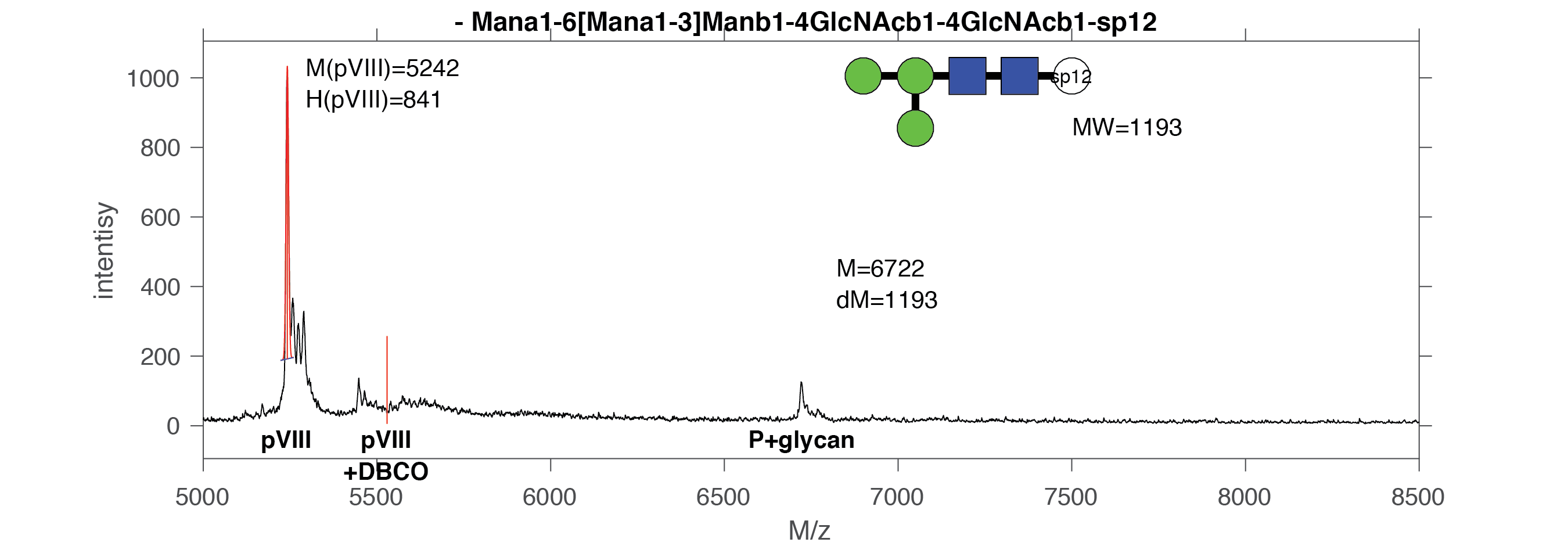


**SDB Number:** SDB222

**Barcode:** CTGCTATTTGCTATCCCGCTGAGTGTTGAGAAAAACGATCAAAAAACGTACCACGCTGGAGGTGGT

**Axis Name:** 11-[1000]

**IUPAC:** Man(a1-6)[Man(a1-3)]Man(b1-4)GlcNAc(b1-4)GlcNAc(b1-Sp

**Maldi File:** TL-IV-131-DBCO-SDB222-2nd_0003.txt and TL-IV-154_0002.txt

**Density:** based on DBCO density was 36%
